# Reductive radical-initiated 1,2-C migration assisted by an azidyl group†

**DOI:** 10.1039/d0sc02559c

**Published:** 2020-07-07

**Authors:** Xueying Zhang, Zhansong Zhang, Jin-Na Song, Zikun Wang

**Affiliations:** Jilin Province Key Laboratory of Organic Functional Molecular Design & Synthesis, Department of Chemistry, Northeast Normal University Changchun 130024 China wangzk076@nenu.edu.cn; School of Life Science, Jilin University Changchun 130012 China songjn2010@jlu.edu.cn

## Abstract

We report here a novel reductive radical-polar crossover reaction that is a reductive radical-initiated 1,2-C migration of 2-azido allyl alcohols enabled by an azidyl group. The reaction tolerates diverse migrating groups, such as alkyl, alkenyl, and aryl groups, allowing access to *n*+1 ring expansion of small to large rings. The possibility of directly using propargyl alcohols in one-pot is also described. Mechanistic studies indicated that an azidyl group is a good leaving group and provides a driving force for the 1,2-C migration.

## Abstract 2

We report here a novel reductive radical-polar crossover reaction that is a reductive radical-initiated 1,2-C migration of 2-azido allyl alcohols enabled by an azidyl group.

## Graphical abstract

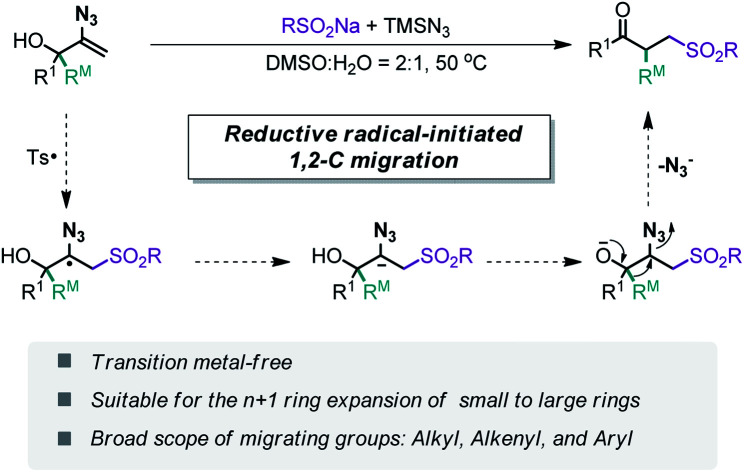

Since the groups of Ryu and Sonoda described the reductive radical-polar crossover (RRPCO) concept in the 1990s,^1^ it has attracted considerable attention in modern organic synthesis.^2^ By using this concept, a variety of complex molecules could be assembled in a fast step-economic fashion which is not possible using either radical or polar chemistry alone. However, only two RRPCO reaction modes are known to date: nucleophilic addition and nucleophilic substitution (Fig. 1A). The first RRPCO reaction is the nucleophilic addition of organometallic species, which is generated *in situ* from the reduction of a strong reducing metal with a carbon-centered radical intermediate and cations (E^+^ = H^+^, I^+^, Br^+^, path 1).^3^ However, the necessity for a large amount of harmful and strong reducing metals has greatly limited the scope and functional group tolerance of the reaction. Recently, photoredox catalysis has not only successfully overcome the shortcomings of using toxic strong reducing metals in the RRPCO reaction,^4^ but also enabled the development of several new RRPCO reaction types, including the nucleophilic addition with carbonyl compounds or carbon dioxide (path 2),^5^ the cyclization of alkyl halides/tosylates (path 3),^6^ and β-fluorine elimination (path 4).^7^ Although the RRPCO reaction has been greatly advanced by photoredox catalysis, it is still in its infancy, and the development of a novel RRPCO reaction is of great importance.

**Fig. 1:**
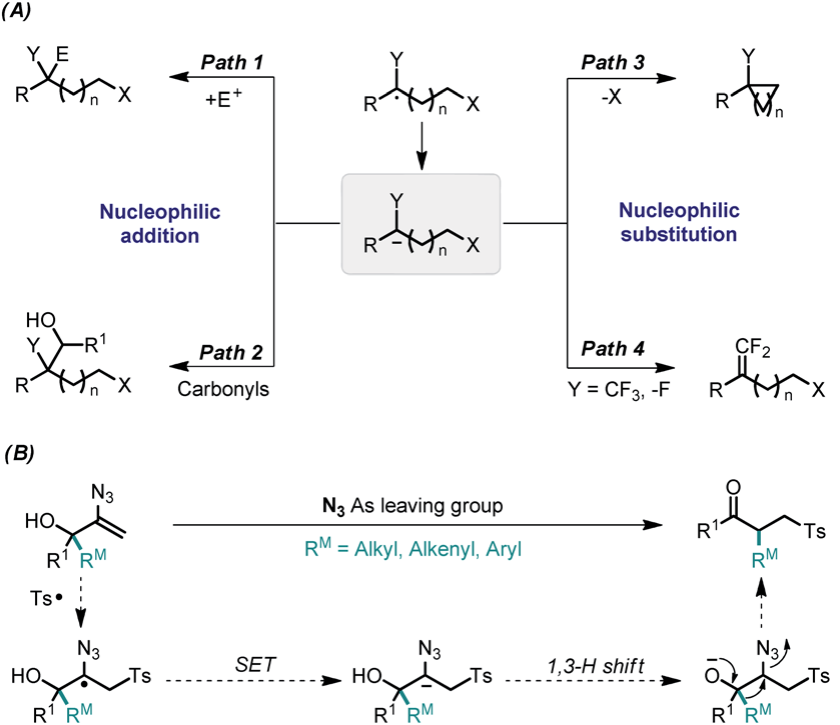
(A) Reductive radical-polar crossover reactions; (B) this work: reductive radical-initiated 1,2-C migration assisted by an azidyl group.

Herein, we wish to report a new type of reductive radical-polar crossover cascade reaction that is the reductive radical-initiated 1,2-C migration under metal-free conditions (Fig. 1B). The development of this approach is not only to further expand the application of the RRPCO reaction, but also to solve the problems associated with the oxidative radical-initiated 1,2-C migration, such as the necessity for an oxidant and/or transition metal for the oxidative termination of the radicals, and also required sufficient ring strain to avoid the generation of epoxy byproducts.^8^ To realize this reaction, a driving force is needed to drive the 1,2-C migration after reductive termination, to avoid the otherwise inevitable protonation of the generated anion.^9^ Inspired by the leaving group-induced semipinacol rearrangement,^10^ we envisaged that 2-azidoallyl alcohols^11^ might be the ideal substrates for the reductive radical-initiated 1,2-C migration because these compounds contain both an allylic alcohol motif, which is vital for the radical-initiated 1,2-C migration, and an azidyl group, a good leaving group,^12^ which may facilitate the 1,2-C migration after the reductive termination of the radicals.

With the optimal conditions established (ESI, Table S1†), we then explored the scope of this radical-initiated 1,2-migration. As shown in Table 1, a series of naphthenic allylic alcohols could undergo *n*+1 ring expansion with minimal impact on the product yield (Table 1, **3aa–aq**). Notably, only the alkyl groups were migrated when using benzonaphthenic allylic alcohols in the reaction. These results might be attributed to the aryl group possessing greater steric resistance. The structure of **3an** was further verified by single-crystal diffraction. Interestingly, the vinyl azide derived from a pharmaceutical ethisterone was also a viable substrate, affording the migration product **3aq** in 57% yield, which highlighted the applicability of this strategy in the late-stage modification of pharmaceuticals. Moreover, the acyclic allylic alcohol with an alkyl chain also successfully delivered the migration product **3ar** in 64% yield.

**Table 1:** Substrate scope of 2-azidoallyl alcohols^a^^b^

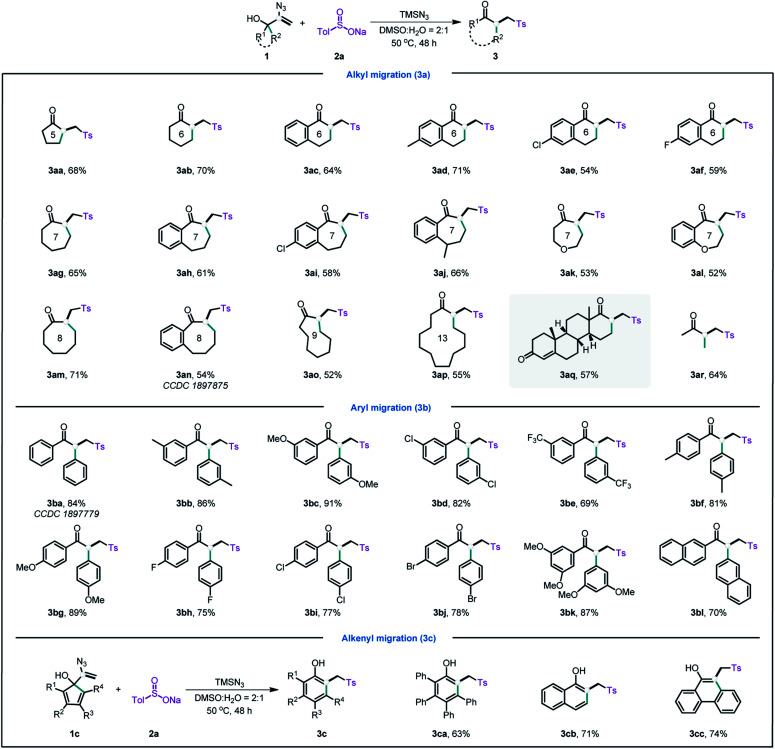

a Standard reaction conditions: **1** (0.5 mmol), TMSN_3_ (2.0 mmol), **2a** (3.0 mmol) in H_2_O (0.7 mL) and DMSO (1.4 mL) at 50 °C in air for 48 h.

b Isolated yields.

Next, we extend the reaction scope to a range of aryl allylic alcohols. In comparison with alkyl allylic alcohols, aryl allylic alcohols gave the migration products in higher yields. The structure of **3ba** was unambiguously confirmed by X-ray single crystal diffraction (CCDC 1897779).† As demonstrated by the arene scope (Table 1, **3ba–bl**), a variety of aryl allylic alcohols, including electron-withdrawing phenyl, electron-donating phenyl, polysubstituted phenyl, and fused rings, afforded the corresponding products in moderate to high yields (67–89%). Unsurprisingly, the substrates containing electron-donating groups afforded higher yields than those containing electron-withdrawing groups.

Phenols and their derivatives are important structural constituents of numerous pharmaceuticals, agrochemicals, polymers, and natural products.^13^ The most common method for synthesising phenols is the hydroxylation of aryl halides.^14^ However, the method usually requires transition metals and harsh reaction conditions. Interestingly, by using the current strategy, inexpensive and abundant cyclopentadiene moieties can also be easily converted into phenols (Table 1, **3ca–cc**) in moderate to good yield. Thus, this strategy provides metal-free and mild conditions for accessing phenols.

Next, we investigated the migration capabilities of different groups (Table 2). When using a substrate that contains two different alkyl groups (**1da**), the product with the less sterically hindered alkyl group is obtained in a higher migration ratio. A comparison of aryl groups and alkyl groups in the same allylic alcohols showed that the migration of aryl groups was more facile, and the migration ratio ranged from 1 : 4 to 1 : 1.3 (**3db–dd**). The results of the migration ratio of different aryl groups (**3de–dh**) revealed that aryl moieties with electron-donating groups possessed higher migration ratios than aryl moieties with electron-withdrawing groups.

**Table 2:** Investigation of the migration efficiency

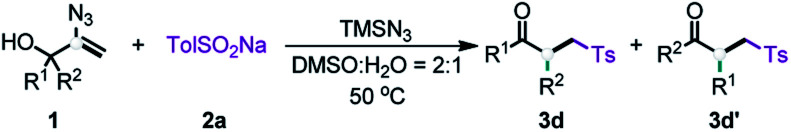					
Entry	**1**	R^1^	R^2^	Yield^a^ (%)	
				**3d**	**3d′**
1	**1da**	Me	*t*-Bu	15	42
2	**1db**	Me	C_6_H_5_	53	26
3	**1dc**	Me	4-MeOC_6_H_5_	56	14
4	**1dd**	Me	4-CF_3_C_6_H_5_	42	32
5	**1de**	C_6_H_5_	4-MeC_6_H_5_	42	40
6	**1df**	C_6_H_5_	4-MeOC_6_H_5_	46	39
7	**1dg**	C_6_H_5_	4-ClC_6_H_5_	41	44
8	**1dh**	C_6_H_5_	4-CF_3_C_6_H_5_	36	48

a Isolated yields.

After the evaluation of the scope of our allylic alcohols, we turned our attention to sulfonyl radical precursors (Table 3). We carried out the reaction of various sodium sulfinates with allylic alcohol **1ba** under standard conditions. Pleasingly, the sodium sulfinates with straight chain alkyl (**3ea**), cyclic alkyl (**3eb**), and aryl (**3ec–ef**) groups were all suitable for this radical-initiated 1,2-carbon migration, and afforded corresponding products in 71–91% yield.

**Table 3:** Substrate scope of sodium sulfinates^a^

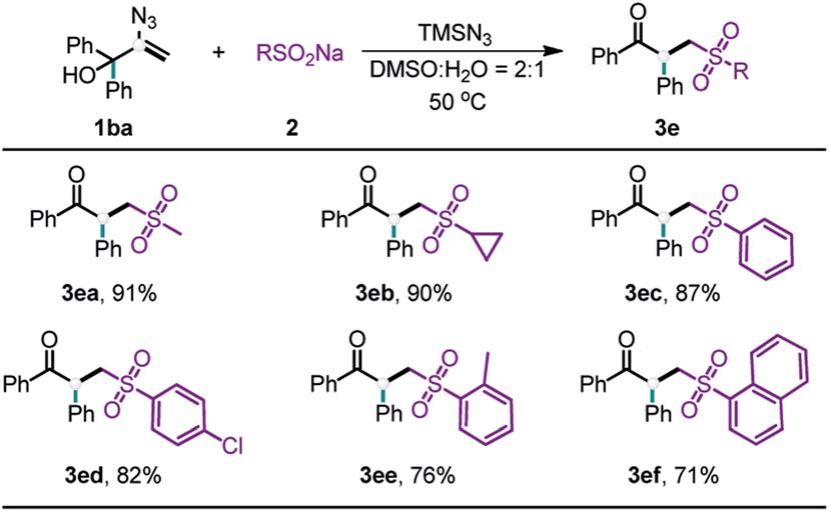

a Isolated yields.

In this work, the 2-azidoallyl alcohols substrates were derived from propargylic alcohols through a silver-catalyzed hydroazidation of alkynes.^15^ Consequently, we hypothesized that the radical-initiated 1,2-carbon migration could be directly achieved from propargylic alcohols in a one pot process. With a slight modification of the reaction conditions, we realized the one-pot preparation of the desired products from propargylic alcohols (Table 4). Propargylic alcohols containing cyclic alkyl (**3ag** and **3ah**), heterocyclic alkyl (**3ak** and **3al**), acyclic alkyl (**3ar**), and aryl (**3ba**) groups all gave the desired migration products, although the yields were slightly lower than those from the reactions of the 2-azidoallyl alcohols. It should be noted that the ring expansion products could be directly generated from a bioactive compound, ethisterone (**3aq**). Performing such a reaction in a single step could greatly reduce the cost of pharmaceutical modification. The fused phenol (**3cd**) could also be obtained in moderate yield *via* the one-step reaction. In addition, the migration order of the different substituted groups (**3db**) was nearly identical to that observed in vinyl azide-based protocol. Furthermore, alkyl sodium sulfinates (**3ea**) were also well tolerated.

**Table 4:** Substrate scope of propargyl alcohols^a^^,^^b^

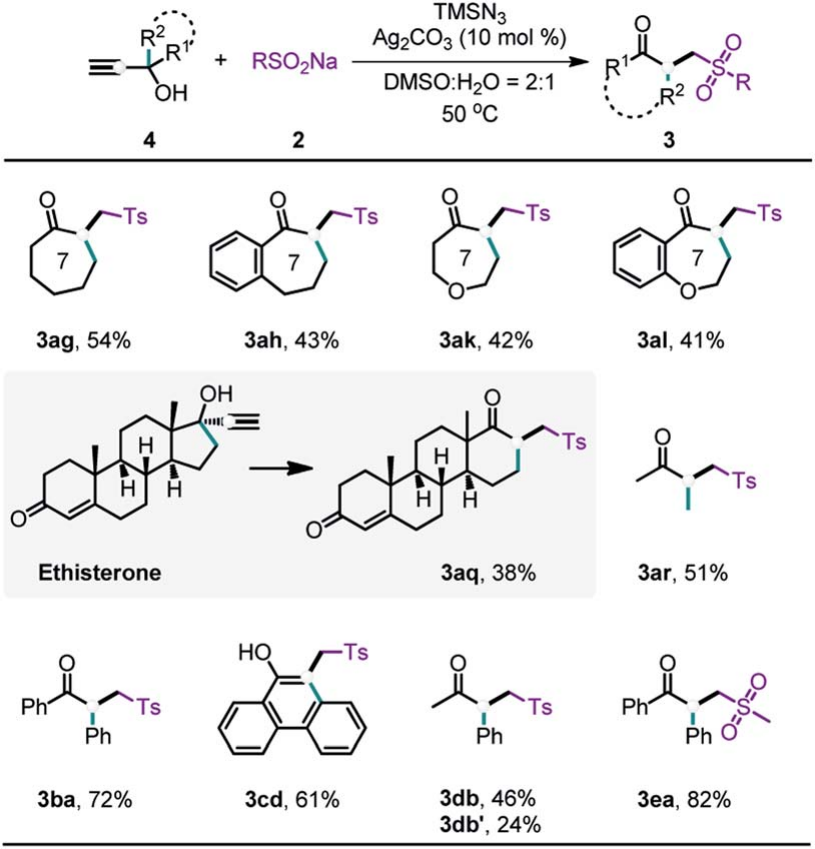

a Standard reaction conditions: **4** (0.5 mmol), TMSN_3_ (2.0 mmol), **2** (3.0 mmol), Ag_2_CO_3_ (0.05 mmol) in H_2_O (0.7 mL) and DMSO (1.4 mL) at 50 °C in air for 48 h.

b Isolated yields.

To gain more insight into the mechanism of radical-initiated 1,2-carbon migration, we conducted various experiments to confirm the presence or absence of radical and carbanion intermediates (Scheme 1). When the reaction of **1ba** was performed in the presence of TEMPO (6.0 equiv.), the reaction was suppressed under the standard conditions (Scheme 1, eqn (1)), supporting the involvement of a radical intermediate. To prove the formation of a carbanion intermediate, we carried out two deuterium labeling experiments (Scheme 1, eqn (2) and (3)). The resulting products [d]-**3ba** and **MA-1** contain the deuterium atom α in the carbonyl group, confirming the formation of a carbanion intermediate. To identify the key intermediate of the 1,2-migration, we prepared a potential intermediate **M1** and subjected it to the standard conditions (Scheme 1, eqn (4)). But, the product **3ba** was not observed and almost all of the **M1** was recovered, which indicates that **M1** is not a key intermediate. However, the product **3ba** was obtained in a yield of 41% while **M2** was subjected to the standard conditions (eqn (5)). If the hydroxyl group in the 2-azidoallyl alcohols was protected (**M3**), the reaction would not give the corresponding migration product (**3ga**), but generate product **5** with a yield of 51% (eqn (6)).^11*c*^ These results proved that the reaction involved a 1,3-H migration process thereby enabling an oxygen anion intermediate **IV** (other mechanistic studies are discussed in ESI Fig. S1†).

**Scheme 1:**
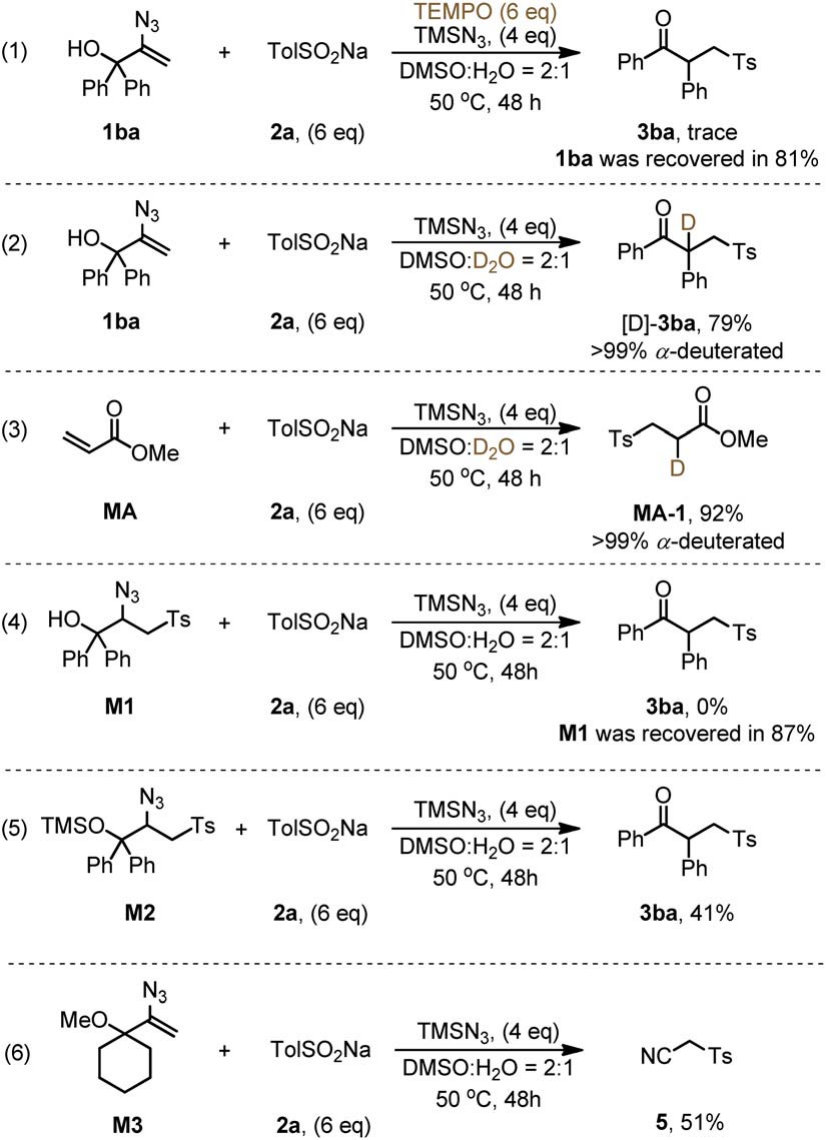
Mechanistic investigations.

Based on the above experimental results and relevant literature, a possible reaction pathway was proposed as shown in Fig. 2. First, TolSO_2_TMS (**I**) is generated by the anion exchange of TolSO_2_Na with TMSN_3_. Such intermediates are known to be somewhat unstable,^16^ as similar to the analogous compounds, such as TolSO_2_I,^17^ and TMSTePh^18^ and thus undergo homolysis. Therefore, we anticipated that TolSO_2_TMS (**I**) should also yield sulfonyl and trimethylsilyl radicals.^19^ Then the 2-azidoallyl alcohol **1ba** is readily attacked by the sulfonyl radical, leading to carbon-centered radical **II**. Subsequently, the carbon-centered radical **II** undergoes single electron transfer by the oxidation of sulfinate to the sulfonyl radical yielding the carbanion **III**.^20^ A 1,3-H shift of carbanion **III** affords the intermediate **IV**^21^ which rapidly undergoes 1,2-migration with the assistance of the azidyl leaving group, generating the desired product. It is worth noting that the present work is a novel radical reaction mode for vinyl azides compared to the existing reports that involve N–N bond breaking in the presence of radicals. Moreover, the development of this strategy is of great significance for the application of vinyl azides in the reconstruction of C–C bonds.

**Fig. 2:**
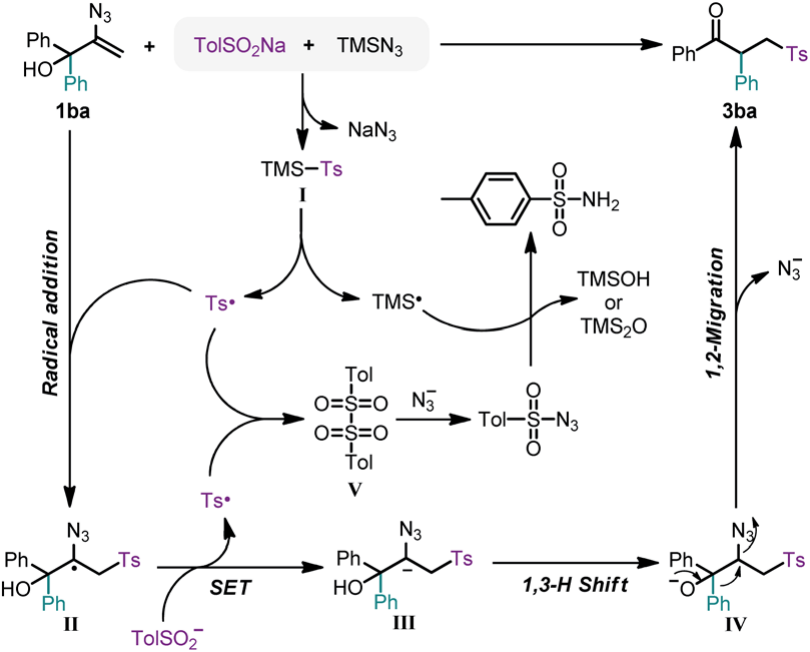
Proposed mechanism.

On the other hand, the coupling of sulfonyl radicals produces intermediate **V**.^22^ The azidyl anion that is generated in the reaction is more prone to attack intermediate **V** to afford tosyl azide.^23^ Subsequently, tosyl azide is reduced to *p*-toluenesulfonamide by the trimethylsilyl radical.^24^ The sideproducts tosyl azide and *p*-toluenesulfonamide were isolated by column chromatography, and the associated TMSOH and TMS_2_O have been detected by GC-MS.^25^

## Conclusions

In conclusion, we report a novel RRPCO reaction: reductive radical-initiated 1,2-C migration under transition-metal free conditions. The key driving force for this procedure was the presence of an azidyl group as a good leaving group. This reaction features broad substrate scope, good functional group tolerance, and the facile generation of diverse ketones and phenols. Moreover, the direct use of propargyl alcohols in a one-pot process was also established, providing high step economy. Mechanistic studies reveal that the combination of sodium sulfinates and TMSN_3_ plays a vital role in the generation of sulfonyl radicals and the reduction of carbon radicals. Further efforts to develop an asymmetrical version of this novel reductive radical-initiated 1,2-C migration are underway in our laboratory.

## Conflicts of interest

There are no conflicts to declare.

## Supplementary Material

SC-011-D0SC02559C-s001

SC-011-D0SC02559C-s002
