# Supplementary material for: Reductive radical-initiated 1,2-C migration assisted by an azidyl group
Source: Chem Sci. 2020 Jul 7;11(30):7921–6. doi: 10.1039/d0sc02559c (PMC8163324; doi:10.1039/d0sc02559c)
Supplement: SC-011-D0SC02559C-s001 [file SC-011-D0SC02559C-s001.pdf]

Electronic Supplementary Material (ESI) for Chemical Science.
This journal is © The Royal Society of Chemistry 2020

Supporting Information For:

Reductive radical-initiated 1,2-C migration assisted by azidyl group

Xueying Zhang,†[a] Zhansong Zhang,†[a] Jin-Na Song,*[b] and Zikun Wang*[a]

[a] Jilin Province Key Laboratory of Organic Functional Molecular Design & Synthesis, Department of Chemistry, Northeast Normal University, Changchun 130024, China. [b] School of Life Science, Jilin University, Changchun 130012, China.

Email: wangzk076@nenu.edu.cn; songjn2010@jlu.edu.cn

Contents

1. General Information 2

2. Optimization of Reaction Conditions 2

3. Typical Procedure for Synthesis of Substrates and Products..........4

4. Investigation of mechanism..18

5. X-ray Structure of **3an** and **3ba**...25

6. NMR Spectra of the Products 27

7. References 81

1. General Information

Reagents and Instruments: All reagents were purchased from commercial sources and used without purification unless otherwise mentioned. The products were purified by column chromatography over silica gel (300-400 size). ^{1}H, ^{13}C Nuclear Magnetic Resonance (NMR) spectra were recorded at 25 °C on a Bruker 600 MHz, 150 MHz, and TMS was used as internal standard. High resolution mass spectra (HRMS) were recorded on Bruck microTof by using ESI method.

2. Optimization of Reaction Conditions

Table S1. Optimization of the reaction conditions[a]

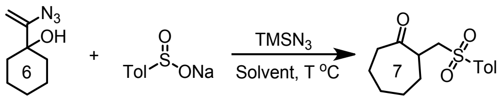

1ag **2a** **3ag**

entry	$TMSN_3$	**2a**	solvent	T (°C)	yield (%)[b]
1	4 eq.	4 eq.	DMSO:H_2O/2:1	50	58
2	4 eq.	4 eq.	DMSO:H_2O/3:1	50	55
3	4 eq.	4 eq.	DMSO:H_2O/1:1	50	49
4	4 eq.	4 eq.	DMF:H_2O/2:1	50	38
5	4 eq.	4 eq.	NMP:H_2O/2:1	50	41
6	4 eq.	4 eq.	DCM:H_2O/2:1	50	0[c]
7	4 eq.	4 eq.	CH_3CN:H_2O/2:1	50	43
8	4 eq.	4 eq.	CH_3CH_2OH:H_2O/2:1	50	52
9	4 eq.	4 eq.	DMSO:H_2O/2:1	70	44
10	4 eq.	4 eq.	DMSO:H_2O/2:1	30	54
11	3 eq.	4 eq.	DMSO:H_2O/2:1	50	56
12	4 eq.	3 eq.	DMSO:H_2O/2:1	50	39
**13**	**4 eq.**	**6 eq.**	**DMSO:H_2O/2:1**	**50**	**65**
14	4 eq.	7 eq.	DMSO:H_2O/2:1	50	64

[a] Standard reaction conditions: **1ag** (0.5 mmol), $TMSN_3$ (2.0 mmol), **2a** (3.0 mmol), in H_2O (0.7 mL) and DMSO (1.4 mL) at 50 °C under air for 48 h. [b] Yield of isolated product. [c] No **3ag** was detected by ^{1}H NMR analysis of crude product. DCM = dichloromethane, DMSO = dimethyl sulfoxide, NMP = *N*-methyl pyrrolidone

To probe the feasibility of our proposed reaction, we investigated the reaction of 2-azidoallyl alcohols **1ag** with sodium *p*-toluenesulfinate **2a** in the presence of $TMSN_3$ in a DMSO/H_2O cosolvent system at 50 °C (Table S1, entry 1). Gratifyingly, after 48 h, the 1,2-migration product was isolated in 58% yield. We then optimized the other reaction parameters, including solvent, temperature and the amount of $TMSN_3$ and **2a**. Except for dichloromethane (DCM), the cosolvents of H_2O with dimethylsulfoxide (DMSO), dimethylformamide (DMF), *N*-methyl-2-

pyrrolidone (NMP), acetonitrile, or ethyl alcohol all afforded the desired product in moderate yields (entries 1-8). The yield decreased if the temperature was raised to 70 ^{o}C or reduced to 30 ^{o}C (entries 9 and 10). The reason might be that the quantity of byproducts was increased when the temperature was increased or lengthened the reaction timeextended (**1ag** was not observed after 72 h at 30 ^{o}C). The amounts of **2a** and $TMSN_3$ also were also important, and the results listed in entries 11-14 demonstrated that the yield of **3ag** was the highest (65%) when four equivalents of $TMSN_3$ and six equivalents of **2a** were used. After systematic screening of the reaction parameters, the conditions listed in entry 13 were found to be optimum for this reductive 1,2-carbon migration reaction.

3. Typical Procedure for Synthesis of Substrates and Products

3.1 Typical procedure for the preparation of 2-azidoallyl alcohols

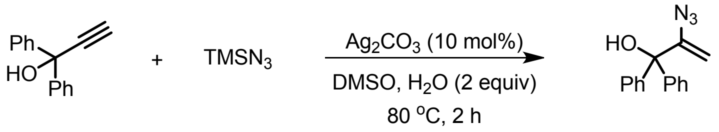

A-1 **1ba**, 83%

To a solution of ethynyl alcohol **A-1** (5.0 mmol, 1.041 g), $TMSN_3$ (7.5 mmol, 0.863 g), and H_2O (10.0 mmol, 0.180 g) in DMSO (10 mL), Ag_2CO_3 (0.5 mmol, 0.138 g) was added. The mixture was stirred at 80 °C for 2 h. Upon completion of the reaction, saturated NH_4Cl (aq.) was added to quench the reaction and the mixture was extracted with CH_2Cl_2 (3 × 50 mL). The combined organic layers were washed with brine, dried over $MgSO_4$, filtered, and concentrated *in vacuo*. The resulting mixture was purified by silica gel column chromatography using (petroleum ether:ethyl acetate = 20:1) to afford 2-azidoallyl alcohol **1ba** (1.043 g, 83%).[1],[2]

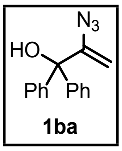

2-Azido-1,1-diphenylprop-2-en-1-ol (1ba): Yellow solid; m.p. 26-28 °C; ^1H NMR (600 MHz, $CDCl_3$) δ 7.39-7.37 (m, 4H), 7.34-7.28 (m, 6H), 5.01 (d, J = 2.4 Hz, 1H), 4.71 (d, J = 1.8 Hz, 1H), 3.05 (s, 1H). ^{13}C NMR (150 MHz, $CDCl_3$) δ 150.96, 143.17, 128.09, 127.90, 127.60, 102.11, 80.67. **HRMS** (ESI) m/z calculated for $C_{15}H_{13}N_3NaO$ $[M+Na]^+$ 274.0960, found 274.0956.

3.2 Typical procedure for the radical-initiated reductive 1,2-carbon migration

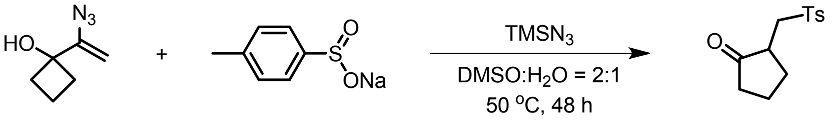

1aa **2a** **3aa**, 68%

To a solution of 2-azidoallyl alcohol **1aa** (0.5 mmol, 0.070 g) and sodium *p*-tolylsulfinate (3.0 mmol, 0.534 g) in the co-solvent (DMSO 1.4 mL, H_2O 0.7 mL), $TMSN_3$ (2.0 mmol, 0.230 g) was added. The mixture was stirred at 50 °C for 48 h. Upon completion of the reaction, saturated NH_4Cl (aq.) was added to quench the reaction and the mixture was extracted with CH_2Cl_2 (3 × 10 mL). The combined organic layers were washed with brine, dried over $MgSO_4$, filtered, and concentrated *in vacuo*. The resulting mixture was purified by silica gel column chromatography using (petroleum ether:ethyl acetate = 6:1) to afford product **3aa** (0.086 g, 68%).

3.3 Typical procedure for the 1,2-carbon migration from propargyl alcohols

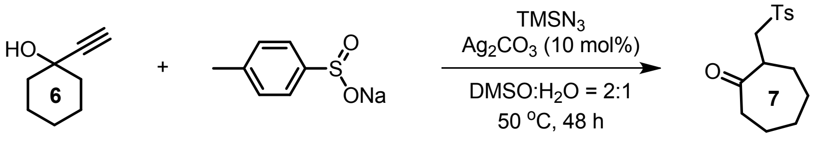

P-1 **2a** **3ag**, 54%

To a solution of propargyl alcohol **P-1** (0.5 mmol, 0.062 g), Ag_2CO_3 (0.05 mmol, 0.014 g) and sodium *p*-tolylsulfinate (3.0 mmol, 0.534 g) in the co-solvent (DMSO 1.4 mL, H_2O 0.7 mL), $TMSN_3$ (2.0 mmol, 0.230 g) was added. The mixture was stirred at 50 ^{o}C for 48 h. Upon completion of the reaction, saturated NH_4Cl (aq.) was added to quench the reaction and the mixture was extracted with CH_2Cl_2 (3 × 10 mL). The combined organic layers were washed with brine, dried over $MgSO_4$, filtered, and concentrated *in vacuo*. The resulting mixture was purified by silica gel column chromatography using (petroleum ether:ethyl acetate = 6:1) to afford product **3ag** (0.076 g, 54%).

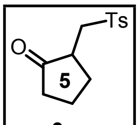

3aa

2-(Tosylmethyl)cyclopentanone (3aa): Yellow oil; ^{1}H NMR (600 MHz, $CDCl_3$) δ 7.80 (d, *J* = 8.2 Hz, 2H), 7.37 (d, *J* = 8.2 Hz, 2H), 3.64 (dd, *J* = 14.2, 1.8 Hz, 1H), 2.91 (dd, *J* = 14.2, 10.2 Hz, 1H), 2.60-2.55 (m, 2H), 2.46 (s, 3H), 2.40-2.33 (m, 1H), 2.15-2.04 (m, 2H), 1.88-1.76 (m, 1H), 1.72-1.58 (m, 1H). ^{13}C NMR (150 MHz, $CDCl_3$) δ 216.63, 144.94, 136.49, 130.03, 127.91, 56.68, 44.15, 36.72, 29.96, 21.66, 20.58. **HRMS** (ESI) m/z calculated for $C_{13}H_{16}NaO_3S$ $[M+Na]^+$ 275.0712, found 275.0708.

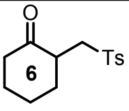

3ab

2-(Tosylmethyl)cyclohexanone (3ab): Yellow oil; ^{1}H NMR (600 MHz, $CDCl_3$) δ 7.78 (d, *J* = 8.2 Hz, 2H), 7.35 (d, *J* = 8.2 Hz, 2H), 3.87 (dd, *J* = 14.4, 3.6 Hz, 1H), 3.07-3.02 (m, 1H), 2.82 (dd, *J* = 14.4, 7.7 Hz, 1H), 2.59-2.55 (m, 1H), 2.45 (s, 3H), 2.43-2.40 (m, 1H), 2.38-2.32 (m, 1H), 2.14-2.01 (m, 1H), 1.91-1.88 (m, 1H), 1.82-1.70 (m, 1H), 1.67-1.58 (m, 1H), 1.47 (qd, *J* = 12.9, 3.6 Hz, 1H). ^{13}C NMR (150 MHz, $CDCl_3$) δ 208.43, 144.74, 136.99, 129.93, 127.80, 55.42, 45.19, 41.85, 34.79, 27.73, 25.08, 21.65. **HRMS** (ESI) m/z calculated for $C_{14}H_{18}NaO_3S$ $[M+Na]^+$ 289.0869, found 289.0863.

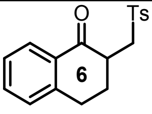

3ac

2-(Tosylmethyl)-3,4-dihydronaphthalen-1(2H)-one (3ac): Yellow solid; m.p: 97-98 ^{o}C;^{1}H NMR (600 MHz, $CDCl_3$) δ 7.89 (d, *J* = 7.8 Hz, 1H), 7.77 (d, *J* = 8.2 Hz, 2H), 7.44-7.38 (m, 1H), 7.30 (d, *J* = 8.2 Hz, 2H), 7.22 (t, *J* = 7.6 Hz, 1H), 7.20-7.16 (m, 1H), 4.08 (dd, *J* = 14.4, 2.2 Hz, 1H), 3.08-3.02 (m, 2H), 2.98-2.89 (m, 2H), 2.76-2.69 (m, 1H), 2.38 (s, 3H), 1.95 (qd, *J* = 13.2, 4.2 Hz, 1H). ^{13}C NMR (150 MHz, $CDCl_3$) δ 195.79, 144.85, 143.91, 136.81, 133.90, 131.61, 130.03, 128.90, 127.91, 127.75, 126.80, 55.74, 43.11, 29.37, 28.98, 21.67. **HRMS** (ESI) m/z calculated for $C_{18}H_{18}NaO_3S$ $[M+Na]^+$ 337.0869, found 337.0873.

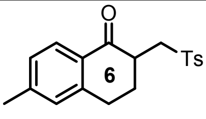

3ad

6-Methyl-2-(tosylmethyl)-3,4-dihydronaphthalen-1(2H)-one (3ad): Yellow solid; m.p: 49-50 ^{o}C; **^{1}H NMR** (600 MHz, $CDCl_3$) δ 7.86 (d, *J* = 7.8 Hz, 1H), 7.84 (d, *J* = 8.0 Hz, 2H), 7.36 (d, *J* = 8.0 Hz, 2H), 7.09 (d, *J* = 7.8 Hz, 1H), 7.05 (s, 1H), 4.15 (dd, *J* = 14.4, 2.0 Hz, 1H), 3.12-3.04 (m, 2H), 3.02-2.98 (m, 1H), 2.94 (dt, *J* = 16.8, 3.6 Hz, 1H), 2.80-2.74 (m, 1H), 2.45 (s, 3H), 2.37 (s, 3H), 2.04-1.95 (m, 1H). **^{13}C NMR** (150 MHz, $CDCl_3$) δ 195.44, 144.87, 144.80, 143.98, 136.84, 130.01, 129.30, 129.28, 127.90, 127.85, 127.82, 55.79, 43.03, 29.42, 28.94, 21.75, 21.66. **HRMS** (ESI) m/z calculated for $C_{19}H_{20}NaO_3S$ $[M+Na]^+$ 351.1034, found 351.1025.

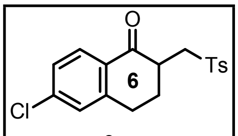

3ae

6-Chloro-2-(tosylmethyl)-3,4-dihydronaphthalen-1(2H)-one (3ae): Yellow solid; m.p: 71-72 °C; **^{1}H NMR** (600 MHz, $CDCl_3$) δ 7.91 (d, *J* = 8.0 Hz, 1H), 7.84 (d, *J* = 8.0 Hz, 2H), 7.38 (d, *J* = 8.0 Hz, 2H), 7.29-7.25 (m, 2H), 4.12 (dd, *J* = 14.4, 2.0 Hz, 1H), 3.16-3.10 (m, 2H), 3.07-2.94 (m, 2H), 2.84-2.76 (m, 1H), 2.46 (s, 3H), 2.02 (qd, *J* = 13.0, 4.2 Hz, 1H). **^{13}C NMR** (150 MHz, $CDCl_3$) δ 194.78, 145.44, 144.94, 140.30, 136.76, 130.06, 130.04, 129.44, 128.77, 127.90, 127.39, 55.61, 42.97, 29.10, 28.83, 21.68. **HRMS** (ESI) m/z calculated for $C_{18}H_{17}ClNaO_3S$ $[M+Na]^+$ 371.0479, found 371.0483.

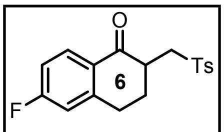

3af

6-Fluoro-2-(tosylmethyl)-3,4-dihydronaphthalen-1(2H)-one (3af): Yellow oil; ^{1}H NMR (600 MHz, $CDCl_3$) δ 8.00 (dd, *J* = 8.6, 6.0 Hz, 1H), 7.84 (d, *J* = 8.0 Hz, 2H), 7.38 (d, *J* = 8.0 Hz, 2H), 6.98 (td, *J* = 8.6, 2.0 Hz, 1H), 6.93 (d, *J* = 9.0 Hz, 1H), 4.13 (dd, *J* = 14.4, 2.0 Hz, 1H), 3.16-3.10 (m, 2H), 3.04-2.97 (m, 2H), 2.82-2.78 (m, 1H), 2.46 (s, 3H), 2.02 (qd, *J* = 13.2, 4.2 Hz, 1H). ^{13}C NMR (150 MHz, $CDCl_3$) δ 194.32, 165.96 (d, *J* = 253.5 Hz), 146.99 (d, *J* = 9.2 Hz), 144.89, 136.81, 130.95 (d, *J* = 9.9 Hz), 130.04, 128.27 (d, *J* = 2.6 Hz), 127.89, 115.20 (d, *J* = 21.5 Hz), 114.60 (d, *J* = 22.1 Hz), 55.64, 42.87, 29.16, 29.12, 21.66. **HRMS** (ESI) m/z calculated for $C_{18}H_{17}FNaO_3S$ $[M+Na]^+$ 355.0775, found 355.0778.

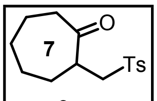

3ag

2-(Tosylmethyl)cycloheptanone (3ag): White solid; m.p: 58-59 °C; ^{1}H NMR (600 MHz, $CDCl_3$) δ 7.77 (d, *J* = 8.0 Hz, 2H), 7.35 (d, *J* = 8.0 Hz, 2H), 3.83 (dd, *J* = 14.2, 6.0 Hz, 1H), 3.35-3.28 (m, 1H), 2.96 (dd, *J* = 14.2, 6.0 Hz, 1H), 2.59 (dt, *J* = 16.0, 4.8 Hz, 1H), 2.45 (s, 3H), 2.43-2.38 (m, 1H), 2.05-1.98 (m, 1H), 1.91-1.87 (m, 1H), 1.85-1.69 (m, 3H), 1.65-1.56 (m, 1H), 1.50-1.44 (m, 1H), 1.32-1.23 (m, 1H). ^{13}C NMR (150 MHz, $CDCl_3$) δ 211.43, 144.77, 136.93, 129.93, 127.90, 57.60, 45.38, 42.99, 31.69, 28.84, 28.43, 23.24, 21.65. **HRMS** (ESI) m/z calculated for $C_{15}H_{20}NaO_3S$ $[M+Na]^+$ 303.1025, found 303.1042.

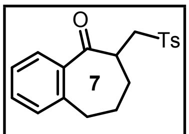

3ah

6-(Tosylmethyl)-6,7,8,9-tetrahydro-5H-benzo[7]annulen-5-one (3ah): White solid; m.p: 84-85 °C; ^{1}H NMR (600 MHz, $CDCl_3$) δ 7.74 (d, *J* = 8.2 Hz, 2H), 7.44 (dd, *J* = 7.6, 1.0 Hz, 1H), 7.39 (td, *J* = 7.6, 1.2 Hz, 1H), 7.31 (d, *J* = 8.0 Hz, 2H), 7.26-7.21 (m, 2H), 4.06 (dd, *J* = 14.4, 6.6 Hz, 1H), 3.59-3.54 (m, 1H), 3.13 (dd, *J* = 14.2, 5.0 Hz, 1H), 3.09-3.06 (m, 1H), 3.01-2.91 (m, 1H), 2.45 (s, 3H), 2.26-2.12 (m, 2H), 1.73-1.57 (m, 2H).^{13}C NMR (150 MHz, $CDCl_3$) δ 202.33, 144.73, 142.42, 138.43, 136.62, 131.86, 130.11, 129.95, 128.91, 128.09, 126.32, 57.00, 44.03, 33.32, 30.57, 25.35, 21.66. **HRMS** (ESI) m/z calculated for $C_{19}H_{20}NaO_3S$ $[M+Na]^+$ 351.1025, found 351.1033.

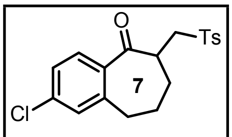

3ai

2-Chloro-6-(tosylmethyl)-6,7,8,9-tetrahydro-5*H*-benzo[7]annulen-5-one (3ai): White solid; m.p: 55-56 °C; ^{1}H NMR (600 MHz, $CDCl_3$) δ 7.74 (d, *J* = 8.2 Hz, 2H), 7.42 (d, *J* = 8.2 Hz, 1H), 7.32 (d, *J* = 8.2 Hz, 2H), 7.24-7.22 (m, 2H), 4.04 (dd, *J* = 14.2, 6.6 Hz, 1H), 3.56-3.51 (m, 1H), 3.12 (dd, *J* = 14.4, 5.0 Hz, 1H), 3.10-3.04 (m, 1H), 2.98-2.89 (m, 1H), 2.45 (s, 3H), 2.26-2.13 (m, 2H), 1.72-1.57 (m, 2H). ^{13}C NMR (150 MHz, $CDCl_3$) δ 201.10, 144.81, 144.21, 137.99, 136.77, 136.60, 130.57, 130.04, 129.97, 128.06, 126.66, 56.90, 43.96, 33.08, 30.24, 25.12, 21.66. **HRMS** (ESI) m/z calculated for $C_{19}H_{19}ClNaO_3S$ $[M+Na]^+$ 385.0636, found 385.0645.

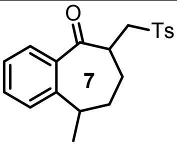

3aj

9-Methyl-6-(tosylmethyl)-6,7,8,9-tetrahydro-5*H*-benzo[7]annulen-5-one (3aj): Yellow oil; ^{1}H NMR (600 MHz, $CDCl_3$) δ 7.79 (d, *J* = 8.2 Hz, 2H), 7.44 (td, *J* = 7.8, 1.2 Hz, 1H), 7.38-7.33 (m, 3H), 7.29 (d, *J* = 7.8 Hz, 1H), 7.26 (t, *J* = 7.8 Hz, 1H), 4.02 (dd, *J* = 14.4, 4.8 Hz, 1H), 3.33-3.29 (m, 1H), 3.16-3.07 (m, 2H), 2.46 (s, 3H), 2.29-2.25 (m, 1H), 2.14-2.09 (m, 1H), 1.79-1.69 (m, 1H), 1.69-1.62 (m, 1H), 1.34 (d, *J* = 7.0 Hz, 3H). ^{13}C NMR (150 MHz, $CDCl_3$) δ 205.29, 144.75, 143.73, 139.11, 136.91, 131.98, 129.97, 128.67, 128.01, 126.51, 126.30, 57.51, 45.39, 35.69, 34.38, 27.77, 21.66, 20.32. **HRMS** (ESI) m/z calculated for $C_{20}H_{22}NaO_3S$ $[M+Na]^+$ 365.1182, found 365.1189.

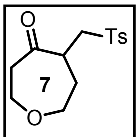

3ak

5-(Tosylmethyl)oxepan-4-one (3ak): White solid; m.p: 84-85 °C; ^{1}H NMR (600 MHz, $CDCl_3$) δ 7.78 (d, *J* = 8.2 Hz, 2H), 7.37 (d, *J* = 8.2 Hz, 2H), 4.11-4.02 (m, 2H), 3.86-3.75 (m, 2H), 3.75-3.67 (m, 1H), 3.56-3.48 (m, 1H), 2.99 (dd, *J* = 14.4, 6.6 Hz, 1H), 2.87-2.76 (m, 1H), 2.64 (dt, *J* = 16.8, 3.6 Hz, 1H), 2.46 (s, 3H), 2.15-2.08 (m, 1H), 1.78-1.71 (m, 1H). ^{13}C NMR (150 MHz, $CDCl_3$) δ 208.11, 145.01, 136.82, 130.03, 127.86, 65.92, 56.71, 45.20, 45.12, 39.80, 33.30, 21.67. **HRMS** (ESI) m/z calculated for $C_{14}H_{18}NaO_4S$ $[M+Na]^+$ 305.0818, found 305.0837.

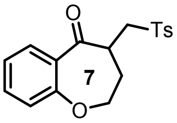

3al

4-(Tosylmethyl)-3,4-dihydrobenzo[b]oxepin-5(2*H*)-one (3al): Yellow solid; m.p: 70-71 °C; ^{1}H NMR (600 MHz, $CDCl_3$) δ 7.73 (d, *J* = 8.2 Hz, 2H), 7.48 (dd, *J* = 7.8, 1.8 Hz, 1H), 7.42 (td, *J* = 7.8, 1.8 Hz, 1H), 7.30 (d, *J* = 8.2 Hz, 2H), 7.07-7.03 (m, 2H), 4.57-4.53 (m, 1H), 3.98 (dd, *J* = 14.4, 6.0 Hz, 1H), 3.83 (td, *J* = 12.6, 5.2 Hz, 1H), 3.76-3.71 (m, 1H), 3.18 (dd, *J* = 14.4, 6.0 Hz, 1H), 2.85-2.77 (m, 1H), 2.44 (s, 3H), 1.89-1.77 (m, 1H). ^{13}C NMR (150 MHz, $CDCl_3$) δ 198.29, 162.37, 144.84, 136.39, 133.93, 129.98, 129.61, 128.09, 127.72, 122.44, 120.30, 72.01, 56.07, 43.81, 34.56, 21.65. **HRMS** (ESI) m/z calculated for $C_{18}H_{18}NaO_4S$ $[M+Na]^+$ 353.0818, found 353.0833.

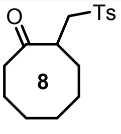

3am

2-(Tosylmethyl)cyclooctanone (3am): Yellow oil; ^{1}H NMR (600 MHz, $CDCl_3$) δ 7.76 (d, *J* = 8.2 Hz, 2H), 7.35 (d, *J* = 8.2 Hz, 2H), 3.82 (dd, *J* = 14.0, 8.4 Hz, 1H), 3.48-3.43 (m, 1H), 2.98 (dd, *J* = 14.0, 3.6 Hz, 1H), 2.69-2.66 (m, 1H), 2.45 (s, 3H), 2.34-2.26 (m, 1H), 2.16-2.07 (m, 1H), 1.95-1.90 (m, 1H), 1.84-1.75 (m, 1H), 1.70-

1.61 (m, 3H), 1.57-1.53 (m, 2H), 1.50-1.41 (m, 1H), 1.06-0.99 (m, 1H). ^{13}C NMR (150 MHz, $CDCl_3$) δ 215.79, 144.82, 136.73, 129.94, 127.87, 58.29, 43.09, 42.53, 34.15, 28.18, 24.44, 24.42, 23.55, 21.66. **HRMS** (ESI) m/z calculated for $C_{16}H_{22}NaO_3S$ $[M+Na]^+$ 317.1182, found 317.1186.

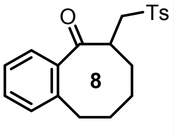

3an

6-(Tosylmethyl)-7,8,9,10-tetrahydrobenzo[8]annulen-5(6H)-one (3an): White solid; m.p: 82-83 °C; ^{1}H NMR (600 MHz, $CDCl_3$) δ 7.72 (d, *J* = 8.2 Hz, 2H), 7.56 (d, *J* = 7.8 Hz, 1H), 7.41 (td, *J* = 7.8, 1.2 Hz, 1H), 7.28-7.23 (m, 3H), 7.19 (d, *J* = 7.8 Hz, 3H), 4.07 (dd, *J* = 14.0, 7.4 Hz, 1H), 4.04-3.98 (m, 1H), 3.45-3.32 (m, 1H), 3.09 (dd, *J* = 14.0, 4.0 Hz, 1H), 2.87 (dt, *J* = 15.0, 5.2 Hz, 1H), 2.41 (s, 3H), 2.05-1.98 (m, 1H), 1.87-1.79 (m, 2H), 1.74-1.65 (m, 2H), 1.56-1.48 (m, 1H), 1.41-1.31 (m, 1H).^{13}C NMR (150 MHz, $CDCl_3$) δ 204.08, 144.72, 139.40, 139.11, 136.44, 132.32, 131.62, 129.89, 128.21, 128.11, 126.41, 57.99, 44.87, 34.50, 32.24, 27.71, 22.34, 21.62. **HRMS** (ESI) m/z calculated for $C_{20}H_{22}NaO_3S$ $[M+Na]^+$ 365.1182, found 365.1188.

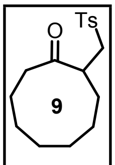

3ao

2-(Tosylmethyl)cyclononanone (3ao): Yellow solid; m.p: 61-64 °C; ^{1}H NMR (600 MHz, $CDCl_3$) δ 7.76 (d, *J* = 8.2 Hz, 2H), 7.35 (d, *J* = 8.2 Hz, 2H), 3.76 (dd, *J* = 14.0, 8.0 Hz, 1H), 3.54-3.32 (m, 1H), 3.00 (dd, *J* = 14.2, 4.4 Hz, 1H), 2.80-2.76 (m, 1H), 2.45 (s, 3H), 2.38-2.34 (m, 1H), 1.99-1.94 (m, 1H), 1.93-1.77 (m, 2H), 1.77-1.68 (m, 1H), 1.67-1.59 (m, 4H), 1.48-1.38 (m, 2H), 1.31-1.23 (m, 2H). ^{13}C NMR (150 MHz, $CDCl_3$) δ 215.63, 144.81, 136.75, 129.94, 127.88, 58.26, 46.31, 43.12, 30.87, 26.68, 26.10, 24.26, 24.07, 23.57, 21.66. **HRMS** (ESI) m/z calculated for $C_{17}H_{24}NaO_3S$ $[M+Na]^+$ 331.1331, found 331.1344.

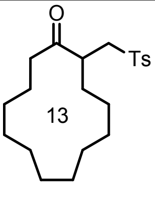

3ap

2-(Tosylmethyl)cyclotridecanone (3ap): Yellow solid; m.p: 67-68 °C; ^{1}H NMR (600 MHz, $CDCl_3$) δ 7.75 (d, *J* = 8.2 Hz, 2H), 7.35 (d, *J* = 8.2 Hz, 2H), 3.73 (dd, *J* = 14.0, 9.2 Hz, 1H), 3.22-3.18 (m, 1H), 2.98 (dd, *J* = 14.0, 3.2 Hz, 1H), 2.77-2.72 (m, 1H), 2.50-2.47 (m, 1H), 2.45 (s, 3H), 1.99-1.93 (m, 1H), 1.60-1.56 (m, 2H), 1.43-1.14 (m, 17H). ^{13}C NMR (150 MHz, $CDCl_3$) δ 210.88, 144.81, 136.65, 129.93, 127.92, 57.59, 44.94, 41.72, 31.78, 26.68, 26.27, 25.97, 24.67, 24.55, 23.81, 23.75, 23.28, 21.70, 21.64. **HRMS** (ESI) m/z calculated for $C_{21}H_{32}NaO_3S$ $[M+Na]^+$ 387.1964, found 387.1971.

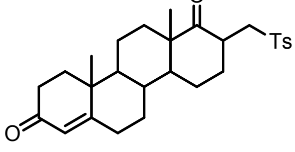

3aq

10a,12a-Dimethyl-2-(tosylmethyl)-2,3,4,4a,5,6,10,10a,10b,11,12,12a-dodecahydrochrysene-1,8(4b*H*,9*H*)-dione (3aq): White solid; m.p: 142-143 °C; ^{1}H NMR (600 MHz, $CDCl_3$) δ 7.78 (d, *J* = 8.0 Hz, 2H), 7.35 (d, *J* = 8.0 Hz, 2H), 5.72 (s, 1H), 3.84 (dd, *J* = 14.4, 4.0 Hz, 1H), 3.52-3.38 (m, 1H), 2.78 (dd, *J* = 14.4, 7.0 Hz, 1H), 2.65-2.54 (m, 1H), 2.45 (s, 3H), 2.41-2.23 (m, 4H), 2.12-1.99 (m, 2H), 1.96-1.82 (m, 1H), 1.73-1.63 (m, 4H), 1.61-1.47 (m, 2H), 1.41-1.32 (m, 2H), 1.18 (s, 3H), 1.15 (s, 3H), 1.01-0.92 (m, 2H), 0.89-0.84 (m, 1H). ^{13}C NMR (150

MHz, $CDCl_3$) δ 212.13, 199.27, 170.15, 144.74, 137.07, 129.90, 127.82, 123.71, 55.90, 52.77, 51.80, 48.33, 39.89, 38.61, 35.41, 35.23, 34.00, 33.88, 32.56, 32.34, 31.17, 23.31, 21.66, 19.75, 17.51, 16.56. **HRMS** (ESI) m/z calculated for $C_{28}H_{36}NaO_4S$ $[M+Na]^+$ 491.2227, found 491.2227.

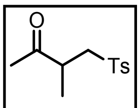
3ar

3-Methyl-4-tosylbutan-2-one (3ar): Yellow oil; ^1H NMR (600 MHz, $CDCl_3$) δ 7.77 (d, *J* = 8.0 Hz, 2H), 7.36 (d, *J* = 8.0 Hz, 2H), 3.73 (dd, *J* = 14.2, 7.0 Hz, 1H), 3.24-3.20 (m, 1H), 2.96 (dd, *J* = 14.2, 5.2 Hz, 1H), 2.45 (s, 3H), 2.21 (s, 3H), 1.27 (d, *J* = 7.2 Hz, 3H). ^{13}C NMR (150 MHz, $CDCl_3$) δ 208.20, 144.90, 136.68, 129.98, 127.92, 58.06, 40.97, 28.30, 21.65, 17.30. **HRMS** (ESI) m/z calculated for $C_{12}H_{16}NaO_3S$ $[M+Na]^+$ 263.0712, found 263.0719.

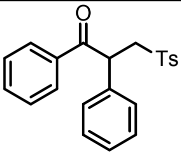
3ba

1,2-Diphenyl-3-tosylpropan-1-one (3ba): White solid; m.p: 90-91 °C; ^1H NMR (600 MHz, $CDCl_3$) δ 7.89 (d, *J* = 7.8 Hz, 2H), 7.70 (d, *J* = 7.8 Hz, 2H), 7.50 (t, *J* = 7.2 Hz, 1H), 7.38 (t, *J* = 7.8 Hz, 2H), 7.25-7.21 (m, 6H), 7.20-7.18 (m, 1H), 5.28 (dd, *J* = 8.8, 3.6 Hz, 1H), 4.42 (dd, *J* = 14.2, 8.8 Hz, 1H), 3.42 (dd, *J* = 14.2, 3.6 Hz, 1H), 2.39 (s, 3H). ^{13}C NMR (150 MHz, $CDCl_3$) δ 195.88, 144.70, 136.54, 136.44, 135.53, 133.41, 129.81, 129.38, 128.88, 128.58, 128.15, 128.11, 127.96, 59.34, 47.56, 21.61. **HRMS** (ESI) m/z calculated for $C_{22}H_{20}NaO_3S$ $[M+Na]^+$ 387.1025, found 387.1026.

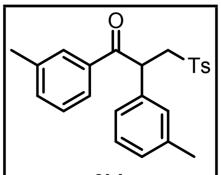
3bb

1,2-Di-*m*-tolyl-3-tosylpropan-1-one (3bb): White solid; m.p: 99-100 °C; ^1H NMR (600 MHz, $CDCl_3$) δ 7.66-7.57 (m, 4H), 7.24 (d, *J* = 7.6 Hz, 1H), 7.21-7.18 (m, 1H), 7.15 (d, *J* = 7.8 Hz, 2H), 7.05 (t, *J* = 7.6 Hz, 1H), 6.96 (d, *J* = 7.8 Hz, 1H), 6.92 (d, *J* = 11.2 Hz, 2H), 5.16 (dd, *J* = 8.8, 3.6 Hz, 1H), 4.32 (dd, *J* = 14.2, 8.8 Hz, 1H), 3.33 (dd, *J* = 14.2, 3.6 Hz, 1H), 2.32 (s, 3H), 2.28 (s, 3H), 2.16 (s, 3H). ^{13}C NMR (150 MHz, $CDCl_3$) δ 196.12, 144.61, 139.11, 138.39, 136.48, 136.43, 135.58, 134.21, 129.75, 129.41, 129.21, 128.69, 128.63, 128.43, 128.10, 126.14, 125.30, 59.39, 47.46, 21.61, 21.37, 21.34. **HRMS** (ESI) m/z calculated for $C_{24}H_{24}NaO_3S$ $[M+Na]^+$ 415.1338, found 415.1337.

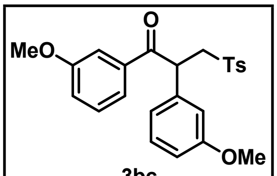
3bc

1,2-Bis(3-methoxyphenyl)-3-tosylpropan-1-one (3bc): Yellow oil; ^1H NMR (600 MHz, $CDCl_3$) δ 7.70 (d, *J* = 8.0 Hz, 2H), 7.51 (d, *J* = 7.8 Hz, 1H), 7.39 (t, *J* = 1.2 Hz, 1H), 7.29 (t, *J* = 8.0 Hz, 1H), 7.24 (d, *J* = 8.0 Hz, 2H), 7.18-7.15 (m, 1H), 7.05 (dd, *J* = 8.0, 2.4 Hz, 1H), 6.82 (d, *J* = 7.8 Hz, 1H), 6.73-6.72 (m, 2H), 5.20 (dd, *J* = 9.0, 3.5 Hz, 1H), 4.40 (dd, *J* = 14.2, 9.0 Hz, 1H), 3.79 (s, 3H), 3.72 (s, 3H), 3.41 (dd, *J* = 14.2, 3.6 Hz, 1H), 2.39 (s, 3H). ^{13}C NMR (150 MHz, $CDCl_3$) δ 195.51, 160.17, 159.75, 144.70, 137.94, 136.84, 136.41, 130.41, 129.80, 129.56, 128.12, 121.49, 120.44, 120.02, 113.64, 113.37, 113.12, 59.24, 55.40, 55.24, 47.72, 21.61. **HRMS** (ESI) m/z calculated for $C_{24}H_{24}NaO_5S$ $[M+Na]^+$ 447.1237, found 447.1236.

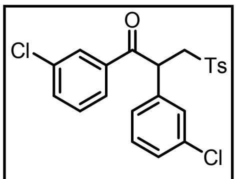

3bd

1,2-Bis(3-chlorophenyl)-3-tosylpropan-1-one (3bd): Yellow solid; m.p: 74-75 °C; ^{1}H NMR (600 MHz, $CDCl_3$) δ 7.83 (t, *J* = 1.8 Hz, 1H), 7.80-7.75 (m, 1H), 7.68 (d, *J* = 8.4 Hz, 2H), 7.51-7.49 (m, 1H), 7.36 (t, *J* = 7.8 Hz, 1H), 7.28-7.23 (m, 2H), 7.21-7.16 (m, 3H), 7.14 (dt, *J* = 6.6, 1.8 Hz, 1H), 5.19 (dd, *J* = 8.6, 4.0 Hz, 1H), 4.33 (dd, *J* = 14.2, 8.6 Hz, 1H), 3.43 (dd, *J* = 14.2, 4.0 Hz, 1H), 2.41 (s, 3H).^{13}C NMR (150 MHz, $CDCl_3$) δ 194.43, 145.07, 137.70, 136.81, 136.11, 135.28, 135.17, 133.63, 130.73, 130.06, 129.92, 128.88, 128.49, 128.18, 128.06, 126.90, 126.44, 59.08, 47.22, 21.64. **HRMS** (ESI) m/z calculated for $C_{22}H_{18}Cl_2NaO_3S$ $[M+Na]^+$ 455.0246, found 455.0254.

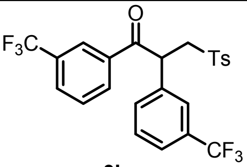

3be

3-Tosyl-1,2-bis(3-(trifluoromethyl)phenyl)propan-1-one (3be): Yellow solid; m.p: 76-77 °C; ^{1}H NMR (600 MHz, $CDCl_3$) δ 8.14 (s, 1H), 8.11 (d, *J* = 7.8 Hz, 1H), 7.80 (d, *J* = 7.8 Hz, 1H), 7.68 (d, *J* = 8.2 Hz, 2H), 7.58 (t, *J* = 7.8 Hz, 1H), 7.51-7.45 (m, 3H), 7.41 (t, *J* = 8.0 Hz, 1H), 7.25 (d, *J* = 8.2 Hz, 2H), 5.36 (dd, *J* = 8.5, 4.2 Hz, 1H), 4.37 (dd, *J* = 14.2, 8.5 Hz, 1H), 3.49 (dd, *J* = 14.2, 4.2 Hz, 1H), 2.40 (s, 3H).^{13}C NMR (150 MHz, $CDCl_3$) δ 194.41, 145.20, 136.63, 136.03, 135.65, 131.93 (q, *J* = 32.5 Hz), 131.87, 131.67, 130.17 (q, *J* = 3.0 Hz), 130.11, 129.96, 129.54, 128.02, 125.72 (q, *J* = 3.0 Hz), 125.27 (q, *J* = 3.0 Hz), 124.89 (q, *J* = 3.0 Hz), 123.51 (q, *J* = 273.0 Hz), 123.44 (q, *J* = 273.0 Hz), 59.08, 47.34, 21.56. **HRMS** (ESI) m/z calculated for $C_{24}H_{18}F_6NaO_3S$ $[M+Na]^+$ 523.0773, found 523.0776.

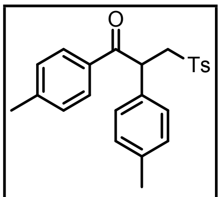

3bf

1,2-Di-*p*-tolyl-3-tosylpropan-1-one (3bf): Yllow solid; m.p: 85-86 °C; ^{1}H NMR (600 MHz, $CDCl_3$) δ 7.79 (d, *J* = 8.2 Hz, 2H), 7.68 (d, *J* = 8.2 Hz, 2H), 7.22 (d, *J* = 8.0 Hz, 2H), 7.16 (d, *J* = 8.0 Hz, 2H), 7.10 (d, *J* = 8.0 Hz, 2H), 7.03 (d, *J* = 8.0 Hz, 2H), 5.21 (dd, *J* = 8.6, 4.0 Hz, 1H), 4.37 (dd, *J* = 14.2, 8.6 Hz, 1H), 3.40 (dd, *J* = 14.2, 4.0 Hz, 1H), 2.38 (s, 3H), 2.34 (s, 3H), 2.23 (s, 3H). ^{13}C NMR (150 MHz, $CDCl_3$) δ 195.52, 144.57, 144.26, 137.68, 136.57, 133.74, 133.01, 129.99, 129.74, 129.26, 129.02, 128.09, 127.99, 59.32, 47.07, 21.64, 21.60, 21.00. **HRMS** (ESI) m/z calculated for $C_{24}H_{24}NaO_3S$ $[M+Na]^+$ 415.1338, found 415.1345.

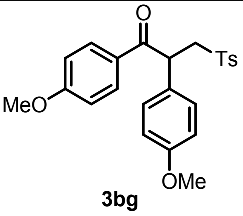

3bg

1,2-Bis(4-methoxyphenyl)-3-tosylpropan-1-one (3bg): Yellow solid; m.p: 88-89 °C;^{1}H NMR (600 MHz, $CDCl_3$) δ 7.88 (d, *J* = 8.8 Hz, 2H), 7.68 (d, *J* = 8.2 Hz, 2H), 7.22 (d, *J* = 8.2 Hz, 2H), 7.14 (d, *J* = 8.8 Hz, 2H), 6.85 (d, *J* = 8.8 Hz, 2H), 6.75 (d, *J* = 8.8 Hz, 2H), 5.18 (dd, *J* = 8.4, 4.0 Hz, 1H), 4.34 (dd, *J* = 14.2, 8.8 Hz, 1H), 3.82 (s, 3H), 3.71 (s, 3H), 3.40 (dd, *J* = 14.2, 4.0 Hz, 1H), 2.39 (s, 3H). ^{13}C NMR (150 MHz, $CDCl_3$) δ 194.45, 163.69, 159.16, 144.53, 136.60, 131.21, 129.71, 129.20, 128.87, 128.45, 128.08, 114.69, 113.78, 59.38, 55.48, 55.23, 46.39, 21.59. **HRMS** (ESI) m/z calculated for $C_{24}H_{24}NaO_5S$ $[M+Na]^+$ 447.1237, found 447.1250.

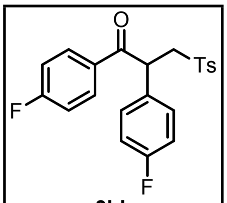

3bh

1,2-Bis(4-fluorophenyl)-3-tosylpropan-1-one (3bh): Yellow solid; m.p: 97-98 °C; ^{1}H NMR (600 MHz, $CDCl_3$) δ 7.93 (dd, *J* = 8.8, 5.4 Hz, 2H), 7.69 (d, *J* = 8.2 Hz, 2H), 7.25 (d, *J* = 8.2 Hz, 2H), 7.21 (dd, *J* = 8.8, 5.4 Hz, 2H), 7.07 (t, *J* = 8.4 Hz, 2H), 6.94 (t, *J* = 8.4 Hz, 2H), 5.25 (dd, *J* = 8.5, 4.0 Hz, 1H), 4.34 (dd, *J* = 14.2, 8.5 Hz, 1H), 3.42 (dd, *J* = 14.2, 4.0 Hz, 1H), 2.40 (s, 3H). ^{13}C NMR (150 MHz, $CDCl_3$) δ 194.41, 165.92 (d, *J* = 254.6 Hz), 162.37 (d, *J* = 246.5 Hz), 144.90, 136.39, 132.05 (d, *J* = 3.3 Hz) , 131.72 (d, *J* = 2.8 Hz), 131.57 (d, *J* = 9.5 Hz), 129.87, 129.84 (d, *J* = 9.5 Hz), 128.03, 116.41 (d, *J* = 21.6 Hz), 115.89 (d, *J* = 21.8 Hz), 59.26, 46.60, 21.60. **HRMS** (ESI) m/z calculated for $C_{22}H_{18}F_2NaO_3S$ $[M+Na]^+$ 423.0837, found 423.0839.

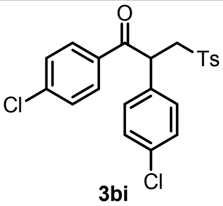

3bi

1,2-Bis(4-chlorophenyl)-3-tosylpropan-1-one (3bi): White solid; m.p: 89-90 °C; ^{1}H NMR (600 MHz, $CDCl_3$) δ 7.83 (d, *J* = 8.4 Hz, 2H), 7.68 (d, *J* = 8.4 Hz, 2H), 7.38 (d, *J* = 8.4 Hz, 2H), 7.26 (d, *J* = 7.8 Hz, 2H), 7.22 (d, *J* = 8.4 Hz, 2H), 7.15 (d, *J* = 8.4 Hz, 2H), 5.21 (dd, *J* = 8.4, 4.2 Hz, 1H), 4.30 (dd, *J* = 14.2, 8.4 Hz, 1H), 3.42 (dd, *J* = 14.2, 4.2 Hz, 1H), 2.41 (s, 3H). ^{13}C NMR (150 MHz, $CDCl_3$) δ 194.61, 144.96, 140.24, 136.33, 134.54, 134.30, 133.57, 130.23, 129.89, 129.64, 129.50, 129.08, 128.00, 59.04, 46.86, 21.63. **HRMS** (ESI) m/z calculated for $C_{22}H_{18}Cl_2NaO_3S$ $[M+Na]^+$ 455.0246, found 455.0243.

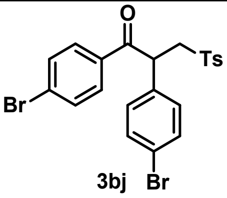

3bj

1,2-Bis(4-bromophenyl)-3-tosylpropan-1-one (3bj): White solid; m.p.: 101-103 °C; ^{1}H NMR (600 MHz, $CDCl_3$) δ 7.67 (d, *J* = 8.4 Hz, 2H), 7.59 (d, *J* = 8.4 Hz, 2H), 7.47 (d, *J* = 8.4 Hz, 2H), 7.29 (d, *J* = 8.4 Hz, 2H), 7.18 (d, *J* = 8.4 Hz, 2H), 7.01 (d, *J* = 8.4 Hz, 2H), 5.12 (dd, *J* = 8.4, 4.2 Hz, 1H), 4.21 (dd, *J* = 14.4, 8.4 Hz, 1H), 3.35 (dd, *J* = 14.4, 4.2 Hz, 1H), 2.34 (s, 3H). ^{13}C NMR (150 MHz, $CDCl_3$) δ 194.75, 144.99, 136.28, 134.99, 133.93, 132.60, 132.10, 130.31, 129.90, 129.83, 129.05, 128.00, 122.43, 58.93, 46.92, 21.66. **HRMS** (ESI) m/z calculated for $C_{22}H_{18}Br_2NaO_3S$ $[M+Na]^+$ 544.9213, found 544.9221.

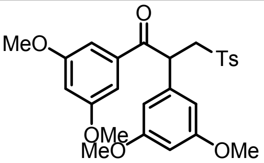

3bk

1,2-Bis(3,5-dimethoxyphenyl)-3-tosylpropan-1-one (3bk): Yellow oil; ^{1}H NMR (600 MHz, $CDCl_3$) δ 7.70 (d, *J* = 8.0 Hz, 2H), 7.34-7.14 (m, 2H), 7.03 (d, 2H), 6.59 (t, *J* = 1.8 Hz, 1H), 6.33 (d, *J* = 1.8 Hz, 2H), 6.27 (t, *J* = 1.8 Hz, 1H), 5.08 (dd, *J* = 8.8, 3.6 Hz, 1H), 4.37 (dd, *J* = 14.2, 8.8 Hz, 1H), 3.78 (s, 6H), 3.70 (s, 6H), 3.39 (dd, *J* = 14.2, 3.6 Hz, 1H), 2.40 (s, 3H). ^{13}C NMR (150 MHz, $CDCl_3$) δ 195.21, 161.38, 160.76, 144.68, 138.62, 137.38, 136.43, 129.78, 128.12, 106.73, 106.08, 105.82, 99.79, 59.17, 55.55, 55.36, 48.00, 21.60. **HRMS** (ESI) m/z calculated for $C_{26}H_{28}NaO_7S$ $[M+Na]^+$ 507.1448, found 507.1449.

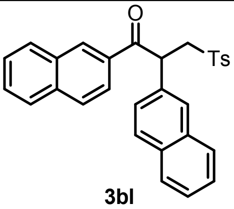

3bl

1,2-Di(naphthalen-2-yl)-3-tosylpropan-1-one (3bl): Yellow oil; ^{1}H NMR (600 MHz, $CDCl_3$) δ 8.50 (s, 1H), 7.94 (d, *J* = 8.8 Hz, 1H), 7.90 (d, *J* = 8.2 Hz, 1H), 7.78 (d, *J* = 8.8 Hz, 2H), 7.74-7.66 (m, 6H), 7.54 (t, *J* = 7.2 Hz, 1H), 7.51-7.47 (m, 1H), 7.43-7.39 (m, 3H), 7.11 (d, *J* = 8.2 Hz, 2H), 5.61 (dd, *J* = 8.2, 4.2 Hz, 1H), 4.51 (dd, *J* = 14.4, 8.2 Hz, 1H), 3.62 (dd, *J* = 14.4, 4.2 Hz, 1H), 2.27 (s, 3H). ^{13}C NMR (150 MHz, $CDCl_3$) δ 195.85, 144.68, 136.43, 135.66, 133.83, 133.49, 132.85, 132.71, 132.35, 130.89, 129.75, 129.72, 129.41, 128.80, 128.51, 128.06, 127.83, 127.70, 127.62, 127.56, 126.86, 126.55, 126.46, 125.57, 124.33, 59.33, 47.90, 21.51. **HRMS** (ESI) m/z calculated for $C_{30}H_{24}NaO_3S$ $[M+Na]^+$ 487.1338, found 487.1347.

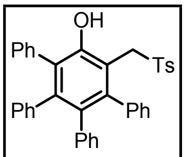

3ca

5',6'-Diphenyl-4'-(tosylmethyl)-[1,1':2',1''-terphenyl]-3'-ol (3ca): Yellow solid; m.p: 189-190 °C; ^{1}H NMR (600 MHz, $CDCl_3$) δ 7.56 (d, *J* = 8.2 Hz, 2H), 7.28-7.25 (m, 3H), 7.22 (t, *J* = 7.4 Hz, 2H), 7.19-7.13 (m, 3H), 7.11-7.07 (m, 3H), 6.98-6.97 (m, 2H), 6.88-6.82 (m, 3H), 6.81-6.74 (m, 5H), 6.70 (d, *J* = 7.4 Hz, 2H), 4.49 (s, 2H), 2.43 (s, 3H). ^{13}C NMR (150 MHz, $CDCl_3$) δ 151.94, 144.79, 143.99, 142.89, 139.96, 139.52, 138.77, 136.54, 136.14, 134.93, 131.46, 130.96, 130.94, 130.70, 129.72, 129.63, 128.31, 128.05, 127.35, 127.04, 126.72, 126.63, 126.56, 125.64, 125.24, 113.80, 57.06, 21.67. **HRMS** (ESI) m/z calculated for $C_{38}H_{30}NaO_3S$ $[M+Na]^+$ 589.1808, found 589.2803.

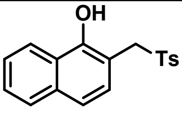

3cb

2-(Tosylmethyl)naphthalen-1-ol (3cb): Brown solid; m.p: 158-161 °C; ^{1}H NMR (600 MHz, $CDCl_3$) δ 8.38 (d, *J* = 7.2 Hz, 1H), 7.92 (s, 1H), 7.74 (d, *J* = 7.2 Hz, 1H), 7.60 (d, *J* = 8.4 Hz, 2H), 7.54-7.51 (m, 2H), 7.26-7.24 (m, 3H), 6.68 (d, *J* = 8.4 Hz, 1H), 4.57 (s, 2H), 2.43 (s, 3H). ^{13}C NMR (150 MHz, $CDCl_3$) δ152.51, 145.43, 134.92, 133.95, 129.84, 128.57, 128.54, 127.37, 127.22, 126.47, 125.86, 122.87, 121.16, 109.51, 60.17, 21.70. **HRMS** (ESI) m/z calculated for $C_{18}H_{16}NaO_3S$ $[M+Na]^+$ 335.0711, found 335.0718.

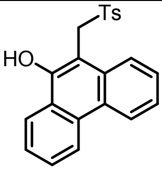

3cc

10-(Tosylmethyl)phenanthren-9-ol (3cc): White solid; m.p: 171-172 °C; ^{1}H NMR (600 MHz, $CDCl_3$) δ 8.63 (d, *J* = 8.2 Hz, 1H), 8.58 (d, *J* = 8.2 Hz, 1H), 8.54 (d, *J* = 8.0 Hz, 1H), 8.28 (s, 1H), 7.76-7.66 (m, 4H), 7.44-7.41 (m, 1H), 7.34-7.30 (m, 2H), 7.18 (d, *J* = 8.0 Hz, 2H), 4.94 (s, 2H), 2.34 (s, 3H). ^{13}C NMR (150 MHz, $CDCl_3$) δ 151.87, 145.55, 134.44, 131.72, 131.09, 129.87, 128.60, 128.19, 127.02, 127.00, 126.88, 126.66, 124.36, 123.70, 122.98, 122.41, 122.17, 105.03, 55.16, 21.61. **HRMS** (ESI) m/z calculated for $C_{22}H_{18}NaO_3S$ $[M+Na]^+$ 385.0869, found 385.0870.

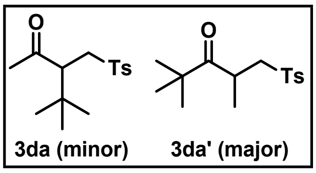

4,4-Dimethyl-3-(tosylmethyl)pentan-2-one (3da, minor): 2,4,4-trimethyl-1-tosylpentan-3-one (3da’, major) = 1:3. ^{1}H NMR (600 MHz, $CDCl_3$) δ 7.78-7.75 (m, 2.67H, major (2H)+ minor (0.67H)), 7.37-7.34 (m, 2.67H, major (2H) + minor (0.67H)), 3.72 (dd, *J* = 12.4, 6.6 Hz, 1H, major), 3.69 (dd, *J* = 12.4, 6.6 Hz, 0.33H, minor), 3.51 (dd, *J* = 13.8, 6.6 Hz, 1H, major), 3.06 (dd, *J* = 13.8, 6.0 Hz, 0.67H, minor), 3.02 (dd, *J* = 13.8, 6.0 Hz, 1H), 2.46 (s, 1H, minor), 2.45 (s, 3H, major), 2.30 (s, 1H, minor), 1.21 (d, *J* = 7.2 Hz, 3H, major), 1.19 (s, 9H, major), 0.92 (s, 3H, minor). ^{13}C NMR (150 MHz, $CDCl_3$) δ **3da**: 209.84, 144.93, 136.32, 129.98, 127.96, 56.31, 53.87, 33.97, 28.76, 27.53, 21.67; **3da’**: 216.14, 144.86, 136.89, 129.98, 127.82, 59.38, 44.91, 34.24, 26.63, 21.65, 19.05. **HRMS** (ESI) m/z calculated for $C_{15}H_{22}NaO_3S$ $[M+Na]^+$ 305.1192, found 305.1187.

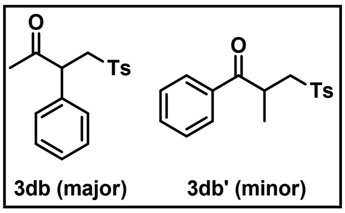

3-Phenyl-4-tosylbutan-2-one (3db, major) : 2-methyl-1-phenyl -3-tosylpropan-1-one (3db’, minor) = 2:1. ^{1}H NMR (600 MHz, $CDCl_3$) δ 7.89 (d, *J* = 7.8 Hz, 1H, minor), 7.73 (d, *J* = 7.8 Hz, 1H, minor), 7.71 (d, *J* = 7.8 Hz, 2H, major), 7.58 (t, *J* = 7.2 Hz, 0.5H, minor), 7.46 (t, *J* = 7.2 Hz, 1H, major), 7.26-7.28 (m, 6H, major (4H)+minor (1H)), 7.12 (d, *J* = 7.2 Hz, 2H, major), 4.34-4.36 (m, 1H, major), 4.20 (dd, *J* = 13.8, 7.8 Hz, 1H, major), 4.18-4.12 (m, 0.5H, minor), 3.89 (dd, *J* = 14.0, 7.8 Hz, 0.5H, minor), 3.29 (dd, *J* = 13.8, 4.2 Hz, 1H, major), 3.16 (dd, *J* = 14.0, 5.0 Hz, 0.5H, minor), 2.42 (s, 3H, major), 2.40 (s, 1.5H, minor),2.10 (s, 3H, major), 1.34 (d, *J* = 7.2 Hz, 1.5H, minor). ^{13}C NMR (150 MHz, $CDCl_3$) δ **3db**: 204.09, 144.75, 136.59, 135.88, 133.56, 129.85, 129.34, 128.19, 127.92, 57.94, 52.76, 28.77, 21.61; **3db’**: 200.12, 144.84, 136.47, 134.96, 129.91, 128.77, 128.50, 128.16, 128.04, 58.73, 35.65, 21.60, 18.73. **HRMS** (ESI) m/z calculated for $C_{17}H_{18}NaO_3S$ $[M+Na]^+$ 325.0878, found 325.0874.

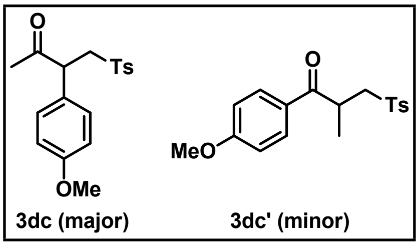

3-(4-Methoxyphenyl)-4-tosylbutan-2-one (3dc major): 1-(4-methoxyphenyl)-2-methyl-3-tosylpropan-1-one (3dc’ minor) = 3:1. ^{1}H NMR (600 MHz, $CDCl_3$) δ 7.81 (d, *J* = 8.4 Hz, 0.67H, minor), 7.65 (d, *J* = 7.8 Hz, 0.67H, minor), 7.61 (d, *J* = 7.8 Hz, 2H, major), 7.24-7.18 (m, 2.67H, major (2H)+minor (0.67H)), 6.96 (d, *J* = 8.4 Hz, 2H, major), 6.86 (d, *J* = 8.4 Hz, 0.67H, minor), 6.72 (d, *J* = 8.4 Hz, 2H, major), 4.22 (dd, *J* = 7.8, 4.8 Hz, 1H, major), 4.08 (dd, *J* = 14.4, 7.8 Hz, 1H, major), 4.06-4.00 (m, 0.33H, minor), 3.81 (s, 1H, minor), 3.79 (dd, *J* = 14.4, 7.2 Hz, 0.33H, minor), 3.69 (s, 3H, major), 3.20 (dd, *J* = 14.4, 4.8 Hz, 1H, major), 3.06 (dd, *J* = 14.4, 5.4 Hz, 0.33H, minor), 2.35 (s, 3H, major), 2.33 (s, 1H, minor), 2.02 (s, 3H, major), 1.25 (d, *J* = 7.2 Hz, 1H, minor). ^{13}C NMR (150 MHz, $CDCl_3$) δ **3dc**: 204.34, 159.47, 144.66, 136.70, 130.86, 129.82, 129.31, 127.92, 114.71, 58.02, 55.30, 51.93, 28.65, 21.64; **3dc’**: 198.56, 163.89, 144.76, 136.54, 129.86, 128.05, 127.87, 127.70, 113.94, 58.83, 55.56, 35.24, 21.64, 18.94. **HRMS** (ESI) m/z calculated for $C_{18}H_{20}NaO_4S$ $[M+Na]^+$ 355.0969, found 355.0980.

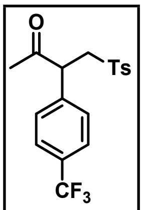
3dd

4-Tosyl-3-(4-(trifluoromethyl)phenyl)butan-2-one (3dd): Yellow oil. ^{1}H NMR (600 MHz, $CDCl_3$) δ 7.64 (d, *J* = 8.4 Hz, 2H), 7.52 (d, *J* = 8.4 Hz, 2H), 7.26-7.25 (m, 4H), 4.43 (t, *J* = 7.2 Hz, 1H), 4.12 (dd, *J* = 14.4, 7.2 Hz, 1H), 3.35 (dd, *J* = 14.4, 6.0 Hz, 1H), 2.41 (s, 3H), 2.12 (s, 3H). ^{13}C NMR (150 MHz, $CDCl_3$) δ 203.33, 144.95, 139.60, 136.39, 130.54 (q, *J* = 32.4 Hz), 129.87, 128.73, 127.85, 126.23 (q, *J* = 3.8 Hz), 123.90 (q, *J* = 272.0 Hz), 57.77, 52.70, 28.92, 21.56. **HRMS** (ESI) m/z calculated for $C_{18}H_{17}F_3NaO_3S$ $[M+Na]^+$ 393.0742, found 393.0748.

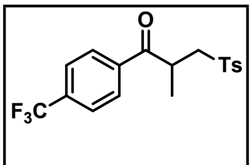
3dd'

2-Methyl-3-tosyl-1-(4-(trifluoromethyl)phenyl) propan-1-one (3dd'): Yellow oil. ^{1}H NMR (600 MHz, $CDCl_3$) δ 8.03 (d, *J* = 8.4 Hz, 2H), 7.76-7.72 (m, 4H), 7.30 (d, *J* = 8.4 Hz, 2H), 4.19-4.16 (m, 1H), 3.90 (dd, *J* = 14.4, 7.8 Hz, 1H), 3.17 (dd, *J* = 14.4, 4.8 Hz, 1H), 2.42 (s, 3H), 1.35 (d, *J* = 7.2 Hz, 3H). ^{13}C NMR (150 MHz, $CDCl_3$) δ 199.43, 145.03, 137.81, 136.39, 134.81 (q, *J* = 32.6 Hz), 129.98, 128.88, 128.01, 125.86 (q, *J* = 3.8 Hz), 125.47 (q, *J* = 272.0 Hz), 58.71, 35.87, 21.63, 18.53. **HRMS** (ESI) m/z calculated for $C_{18}H_{17}F_3NaO_3S$ $[M+Na]^+$ 393.0742, found 393.0748.

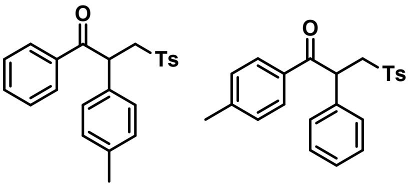
3de (major) 3de' (minor)

1-Phenyl-2-(p-tolyl)-3-tosylpropan-1-one (3de, major) : 2-phenyl-1-(p-tolyl)-3-tosylpropan-1-one (3de', minor) = 4:3. ^{1}H NMR (600 MHz, $CDCl_3$) δ 7.88 (d, *J* = 8.4 Hz, 2H, major), 7.80 (d, *J* = 7.8 Hz, 1.5H, minor), 7.68 (d, *J* = 7.8 Hz, 3.5H, major (2H)+minor (1.5H)), 7.48 (t, *J* = 7.8 Hz, 1.5H, minor), 7.37 (t, *J* = 7.8 Hz, 2H, major), 7.23-7.19 (m, 6H, major(2H)+minor(4H)), 7.18-7.16 (m, 1.75H, major(1H)+minor(0.75H)), 7.11 (d, *J* = 8.4 Hz, 2H, major), 7.03 (d, *J* = 7.8 Hz, 2H, major), 5.27-5.23 (m, 1.75H, major(1H)+minor(0.75H)), 4.42-4.37 (m, 1.75H, major(1H)+minor(0.75H)), 3.43-3.39 (m, 1.75H, major(1H)+minor(0.75H)), 2.38 (s, 5.25H, major(3H)+minor(2.25)), 2.34 (s, 2.25H, minor), 2.23 (s, 3H, major). ^{13}C NMR (150 MHz, $CDCl_3$) δ **3de**: 195.98, 144.65, 137.80, 136.48, 133.35, 130.06, 129.78, 129.33, 129.30, 128.88, 128.56, 128.11, 128.02, 59.35, 47.21, 21.61, 21.02; **3de'**: 195.43, 144.38, 136.80, 135.54, 133.47, 132.98, 129.79, 129.04, 128.88, 128.14, 128.02, 127.87, 125.98, 59.30, 47.42, 21.66, 21.02. **HRMS** (ESI) m/z calculated for $C_{23}H_{22}NaO_3S$ $[M+Na]^+$ 401.1180, found 401.1187.

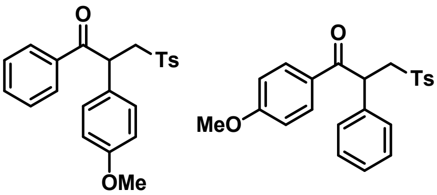
3df (major) 3df' (minor)

2-(4-Methoxyphenyl)-1-phenyl-3-tosylpropan-1-one (3df, major) : 1-(4-methoxyphenyl)-2-phenyl-3-tosylpropan-1-one (3df', minor) = 6:5. ^{1}H NMR (600 MHz, $CDCl_3$) δ 7.89-7.88 (m, 3.67H, major(2H)+minor(1.67H)), 7.69-7.68 (m, 3.67H, major(2H)+minor(1.67H)), 7.49 (t, *J* = 7.2 Hz, 1H, major), 7.37 (t, *J* = 7.8 Hz, 2H, major), 7.24-7.20 (m, 7H, major(2H)+minor(5H)), 7.19-7.17 (m, 0.83H, minor), 7.14 (d, *J* = 9.0 Hz, 2H, major), 6.85 (d, *J* =

8.4 Hz, 1.67H, minor), 6.76 (d, *J* = 9.0 Hz, 2H, major), 5.23 (dd, *J* = 9.0, 4.2 Hz, 1.83H, major(1H)+minor(0.83H)), 4.42-4.35 (m, 1.83H, major(1H)+minor(0.83H)), 3.81 (s, 2.53H, minor), 3.71 (s, 3H, major), 3.43-3.40 (m, 1.83H, major(1H)+minor(0.83H)), 2.38 (s, 5.55H, major(3H)+minor(2.55H)). ^{13}C NMR (150 MHz, $CDCl_3$) δ **3df**: 196.05, 159.26, 144.63, 135.54, 133.33, 131.25, 129.78, 129.30, 128.86, 128.57, 128.11, 128.08, 114.77, 59.37, 55.23, 46.76, 21.60; **3df'**: 194.25, 163.76, 137.04, 136.52, 136.50, 129.76, 128.57, 128.45, 128.32, 128.11, 128.08, 127.83, 113.81, 59.33, 55.49, 47.19, 21.60. **HRMS** (ESI) m/z calculated for $C_{23}H_{22}NaO_4S$ $[M+Na]^+$ 417.1127, found 417.1136.

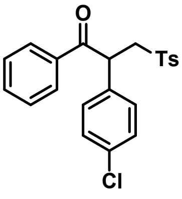

3dg (minor)

2-(4-Chlorophenyl)-1-phenyl-3-tosylpropan-1-one (3dg): White solid; m.p: 95-97 °C; ^{1}H NMR (600 MHz, $CDCl_3$) δ 7.87 (d, *J* = 7.8 Hz, 2H), 7.66 (d, *J* = 7.8 Hz, 2H), 7.52 (t, *J* = 7.2 Hz, 1H), 7.40 (t, *J* = 7.8 Hz, 2H), 7.25-7.18 (m, 4H), 7.16 (d, J = 8.4 Hz, 2H), 5.27 (dd, *J* = 8.4, 4.8 Hz, 1H), 4.31 (dd, *J* = 14.4, 8.4 Hz, 1H), 3.44 (d, *J* = 14.4, 4.8 Hz, 1H), 2.40 (s, 3H). ^{13}C NMR (150 MHz, $CDCl_3$) δ 195.70, 144.86, 136.35, 135.22, 134.87, 134.09, 133.66, 129.84, 129.58, 129.52, 128.86, 128.71, 128.04, 59.12, 46.87, 21.61. **HRMS** (ESI) m/z calculated for $C_{22}H_{19}NaO_3S$ $[M+Na]^+$ 421.0638, found 421.0641.

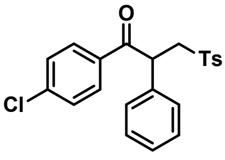

3dg' (major)

1-(4-chlorophenyl)-2-phenyl-3-tosylpropan-1-one (3dg'): White solid; m.p: 94-95 °C; ^{1}H NMR (600 MHz, $CDCl_3$) δ 7.84 (d, *J* = 8.4 Hz, 2H), 7.70 (d, *J* = 8.4 Hz, 2H), 7.36 (d, *J* = 7.8 Hz, 2H), 7.26-7.24 (m, 4H), 7.22-7.20 (m, 3H), 5.22 (dd, *J* = 8.4, 3.6 Hz, 1H), 4.40 (dd, *J* = 13.8, 8.4 Hz, 1H), 3.40 (dd, *J* = 13.8, 3.6 Hz, 1H), 2.41 (s, 3H). ^{13}C NMR (150 MHz, $CDCl_3$) δ 194.79, 144.81, 139.95, 136.43, 136.20, 133.87, 130.26, 129.86, 129.49, 128.96, 128.14, 128.09, 128.07, 59.23, 47.56, 21.62. **HRMS** (ESI) m/z calculated for $C_{22}H_{19}NaO_3S$ $[M+Na]^+$ 421.0636, found 421.0641.

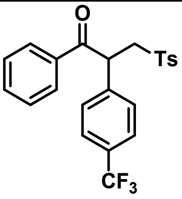

3dh (minor)

1-Phenyl-3-tosyl-2-(4-(trifluoromethyl)phenyl)propan-1-one (3dh): Yellow oil; ^{1}H NMR (600 MHz, $CDCl_3$) δ 7.90 (d, *J* = 8.4 Hz, 2H), 7.65 (d, *J* = 7.8 Hz, 2H), 7.54 (t, *J* = 7.8 Hz, 1H), 7.48 (d, *J* = 8.4 Hz, 2H), 7.42 (t, *J* = 8.4 Hz, 2H), 7.36 (d, *J* = 8.4 Hz, 2H), 7.21 (d, *J* = 8.4 Hz, 2H), 5.37 (dd, *J* = 7.8, 5.4 Hz, 1H), 4.31 (dd, *J* = 14.4, 7.8 Hz, 1H), 3.51 (dd, *J* = 14.4, 5.4 Hz, 1H), 2.39 (s, 3H). ^{13}C NMR (150 MHz, $CDCl_3$) δ 195.46, 144.91, 140.24, 136.26, 135.10 (q, *J* = 30.0 Hz), 133.84, 129.83, 128.88, 128.79, 128.72, 128.00, 126.22 (q, *J* = 3.0 Hz), 123.75 (q, *J* = 272.0 Hz), 59.01, 47.31, 21.55. **HRMS** (ESI) m/z calculated for $C_{23}H_{19}F_3NaO_3S$ $[M+Na]^+$ 455.0912, found 455.0905.

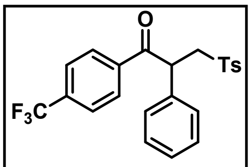

3dh' (major)

2-Phenyl-3-tosyl-1-(4-(trifluoromethyl)phenyl)propan-1-one (3dh'): Yellow oil; ^{1}H NMR (600 MHz, $CDCl_3$) δ 8.01 (d, *J* = 8.4 Hz, 2H), 7.72 (d, *J* = 8.4 Hz, 2H), 7.66 (d, *J* = 8.4 Hz, 2H), 7.28-7.26 (m, 4H), 7.24-7.21 (m, 3H), 5.27 (dd, *J* = 9.0, 3.6 Hz, 1H), 4.42 (dd, *J* = 13.8, 9.0 Hz, 1H),

3.42 (dd, J = 13.8, 3.0 Hz, 1H), 2.41 (s, 3H). ^{13}C NMR (150 MHz, CDCl$_3$) δ 195.20, 144.90, 138.35, 136.41, 135.75, 134.60 (q, J = 30.0 Hz), 129.90, 129.60, 129.17, 128.30, 128.13, 128.05, 125.68 (q, J = 3.0 Hz), 123.45 (q, J = 273.0 Hz), 59.24, 47.87, 21.62. **HRMS** (ESI) m/z calculated for $C_{23}H_{19}F_3NaO_3S$ [M+Na]$^+$ 455.0910, found 455.0905.

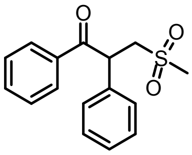

3ea

3-(methylsulfonyl)-1,2-diphenylpropan-1-one (3ea): White solid; m.p: 88-89 °C; ^{1}H NMR (600 MHz, CDCl$_3$) δ 7.98 (d, J = 8.4 Hz, 2H), 7.51 (t, J = 7.4 Hz, 1H), 7.40 (t, J = 7.8 Hz, 2H), 7.32-7.35 (m, 4H), 7.25-7.28 (m, 1H), 5.29 (dd, J = 8.2, 5.0 Hz, 1H), 4.20 (dd, J = 14.4, 8.2 Hz, 1H), 3.41 (dd, J = 14.4, 5.0 Hz, 1H), 2.72 (s, 3H). ^{13}C NMR (150 MHz, CDCl$_3$) δ 196.57, 136.24, 135.26, 133.70, 129.69, 129.05, 128.77, 128.35, 128.31, 58.17, 48.48, 42.52. **HRMS** (ESI) m/z calculated for $C_{16}H_{16}NaO_3S$ [M+Na]$^+$ 311.0712, found 311.0720.

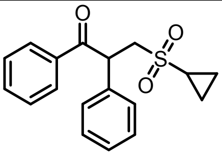

3eb

3-(cyclopropylsulfonyl)-1,2-diphenylpropan-1-one (3eb): Yellow oil; ^{1}H NMR (600 MHz, CDCl$_3$) δ 7.99 (d, J = 7.8 Hz, 2H), 7.51 (t, J = 7.2 Hz, 1H), 7.40 (t, J = 7.8 Hz, 2H), 7.35 (d, J = 7.2 Hz, 2H), 7.31 (t, J = 7.8 Hz, 2H), 7.25 (t, J = 7.2 Hz, 1H), 5.31 (dd, J = 8.4, 4.5 Hz, 1H), 4.31 (dd, J = 14.4, 8.4 Hz, 1H), 3.41 (dd, J = 14.4, 4.5 Hz, 1H), 2.19-2.11 (m, 1H), 1.24-1.17 (m, 1H), 1.14-1.08 (m, 1H), 0.98-0.92 (m, 1H), 0.86-0.79 (m, 1H). ^{13}C NMR (150 MHz, CDCl$_3$) δ 196.50, 136.64, 135.42, 133.59, 129.53, 128.98, 128.76, 128.31, 128.18, 57.13, 47.95, 30.80, 5.51, 4.87. **HRMS** (ESI) m/z calculated for $C_{18}H_{18}NaO_3S$ [M+Na]$^+$ 337.0869, found 337.0873.

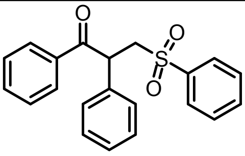

3ec

1,2-diphenyl-3-(phenylsulfonyl)propan-1-one (3ec): White solid; m.p: 100-101 °C; ^{1}H NMR (600MHz, CDCl$_3$) δ 7.90 (d, J = 7.8 Hz, 2H), 7.82 (d, J = 7.8 Hz, 2H), 7.56 (t, J = 7.2 Hz, 1H), 7.48 (t, J = 7.4 Hz, 1H), 7.43 (t, J = 7.8 Hz, 2H), 7.37 (t, J = 7.8 Hz, 2H), 7.24-7.22 (m, 4H), 7.20-7.16 (m, 1H), 5.30 (dd, J = 8.4, 3.6 Hz, 1H), 4.43 (dd, J = 14.2, 8.4 Hz, 1H), 3.46 (dd, J = 14.2, 3.6 Hz, 1H). ^{13}C NMR (150 MHz, CDCl$_3$) δ 195.83, 139.43, 136.42, 135.46, 133.74, 133.48, 129.42, 129.22, 128.91, 128.65, 128.17, 128.05, 128.04, 59.20, 47.54. **HRMS** (ESI) m/z calculated for $C_{21}H_{18}NaO_3S$ [M+Na]$^+$ 373.0869, found 373.0875.

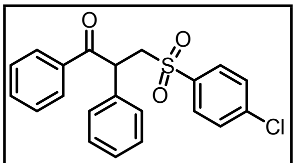

3ed

3-((4-chlorophenyl)sulfonyl)-1,2-diphenylpropan-1-one (3ed): Yellow solid; m.p: 94-95 °C; ^{1}H NMR (600 MHz, CDCl$_3$) δ 7.89 (d, J = 8.0 Hz, 2H), 7.72 (d, J = 8.4 Hz, 2H), 7.50 (t, J = 7.4 Hz, 1H), 7.44-7.35 (m, 4H), 7.29-7.13 (m, 5H), 5.28 (dd, J = 8.4, 4.2 Hz, 1H), 4.40 (dd, J = 14.4, 8.4 Hz, 1H), 3.48 (dd, J = 14.4, 4.2 Hz, 1H). ^{13}C NMR (150 MHz, CDCl$_3$) δ 195.75, 140.47, 137.90, 136.15, 135.34, 133.60, 129.58, 129.48, 128.87, 128.69, 128.17, 128.12, 59.33, 47.67. **HRMS** (ESI) m/z calculated for $C_{21}H_{17}ClNaO_3S$ [M+Na]$^+$ 407.0479, found 407.0480.

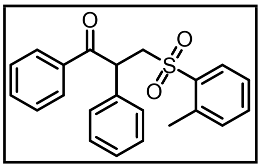

1,2-Diphenyl-3-(o-tolylsulfonyl)propan-1-one (3ee): White solid; m.p.: 64-65 °C; ^{1}H NMR (600 MHz, $CDCl_3$) δ 7.86-7.85 (m, 3H), 7.49 (t, *J* = 7.2 Hz, 1H), 7.41 (t, *J* = 7.2 Hz, 1H), 7.36 (t, *J* = 7.8 Hz, 2H), 7.28 (d, *J* = 7.2 Hz, 1H), 7.26-7.23 (m, 4H), 7.20-7.15 (m, *2*H), 5.28 (dd, *J* = 8.4, 3.0 Hz, 1H), 4.51 (dd, *J* = 14.4, 8.4 Hz, 1H), 3.42 (dd, *J* = 14.4, 3.0 Hz, 1H). ^{13}C NMR (150 MHz, $CDCl_3$) δ 195.86, 138.45, 137.17, 136.55, 135.47, 133.76, 133.41, 132.70, 130.09, 129.40, 128.85, 128.56, 128.09, 128.00, 126.37, 58.20, 47.39, 20.33. **HRMS** (ESI) m/z calculated for $C_{22}H_{20}NaO_3S$ $[M+Na]^+$ 387.1022, found 387.1031.

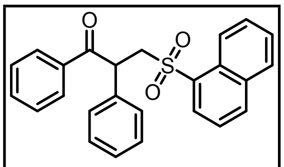

3ef

3-(naphthalen-1-ylsulfonyl)-1,2-diphenylpropan-1-one (3ef): White solid; m.p: 106-107 °C; ^{1}H NMR (600 MHz, $CDCl_3$) δ 8.66 (d, *J* = 8.4 Hz, 1H), 8.07 (d, *J* = 7.2 Hz, 1H), 7.93 (d, *J* = 8.2 Hz, 1H), 7.83 (d, *J* = 8.2 Hz, 1H), 7.79 (d, *J* = 7.8 Hz, 2H), 7.64 (t, *J* = 7.8 Hz, 1H), 7.52 (t, *J* = 7.5 Hz, 1H), 7.39 (t, *J* = 7.2 Hz, 1H), 7.31 (t, *J* = 7.2 Hz, 1H), 7.27 (t, *J* = 7.8 Hz, 2H) 7.11-7.00 (m, 5H), 5.28 (dd, *J* = 8.6, 4.0 Hz, 1H), 4.54 (dd, *J* = 14.2, 8.6 Hz, 1H), 3.54 (dd, *J* = 14.2, 4.0 Hz, 1H). ^{13}C NMR (150 MHz, $CDCl_3$) δ 195.82, 136.27, 135.48, 135.32, 134.32, 134.13, 133.43, 130.55, 129.26, 129.21, 128.92, 128.88, 128.62, 128.13, 127.95, 127.10, 124.22, 124.16, 58.72, 47.48. **HRMS** (ESI) m/z calculated for $C_{25}H_{20}NaO_3S$ $[M+Na]^+$ 423.1025, found 423.1025.

4. Investigation of mechanism

4.1 Control experiments

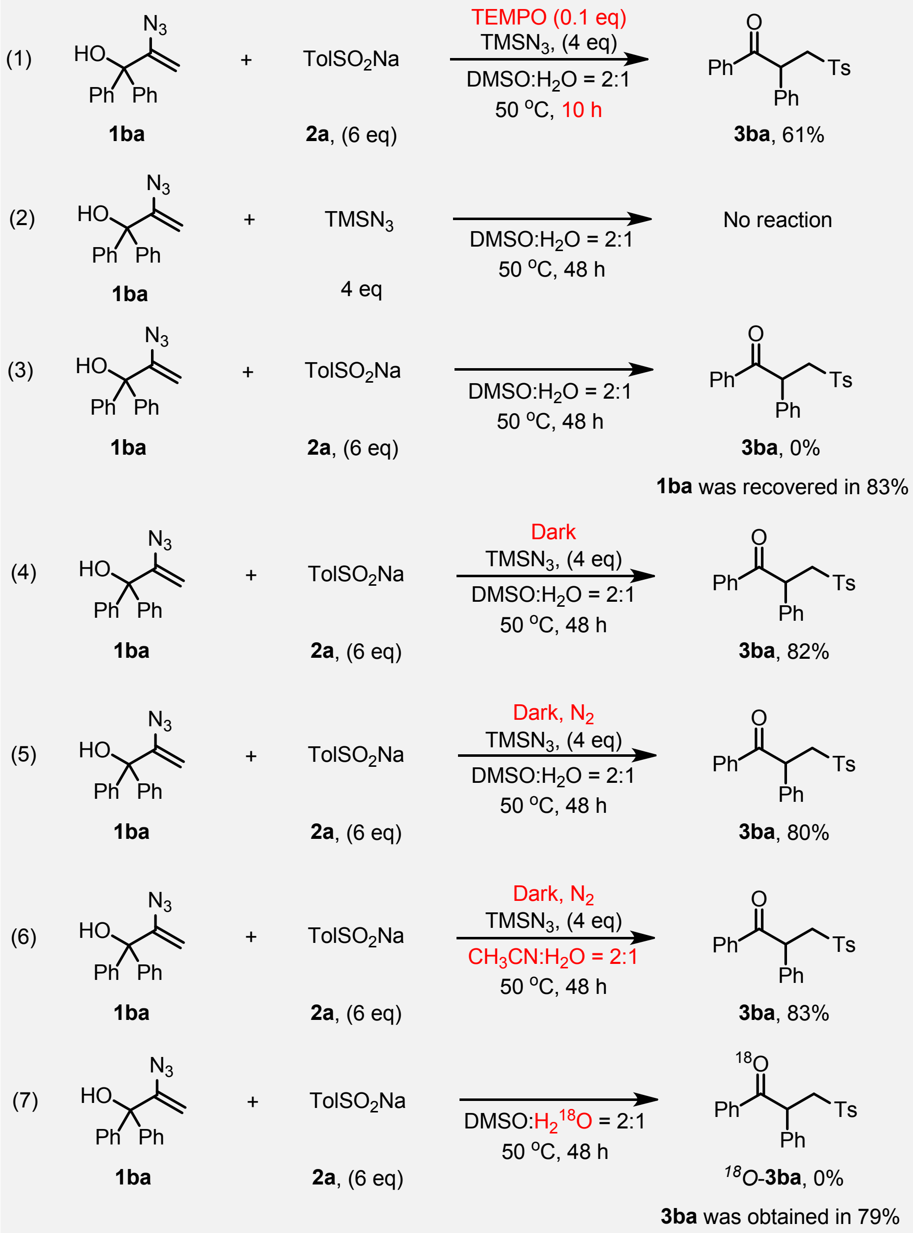

Figure S1. Control experiments

The catalytic amount of TEMPO were beneficial to accelerate the rate of the transformation,[3] despite the yield of desired product would be lowered (eq. 1). Further information on the generation of sulfonyl radical was obtained by submission of allylic alcohols (**1ba**) to various reaction conditions (eq. 2 and 3), which revealed a dual role of $TMSN_3$: in the absence of $TMSN_3$, no reaction took place, whereas in the presence of 4 equivalents, product **3ba** was afforded in high yield (84%). This implies that $TMSN_3$ plays a critical role in the generation of sulfonyl radical. Meanwhile, the results were listed in eq. 4-6 showed that solvent, oxygen, and light all did not affect the generation of sulfonyl radical. Therefore, the sulfonyl radical may be generated from the homolysis process under the action of $TMSN_3$. The experiment of eq. 7 demonstrated that the oxygen of carbonyl in the product was not from water.

4.2 Deuterium-labeling studies

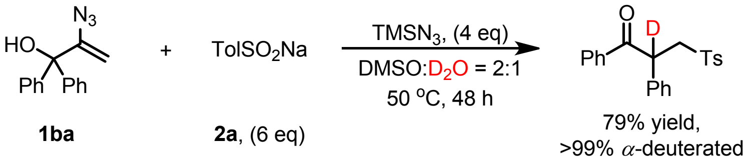

A Schlenk tube was placed compound **1ba** (0.5 mmol), $TolSO_2Na$ (3.0 mmol), $TMSN_3$ (2.0 mmol), D_2O (0.7 mL), and DMSO (1.4 mL). Then the mixture was stirred at 50 °C for 48h. Upon completion of the reaction, saturated NH_4Cl (aq.) was added to quench the reaction and the mixture was extracted with CH_2Cl_2 (3 × 10 mL). The combined organic layers were washed with brine, dried over $MgSO_4$, filtered, and concentrated *in vacuo*. The resulting mixture was purified by silica gel column chromatography using (petroleum ether:ethyl acetate = 6:1) to afford deuterated product ***d*-3ba** in 79% yield.

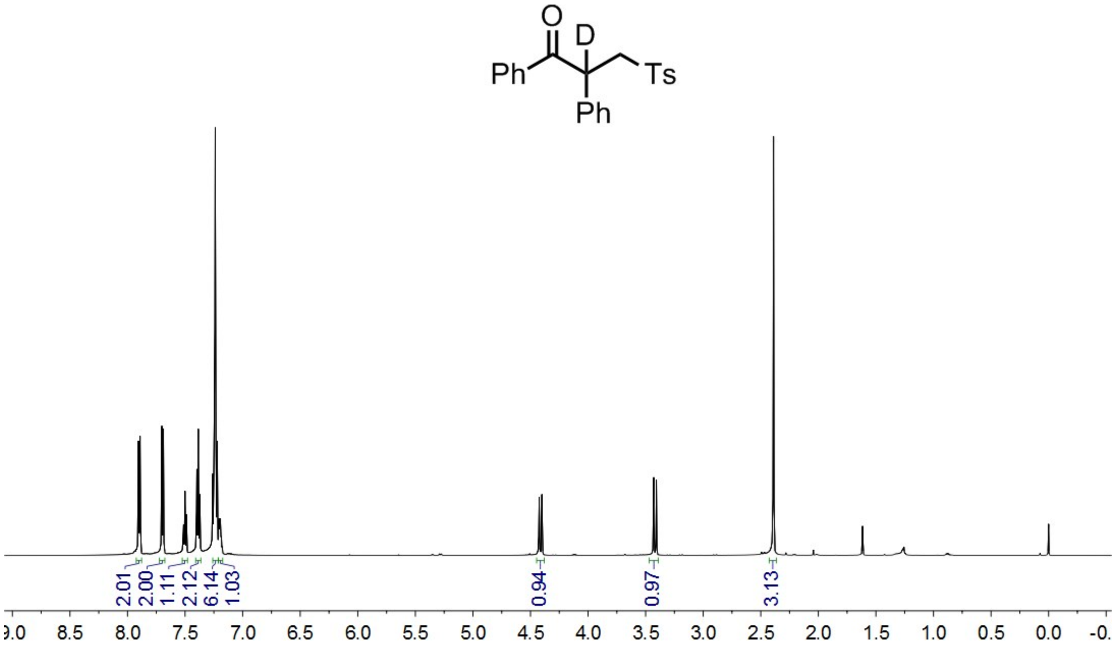

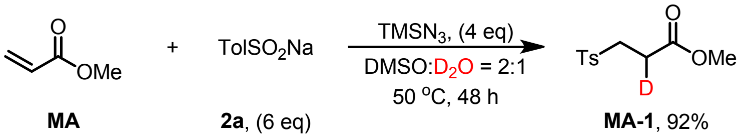

A Schlenk tube was placed compound **MA** (0.5 mmol), $TolSO_2Na$ (3.0 mmol), $TMSN_3$ (2.0 mmol), D_2O (0.7 mL), and DMSO (1.4 mL). Then the mixture was stirred at 50 °C for 48h. Upon completion of the reaction, saturated NH_4Cl (aq.) was added to quench the reaction and the mixture was extracted with CH_2Cl_2 (3 × 10 mL). The combined organic layers were washed with brine, dried over $MgSO_4$, filtered, and concentrated *in vacuo*. The resulting mixture was purified by silica gel column chromatography using (petroleum ether:ethyl acetate = 10:1) to afford deuterated product **MA-1** in 92% yield.[4]

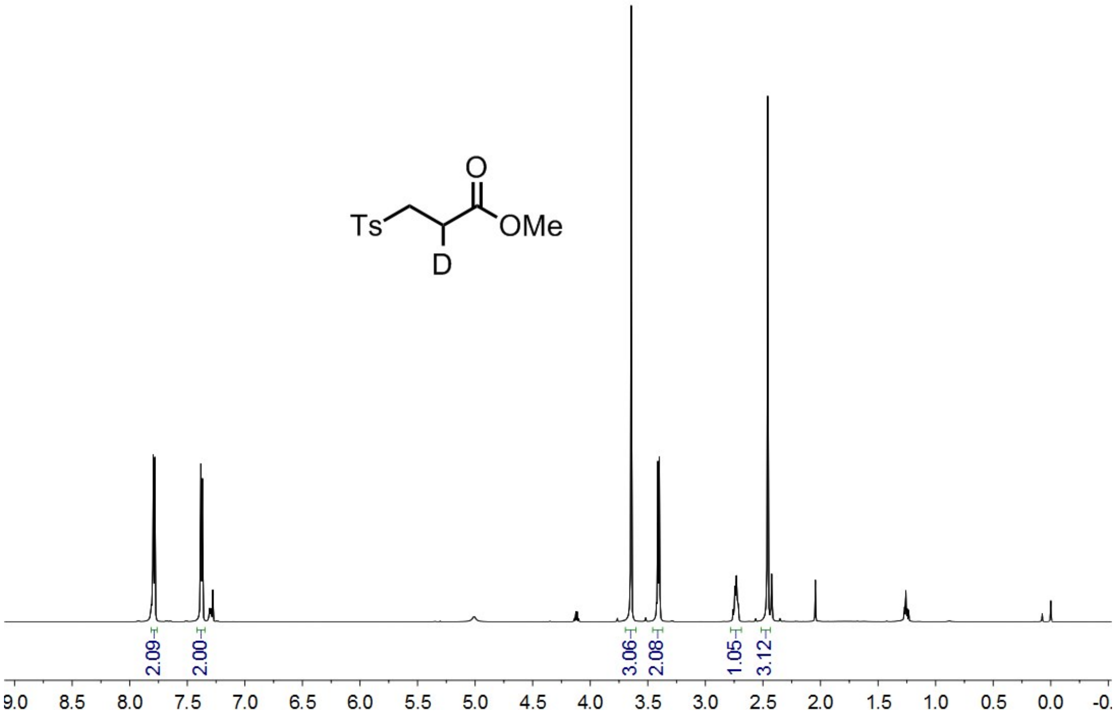

4.3 Research on the key intermediate

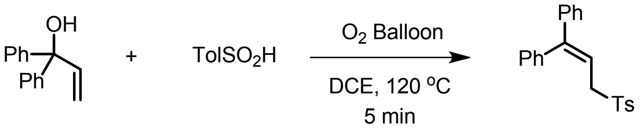

V-1 **V-2**

Allylic alcohol **V-1** (0.3 mmol) and *p*-toluene sulfonic acid (0.6 mmol) in dichloroethane (2 mL) were stirred at 120 °C with an O_2 balloon for 5 min. Upon completion of the reaction (as indicated by TLC analysis), the solvent was removed in vacuo and the residue was purified by flash column chromatography on silica gel with petroleum/ethyl acetate as the eluent to afford pure product **V-2** in 85% yield.[5]

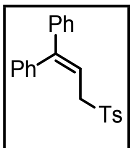

V-2

(3-Tosylprop-1-ene-1,1-diyl)dibenzene (V-2): Yellow solid; ^1H NMR (600 MHz, $CDCl_3$) δ 7.66 (d, *J* = 8.4 Hz, 2H), 7.29-7.22 (m, 8H), 7.17-7.15 (m, 2H), 6.70 (d, *J* = 8.4 Hz, 2H), 6.12 (t, *J* = 7.8 Hz, 1H), 3.90 (d, *J* = 7.8 Hz, 2H), 2.44 (s, 3H). ^{13}C NMR (150 MHz, $CDCl_3$) δ 149.60, 144.63, 140.59, 137.86, 135.81, 129.70, 129.27, 128.52, 128.33, 128.24, 127.77, 127.45, 114.28, 57.60, 21.66.

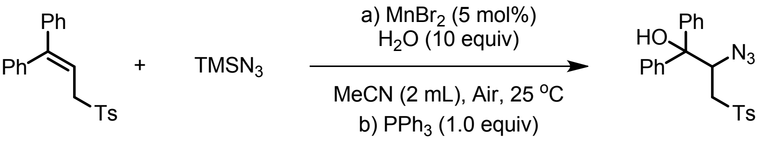

V-2 **M1**

Compound **V-2** (0.3 mmol), $TMSN_3$ (0.6 mmol), $MnBr_2$ (0.015 mmol) and H_2O (3 mmol) in

MeCN (2 mL) at room temperature under ambient air. Monitoring the reaction by TLC to confirm whether the **V-2** disappeared or not. Then PPh_3 (0.3 mmol) was added and the mixture was stirred for 10 min. finally, the mixture was purified through column chromatography to afford the pure product **M1** in 82% yield.[6]

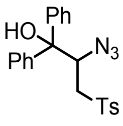

M1

2-Azido-1,1-diphenyl-3-tosylpropan-1-ol (M1): Yellow solid; ^{1}H NMR (600 MHz, $CDCl_3$) δ 7.73 (d, *J* = 8.4 Hz, 2H), 7.50 (d, *J* = 7.8 Hz, 2H), 7.38-7.34 (m, 6H), 7.29-7.26 (m, 3H), 7.23-7.22 (m, 1H), 4.97 (d, *J* = 9.0 Hz, 1H), 3.36-3.32 (m, 1H), 3.21 (d, *J* = 8.4 Hz, 1H), 2.77 (s, 1H), 2.45 (s, 3H). ^{13}C NMR (150 MHz, $CDCl_3$) δ 144.97, 143.82, 136.69, 130.04, 128.87, 128.83, 127.85, 127.83, 127.78, 125.47, 125.25, 80.58, 64.04, 56.92, 21.69. **HRMS** (ESI) m/z calculated for $C_{22}H_{21}N_3NaO_3S$ $[M+Na]^+$ 430.1212, found 430.1201

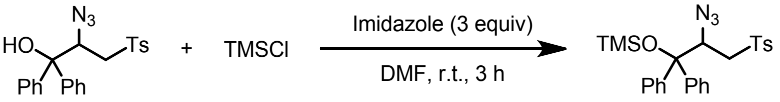

M1 (3 equiv) **M2**

Compound **M1** (0.3 mmol), TMSCl (0.9 mmol), and imidazole (0.9 mmol) in DMF (2 mL) was stirred at room temperature for 3 h. Upon completion of the reaction, saturated NH_4Cl (aq.) was added to quench the reaction and the mixture was extracted with CH_2Cl_2 (3 × 10 mL). The combined organic layers were washed with brine, dried over $MgSO_4$, filtered, and concentrated *in vacuo*. The resulting mixture was purified by silica gel column chromatography using (petroleum ether:ethyl acetate = 10:1) to afford deuterated product **M2** in 94% yield.

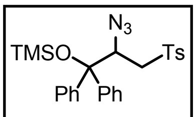

M2

(2-azido-1,1-diphenyl-3-tosylpropoxy)trimethylsilane (M2): White solid; ^{1}H NMR (600 MHz, $CDCl_3$) δ 7.68 (d, *J* = 7.8 Hz, 2H), 7.31-7.29 (m, 2H), 7.28 (d, *J* = 7.8 Hz, 2H), 7.26-7.23 (m, 6H), 7.19-7.16 (m, 2H), 4.75 (d, *J* = 9.6 Hz, 1H), 3.36 (d, *J* = 14.4 Hz, 1H), 2.65 (dd, *J* = 14.4, 9.6 Hz, 1H), 2.37 (s, 3H), -0.26 (s, 9H). ^{13}C NMR (150 MHz, $CDCl_3$) δ 143.89, 140.60, 140.07, 135.54, 128.99, 127.36, 127.27, 127.20, 127.14, 127.04, 127.02, 127.00, 82.10, 62.26, 20.62, 0.59. **HRMS** (ESI) m/z calculated for $C_{25}H_{29}N_3NaO_3SSi$ $[M+Na]^+$ 502.1603, found 502.1597.

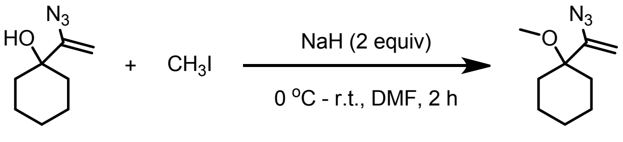

1ag 2 equiv **M3**

Compound **1ag** (0.3 mmol), and NaH (0.6 mmol) in DMF (2 mL) was stirred at 0 °C. After 0.5 h, CH_3I (0.6 mmol) was added to the reaction system. Then the reaction was stirred at room temperature for 2 h. Upon completion of the reaction, saturated NH_4Cl (aq.) was added to quench the reaction and the mixture was extracted with CH_2Cl_2 (3 × 10 mL). The combined organic layers were washed with brine, dried over $MgSO_4$, filtered, and concentrated *in vacuo*. The resulting mixture was purified by silica gel column chromatography using (petroleum ether:ethyl acetate = 15:1) to afford deuterated product **M3** in 96% yield.

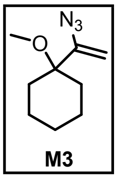

1-(1-azidovinyl)-1-methoxycyclohexane (M3): Yellow oil; ^{1}H NMR (600 MHz, $CDCl_3$) δ 4.94 (d, *J* = 1.2 Hz, 1H), 4.87 (d, *J* = 1.2 Hz, 1H), 3.11 (s, 3H), 1.86-1.84 (m, 2H), 1.60-1.52 (m, 3H), 1.50-1.46 (m, 4H), 1.25-1.20 (m, 1H). ^{13}C NMR (150 MHz, $CDCl_3$) δ 149.74, 99.42, 76.57, 49.55, 32.68, 25.57, 21.51. **HRMS** (ESI) m/z calculated for $C_9H_{15}N_3NaO$ $[M+Na]^+$ 204.1108, found 204.1113.

4.4 The detection of TMSOH and $(TMS)_2O$ by gas chromatography-mass spectrometer (GC-MS)

The Si-containing species in the reaction mixture were identified as TMSOH and $(TMS)_2O$ by GC-MS.

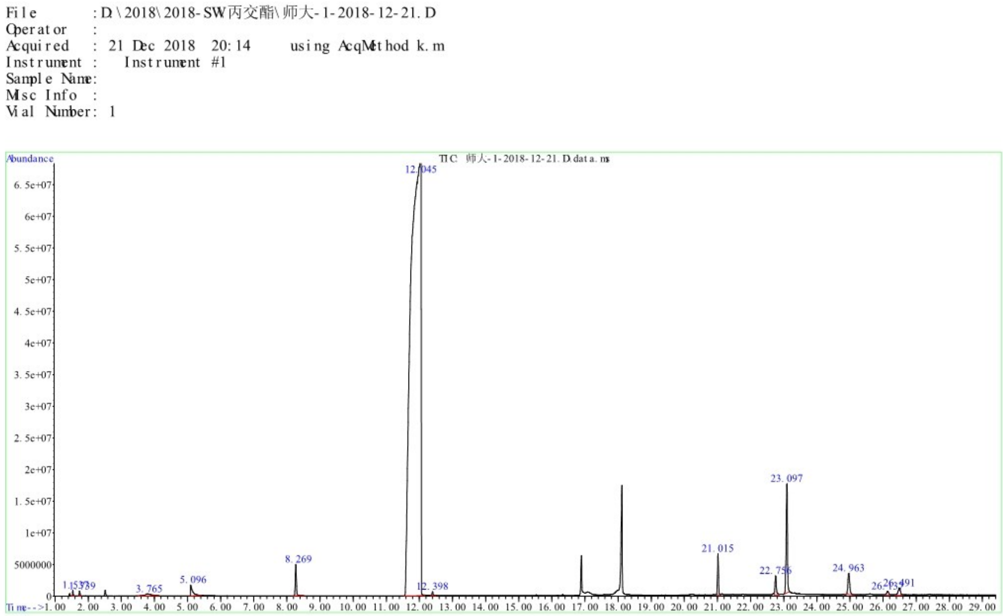

PDF 文件使用 "pdfFactory Pro" 试用版本创建 www.fineprint.com.cn

** Search Report Page 1 of 1 **

Unknown: Scan 264 (1.536 min): -1-2018-12-21.D\data.ms
Compound in Library Factor = 256

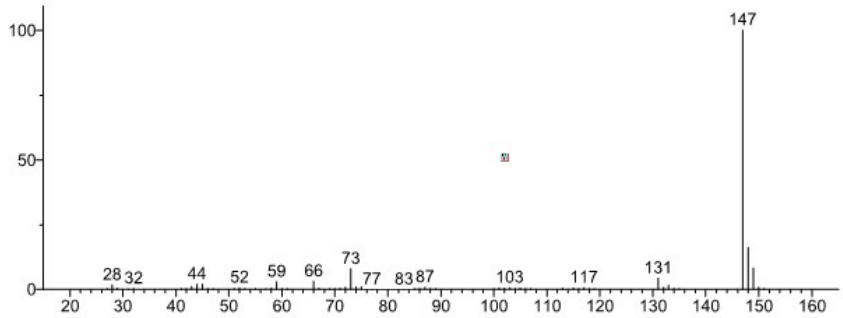

Hit 1 : Disiloxane, hexamethyl-
C6H18OSi2; MF: 924; RMF: 925; Prob 84.1%; CAS: 107-46-0; Lib: replib; ID: 19826.

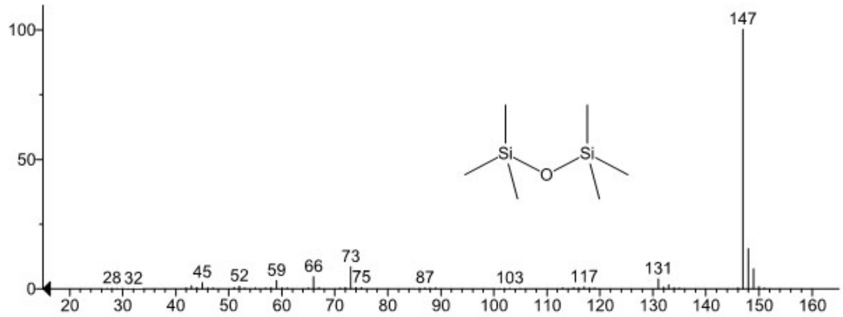

Hit 2 : Disiloxane, hexamethyl-
C6H18OSi2; MF: 913; RMF: 916; Prob 84.1%; CAS: 107-46-0; Lib: replib; ID: 19825.

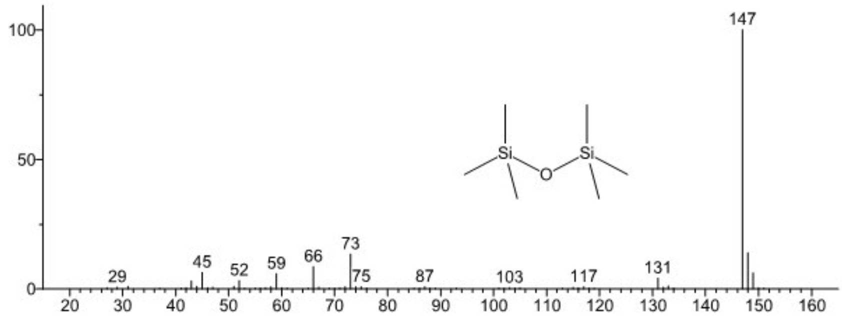

PDF 文件使用 "pdfFactory Pro" 试用版本创建 www.fineprint.com.cn

** Search Report Page 1 of 1 **

Unknown: Scan 670 (3.773 min): -1-2018-12-21.D\data.ms
Compound in Library Factor = -343

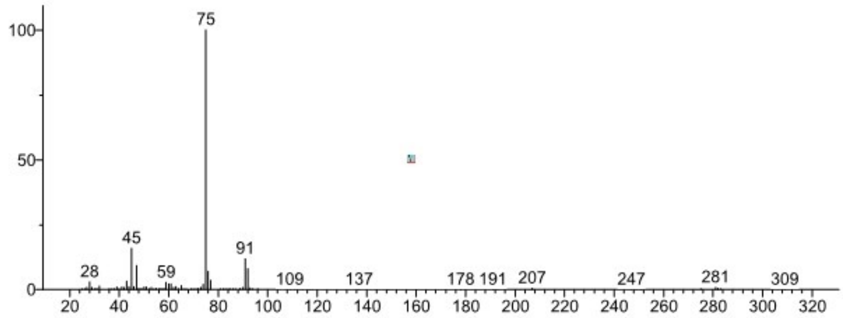

Hit 1 : Silanol, trimethyl-
C3H10OSi; MF: 761; RMF: 793; Prob 62.1%; CAS: 1066-40-6; Lib: mainlib; ID: 38640.

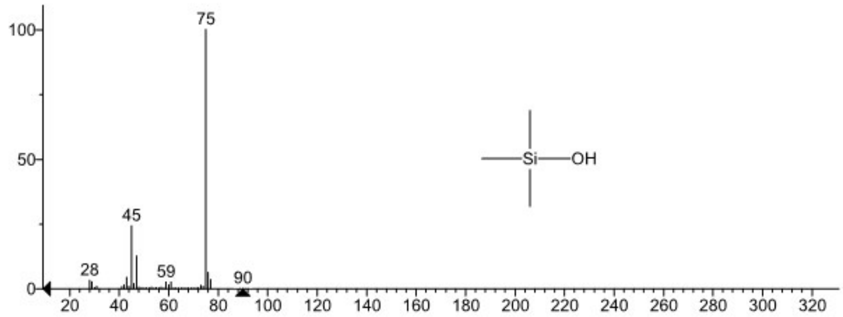

Hit 2 : Silanol, trimethyl-
C3H10OSi; MF: 737; RMF: 888; Prob 62.1%; CAS: 1066-40-6; Lib: replib; ID: 9172.

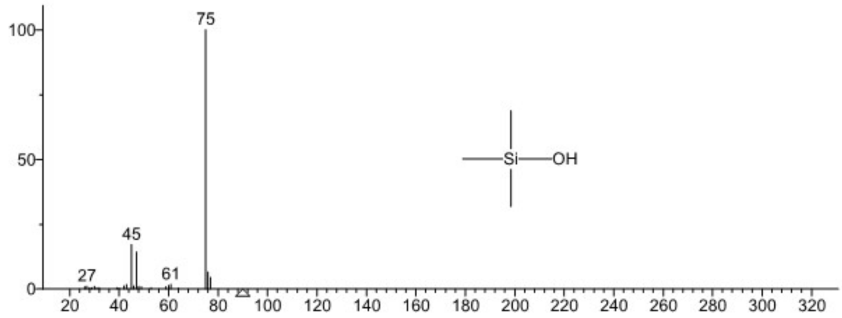

PDF 文件使用 "pdfFactory Pro" 试用版本创建 www.fineprint.com.cn

5. X-ray Structure of 3an and 3ba

Table S2. Crystal Structure of 1,2-diphenyl-3-tosylpropan-1-one 3an (CCDC No. 1897875)

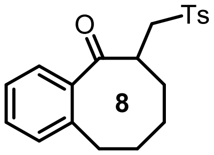

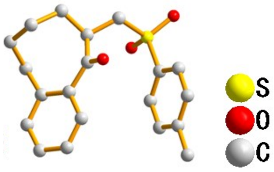

Empirical formula	C20 H22 O3 S
Temperature	293 K
Wavelength	0.71073 Å
Unit cell dimensions	a = 9.2575(7) Å<br>b = 9.6845(7) Å<br>c =10.1145(8)Å<br>alpha = 79.903(6)deg.<br>beta = 84.497(7) deg.<br>gamma = 80.659(6) deg.
Volume	878.80(12)Å ^{3}
Z	2
Calculated density	1.294 g/m^{3}
Absorption coefficient	0.199 mm^{-1}
F(000)	364.0
Crystal size	0.15 × 0.12 × 0.1 mm^{3}
Theta range for data collection	7.622 to 58.388 deg.
Reflections collected / unique	6448 / 3958 [R(int) = 0.0181]
Data / restraints / parameters	3958 / 0 / 218
Goodness-of-fit on F^{2}	1.017
Final R indices [I>2sigma(I)]	R_1 = 0.0503, wR_2 = 0.1134
Rindices (all data)	R_1 = 0.0731, wR_2 = 0.1308

Table S3. Crystal Structure of 1,2-diphenyl-3-tosylpropan-1-one 3ba (CCDC No. 1897779)

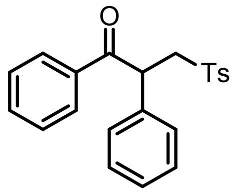

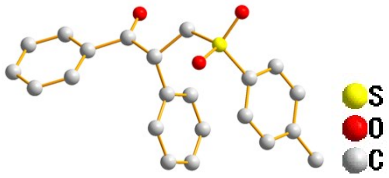

Empirical formula	C22H20O3S
Temperature	293 K
Wavelength	0.71073 Å
Unit cell dimensions	a = 19.855(3) Å<br>b = 5.7928(7) Å<br>c =16.427(2) Å<br>alpha = 90.0 deg.<br>beta = 105.359(13) deg.<br>gamma = 90.0 deg.
Volume	1821.9(4) Å ^{3}
Z	4
Calculated density	1.329 g/m3
Absorption coefficient	0.196 mm-1
F(000)	768.0
Crystal size	0.15 × 0.12 × 0.1 mm^3
Theta range for data collection	7.06 to 58.402 deg.
Reflections collected / unique	7635 / 4207 [R(int) = 0.0293]
Data / restraints / parameters	4207 / 0 / 236
Goodness-of-fit on F^2	1.018
Final R indices [I>2sigma(I)]	R_1 = 0.0495, wR_2 = 0.0986
Rindices (all data)	R_1 = 0.0877, wR_2 = 0.1149

6. NMR Spectra of the Products

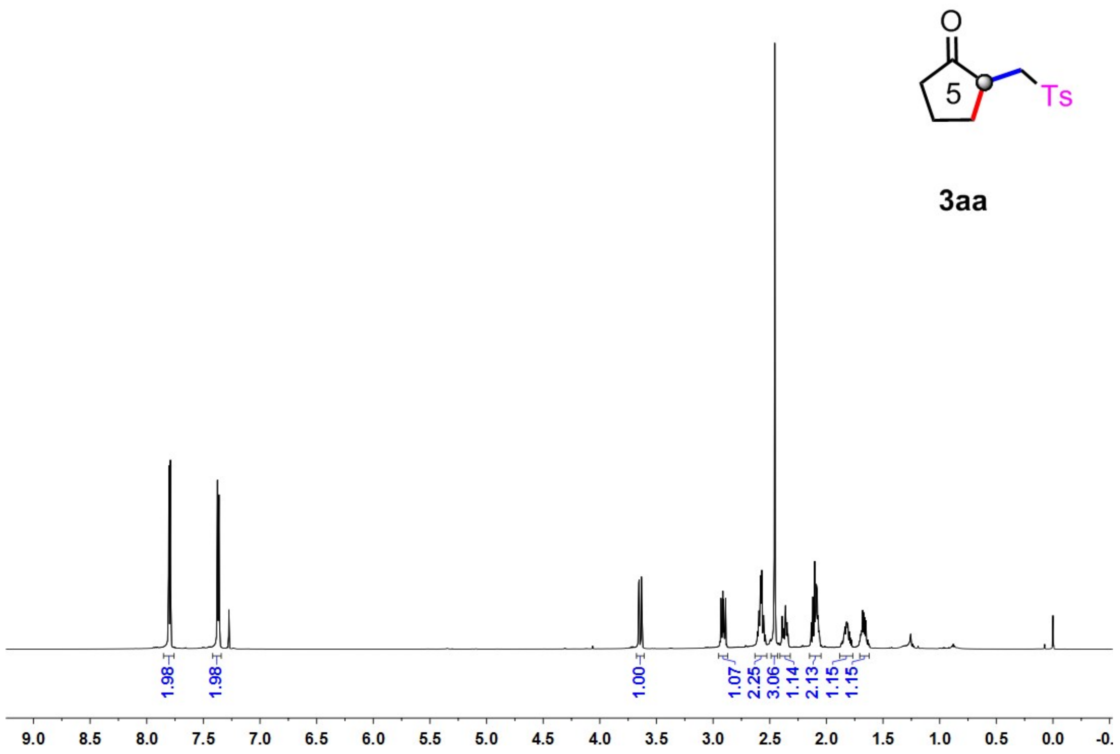

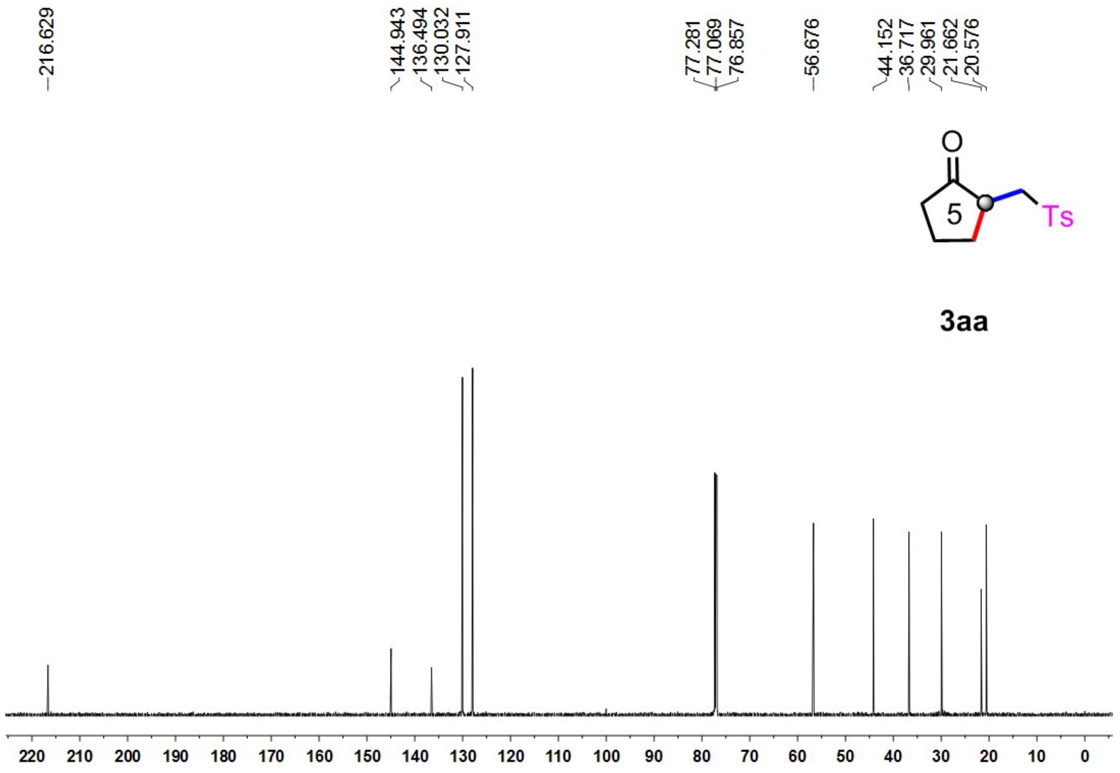

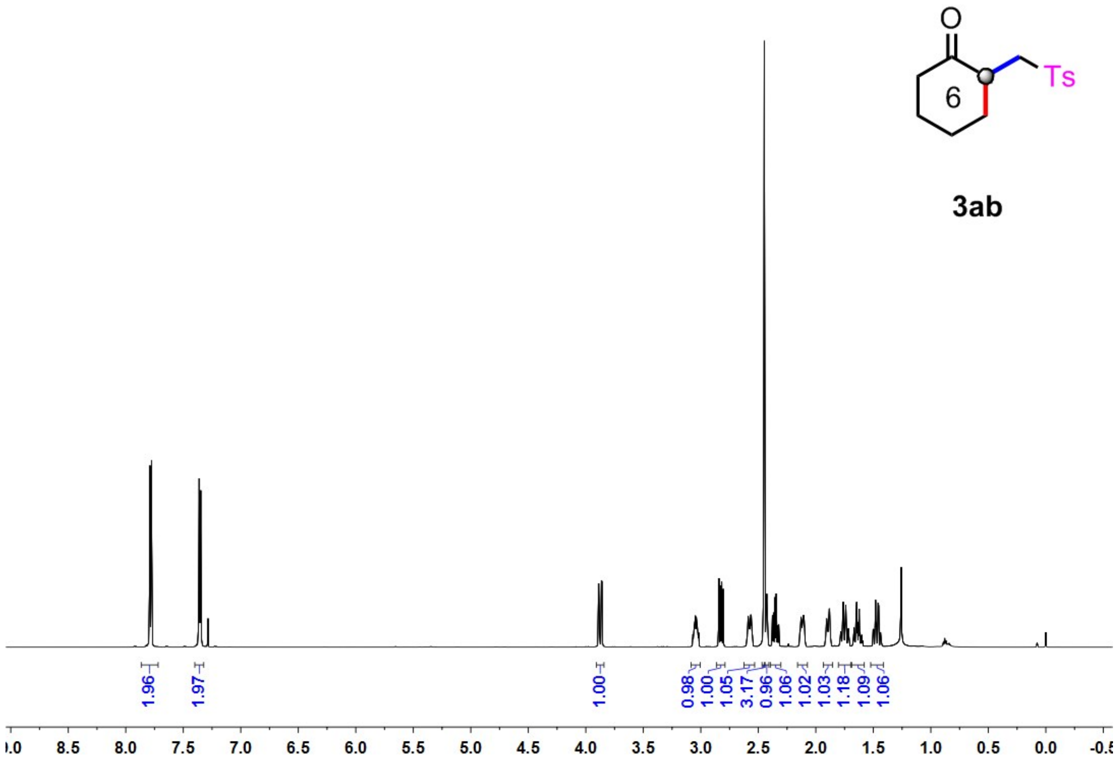
O
6
Ts
3ab
1.96
1.97
1.00
0.98
1.00
1.05
3.17
0.96
1.06
1.02
1.03
1.18
1.09
1.06
8.5
8.0
7.5
7.0
6.5
6.0
5.5
5.0
4.5
4.0
3.5
3.0
2.5
2.0
1.5
1.0
0.5
0.0

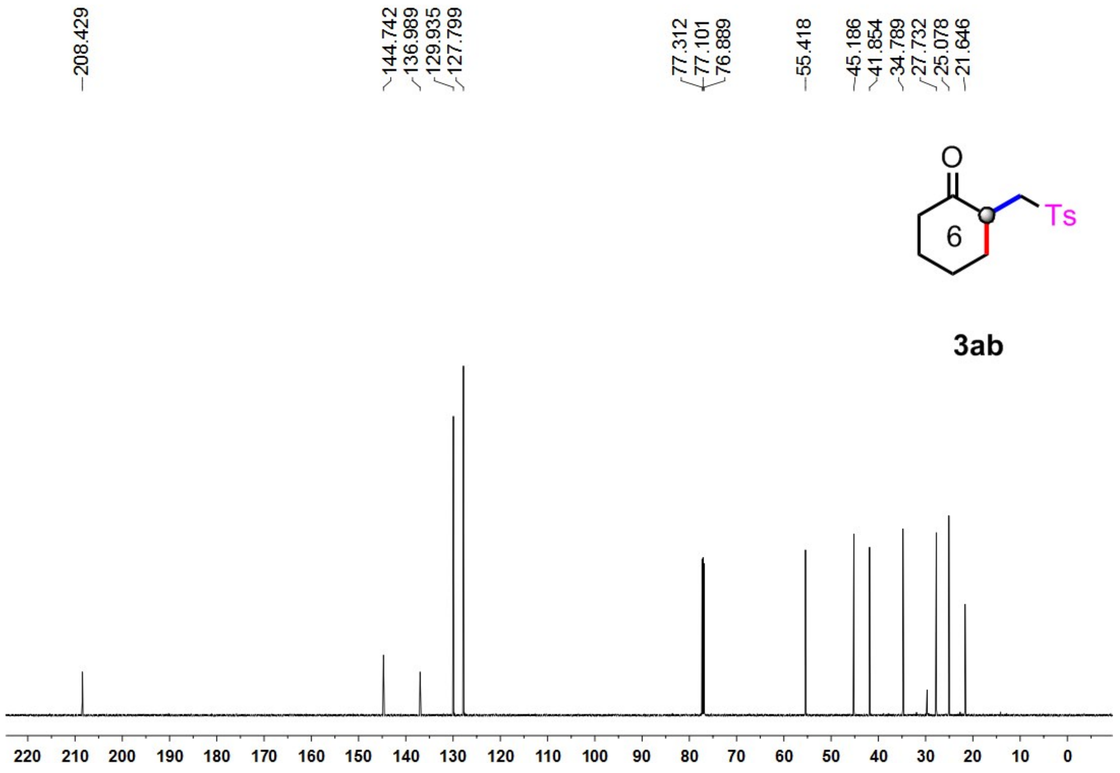
208.429
144.742
136.989
129.935
127.799
77.312
77.101
76.889
55.418
45.186
41.854
34.789
27.732
25.078
21.646
O
6
Ts
3ab
220
210
200
190
180
170
160
150
140
130
120
110
100
90
80
70
60
50
40
30
20
10
0

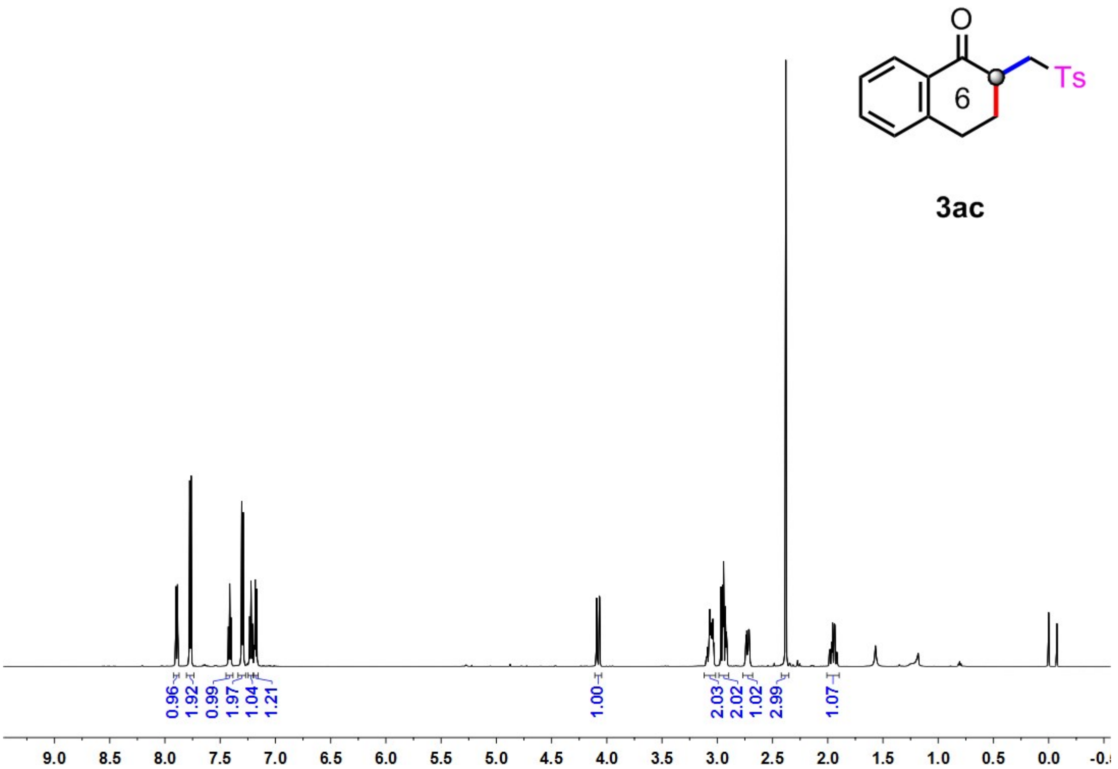
O
6
Ts
3ac
0.96
1.92
0.99
1.97
1.04
1.21
1.00
2.03
2.02
1.02
2.99
1.07
9.0 8.5 8.0 7.5 7.0 6.5 6.0 5.5 5.0 4.5 4.0 3.5 3.0 2.5 2.0 1.5 1.0 0.5 0.0

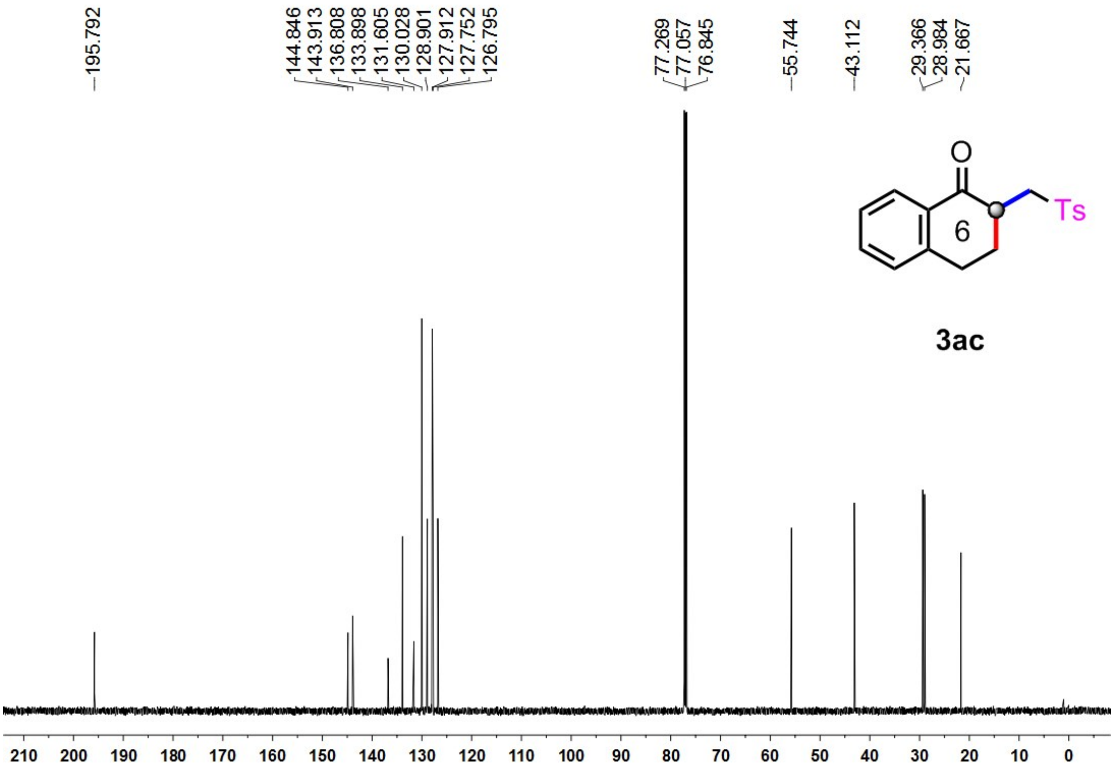
195.792
144.846
143.913
136.808
133.898
131.605
130.028
128.901
127.912
127.752
126.795
77.269
77.057
76.845
55.744
43.112
29.366
28.984
21.667
O
6
Ts
3ac
210 200 190 180 170 160 150 140 130 120 110 100 90 80 70 60 50 40 30 20 10 0

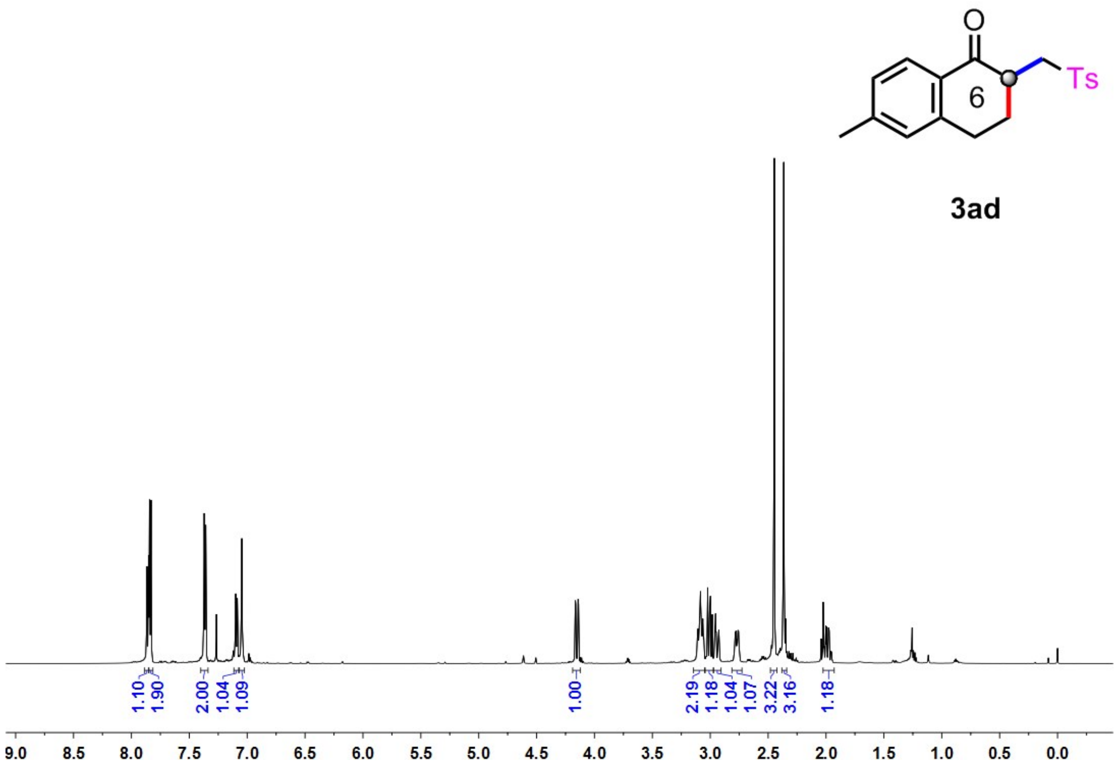
6
Ts
1.10
1.90
2.00
1.04
1.09
1.00
2.19
1.18
1.04
1.07
3.22
3.16
1.18
9.0 8.5 8.0 7.5 7.0 6.5 6.0 5.5 5.0 4.5 4.0 3.5 3.0 2.5 2.0 1.5 1.0 0.5 0.0

3ad

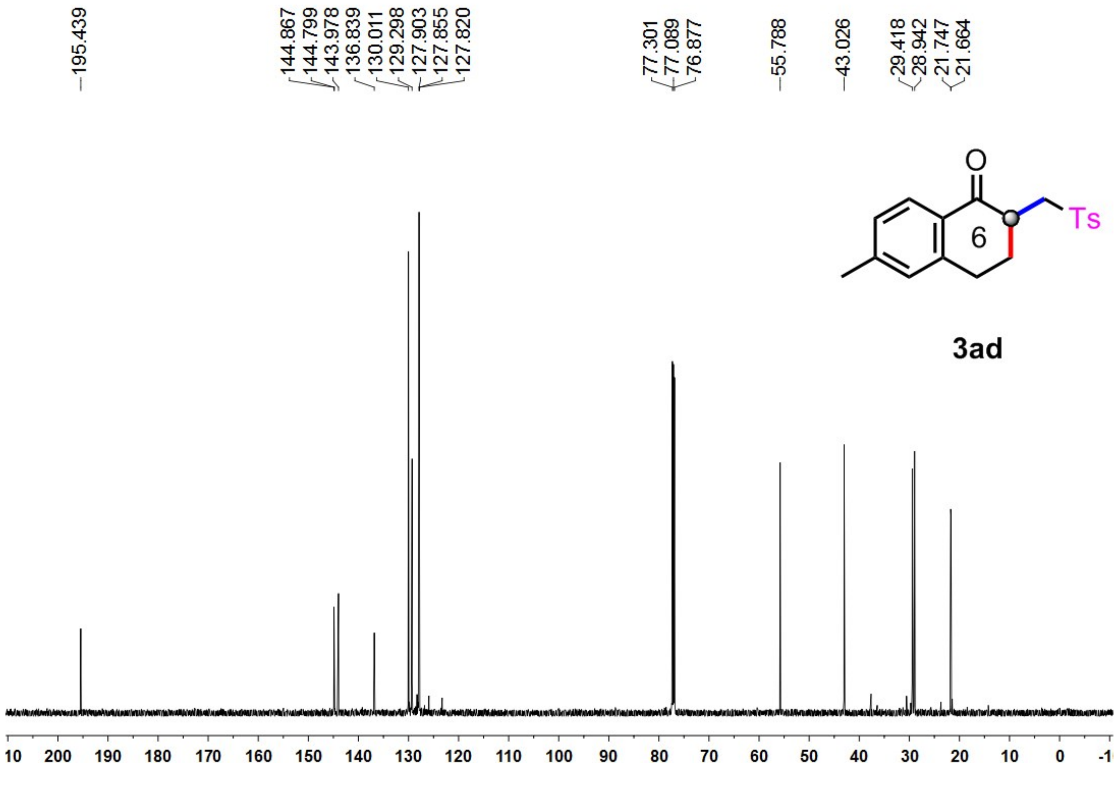
195.439
144.867
144.799
143.978
136.839
130.011
129.298
127.903
127.855
127.820
77.301
77.089
76.877
55.788
43.026
29.418
28.942
21.747
21.664
6
Ts
200 190 180 170 160 150 140 130 120 110 100 90 80 70 60 50 40 30 20 10 0

3ad

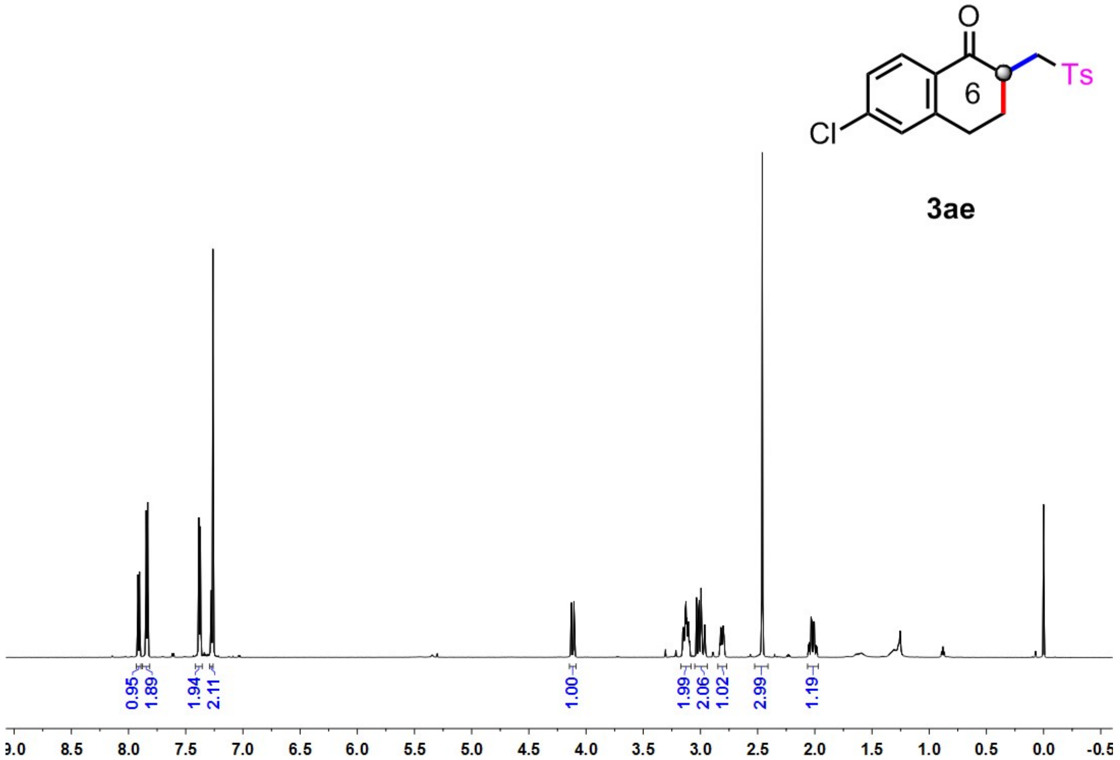
3ae
6
Ts
Cl
O
0.95
1.89
1.94
2.11
1.00
1.99
2.06
1.02
2.99
1.19

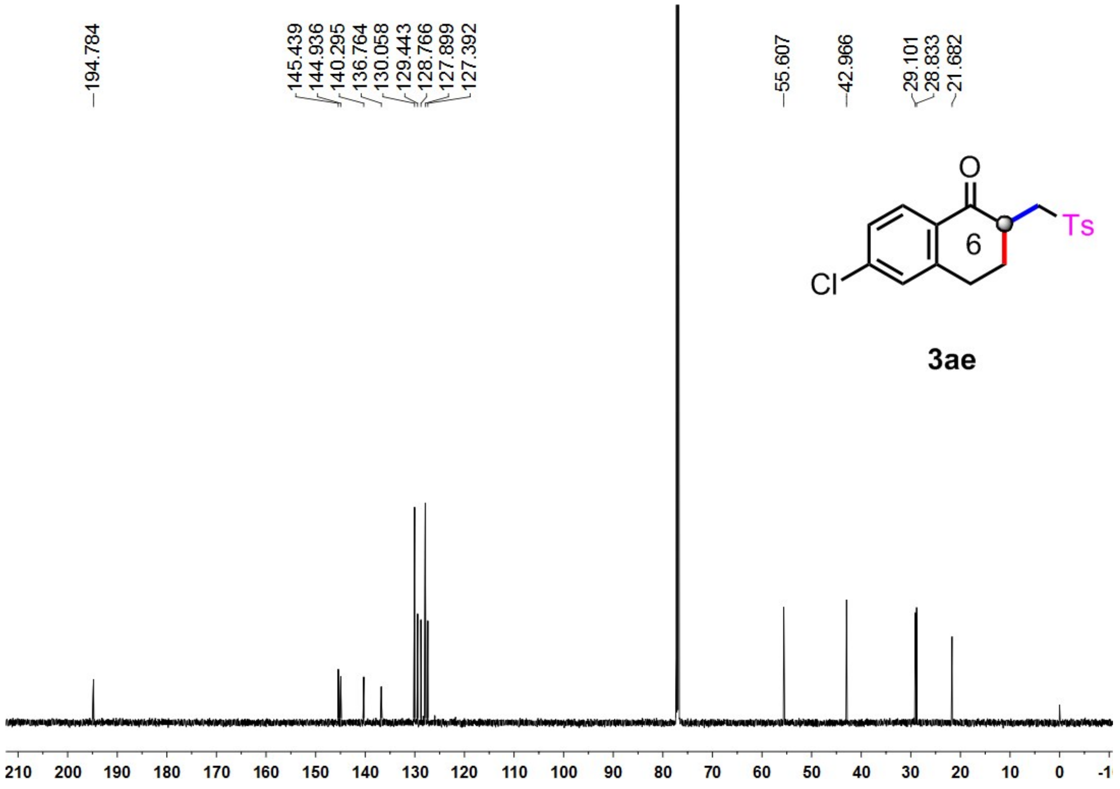
194.784
145.439
144.936
140.295
136.764
130.058
129.443
128.766
127.899
127.392
55.607
42.966
29.101
28.833
21.682
3ae
6
Ts
Cl
O

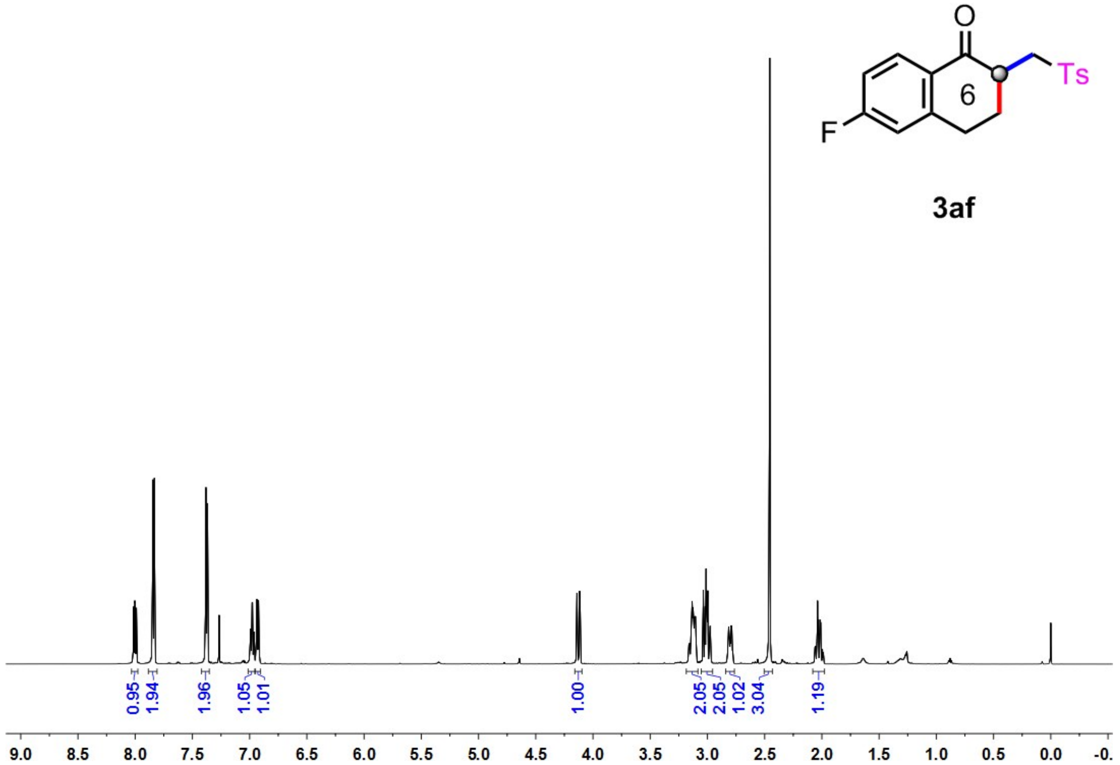
O
6
Ts
F
3af
0.95
1.94
1.96
1.05
1.01
1.00
2.05
2.05
1.02
3.04
1.19
9.0 8.5 8.0 7.5 7.0 6.5 6.0 5.5 5.0 4.5 4.0 3.5 3.0 2.5 2.0 1.5 1.0 0.5 0.0

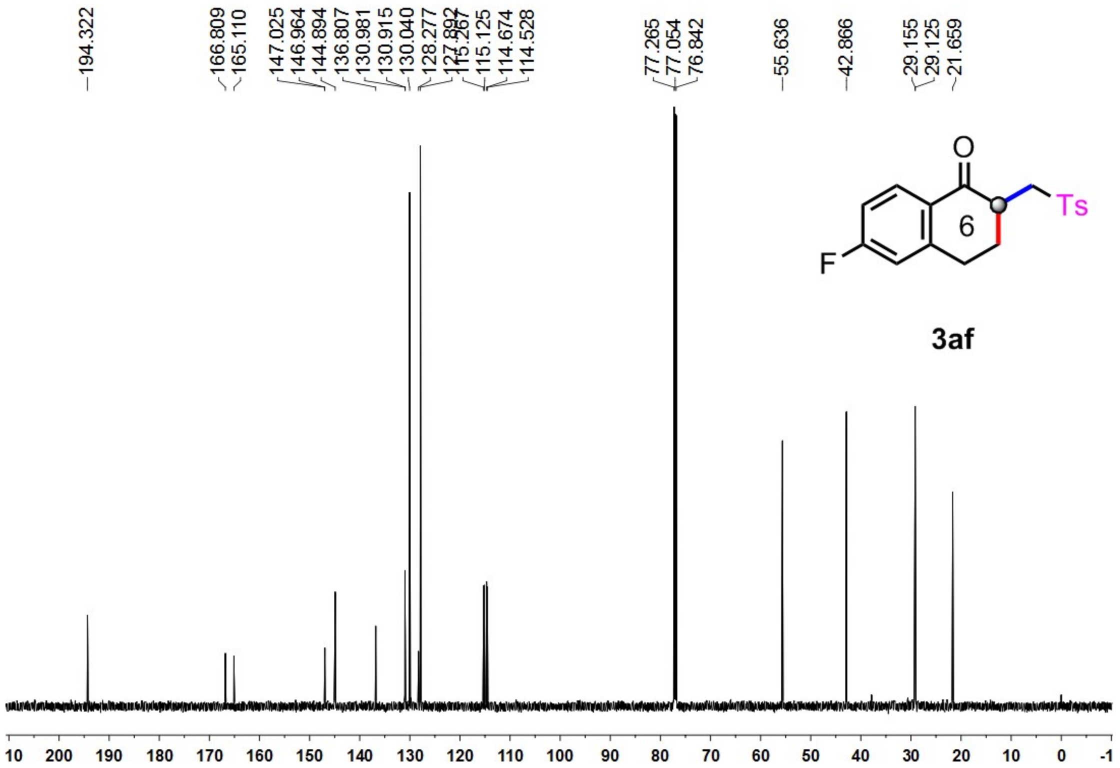
194.322
166.809
165.110
147.025
146.964
144.894
136.807
130.981
130.915
130.040
128.277
115.125
114.674
114.528
77.265
77.054
76.842
55.636
42.866
29.155
29.125
21.659
O
6
Ts
F
3af
200 190 180 170 160 150 140 130 120 110 100 90 80 70 60 50 40 30 20 10 0

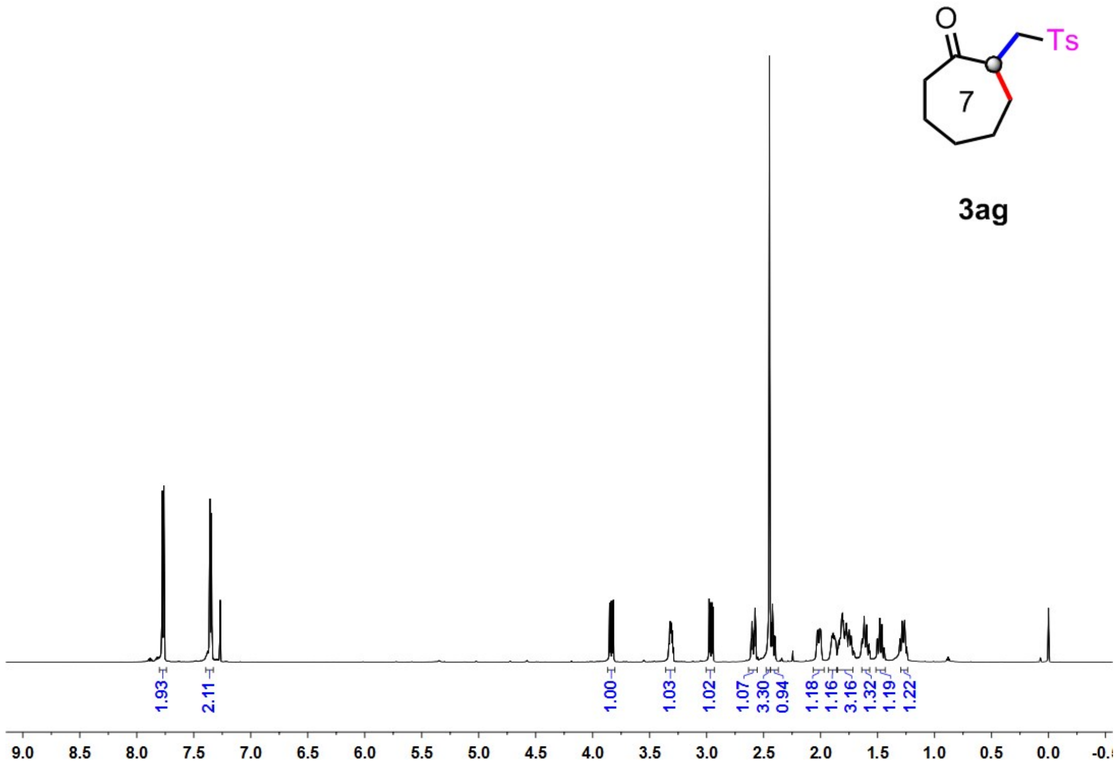
3ag
Ts
7
O
1.93
2.11
1.00
1.03
1.02
1.07
3.30
0.94
1.18
1.16
3.16
1.32
1.19
1.22
9.0 8.5 8.0 7.5 7.0 6.5 6.0 5.5 5.0 4.5 4.0 3.5 3.0 2.5 2.0 1.5 1.0 0.5 0.0

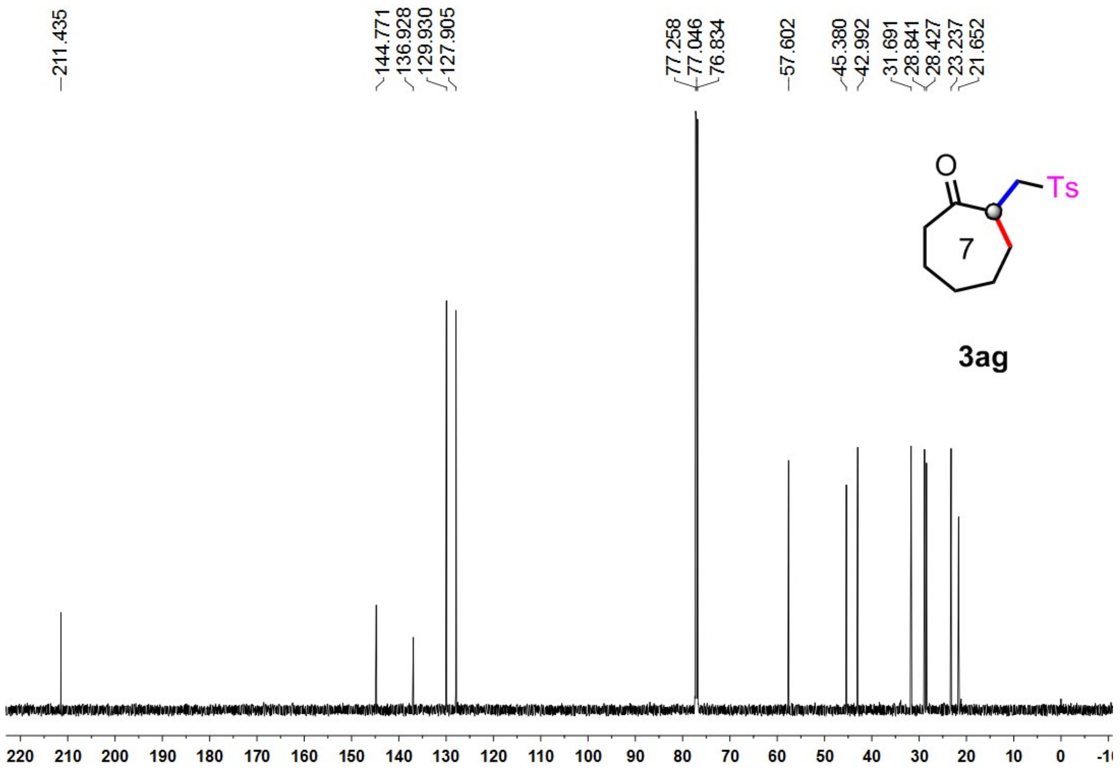
211.435
144.771
136.928
129.930
127.905
77.258
77.046
76.834
57.602
45.380
42.992
31.691
28.841
28.427
23.237
21.652
3ag
Ts
7
O
220 210 200 190 180 170 160 150 140 130 120 110 100 90 80 70 60 50 40 30 20 10 0

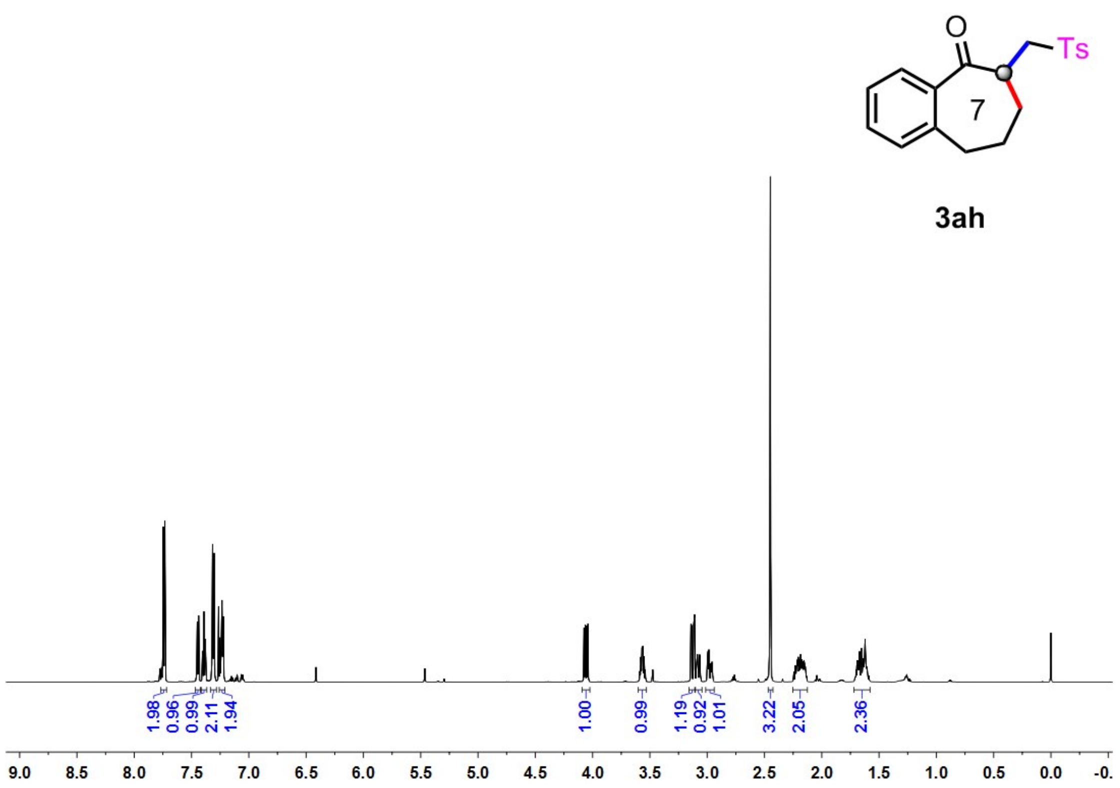
O
Ts
7
3ah
1.98
0.96
0.99
2.11
1.94
1.00
0.99
1.19
0.92
1.01
3.22
2.05
2.36
9.0
8.5
8.0
7.5
7.0
6.5
6.0
5.5
5.0
4.5
4.0
3.5
3.0
2.5
2.0
1.5
1.0
0.5
0.0

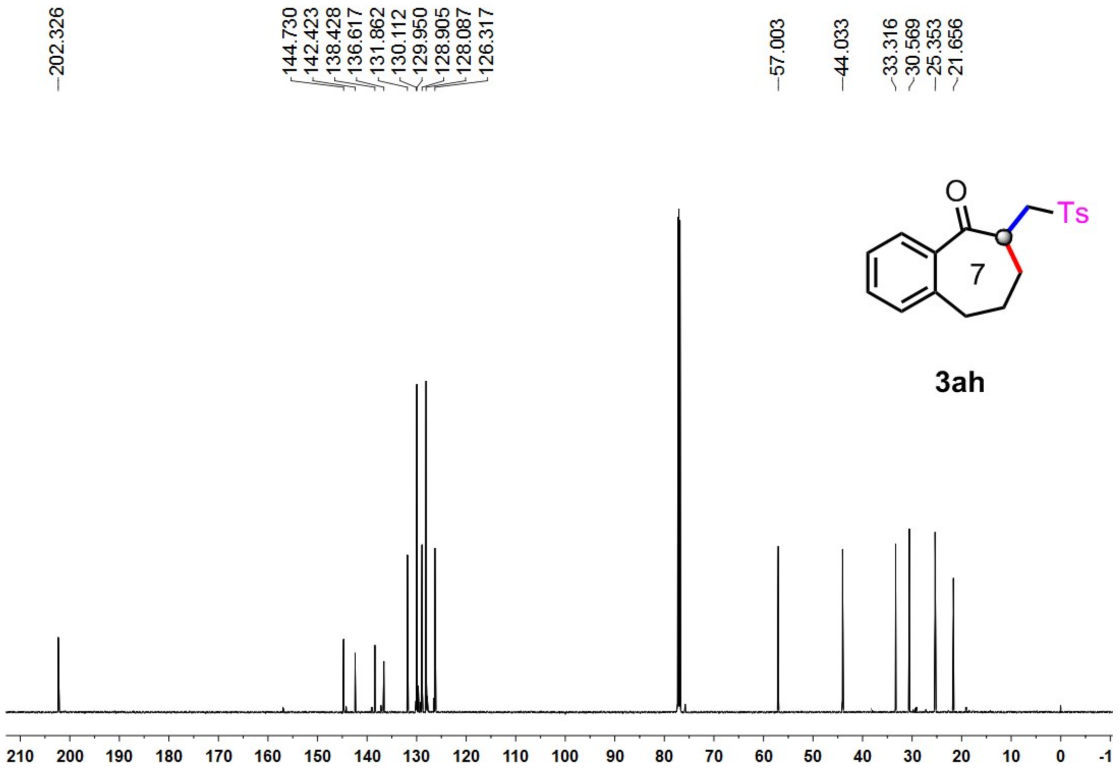
202.326
144.730
142.423
138.428
136.617
131.862
130.112
129.950
128.905
128.087
126.317
57.003
44.033
33.316
30.569
25.353
21.656
O
Ts
7
3ah
210
200
190
180
170
160
150
140
130
120
110
100
90
80
70
60
50
40
30
20
10
0
-1

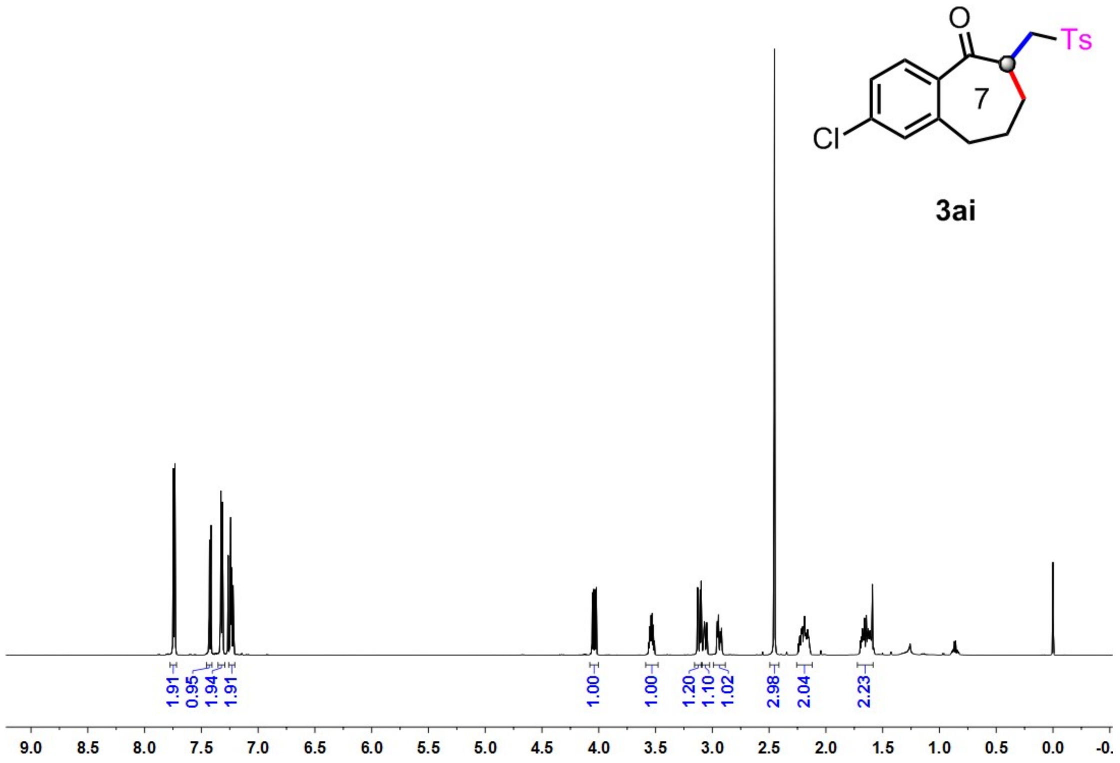
O
Ts
7
Cl
3ai
1.91
0.95
1.94
1.91
1.00
1.00
1.20
1.10
1.02
2.98
2.04
2.23
9.0
8.5
8.0
7.5
7.0
6.5
6.0
5.5
5.0
4.5
4.0
3.5
3.0
2.5
2.0
1.5
1.0
0.5
0.0

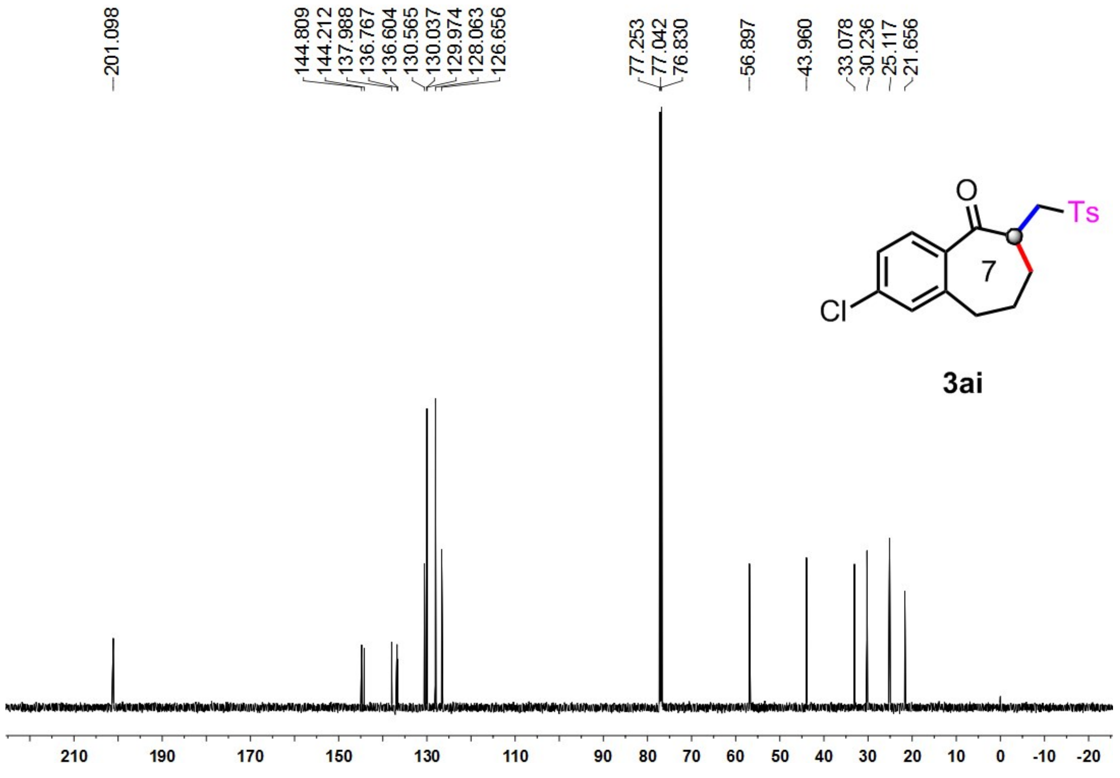
201.098
144.809
144.212
137.988
136.767
136.604
130.565
130.037
129.974
128.063
126.656
77.253
77.042
76.830
56.897
43.960
33.078
30.236
25.117
21.656
O
Ts
7
Cl
3ai
210
190
170
150
130
110
90
80
70
60
50
40
30
20
10
0
-10
-20

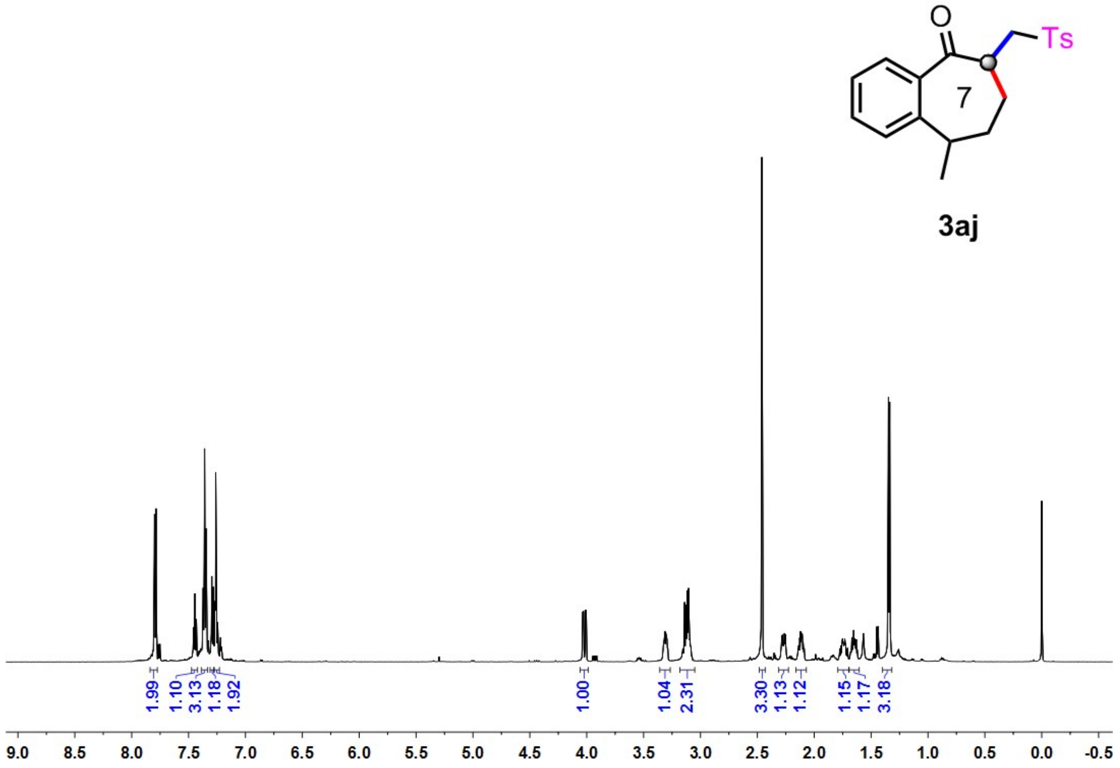
O
Ts
7
3aj
1.99
1.10
3.13
1.18
1.92
1.00
1.04
2.31
3.30
1.13
1.12
1.15
1.17
3.18
9.0 8.5 8.0 7.5 7.0 6.5 6.0 5.5 5.0 4.5 4.0 3.5 3.0 2.5 2.0 1.5 1.0 0.5 0.0 -0.5

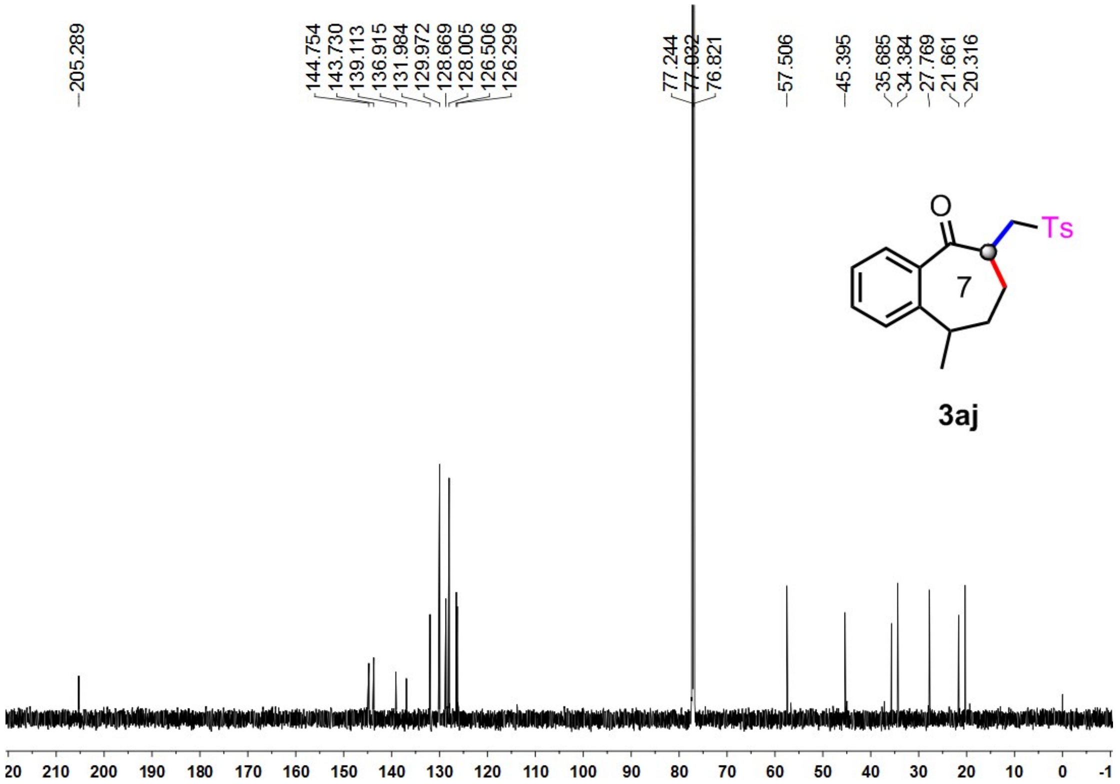
205.289
144.754
143.730
139.113
136.915
131.984
129.972
128.669
128.005
126.506
126.299
77.244
77.032
76.821
57.506
45.395
35.685
34.384
27.769
21.661
20.316
O
Ts
7
3aj
20 210 200 190 180 170 160 150 140 130 120 110 100 90 80 70 60 50 40 30 20 10 0 -1

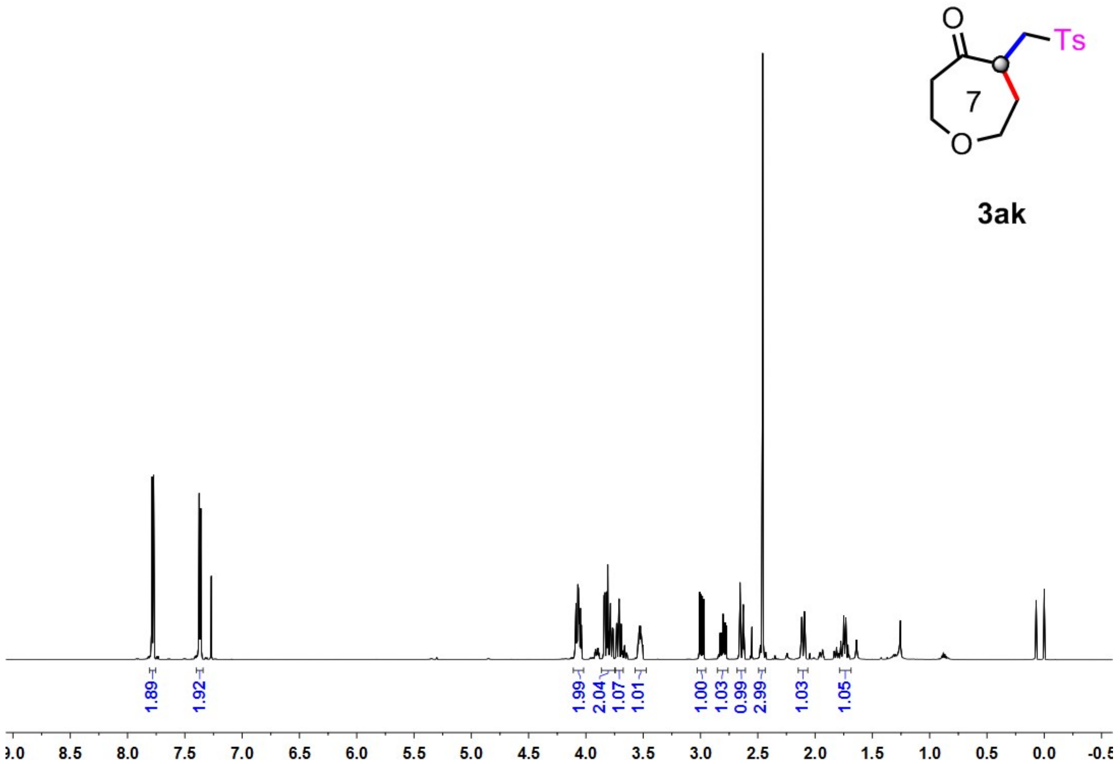
O
Ts
7
O
3ak
1.89
1.92
1.99
2.04
1.07
1.01
1.00
1.03
0.99
2.99
1.03
1.05
9.0 8.5 8.0 7.5 7.0 6.5 6.0 5.5 5.0 4.5 4.0 3.5 3.0 2.5 2.0 1.5 1.0 0.5 0.0 -0.5

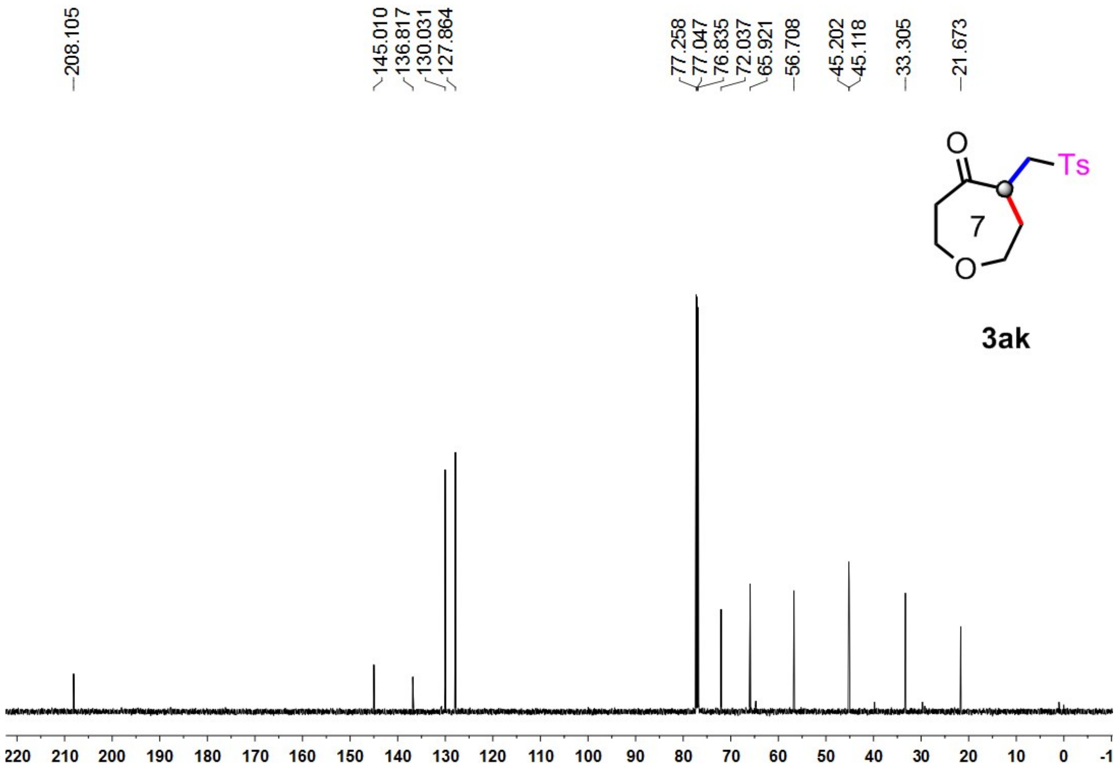
208.105
145.010
136.817
130.031
127.864
77.258
77.047
76.835
72.037
65.921
56.708
45.202
45.118
33.305
21.673
O
Ts
7
O
3ak
220 210 200 190 180 170 160 150 140 130 120 110 100 90 80 70 60 50 40 30 20 10 0 -1

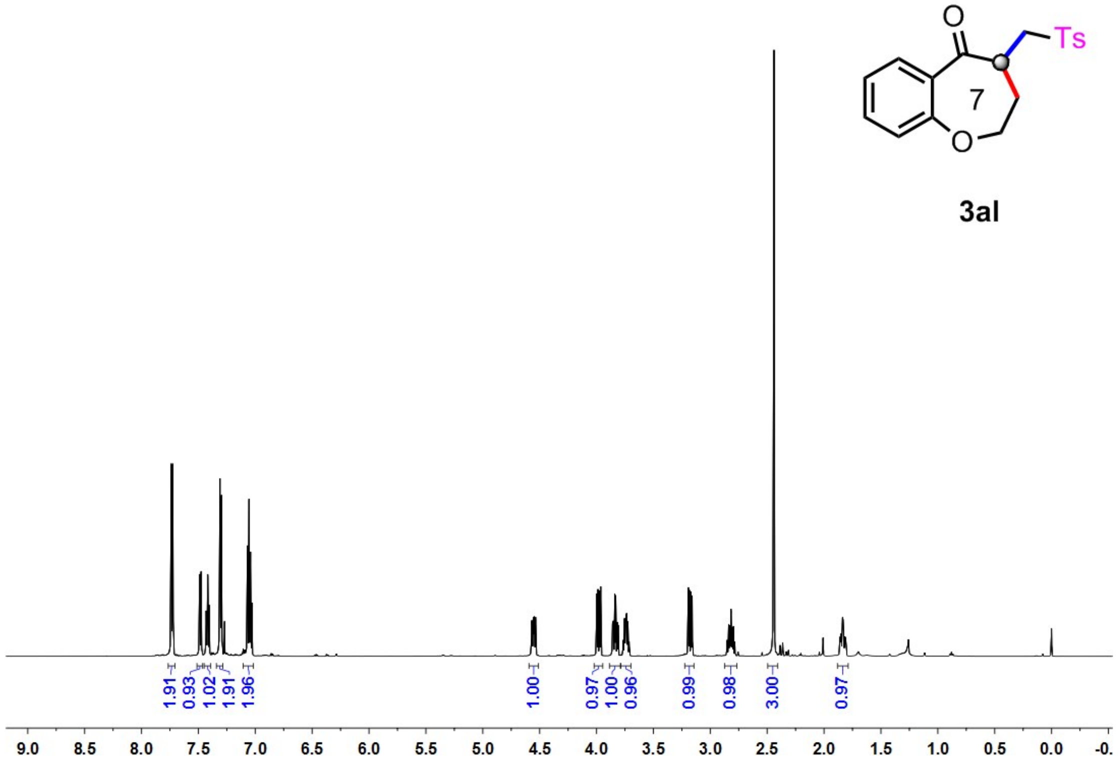

Ts
7
O
3al
1.91
0.93
1.02
1.91
1.96
1.00
0.97
1.00
0.96
0.99
0.98
3.00
0.97
9.0 8.5 8.0 7.5 7.0 6.5 6.0 5.5 5.0 4.5 4.0 3.5 3.0 2.5 2.0 1.5 1.0 0.5 0.0 -0.

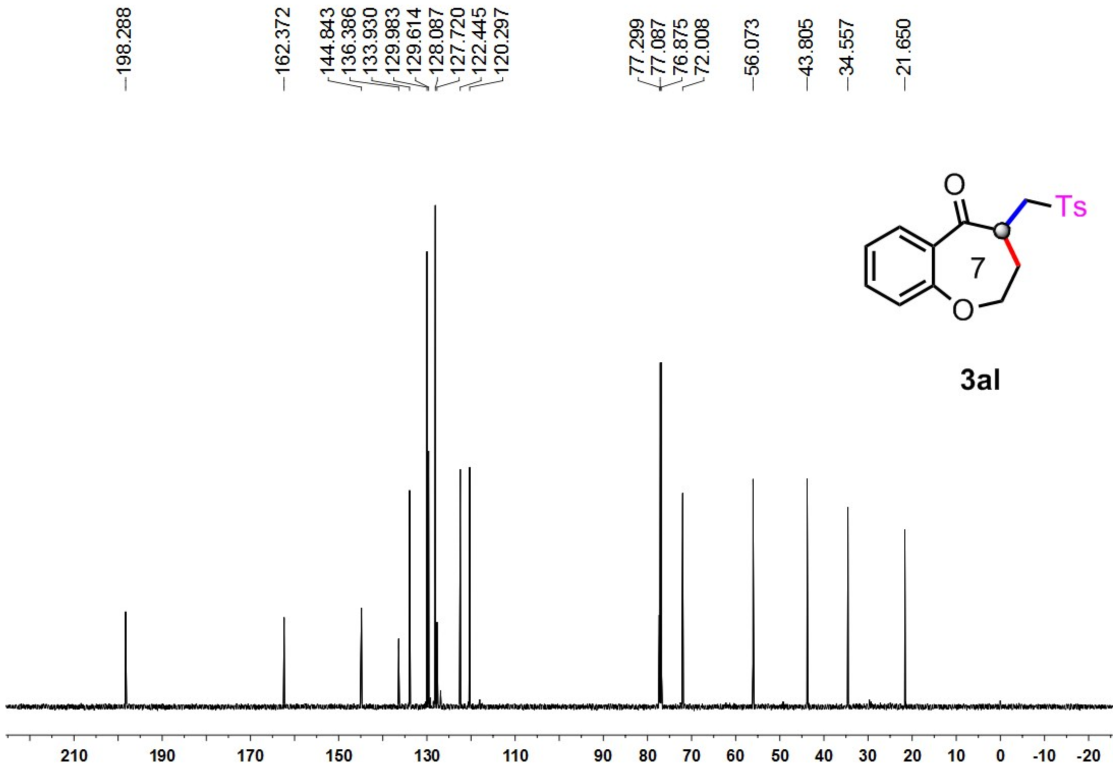

198.288
162.372
144.843
136.386
133.930
129.983
129.614
128.087
127.720
122.445
120.297
77.299
77.087
76.875
72.008
56.073
43.805
34.557
21.650
Ts
7
O
3al
210 190 170 150 130 110 90 80 70 60 50 40 30 20 10 0 -10 -20

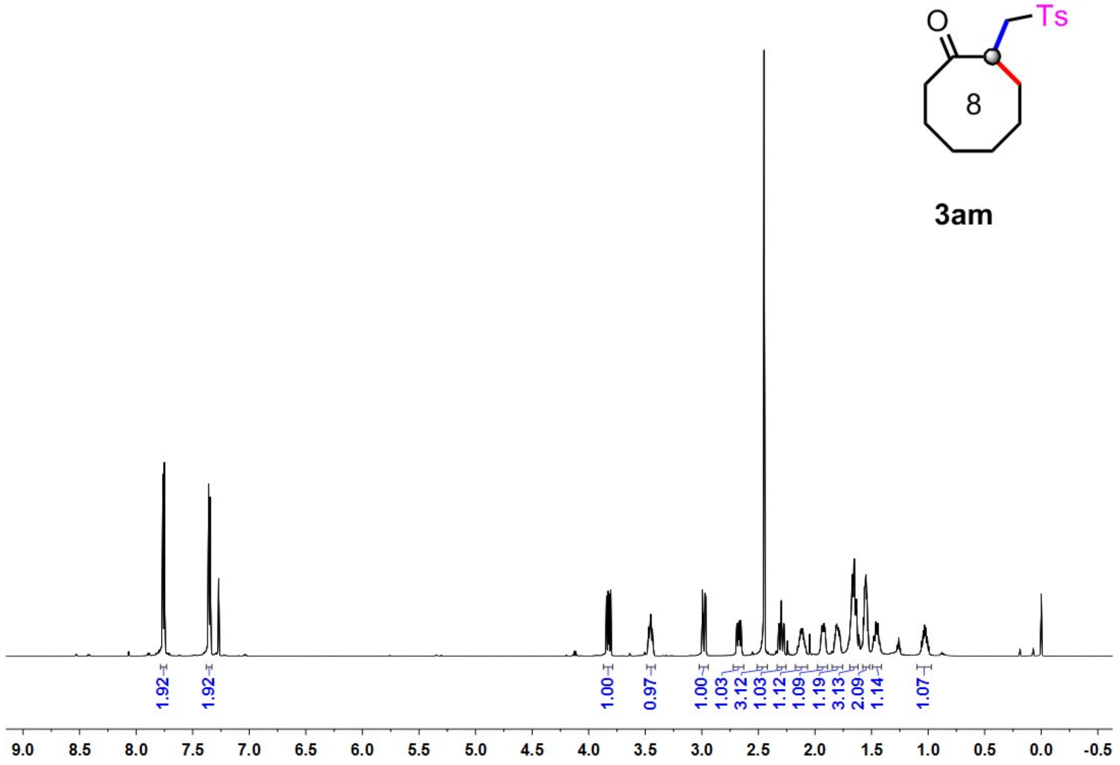
3am
8
O
Ts
1.92
1.92
1.00
0.97
1.00
1.03
3.12
1.03
1.12
1.09
1.19
3.13
2.09
1.14
1.07
9.0 8.5 8.0 7.5 7.0 6.5 6.0 5.5 5.0 4.5 4.0 3.5 3.0 2.5 2.0 1.5 1.0 0.5 0.0 -0.5

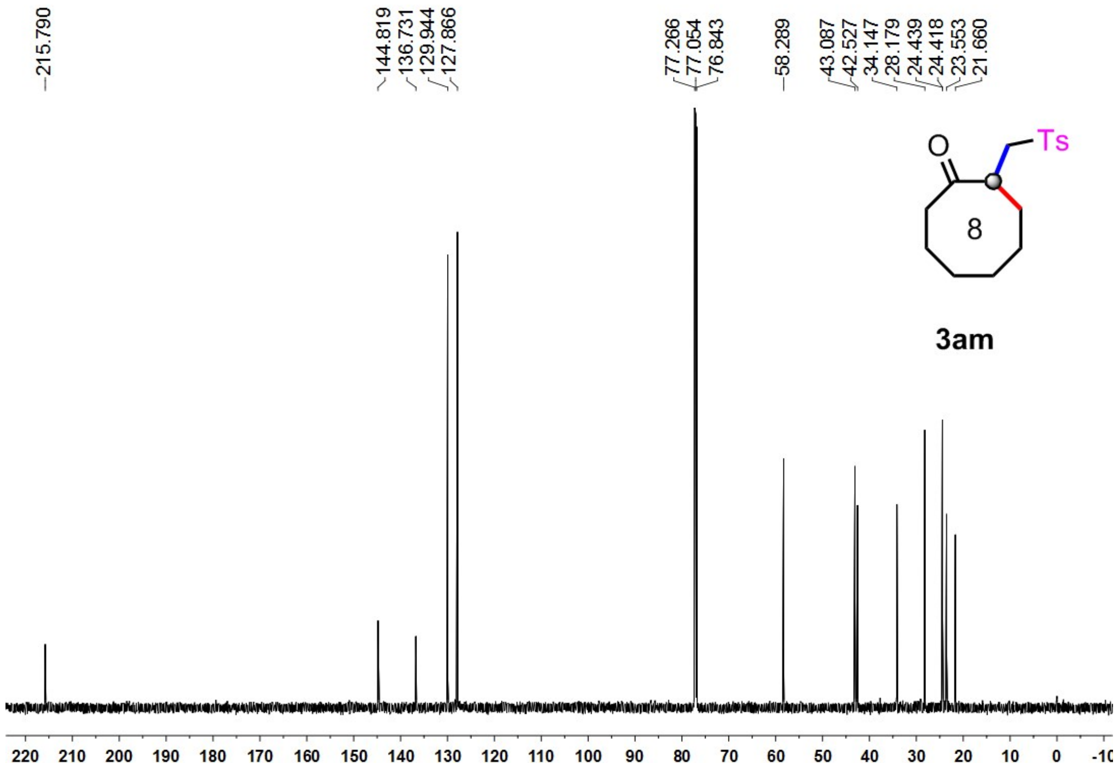
215.790
144.819
136.731
129.944
127.866
77.266
77.054
76.843
58.289
43.087
42.527
34.147
28.179
24.439
24.418
23.553
21.660
3am
8
O
Ts
220 210 200 190 180 170 160 150 140 130 120 110 100 90 80 70 60 50 40 30 20 10 0 -10

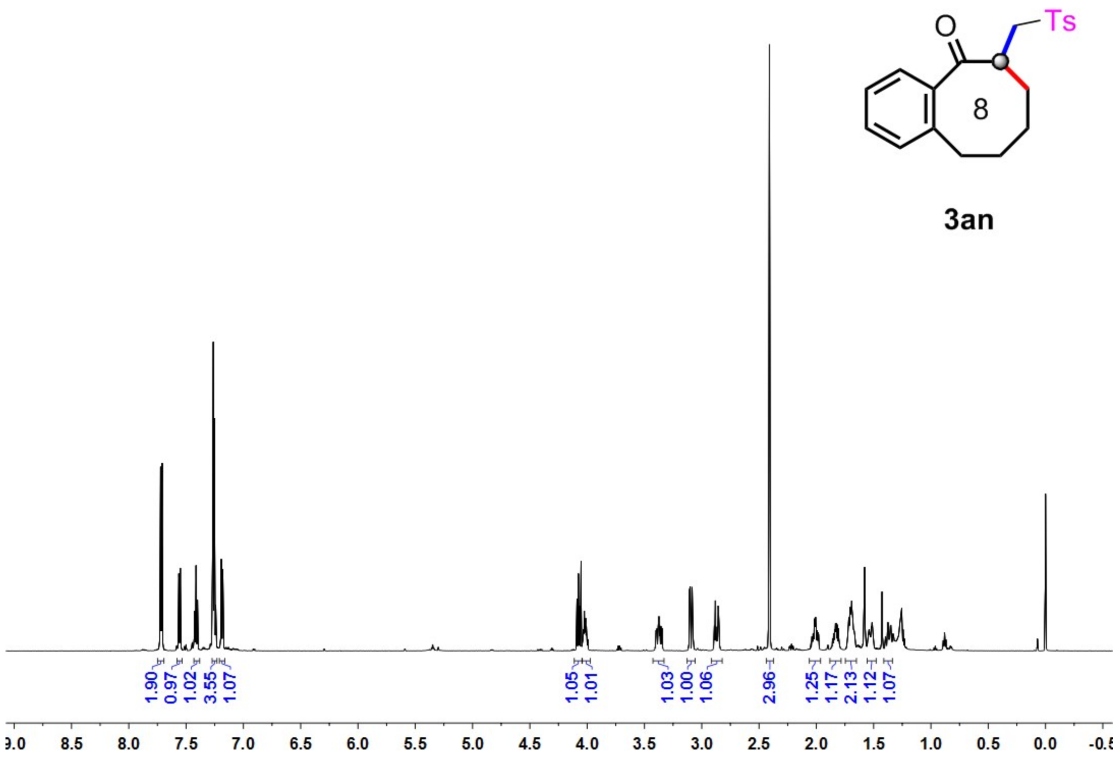
Ts
8
3an
1.90
0.97
1.02
3.55
1.07
1.05
1.01
1.03
1.00
1.06
2.96
1.25
1.17
2.13
1.12
1.07

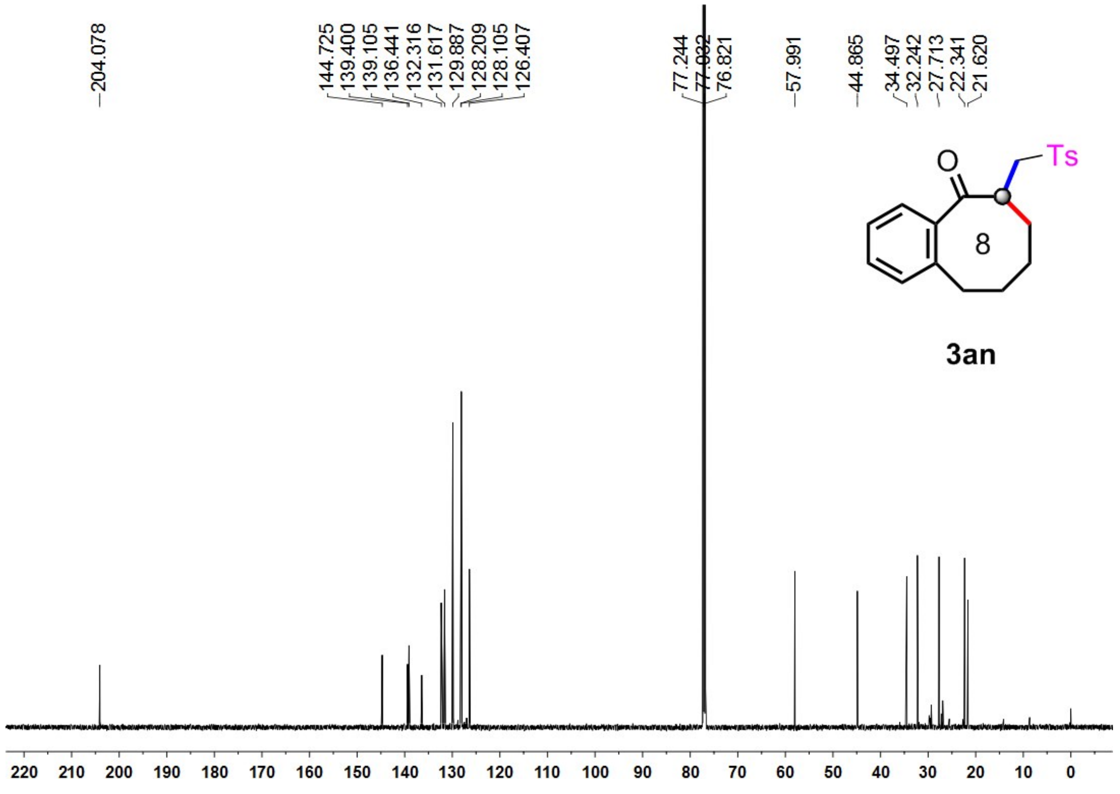
204.078
144.725
139.400
139.105
136.441
132.316
131.617
129.887
128.209
128.105
126.407
77.244
76.821
57.991
44.865
34.497
32.242
27.713
22.341
21.620
Ts
8
3an

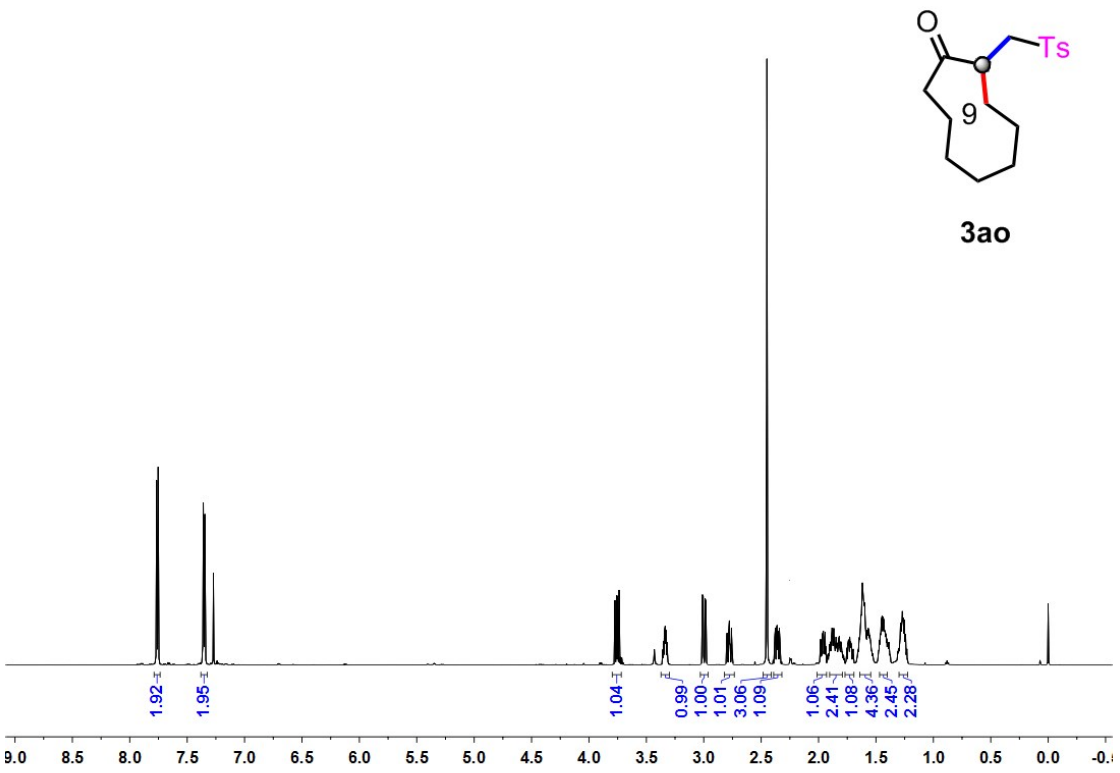
O
Ts
9
3ao
1.92
1.95
1.04
0.99
1.00
1.01
3.06
1.09
1.06
2.41
1.08
4.36
2.45
2.28

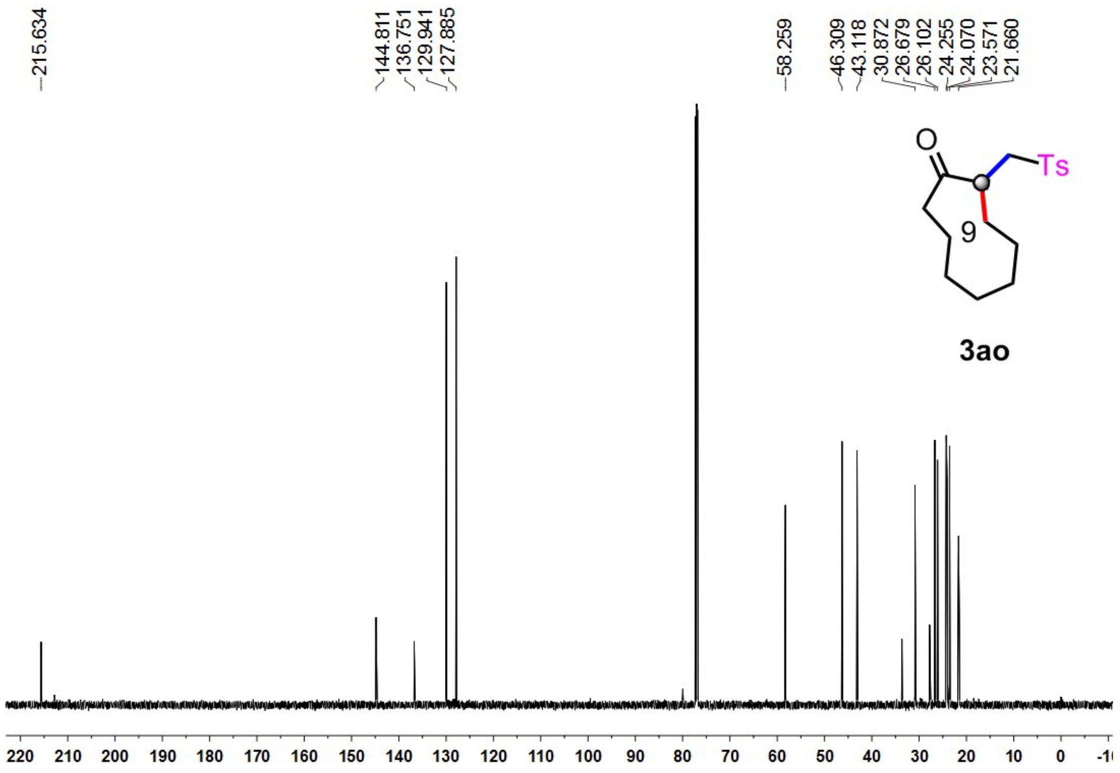
215.634
144.811
136.751
129.941
127.885
58.259
46.309
43.118
30.872
26.679
26.102
24.255
24.070
23.571
21.660
O
Ts
9
3ao

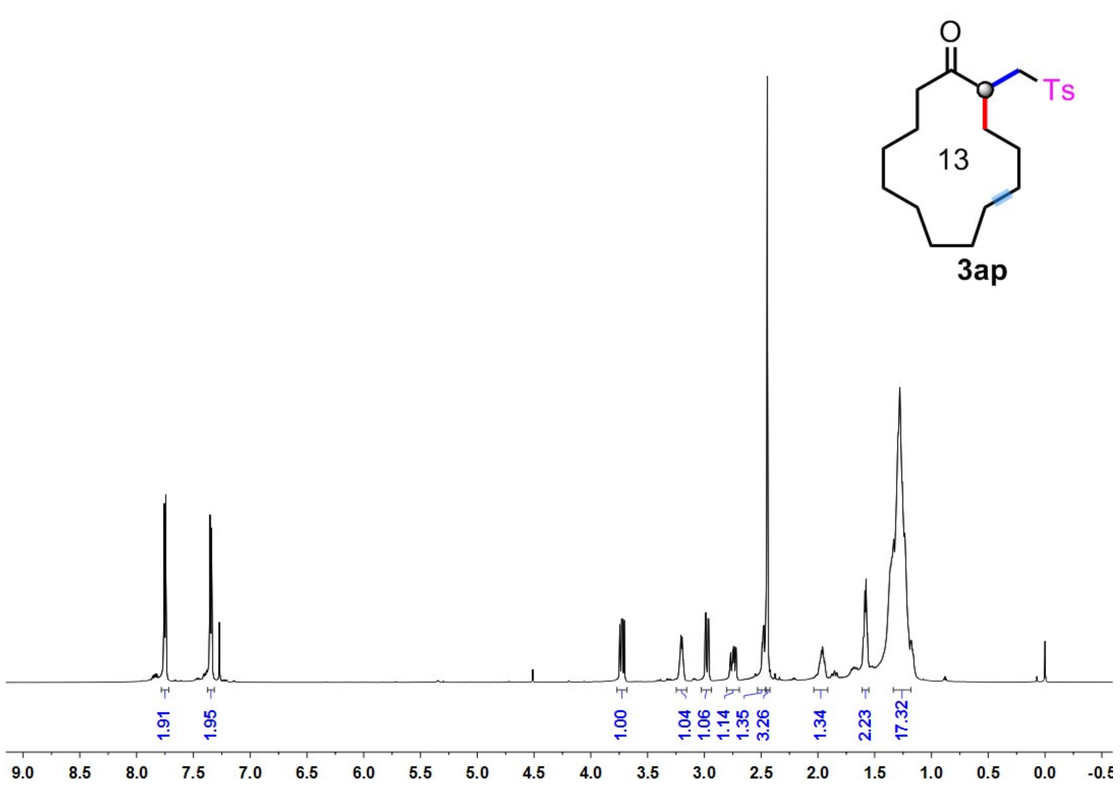

O
Ts
13
3ap
1.91
1.95
1.00
1.04
1.06
1.14
1.35
3.26
1.34
2.23
17.32
9.0 8.5 8.0 7.5 7.0 6.5 6.0 5.5 5.0 4.5 4.0 3.5 3.0 2.5 2.0 1.5 1.0 0.5 0.0

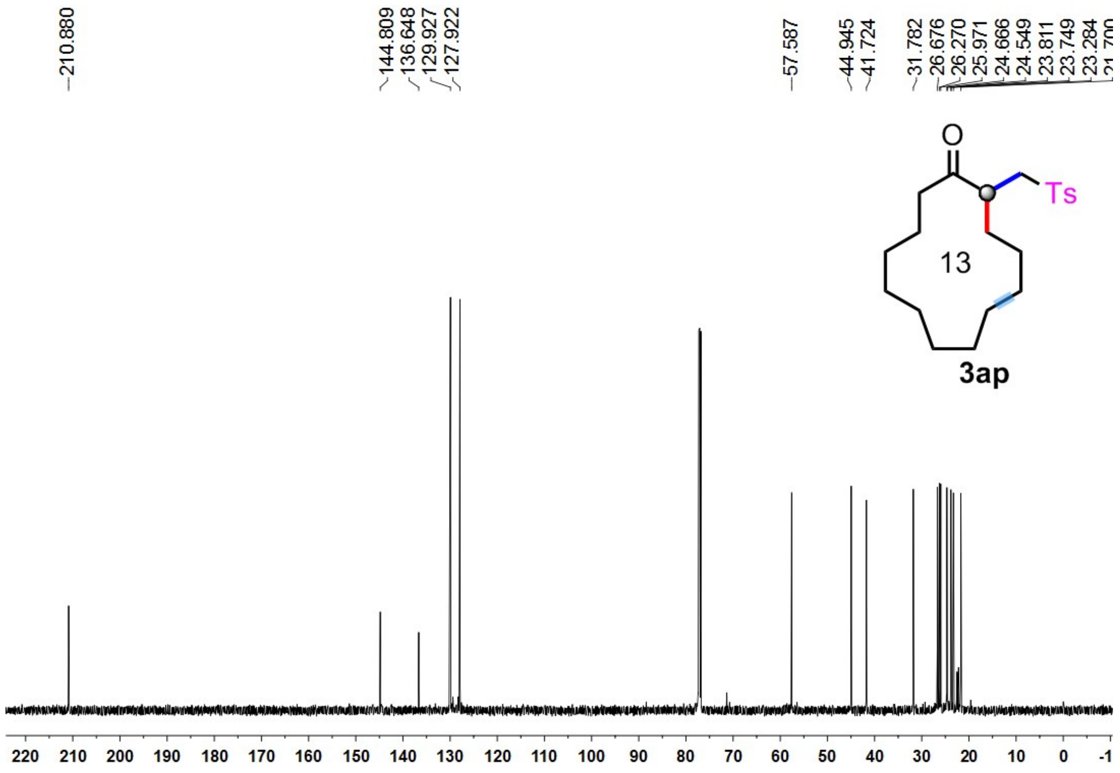

210.880
144.809
136.648
129.927
127.922
57.587
44.945
41.724
31.782
26.676
26.270
25.971
24.666
24.549
23.811
23.749
23.284
O
Ts
13
3ap
220 210 200 190 180 170 160 150 140 130 120 110 100 90 80 70 60 50 40 30 20 10 0

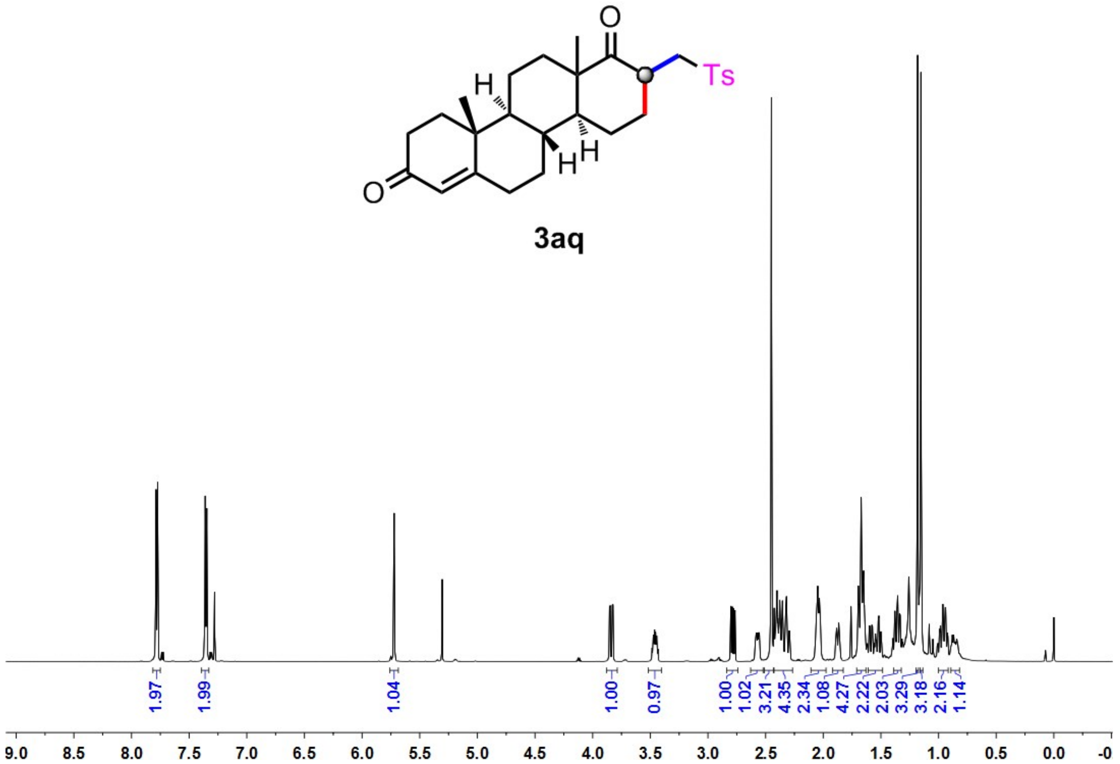
O
H
H
O
Ts
3aq
1.97
1.99
1.04
1.00
0.97
1.00
1.02
3.21
4.35
2.34
1.08
4.27
2.22
2.03
3.29
3.18
2.16
1.14
9.0 8.5 8.0 7.5 7.0 6.5 6.0 5.5 5.0 4.5 4.0 3.5 3.0 2.5 2.0 1.5 1.0 0.5 0.0 -0

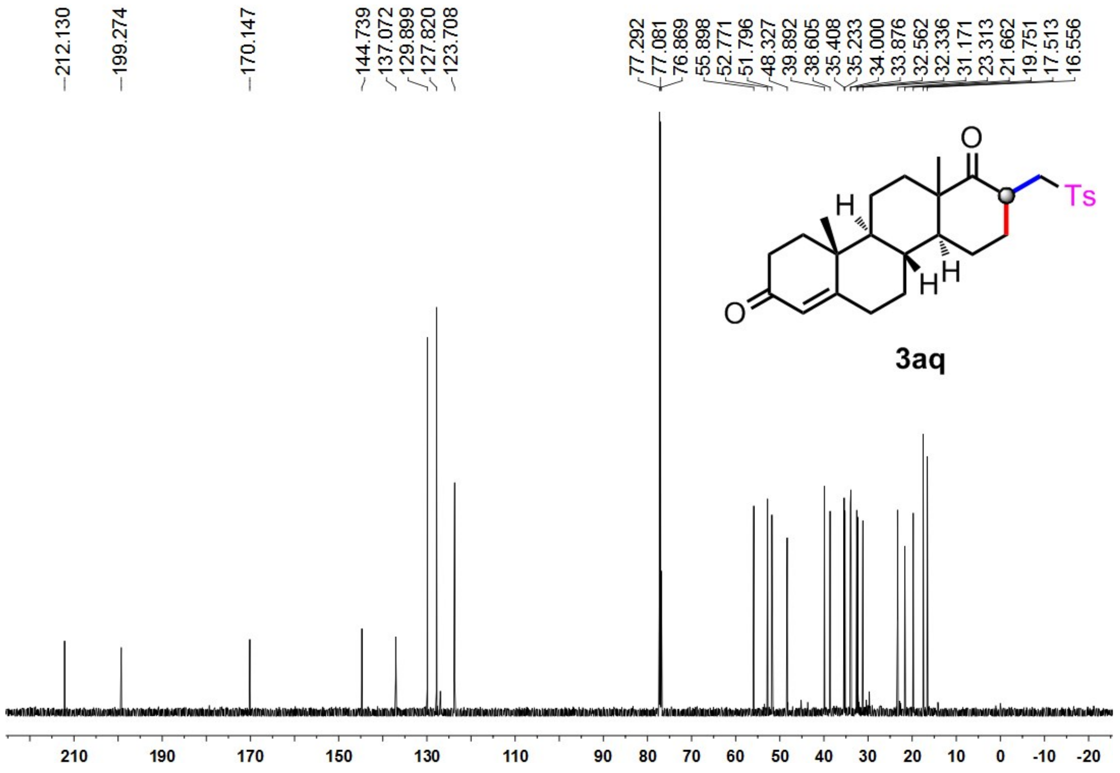
212.130
199.274
170.147
144.739
137.072
129.899
127.820
123.708
77.292
77.081
76.869
55.898
52.771
51.796
48.327
39.892
38.605
35.408
35.233
34.000
33.876
32.562
32.336
31.171
23.313
21.662
19.751
17.513
16.556
O
H
H
O
Ts
3aq
210 190 170 150 130 110 90 80 70 60 50 40 30 20 10 0 -10 -20

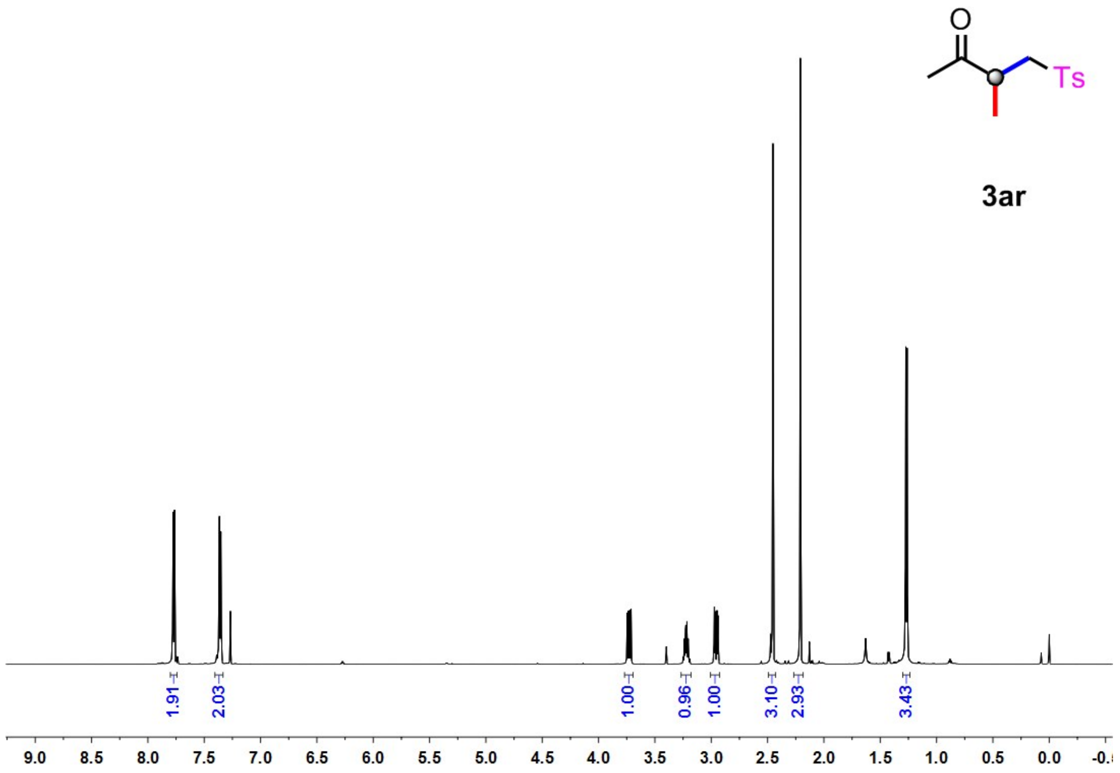
O
Ts
3ar
1.91
2.03
1.00
0.96
1.00
3.10
2.93
3.43
9.0 8.5 8.0 7.5 7.0 6.5 6.0 5.5 5.0 4.5 4.0 3.5 3.0 2.5 2.0 1.5 1.0 0.5 0.0

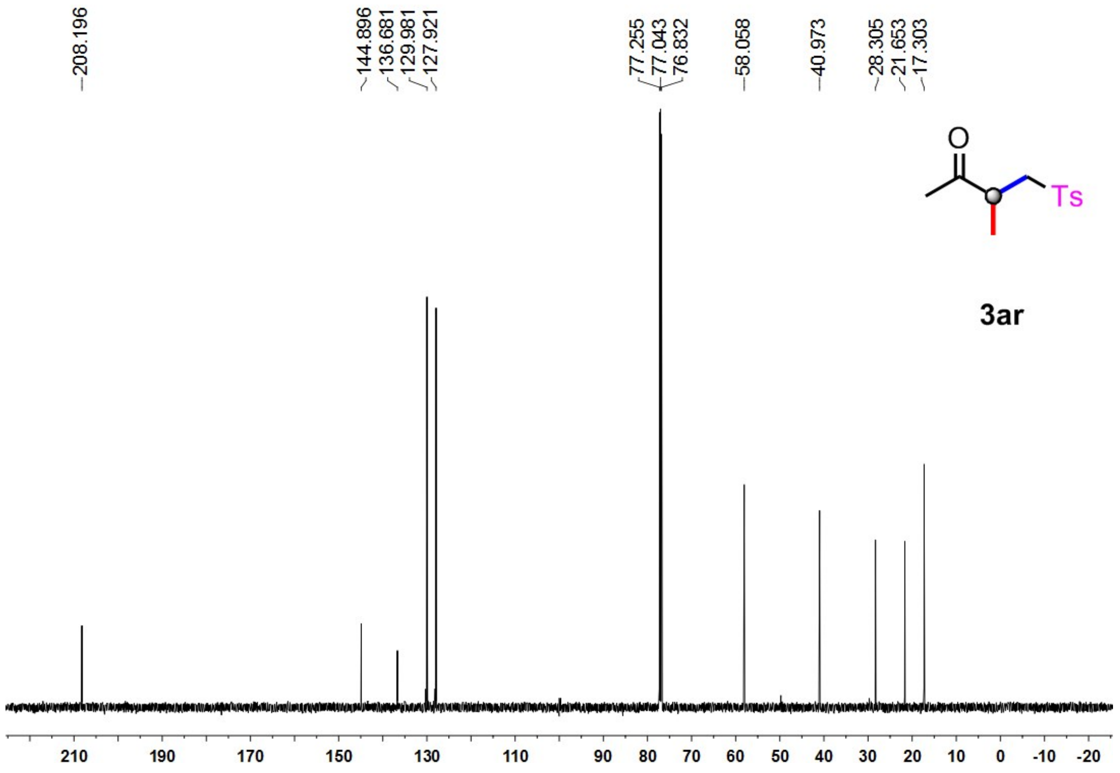
208.196
144.896
136.681
129.981
127.921
77.255
77.043
76.832
58.058
40.973
28.305
21.653
17.303
O
Ts
3ar
210 190 170 150 130 110 90 80 70 60 50 40 30 20 10 0 -10 -20

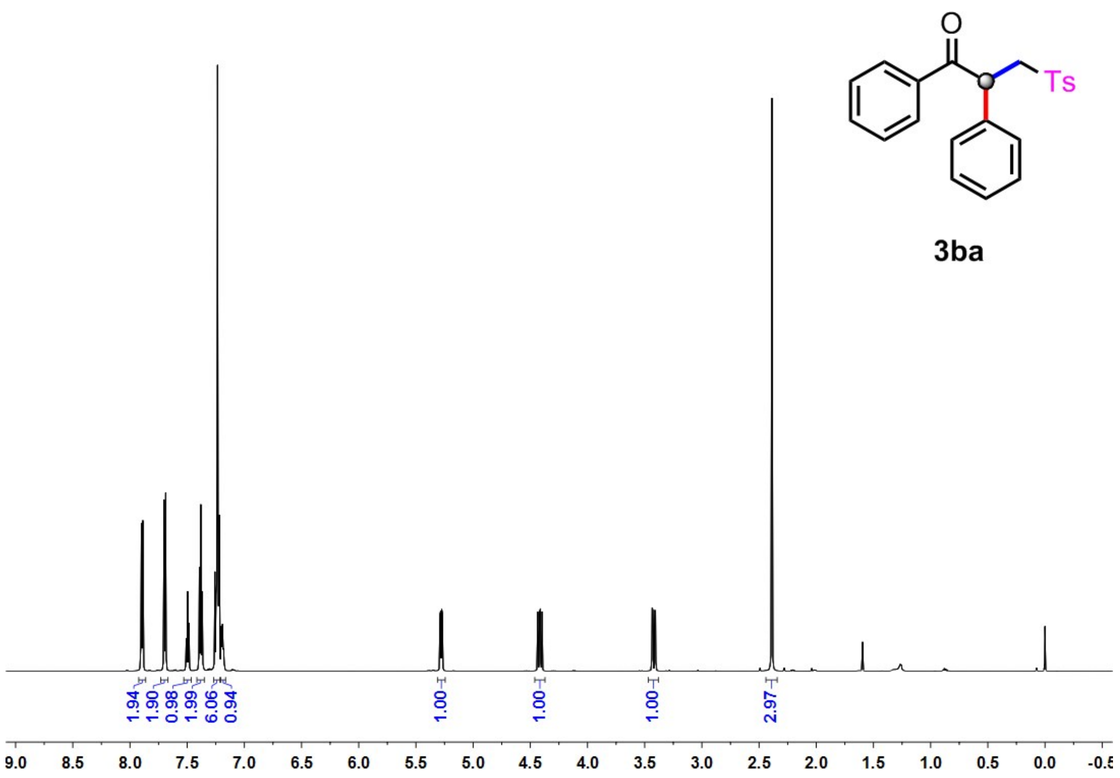
3ba
Ts
1.94
1.90
0.98
1.99
6.06
0.94
1.00
1.00
1.00
2.97
9.0 8.5 8.0 7.5 7.0 6.5 6.0 5.5 5.0 4.5 4.0 3.5 3.0 2.5 2.0 1.5 1.0 0.5 0.0 -0.5

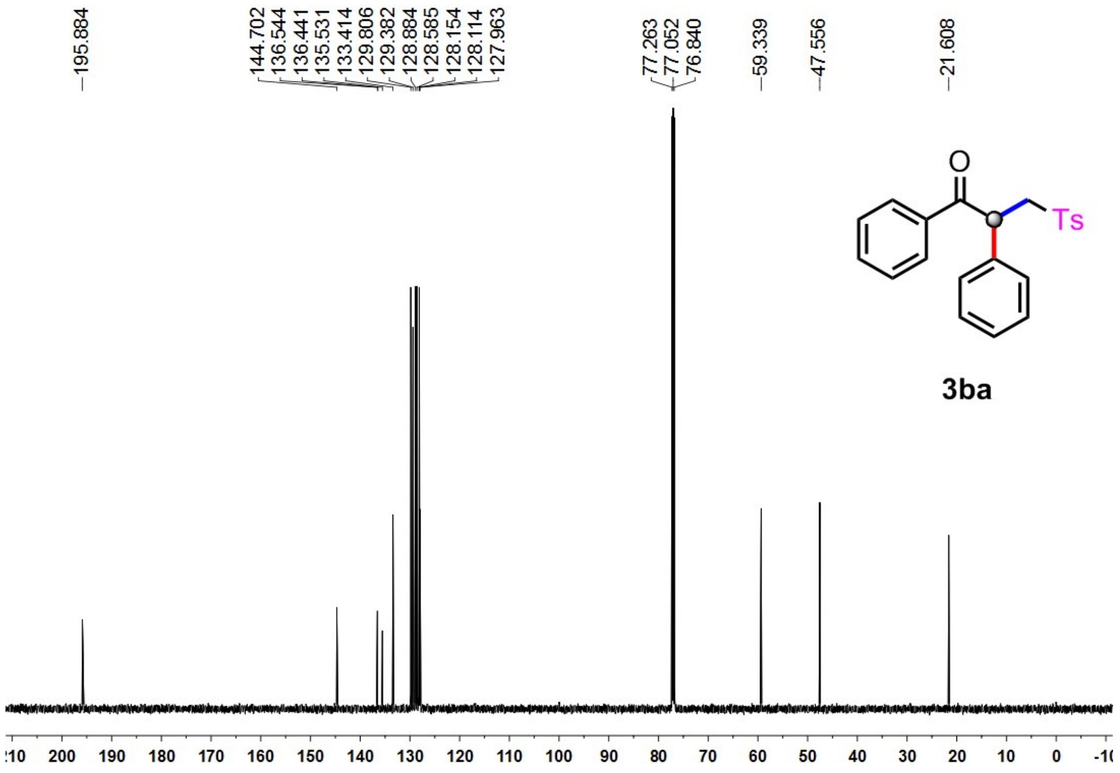
195.884
144.702
136.544
136.441
135.531
133.414
129.806
129.382
128.884
128.585
128.154
128.114
127.963
77.263
77.052
76.840
59.339
47.556
21.608
3ba
Ts
210 200 190 180 170 160 150 140 130 120 110 100 90 80 70 60 50 40 30 20 10 0 -10

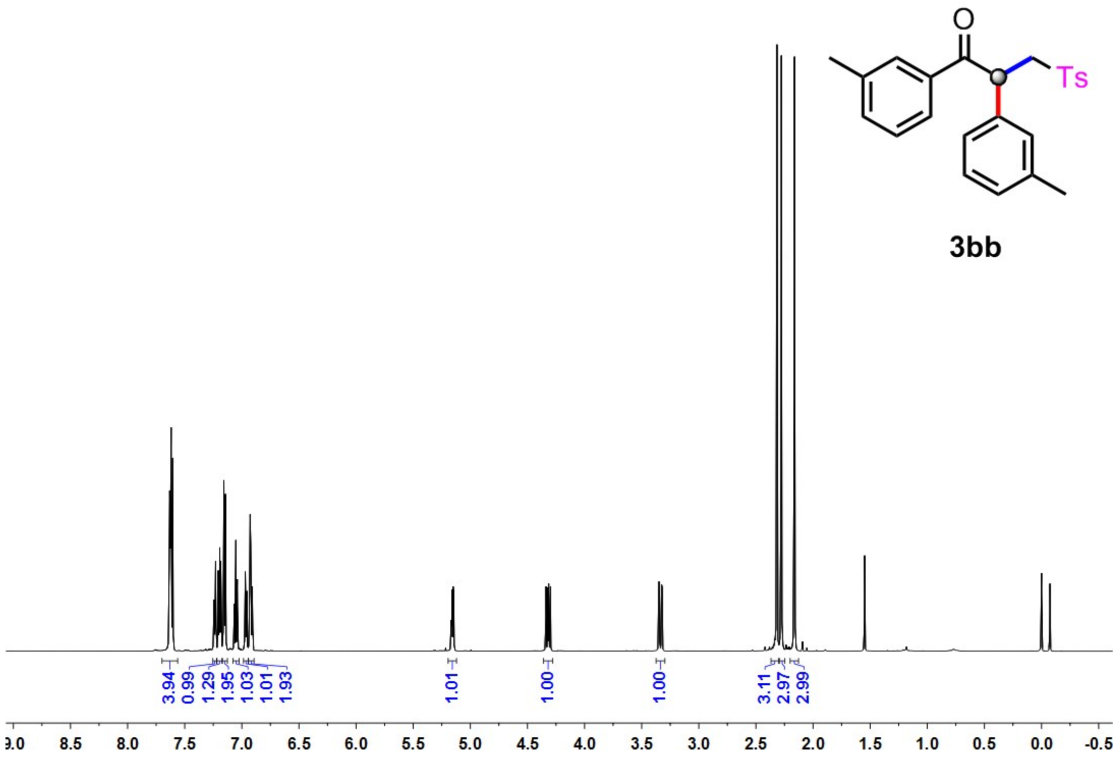
O
Ts
3bb
3.94
0.99
1.29
1.95
1.03
1.01
1.93
1.01
1.00
1.00
3.11
2.97
2.99
9.0 8.5 8.0 7.5 7.0 6.5 6.0 5.5 5.0 4.5 4.0 3.5 3.0 2.5 2.0 1.5 1.0 0.5 0.0 -0.5

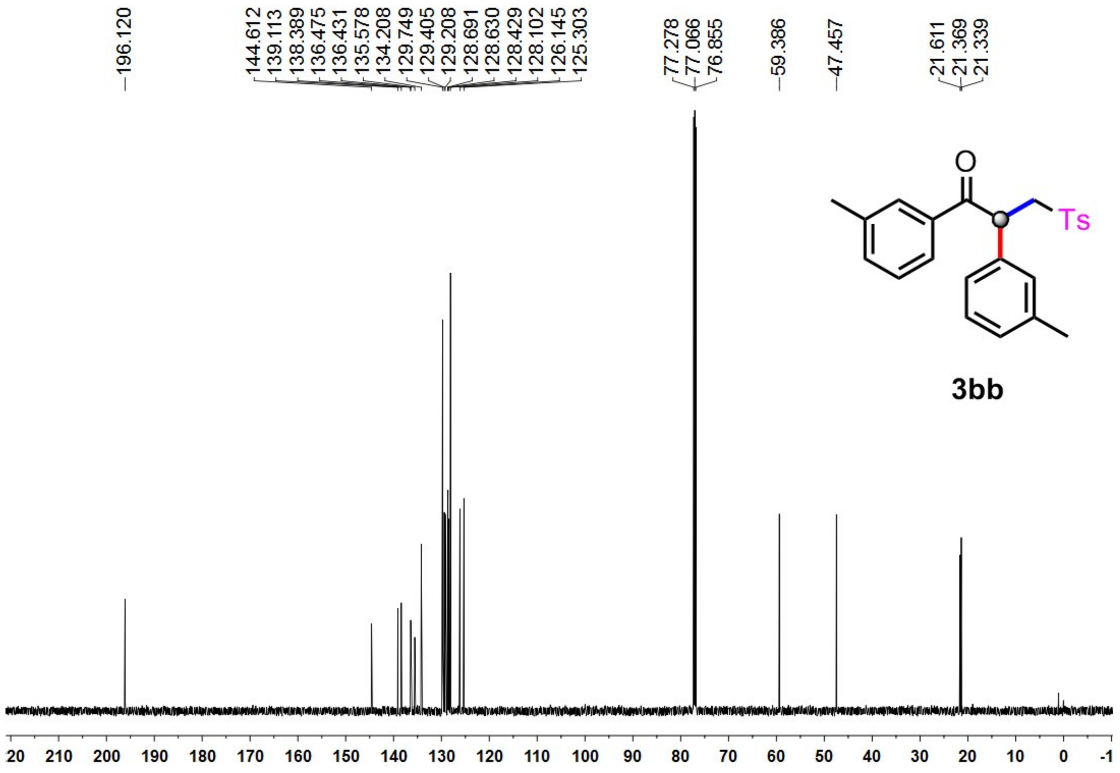
196.120
144.612
139.113
138.389
136.475
136.431
135.578
134.208
129.749
129.405
129.208
128.691
128.630
128.429
128.102
126.145
125.303
77.278
77.066
76.855
59.386
47.457
21.611
21.369
21.339
O
Ts
3bb
20 210 200 190 180 170 160 150 140 130 120 110 100 90 80 70 60 50 40 30 20 10 0 -1

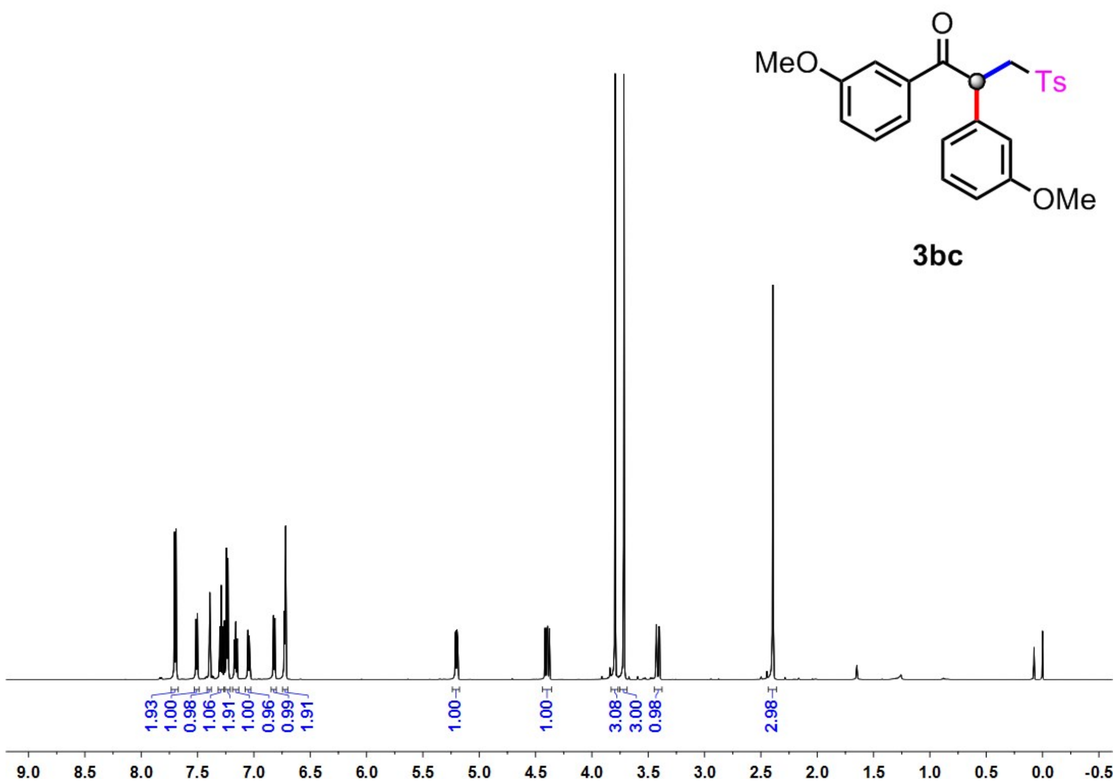

MeO
O
Ts
OMe
3bc
1.93
1.00
0.98
1.06
1.91
1.00
0.96
0.99
1.91
1.00
1.00
3.08
3.00
0.98
2.98
9.0 8.5 8.0 7.5 7.0 6.5 6.0 5.5 5.0 4.5 4.0 3.5 3.0 2.5 2.0 1.5 1.0 0.5 0.0 -0.5

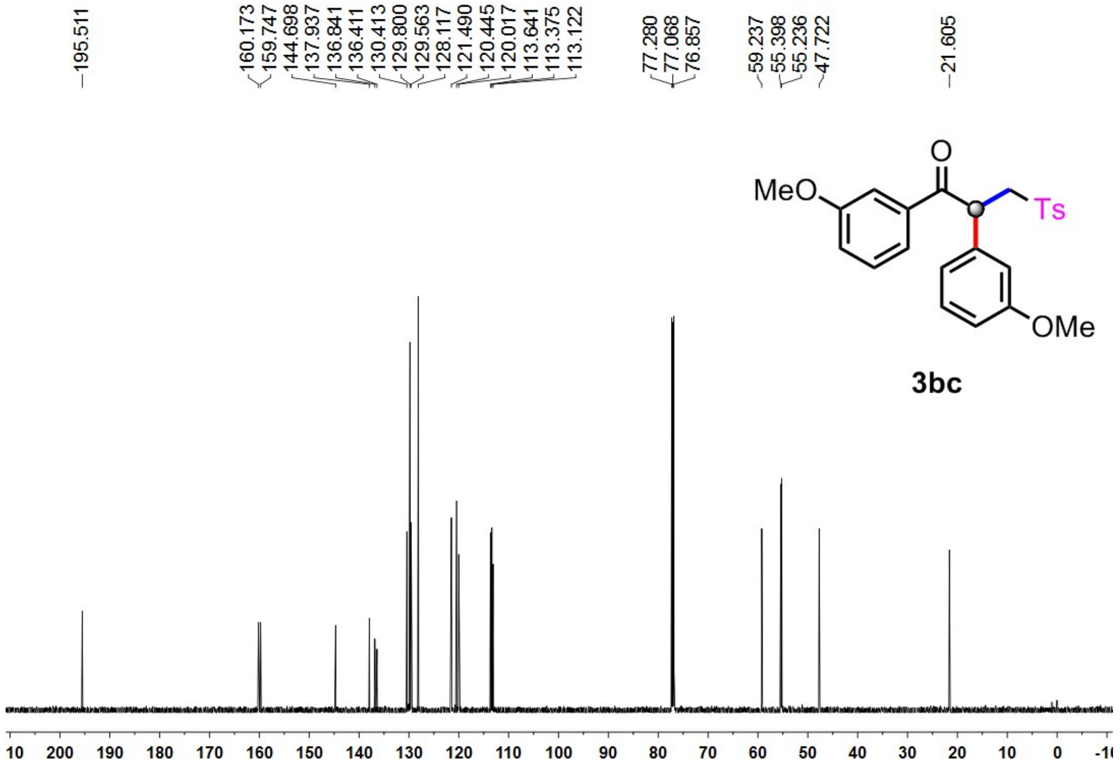

195.511
160.173
159.747
144.698
137.937
136.841
136.411
130.413
129.800
129.563
128.117
121.490
120.445
120.017
113.641
113.375
113.122
77.280
77.068
76.857
59.237
55.398
55.236
47.722
21.605
MeO
O
Ts
OMe
3bc
200 190 180 170 160 150 140 130 120 110 100 90 80 70 60 50 40 30 20 10 0

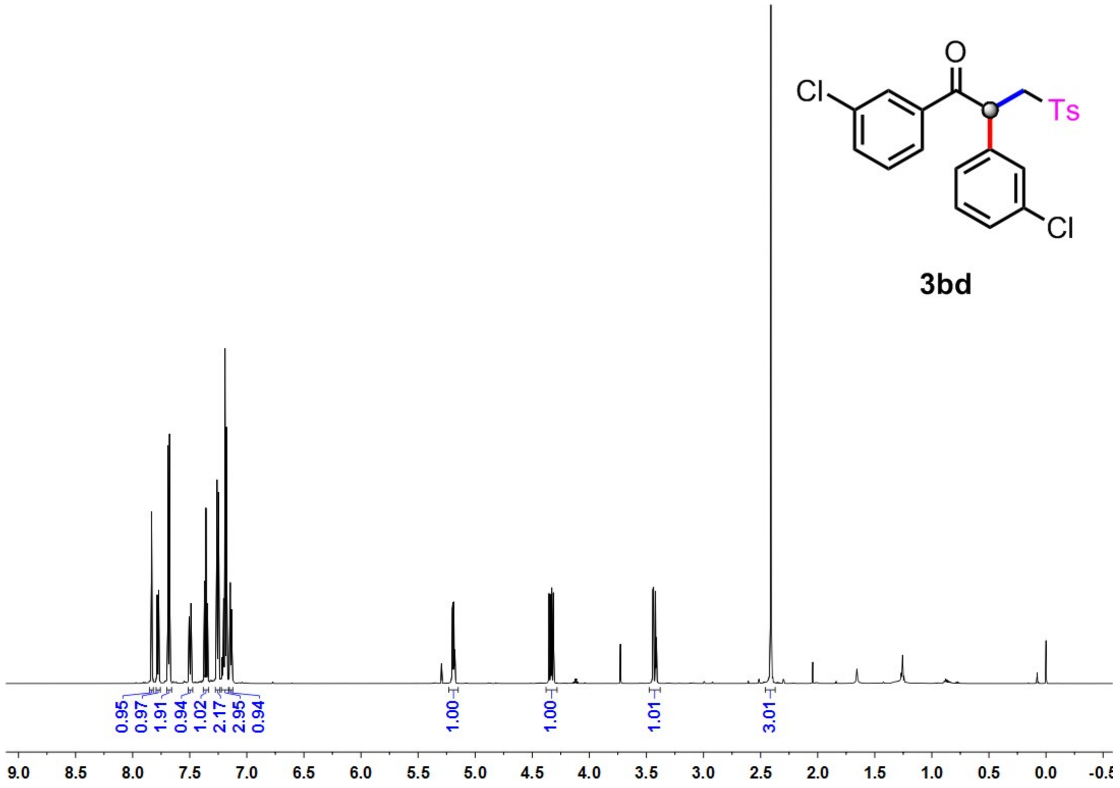
3bd
Ts
0.95
0.97
1.91
0.94
1.02
2.17
2.95
0.94
1.00
1.00
1.01
3.01

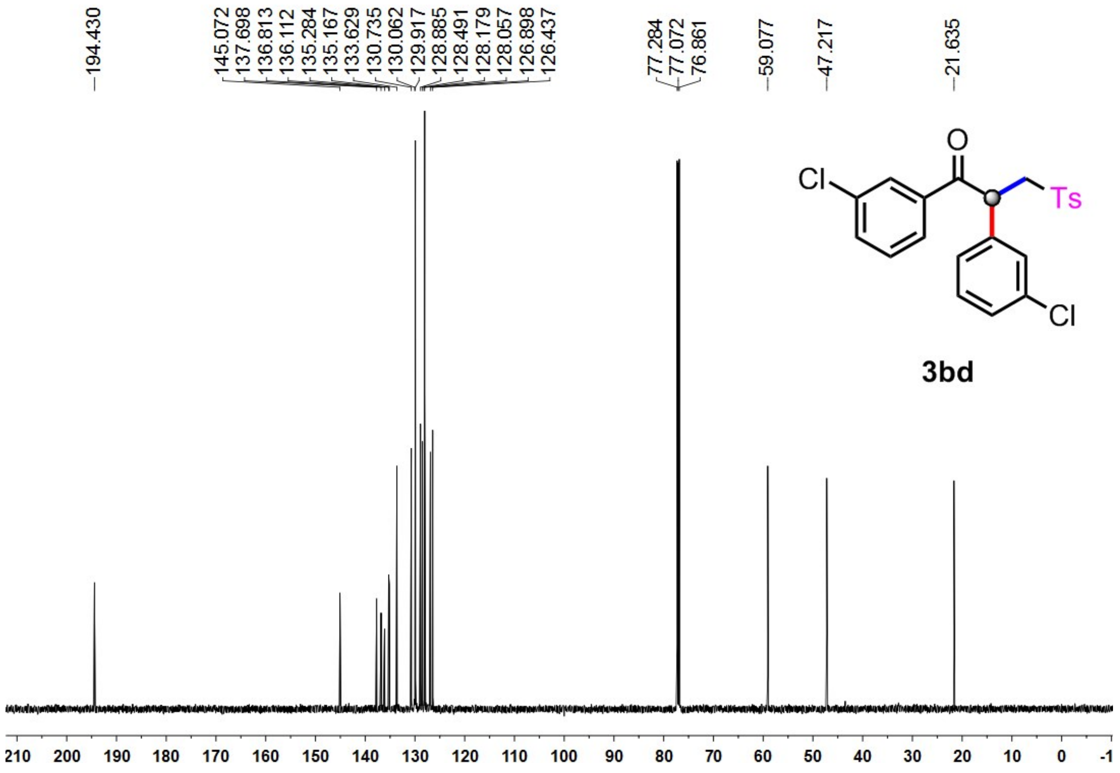
194.430
145.072
137.698
136.813
136.112
135.284
135.167
133.629
130.735
130.062
129.917
128.885
128.491
128.179
128.057
126.898
126.437
77.284
77.072
76.861
59.077
47.217
21.635
3bd
Ts

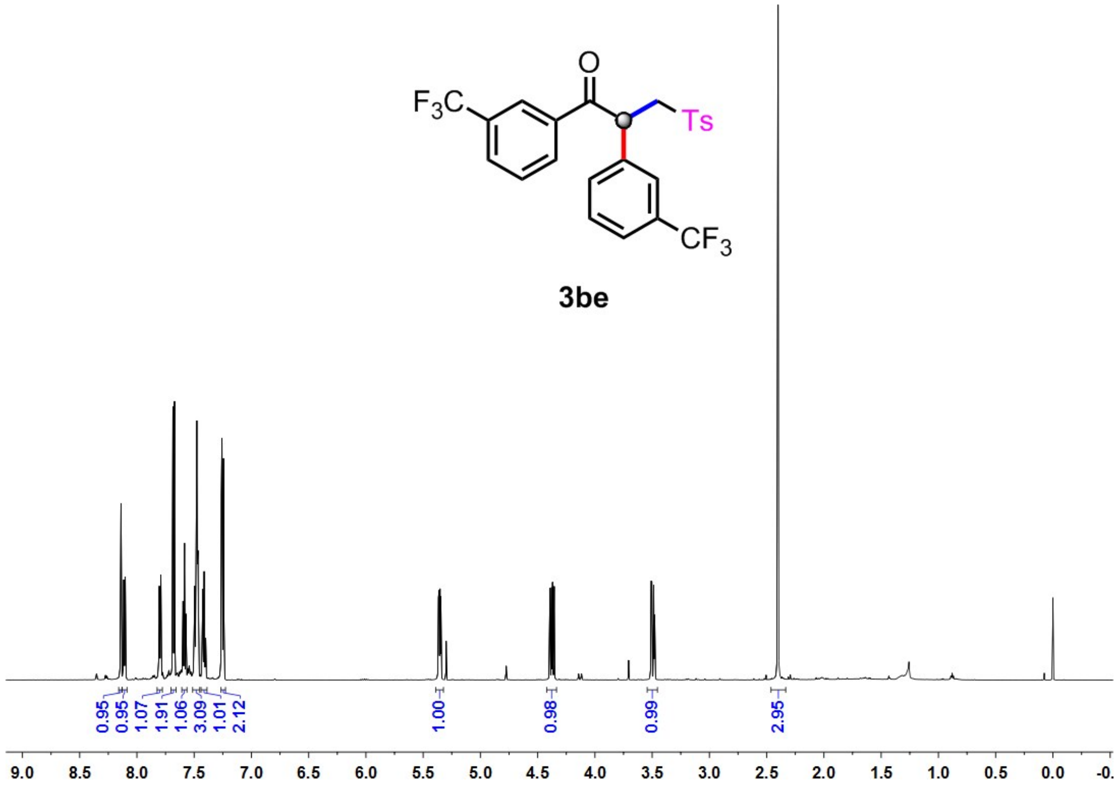

F3C
O
Ts
CF3
3be
0.95
0.95
1.07
1.91
1.06
3.09
1.01
2.12
1.00
0.98
0.99
2.95
9.0 8.5 8.0 7.5 7.0 6.5 6.0 5.5 5.0 4.5 4.0 3.5 3.0 2.5 2.0 1.5 1.0 0.5 0.0

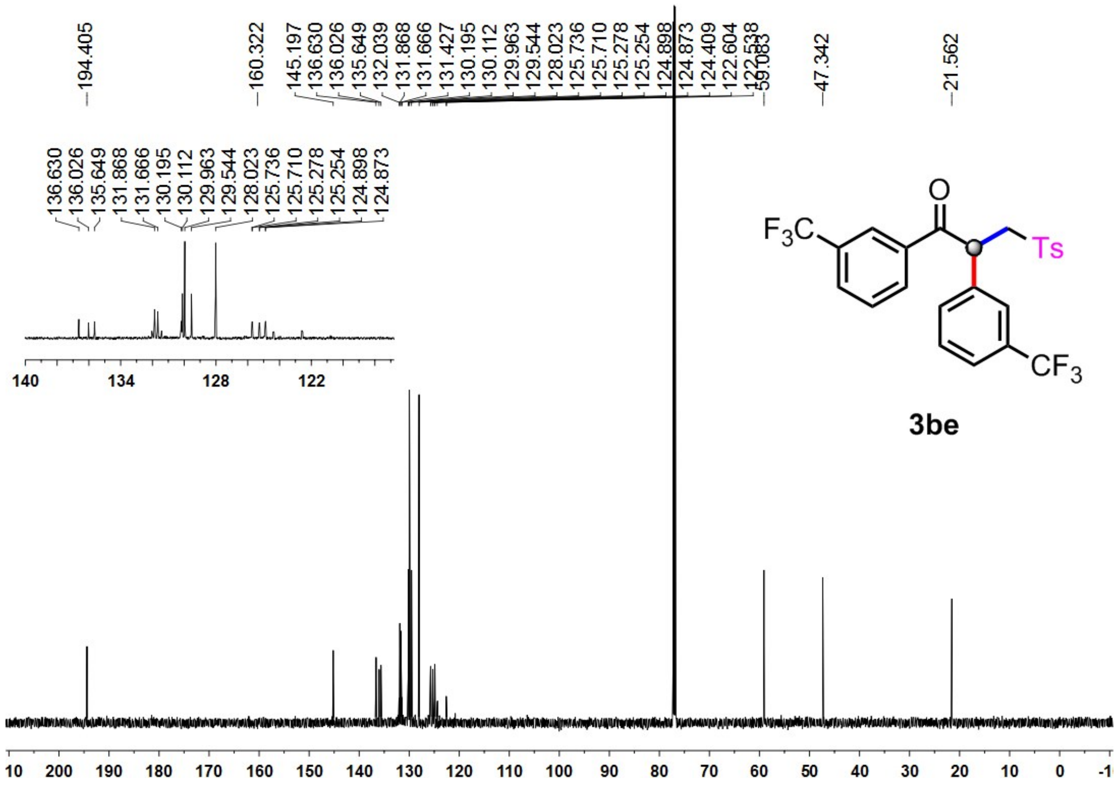

194.405
160.322
145.197
136.630
136.026
135.649
132.039
131.868
131.666
131.427
130.195
130.112
129.963
129.544
128.023
125.736
125.710
125.278
125.254
124.898
124.873
124.409
122.604
47.342
21.562
140 134 128 122
F3C
O
Ts
CF3
3be
200 190 180 170 160 150 140 130 120 110 100 90 80 70 60 50 40 30 20 10 0

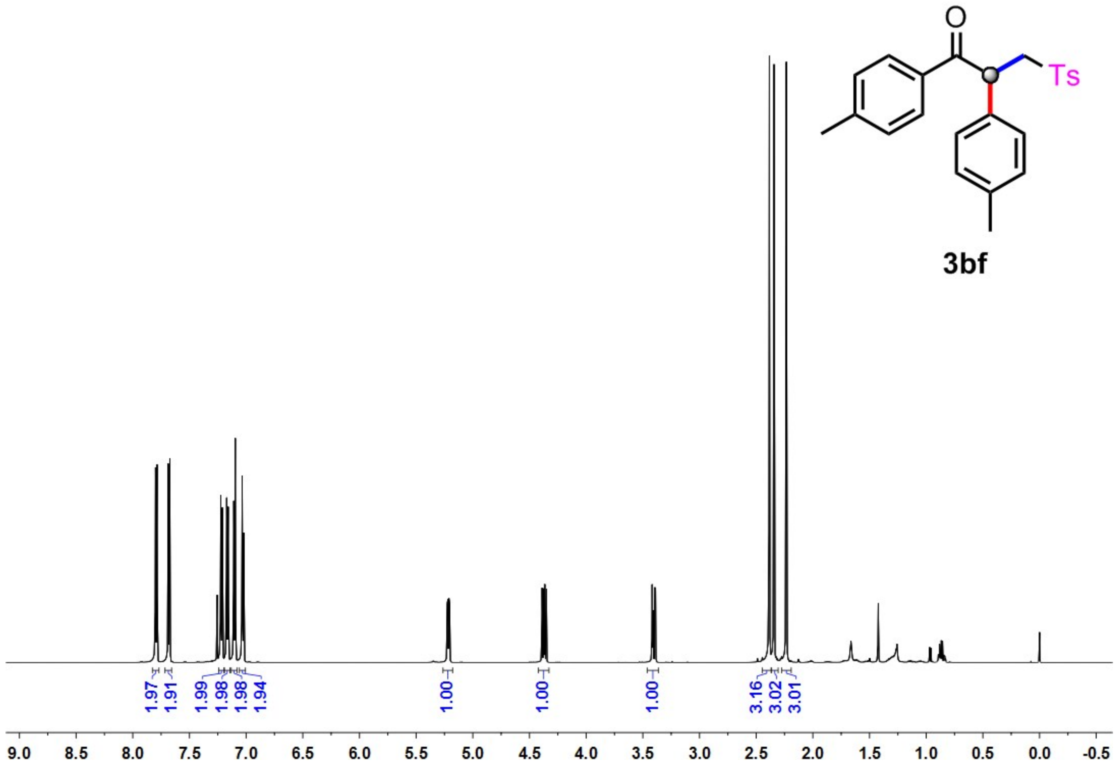
O
Ts
3bf
1.97
1.91
1.99
1.98
1.98
1.94
1.00
1.00
1.00
3.16
3.02
3.01

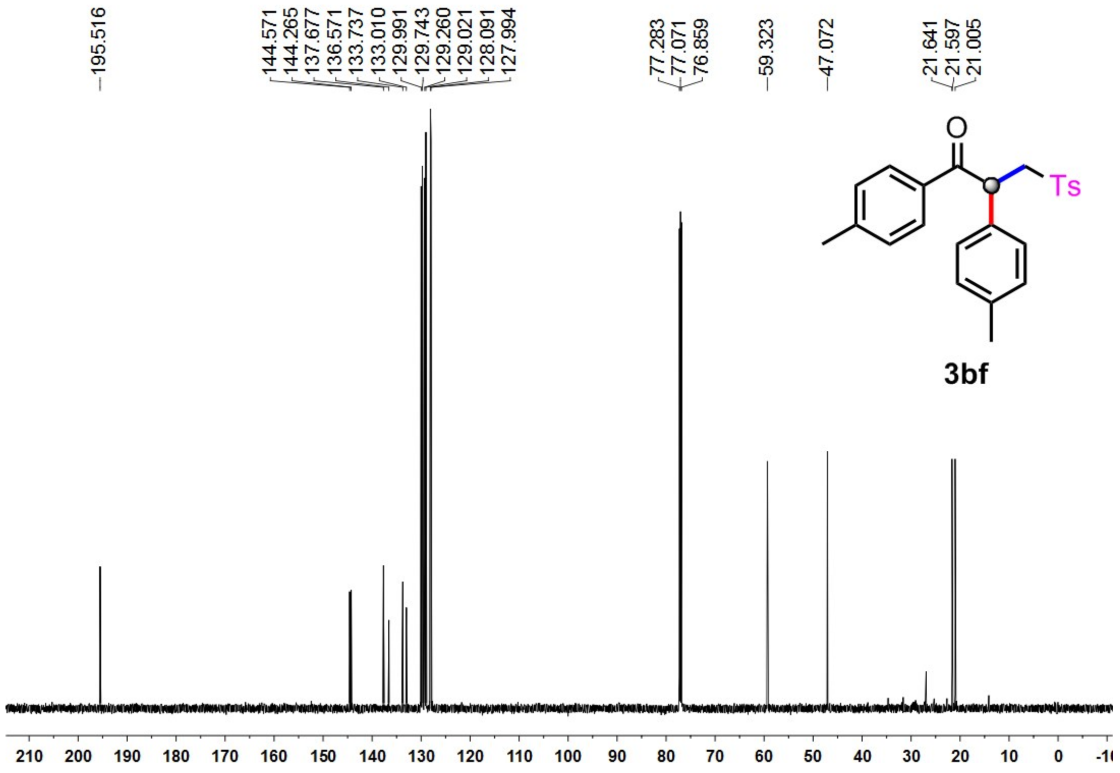
195.516
144.571
144.265
137.677
136.571
133.737
133.010
129.991
129.743
129.260
129.021
128.091
127.994
77.283
77.071
76.859
59.323
47.072
21.641
21.597
21.005
O
Ts
3bf

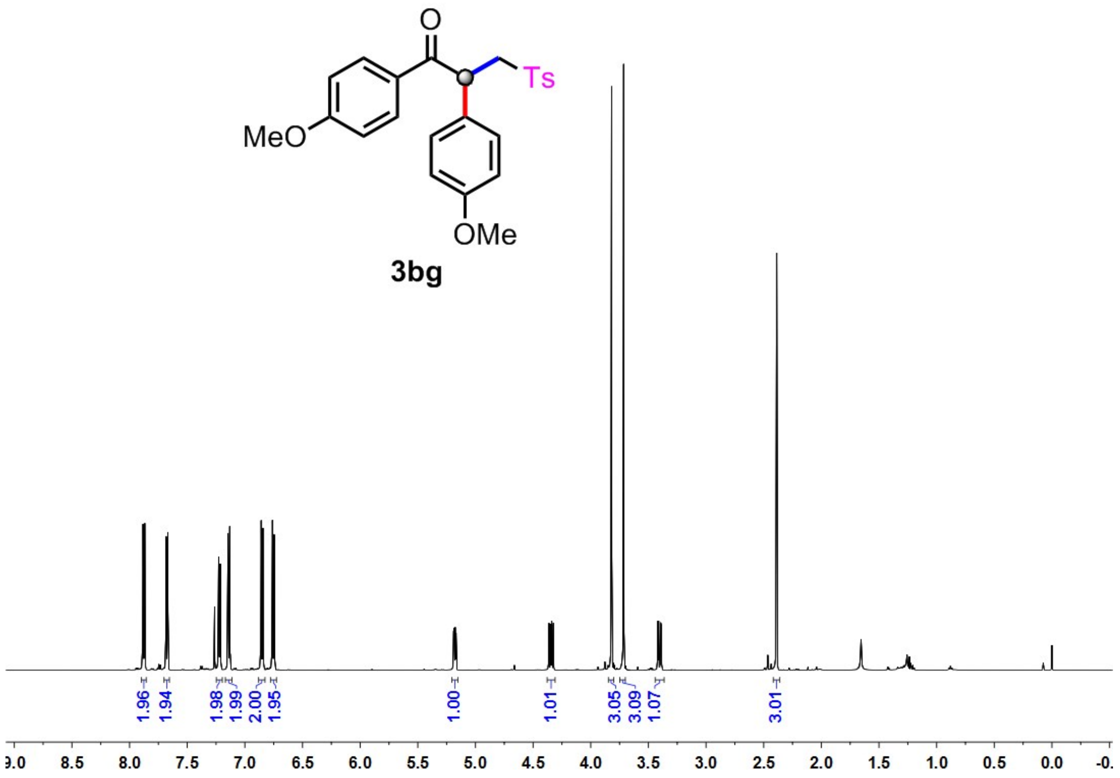
MeO
OMe
Ts
O
3bg
1.96
1.94
1.98
1.99
2.00
1.95
1.00
1.01
3.05
3.09
1.07
3.01
9.0 8.5 8.0 7.5 7.0 6.5 6.0 5.5 5.0 4.5 4.0 3.5 3.0 2.5 2.0 1.5 1.0 0.5 0.0 -0.

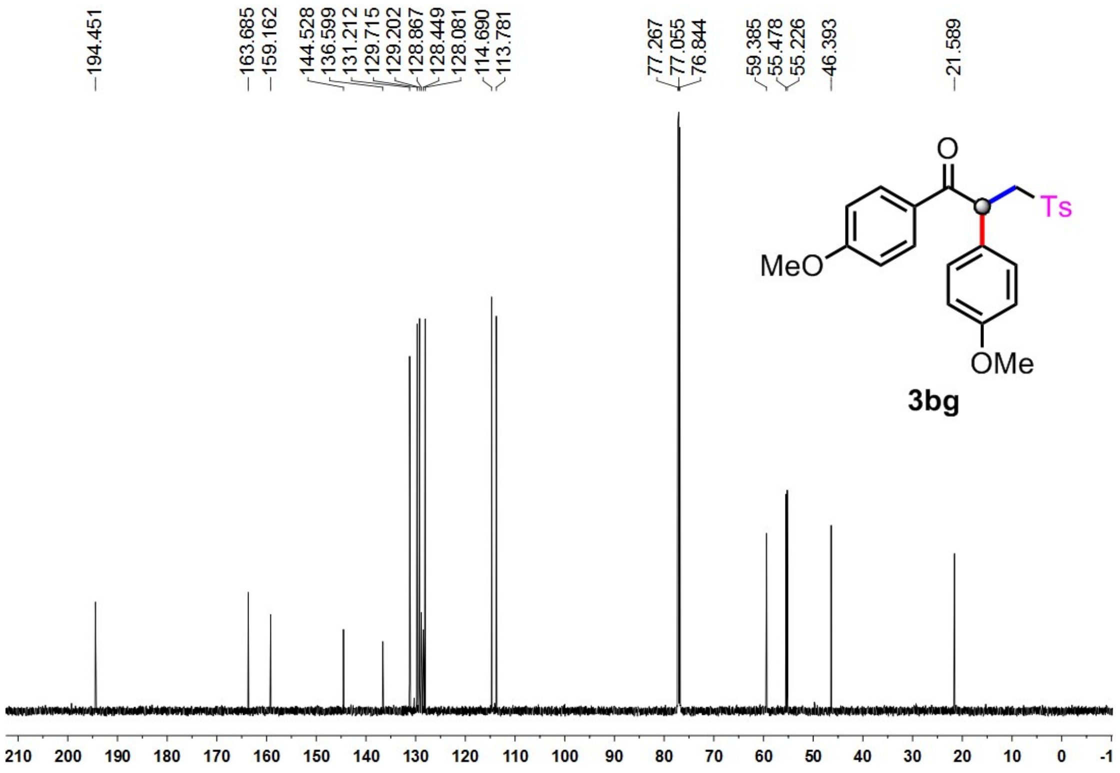
194.451
163.685
159.162
144.528
136.599
131.212
129.715
129.202
128.867
128.449
128.081
114.690
113.781
77.267
77.055
76.844
59.385
55.478
55.226
46.393
21.589
MeO
OMe
Ts
O
3bg
210 200 190 180 170 160 150 140 130 120 110 100 90 80 70 60 50 40 30 20 10 0 -1

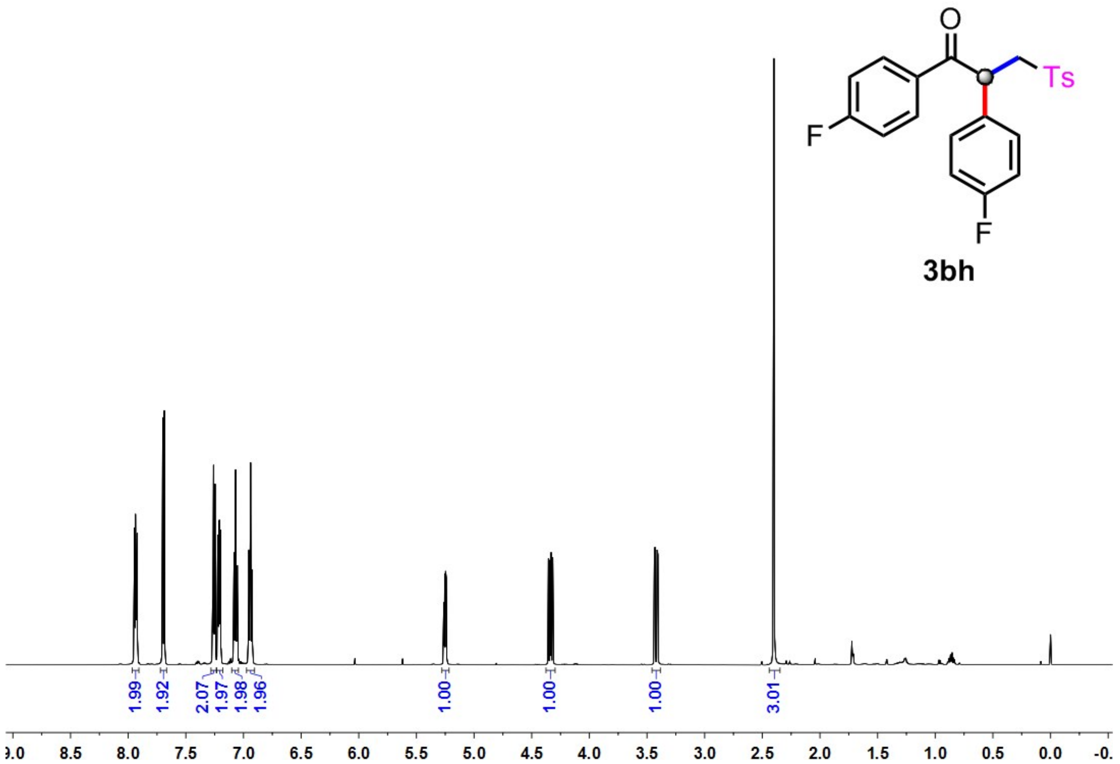
O
Ts
F
F
1.99
1.92
2.07
1.97
1.98
1.96
1.00
1.00
1.00
3.01
9.0 8.5 8.0 7.5 7.0 6.5 6.0 5.5 5.0 4.5 4.0 3.5 3.0 2.5 2.0 1.5 1.0 0.5 0.0 -0.

3bh

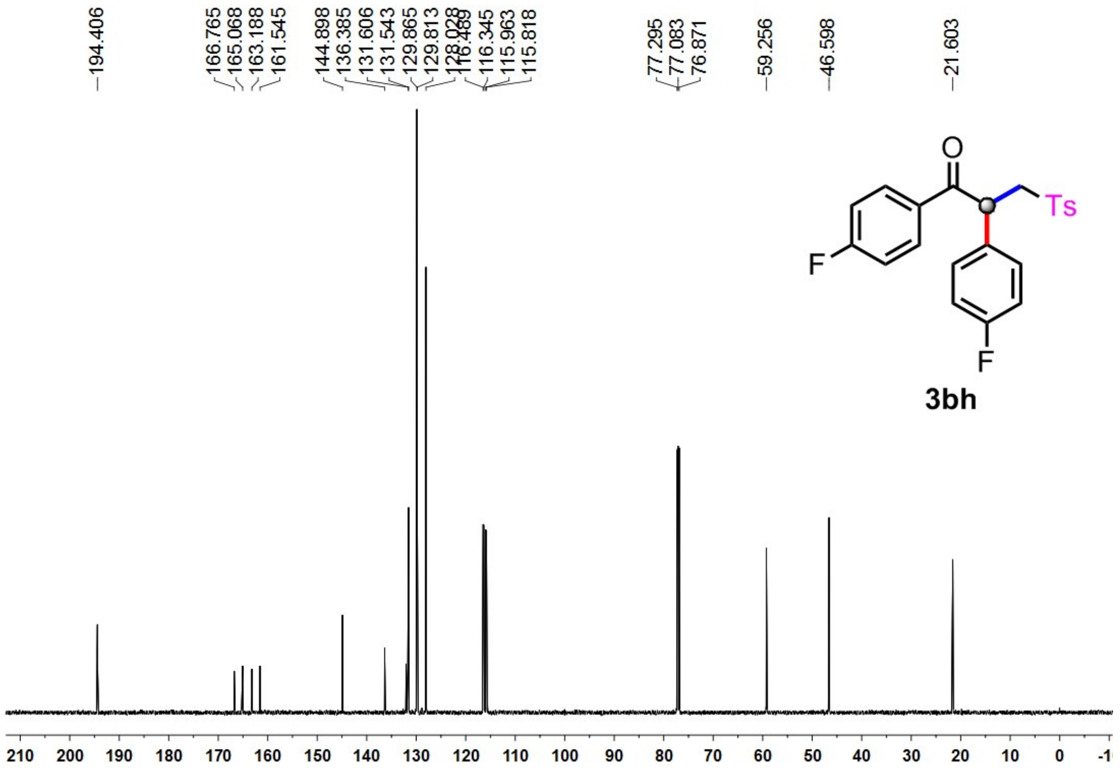
194.406
166.765
165.068
163.188
161.545
144.898
136.385
131.606
131.543
129.865
129.813
128.028
116.489
116.345
115.963
115.818
77.295
77.083
76.871
59.256
46.598
21.603
O
Ts
F
F
210 200 190 180 170 160 150 140 130 120 110 100 90 80 70 60 50 40 30 20 10 0 -1

3bh

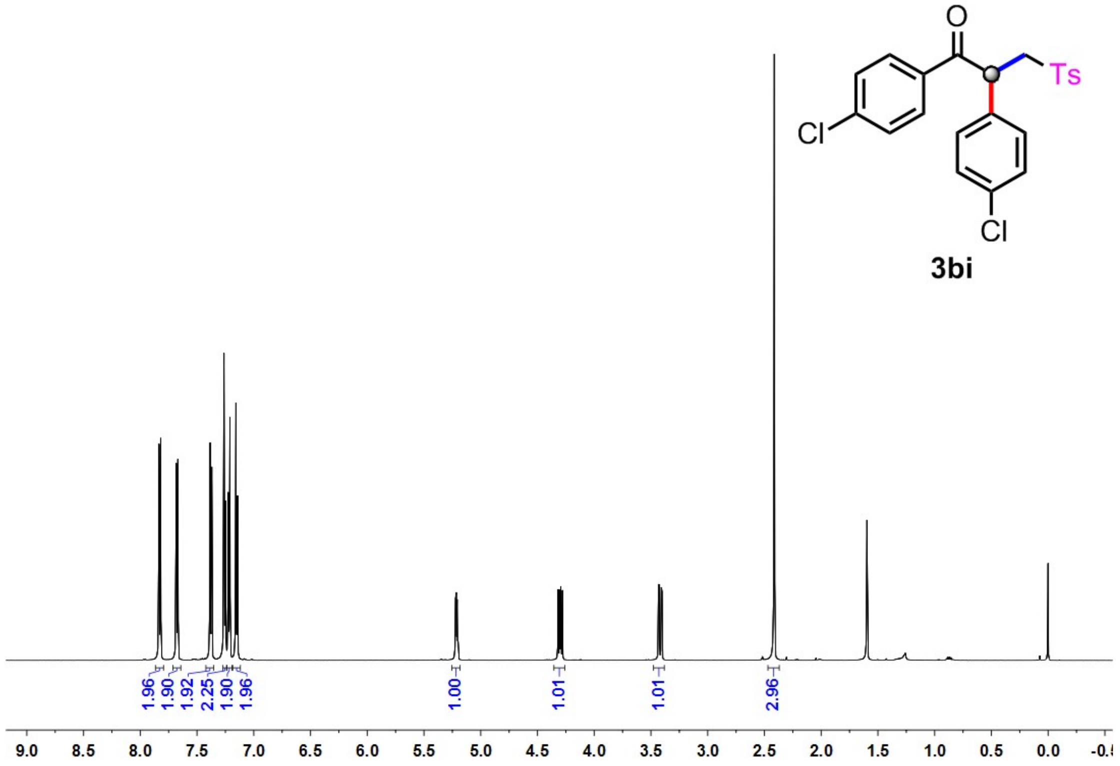

O
Ts
Cl
Cl
3bi
1.96
1.90
1.92
2.25
1.90
1.96
1.00
1.01
1.01
2.96
9.0 8.5 8.0 7.5 7.0 6.5 6.0 5.5 5.0 4.5 4.0 3.5 3.0 2.5 2.0 1.5 1.0 0.5 0.0

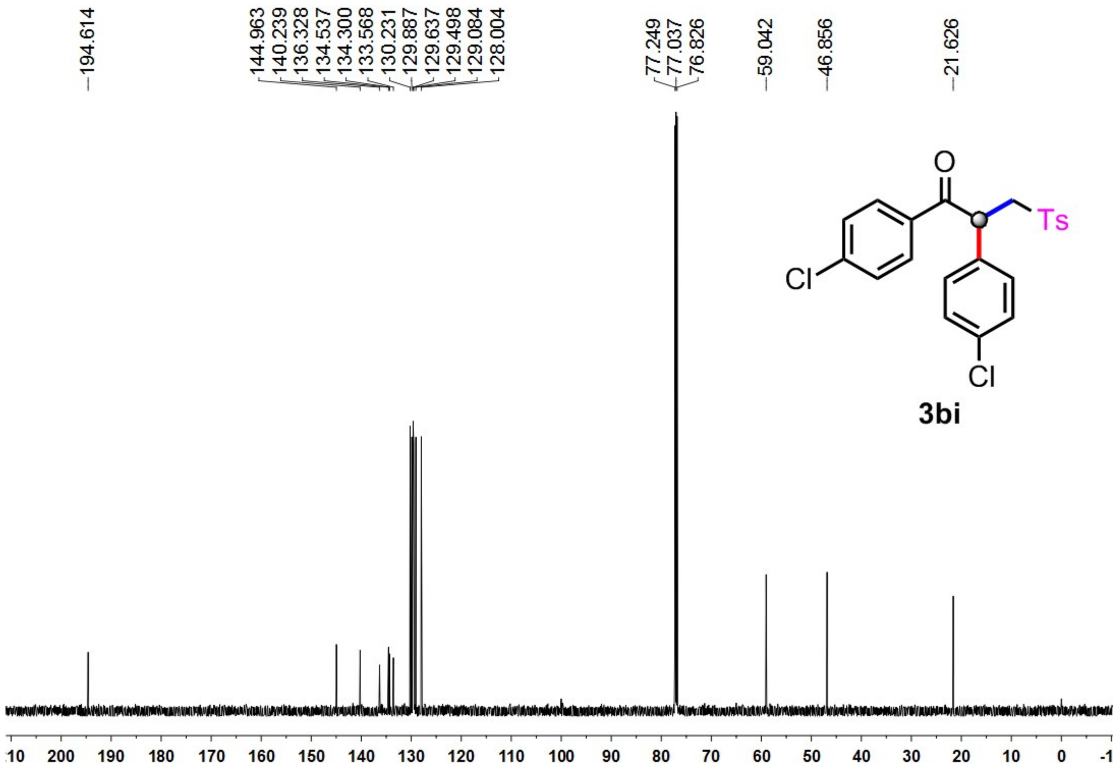

194.614
144.963
140.239
136.328
134.537
134.300
133.568
130.231
129.887
129.637
129.498
129.084
128.004
77.249
77.037
76.826
59.042
46.856
21.626
O
Ts
Cl
Cl
3bi
200 190 180 170 160 150 140 130 120 110 100 90 80 70 60 50 40 30 20 10 0

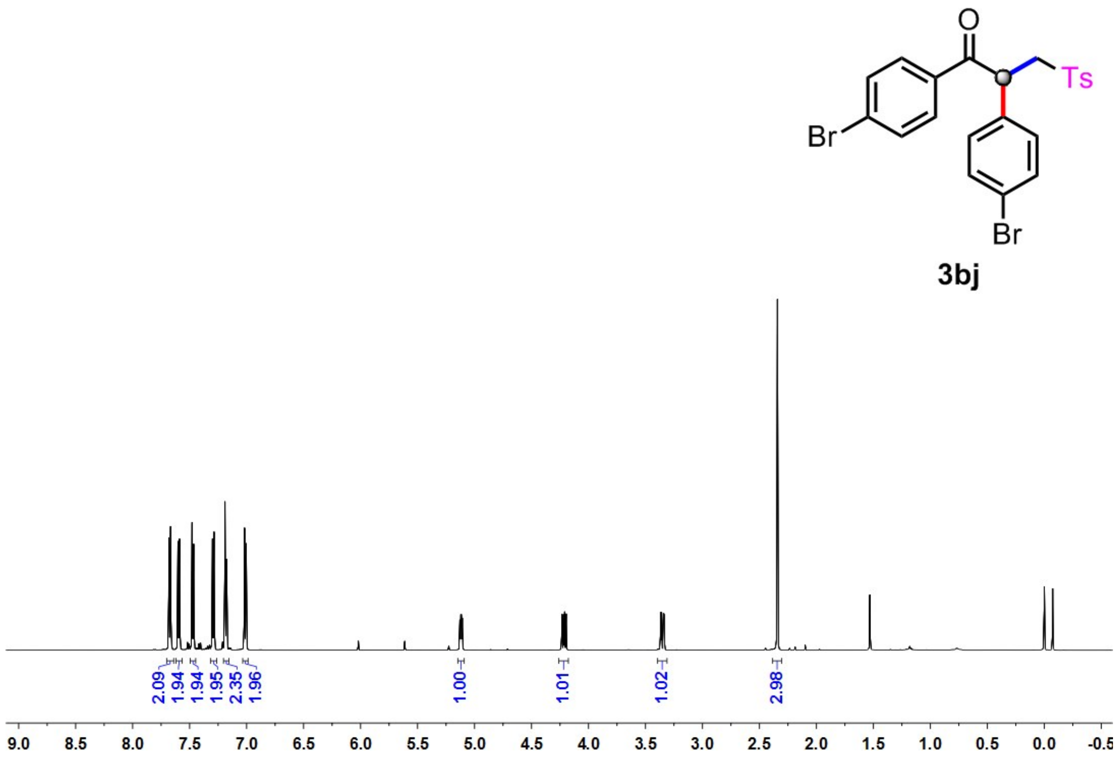
O
Ts
Br
Br
3bj
2.09
1.94
1.94
1.95
2.35
1.96
1.00
1.01
1.02
2.98
9.0 8.5 8.0 7.5 7.0 6.5 6.0 5.5 5.0 4.5 4.0 3.5 3.0 2.5 2.0 1.5 1.0 0.5 0.0 -0.5

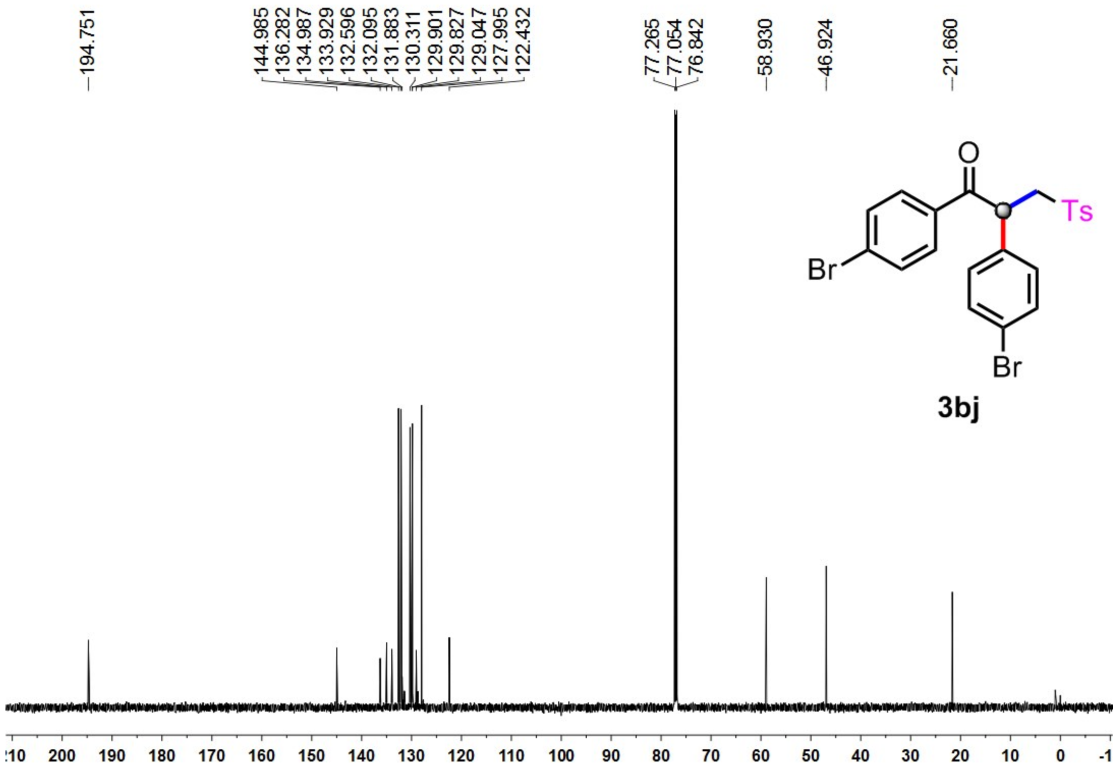
194.751
144.985
136.282
134.987
133.929
132.596
132.095
131.883
130.311
129.901
129.827
129.047
127.995
122.432
77.265
77.054
76.842
58.930
46.924
21.660
O
Ts
Br
Br
3bj
200 190 180 170 160 150 140 130 120 110 100 90 80 70 60 50 40 30 20 10 0

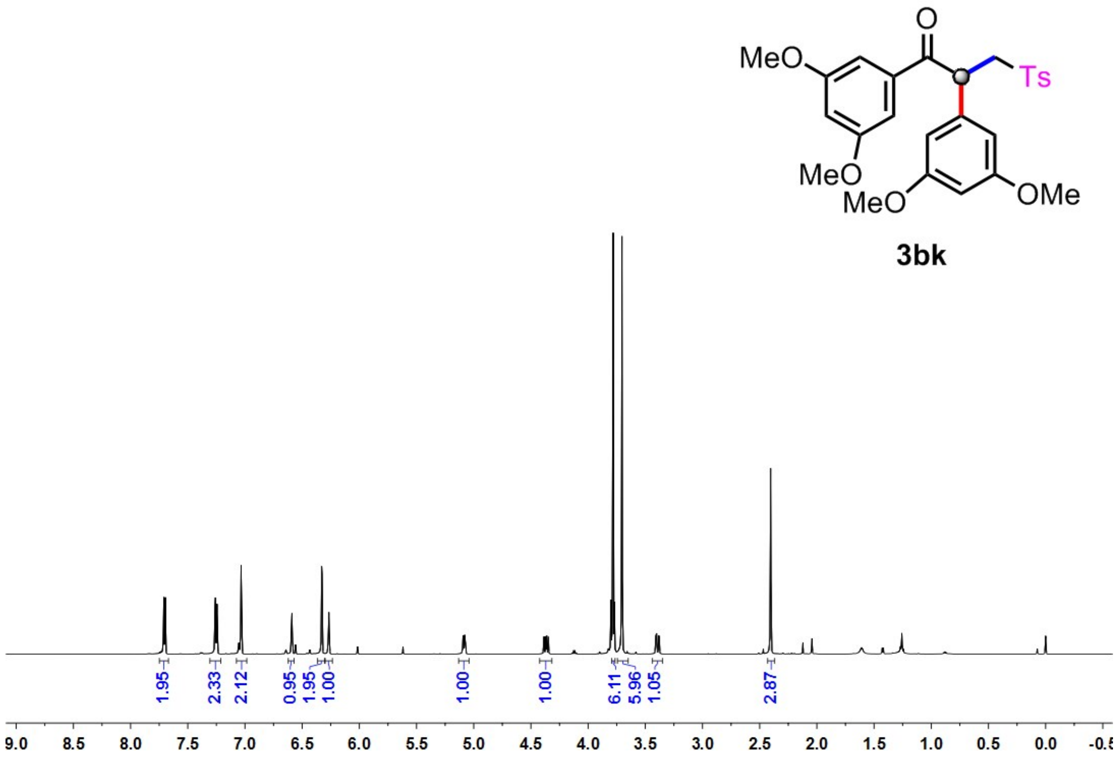
O
MeO
Ts
MeO
MeO
OMe
3bk
1.95
2.33
2.12
0.95
1.95
1.00
1.00
1.00
6.11
5.96
1.05
2.87
9.0 8.5 8.0 7.5 7.0 6.5 6.0 5.5 5.0 4.5 4.0 3.5 3.0 2.5 2.0 1.5 1.0 0.5 0.0 -0.5

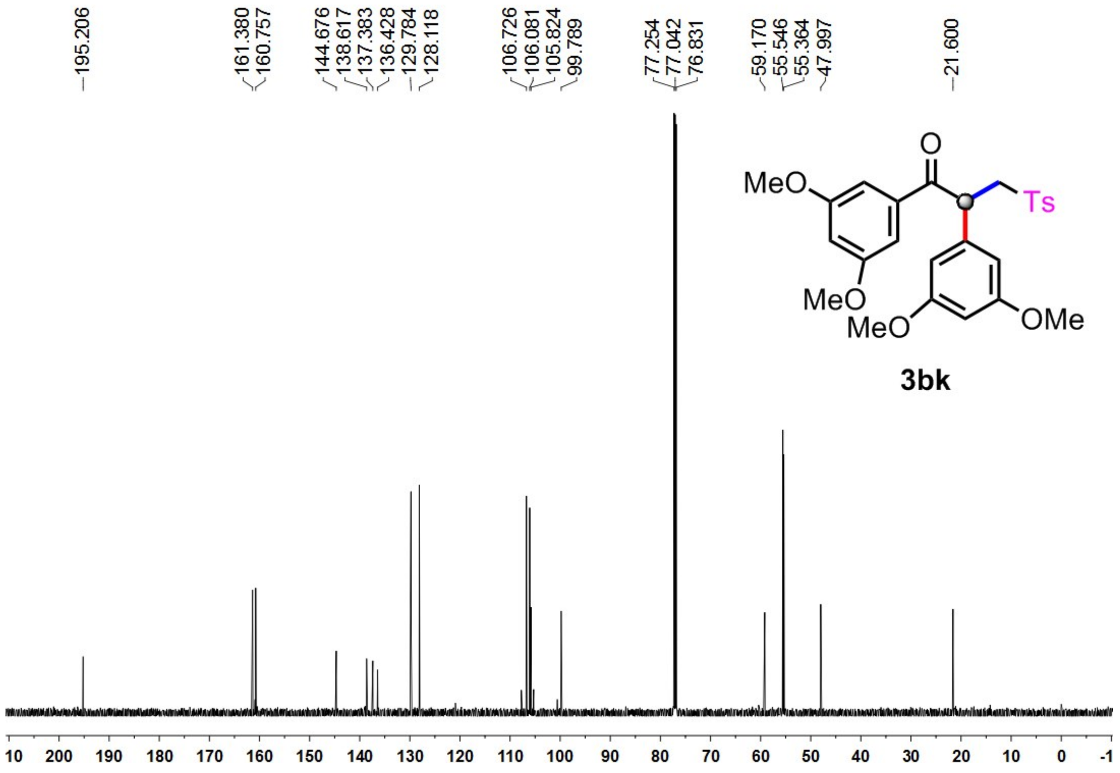
195.206
161.380
160.757
144.676
138.617
137.383
136.428
129.784
128.118
106.726
106.081
105.824
99.789
77.254
77.042
76.831
59.170
55.546
55.364
47.997
21.600
O
MeO
Ts
MeO
MeO
OMe
3bk
10 200 190 180 170 160 150 140 130 120 110 100 90 80 70 60 50 40 30 20 10 0 -1

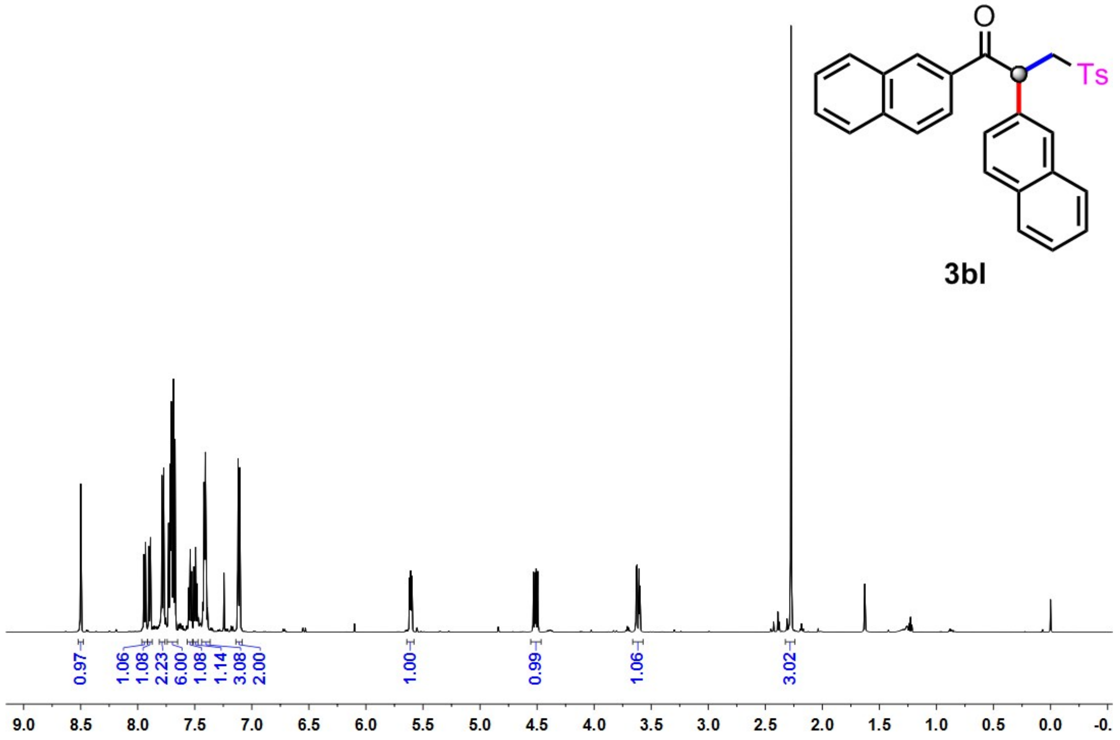
3bl
Ts
0.97
1.06
1.08
2.23
6.00
1.08
1.14
3.08
2.00
1.00
0.99
1.06
3.02
9.0 8.5 8.0 7.5 7.0 6.5 6.0 5.5 5.0 4.5 4.0 3.5 3.0 2.5 2.0 1.5 1.0 0.5 0.0

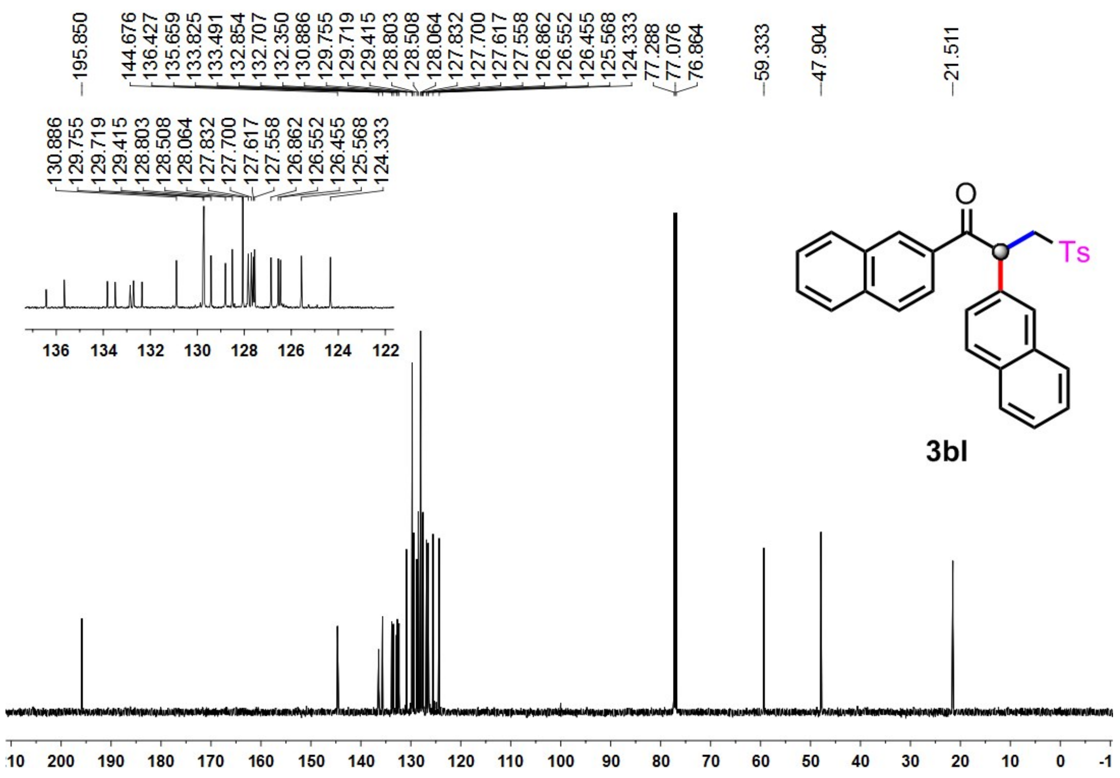
3bl
Ts
195.850
144.676
136.427
135.659
133.825
133.491
132.854
132.707
132.350
130.886
129.755
129.719
129.415
128.803
128.508
128.064
127.832
127.700
127.617
127.558
126.862
126.552
126.455
125.568
124.333
77.288
77.076
76.864
59.333
47.904
21.511
136 134 132 130 128 126 124 122
200 190 180 170 160 150 140 130 120 110 100 90 80 70 60 50 40 30 20 10 0

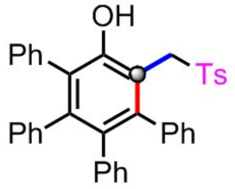
OH
Ph
Ts
Ph
Ph
Ph

3ca

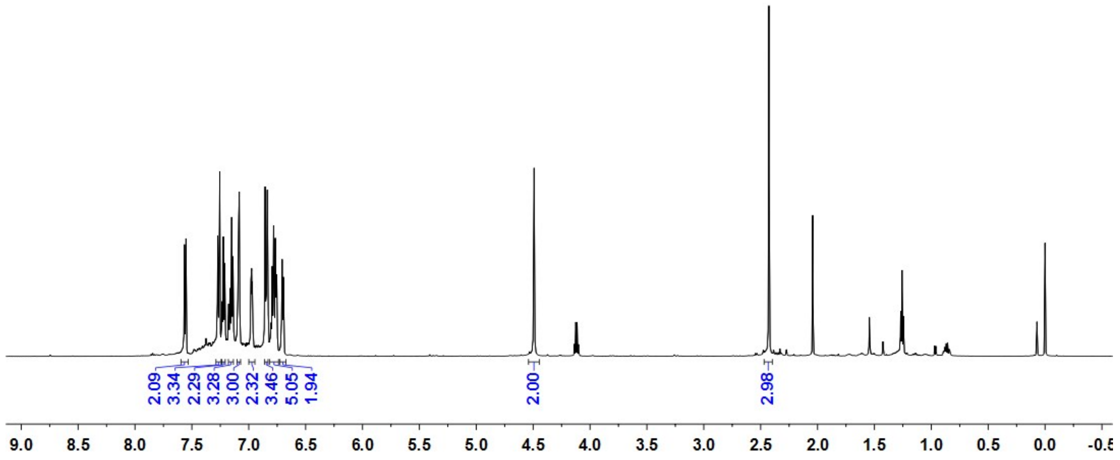
2.09
3.34
2.29
3.28
3.00
2.32
3.46
5.05
1.94
2.00
2.98
9.0 8.5 8.0 7.5 7.0 6.5 6.0 5.5 5.0 4.5 4.0 3.5 3.0 2.5 2.0 1.5 1.0 0.5 0.0 -0.5

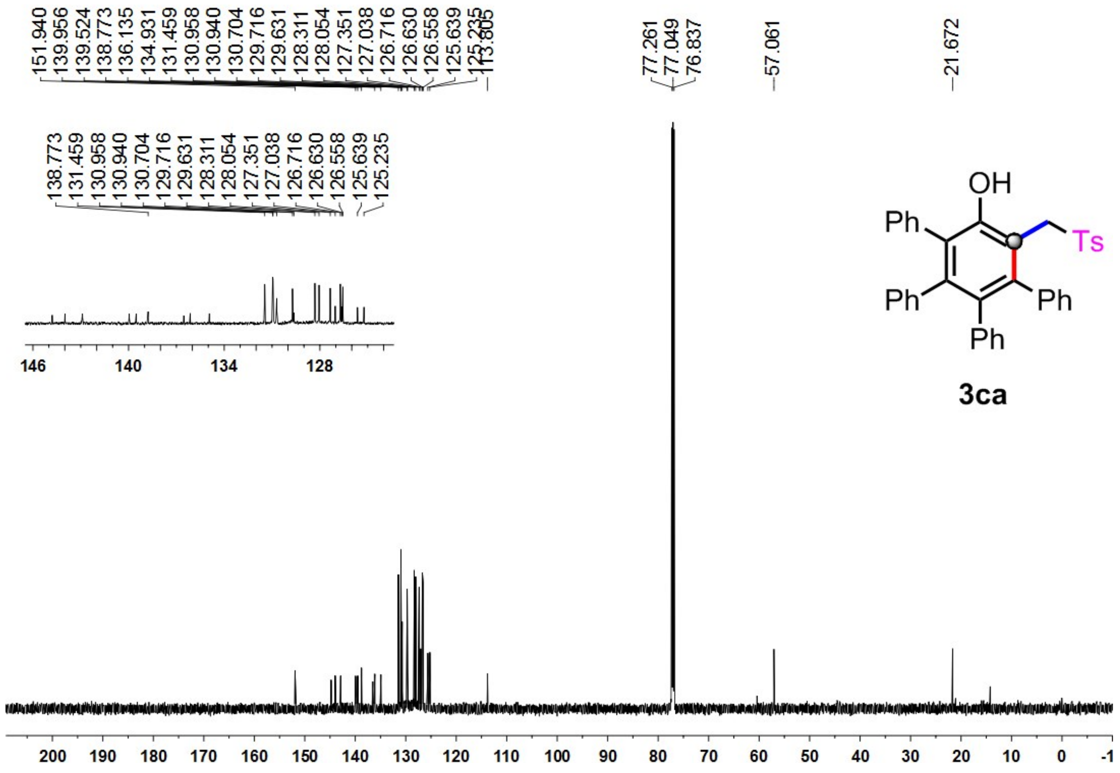
151.940
139.956
139.524
138.773
136.135
134.931
131.459
130.958
130.940
130.704
129.716
129.631
128.311
128.054
127.351
127.038
126.716
126.630
126.558
125.639
125.235
113.805
77.261
77.049
76.837
57.061
21.672
138.773
131.459
130.958
130.940
130.704
129.716
129.631
128.311
128.054
127.351
127.038
126.716
126.630
126.558
125.639
125.235
146 140 134 128
200 190 180 170 160 150 140 130 120 110 100 90 80 70 60 50 40 30 20 10 0 -1
OH
Ph
Ts
Ph
Ph
Ph

3ca

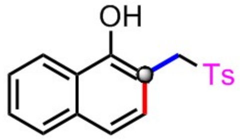
OH
Ts

3cb

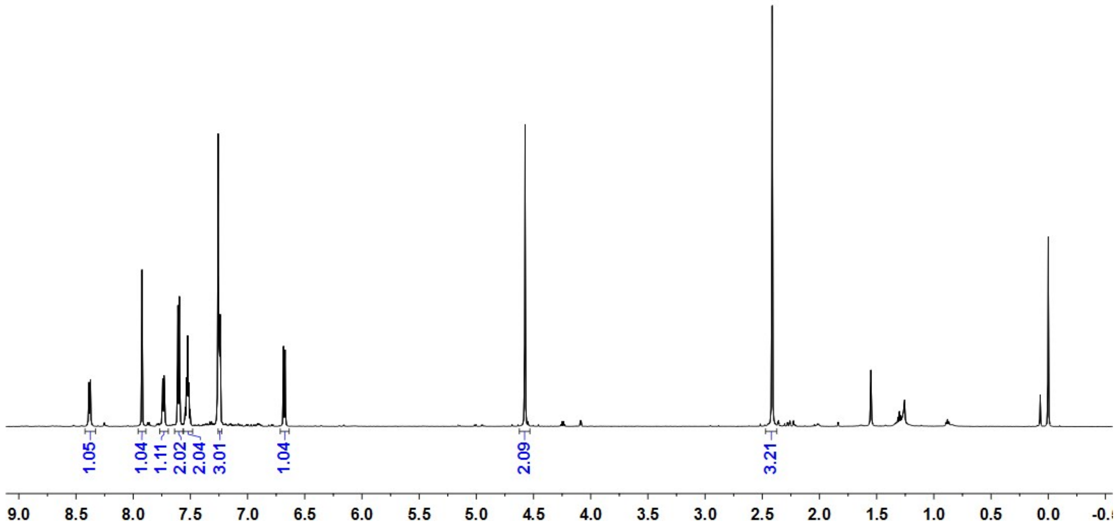
1.05
1.04
1.11
2.02
2.04
3.01
1.04
2.09
3.21
9.0 8.5 8.0 7.5 7.0 6.5 6.0 5.5 5.0 4.5 4.0 3.5 3.0 2.5 2.0 1.5 1.0 0.5 0.0

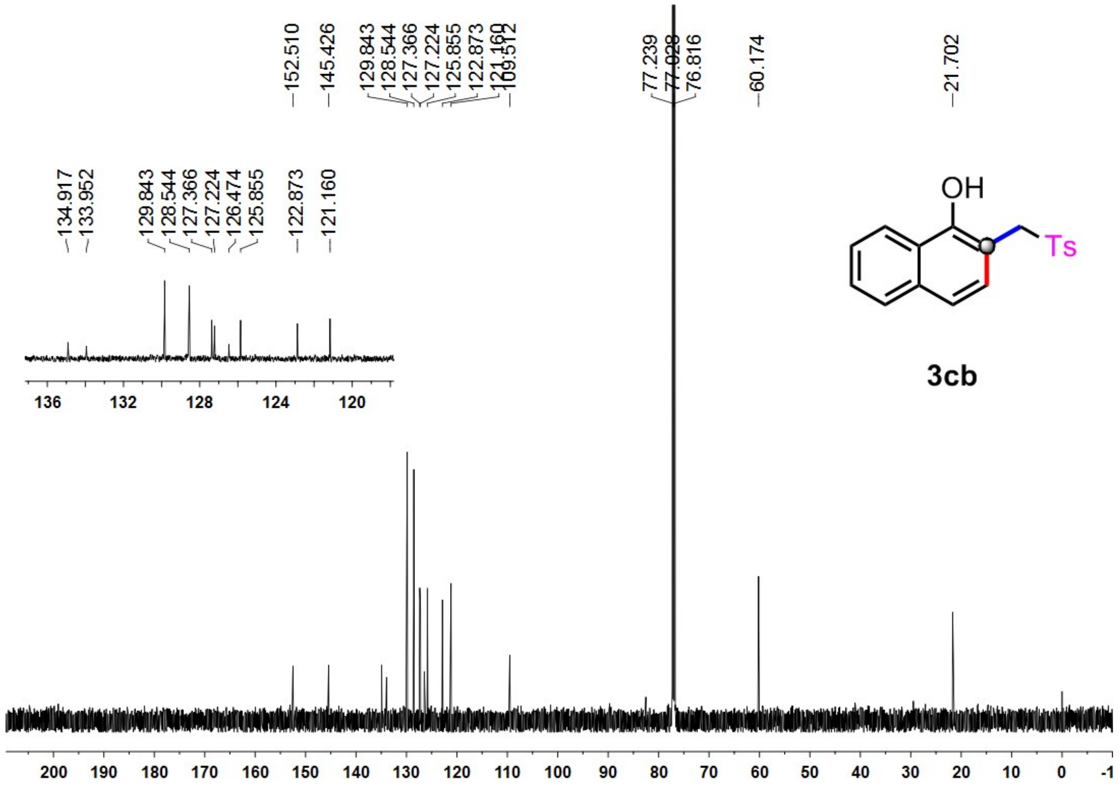
152.510
145.426
129.843
128.544
127.366
127.224
125.855
122.873
121.160
109.512
77.239
77.028
76.816
60.174
21.702
134.917
133.952
129.843
128.544
127.366
127.224
126.474
125.855
122.873
121.160
136 132 128 124 120
200 190 180 170 160 150 140 130 120 110 100 90 80 70 60 50 40 30 20 10 0

3cb

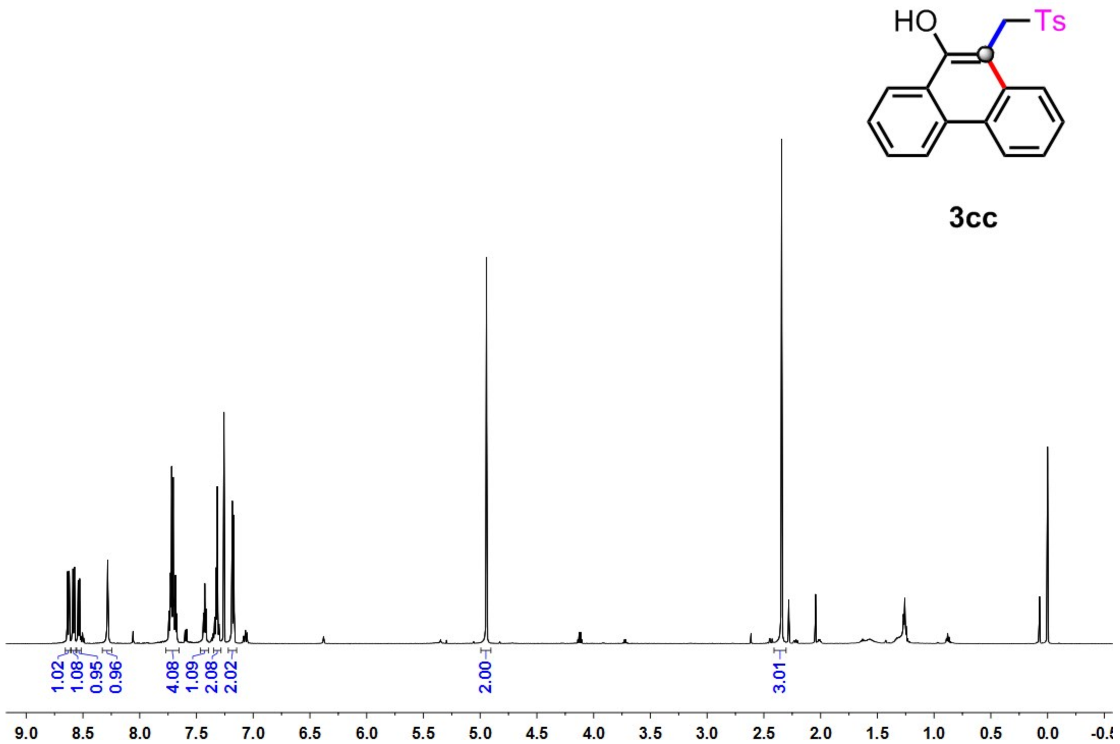
HO
Ts
3cc
1.02
1.08
0.95
0.96
4.08
1.09
2.08
2.02
2.00
3.01
9.0 8.5 8.0 7.5 7.0 6.5 6.0 5.5 5.0 4.5 4.0 3.5 3.0 2.5 2.0 1.5 1.0 0.5 0.0

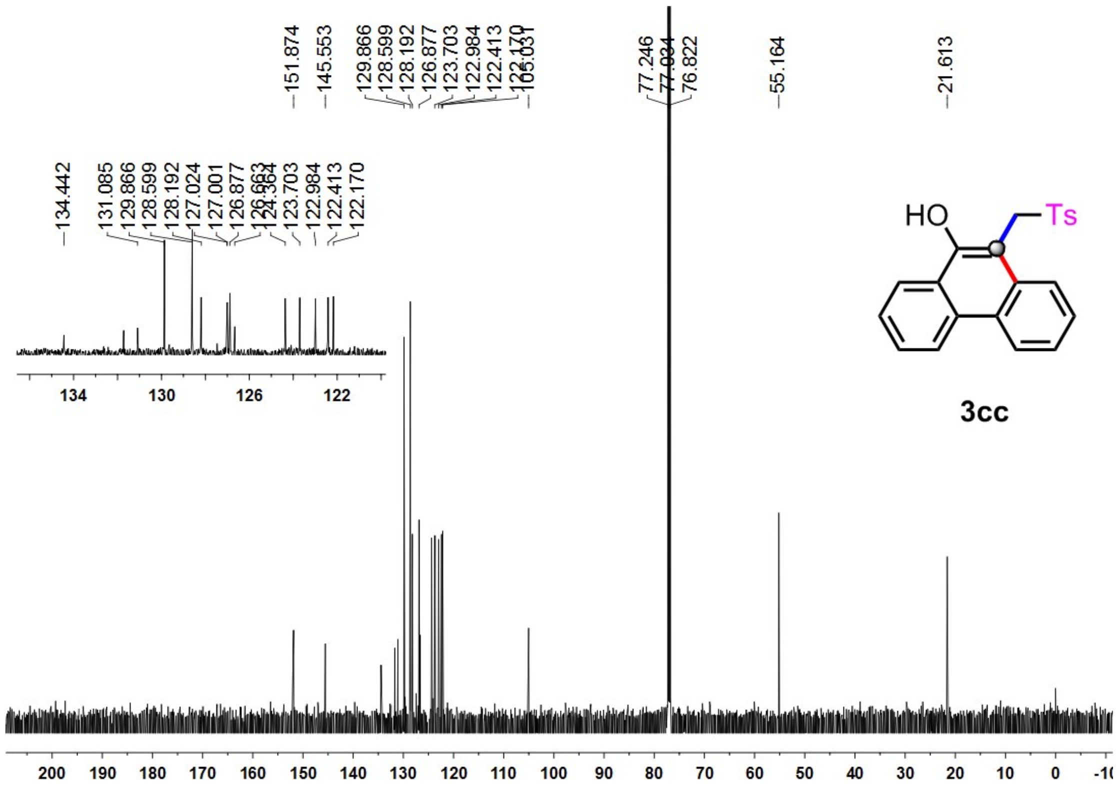
151.874
145.553
129.866
128.599
128.192
126.877
123.703
122.984
122.413
77.246
76.822
55.164
21.613
134.442
131.085
129.866
128.599
128.192
127.024
127.001
126.877
123.703
122.984
122.413
122.170
134 130 126 122
HO
Ts
3cc
200 190 180 170 160 150 140 130 120 110 100 90 80 70 60 50 40 30 20 10 0

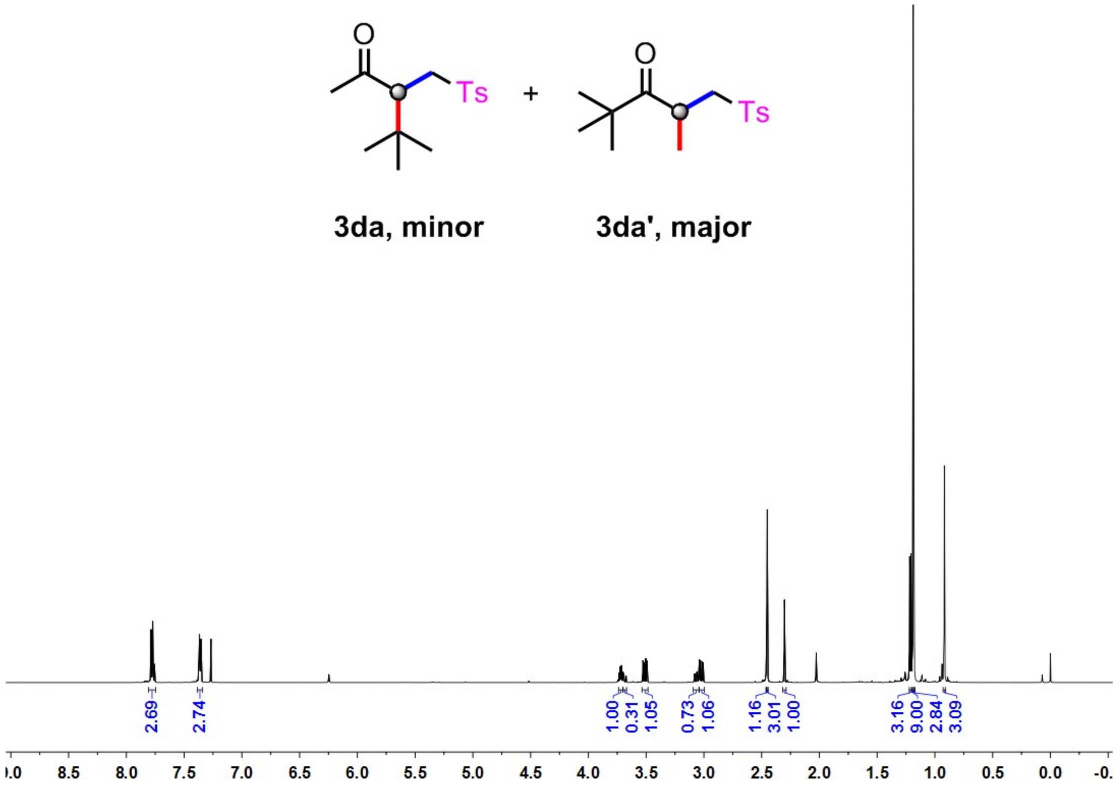

3da, minor
3da', major
2.69
2.74
1.00
0.31
1.05
0.73
1.06
1.16
3.01
1.00
3.16
9.00
2.84
3.09

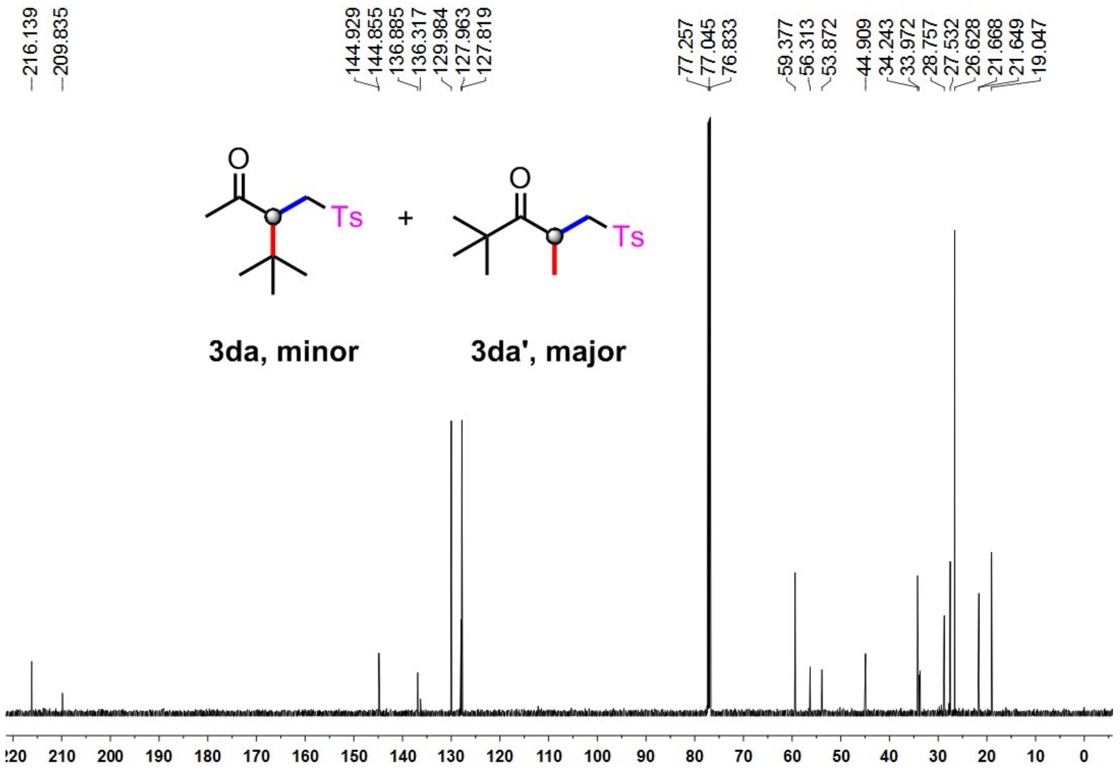

216.139
209.835
144.929
144.855
136.885
136.317
129.984
127.963
127.819
77.257
77.045
76.833
59.377
56.313
53.872
44.909
34.243
33.972
28.757
27.532
26.628
21.668
21.649
19.047
3da, minor
3da', major

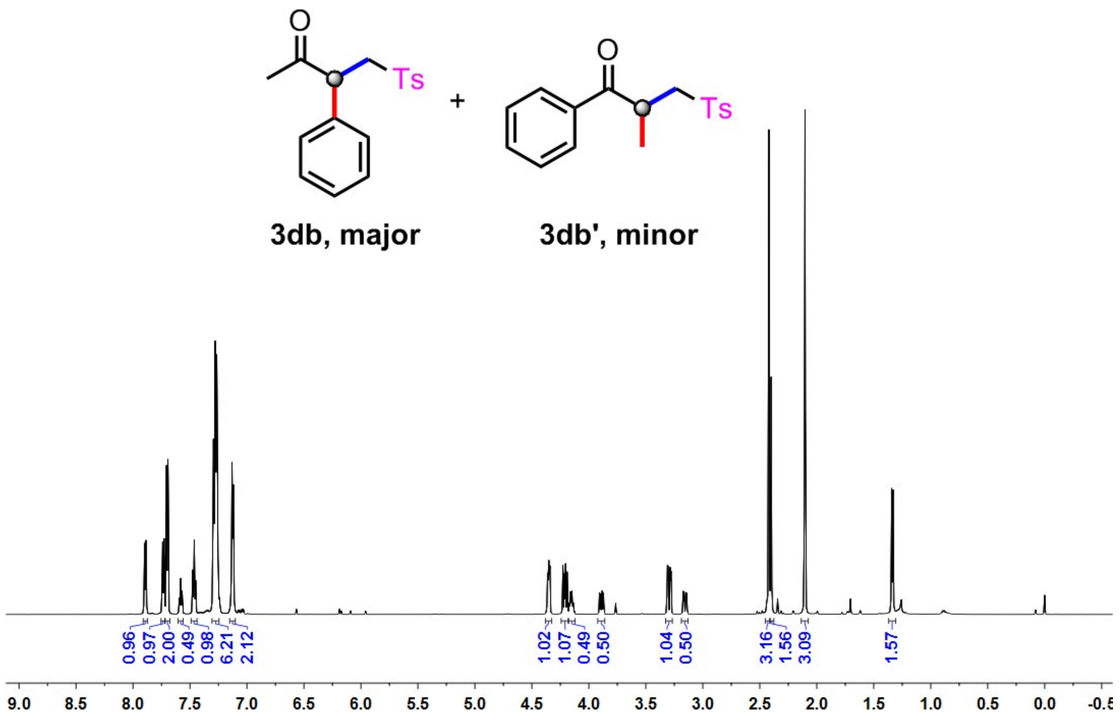
Ts
+
Ts
3db, major
3db', minor
0.96
0.97
2.00
0.49
0.98
6.21
2.12
1.02
1.07
0.49
0.50
1.04
0.50
3.16
1.56
3.09
1.57

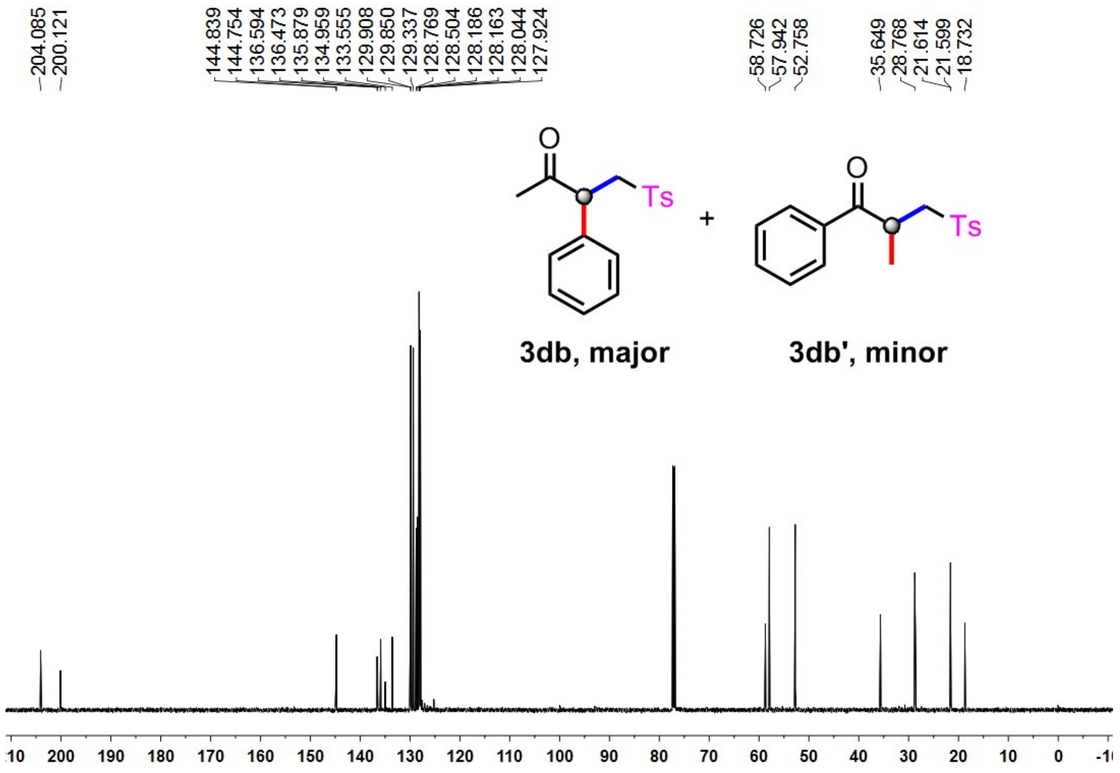
204.085
200.121
144.839
144.754
136.594
136.473
135.879
134.959
133.555
129.908
129.850
129.337
128.769
128.504
128.186
128.163
128.044
127.924
58.726
57.942
52.758
35.649
28.768
21.614
21.599
18.732
Ts
+
Ts
3db, major
3db', minor

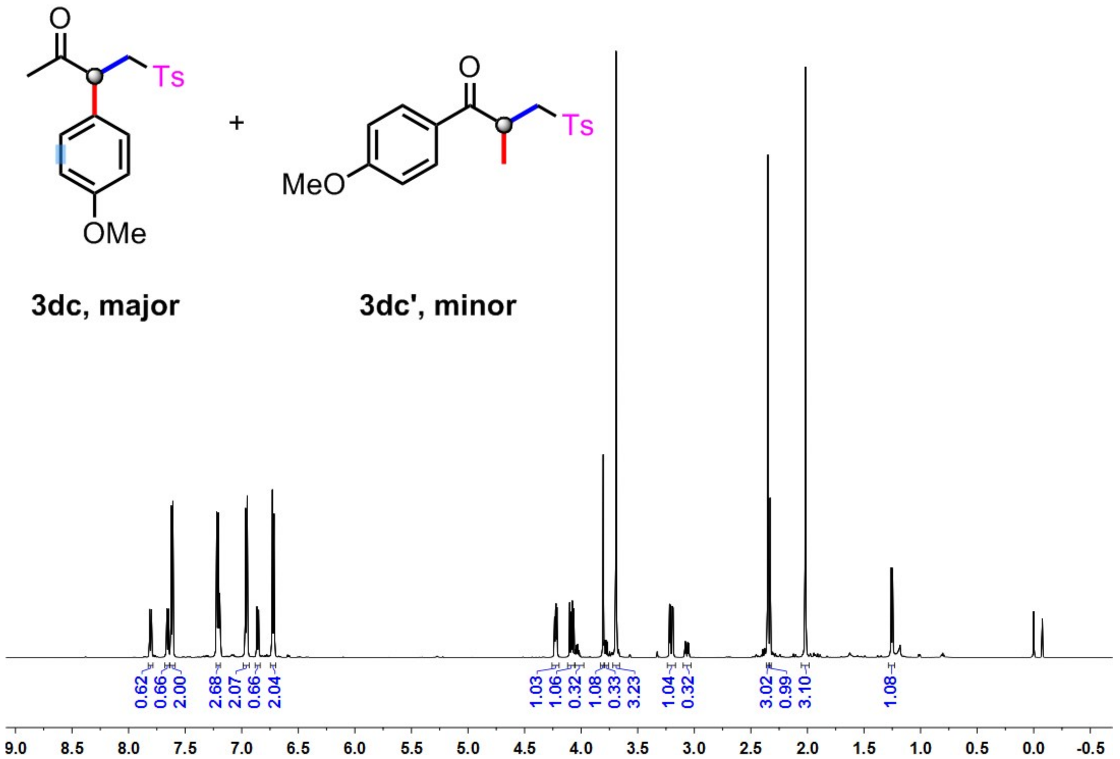
3dc, major
+
3dc', minor

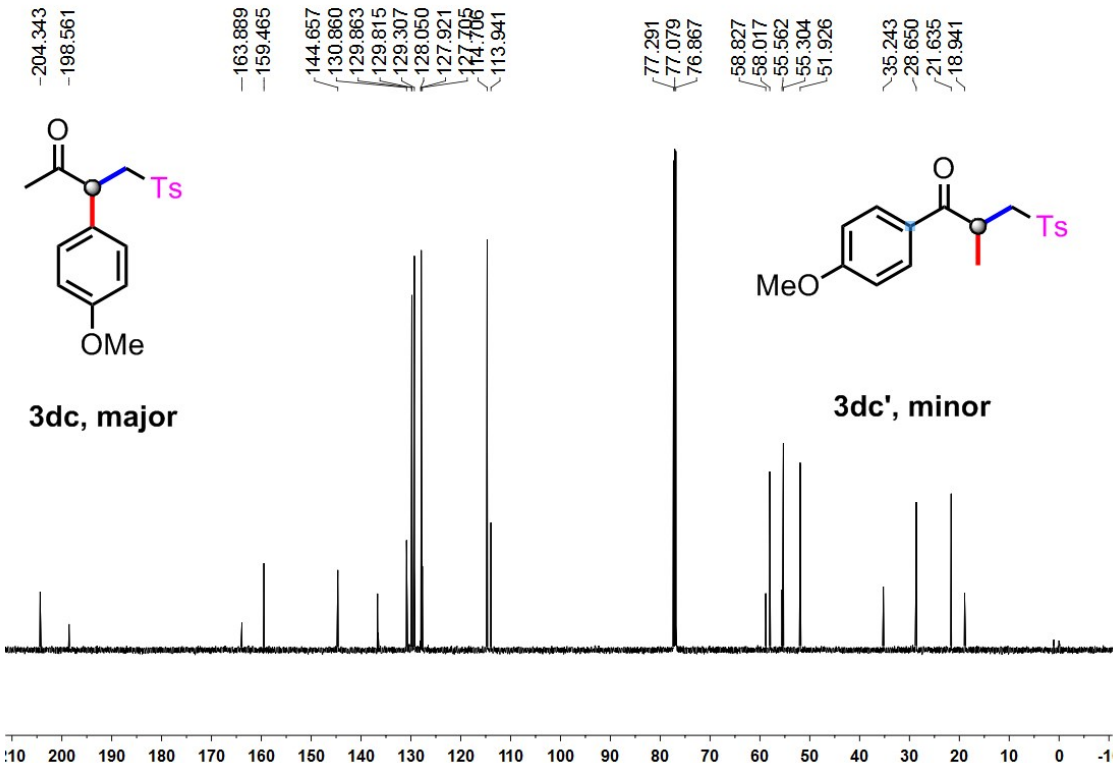
3dc, major
3dc', minor

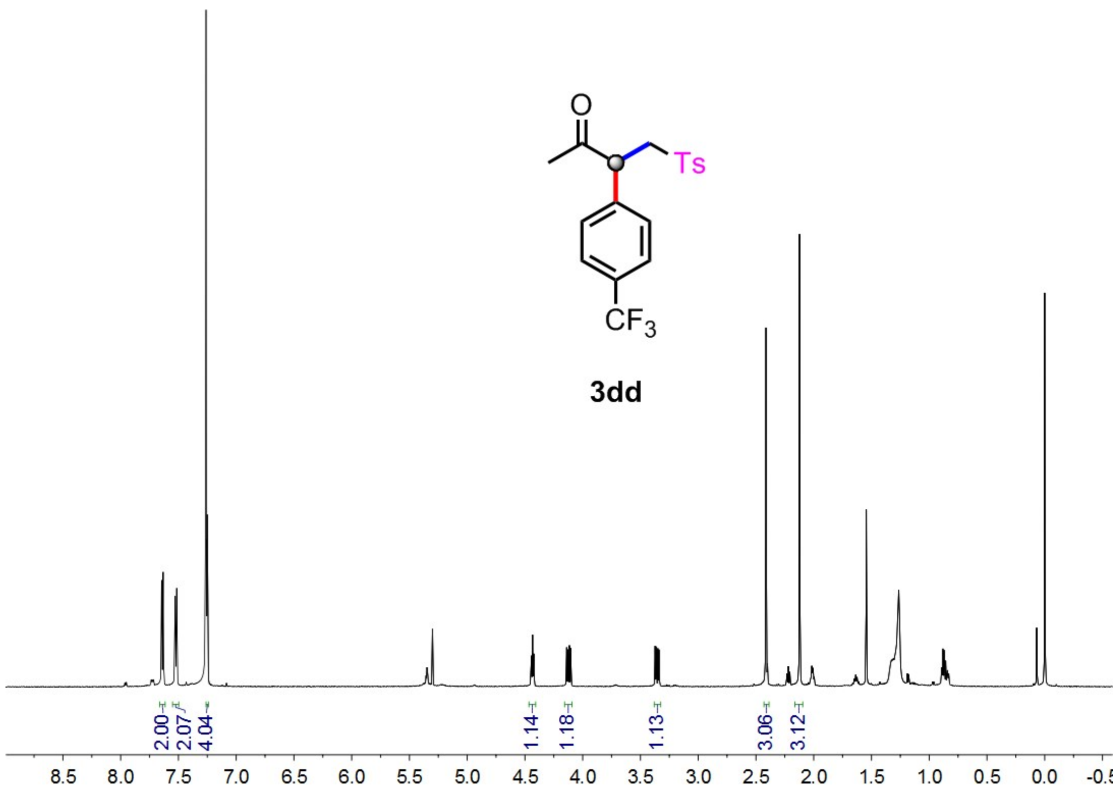
O
Ts
CF3
3dd
2.00
2.07
4.04
1.14
1.18
1.13
3.06
3.12
8.5 8.0 7.5 7.0 6.5 6.0 5.5 5.0 4.5 4.0 3.5 3.0 2.5 2.0 1.5 1.0 0.5 0.0

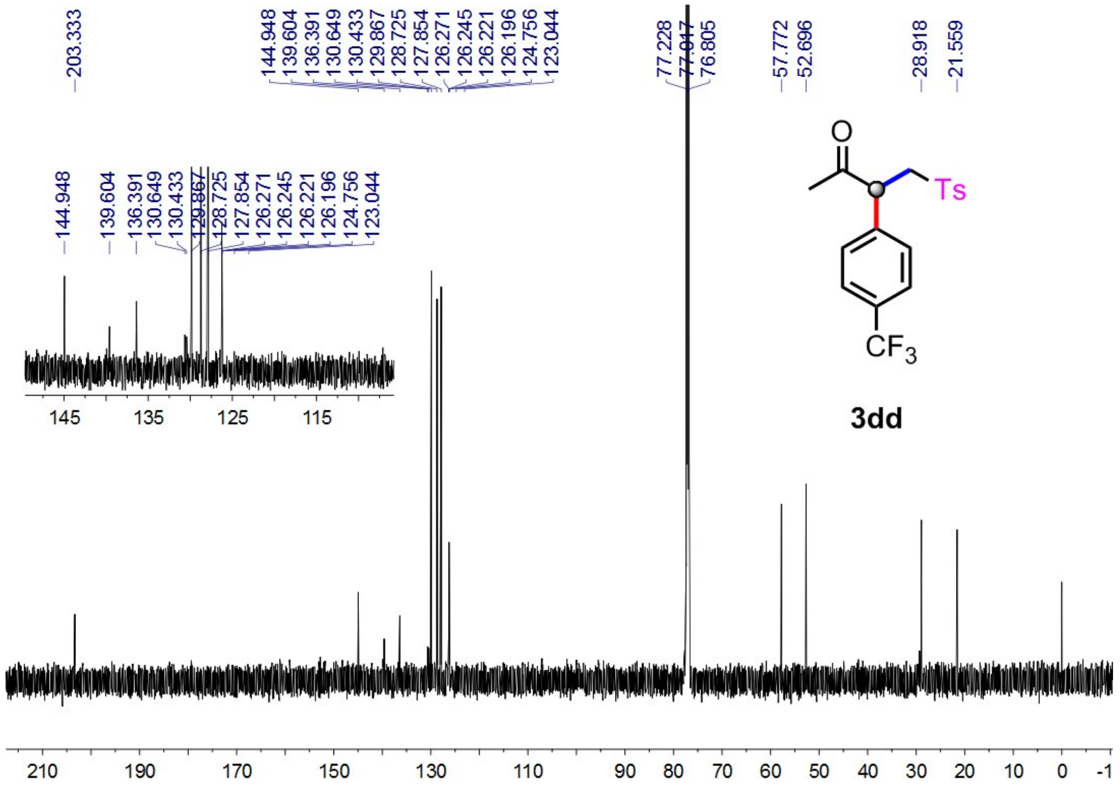
203.333
144.948
139.604
136.391
130.649
130.433
129.867
128.725
127.854
126.271
126.245
126.221
126.196
124.756
123.044
77.228
77.017
76.805
57.772
52.696
28.918
21.559
O
Ts
CF3
3dd
145 135 125 115
210 190 170 150 130 110 90 80 70 60 50 40 30 20 10 0

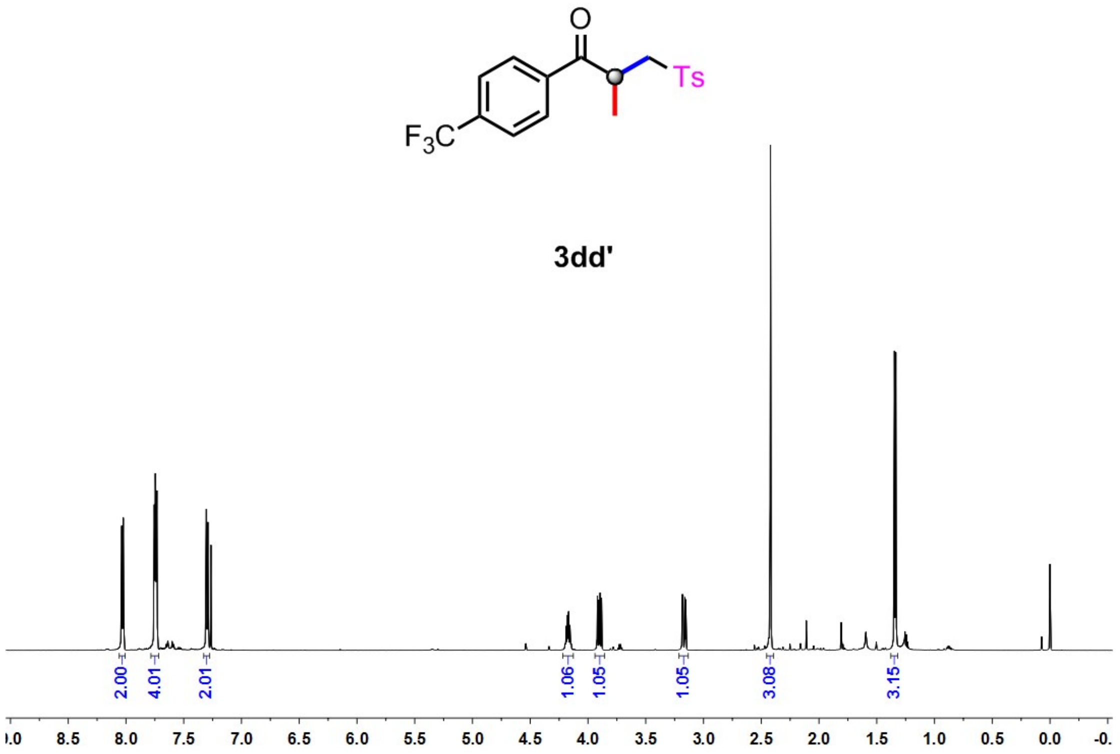
O
Ts
F3C
3dd'
2.00
4.01
2.01
1.06
1.05
1.05
3.08
3.15

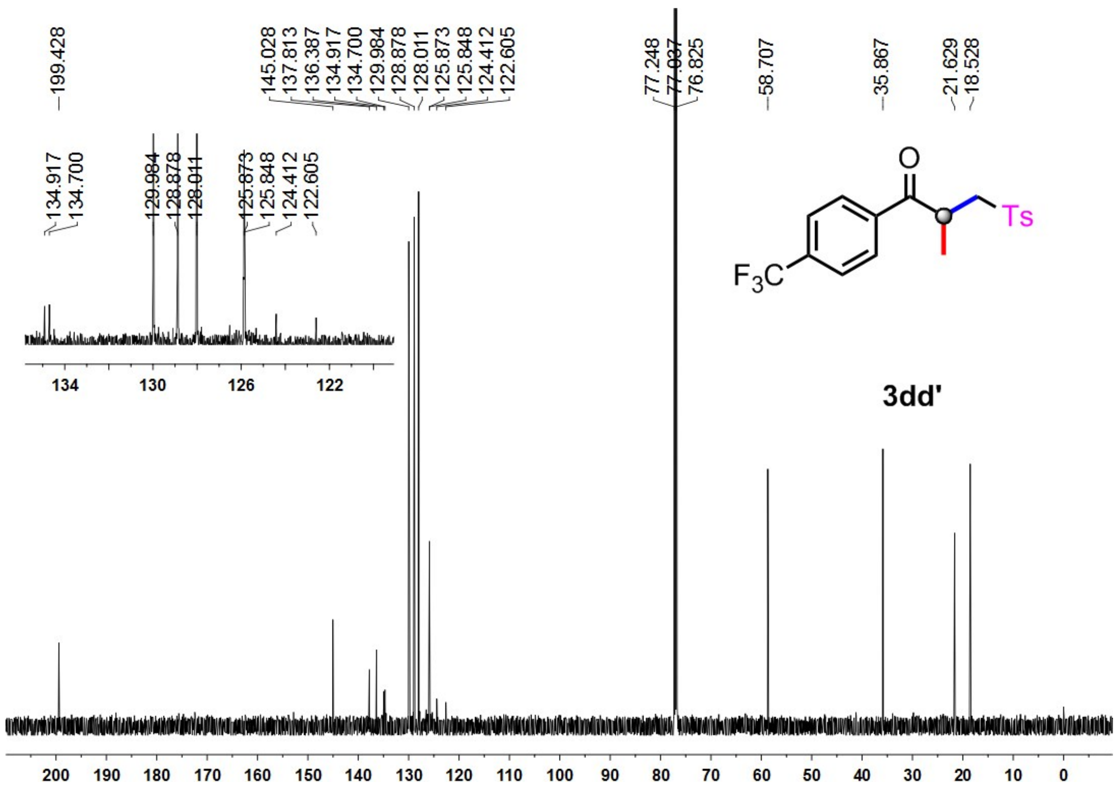
199.428
145.028
137.813
136.387
134.917
134.700
129.984
128.878
128.011
125.873
125.848
124.412
122.605
77.248
76.825
58.707
35.867
21.629
18.528
O
Ts
F3C
3dd'

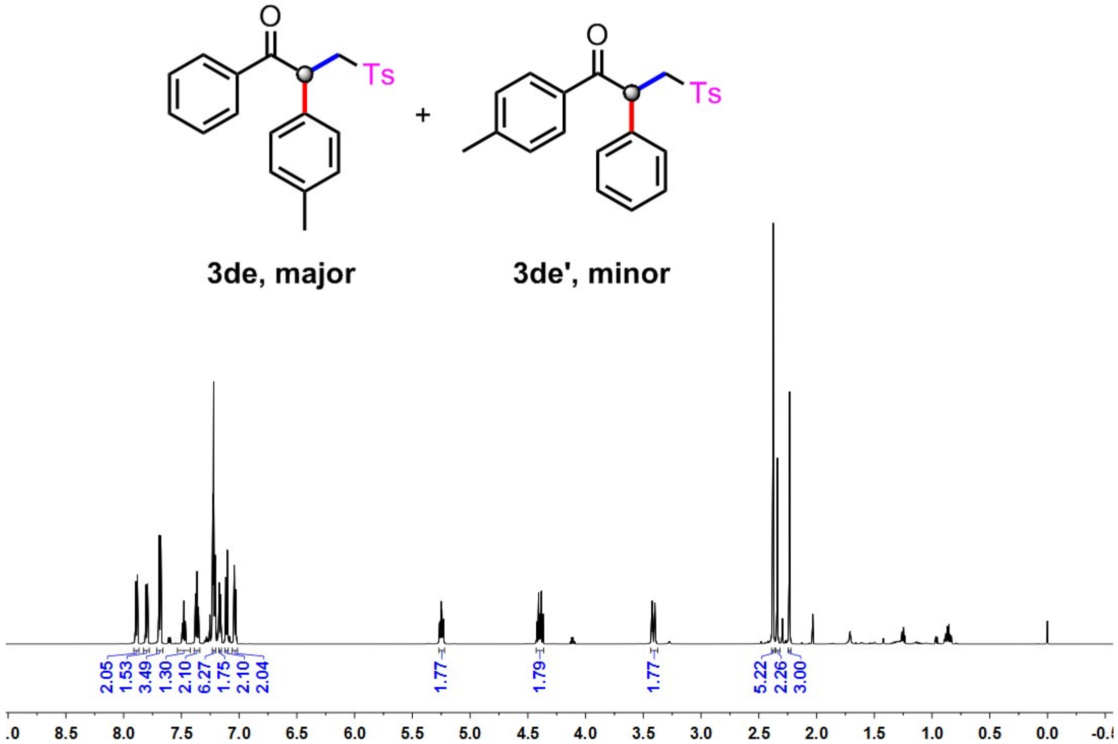
Ts
Ts
+
3de, major
3de', minor
2.05
1.53
3.49
1.30
2.10
6.27
1.75
2.10
2.04
1.77
1.79
1.77
5.22
2.26
3.00
8.5 8.0 7.5 7.0 6.5 6.0 5.5 5.0 4.5 4.0 3.5 3.0 2.5 2.0 1.5 1.0 0.5 0.0

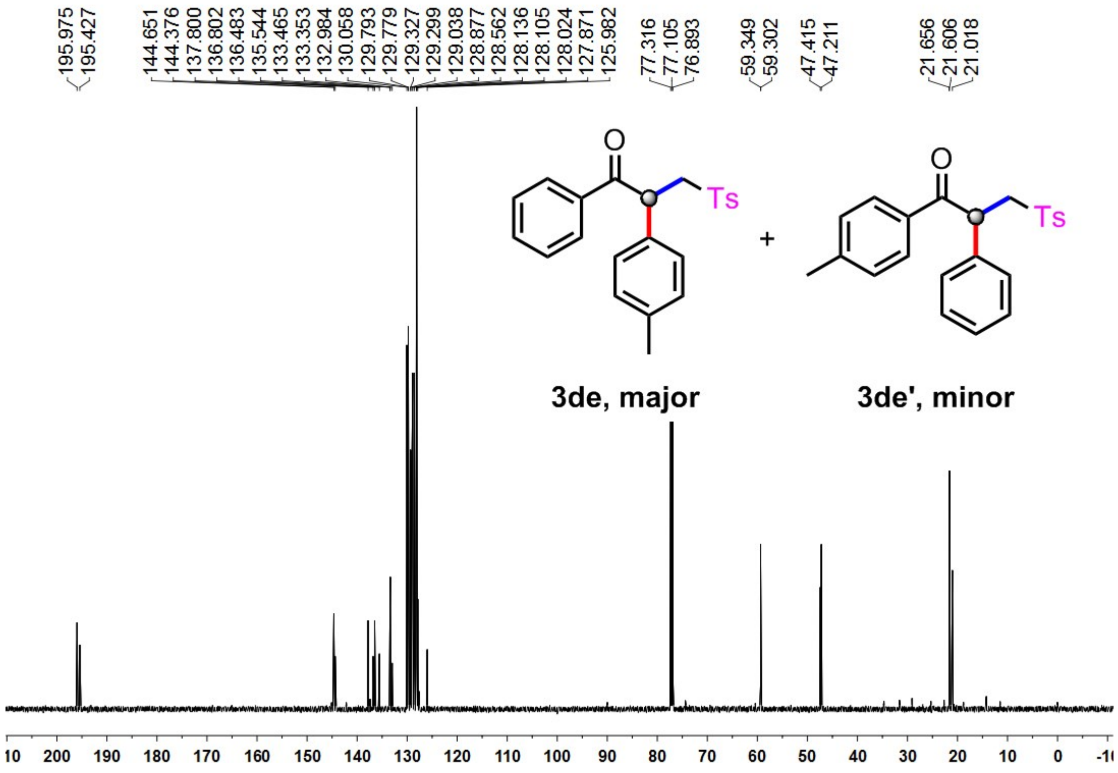
195.975
195.427
144.651
144.376
137.800
136.802
136.483
135.544
133.465
133.353
132.984
130.058
129.793
129.779
129.327
129.299
129.038
128.877
128.562
128.136
128.105
128.024
127.871
125.982
77.316
77.105
76.893
59.349
59.302
47.415
47.211
21.656
21.606
21.018
Ts
Ts
+
3de, major
3de', minor
200 190 180 170 160 150 140 130 120 110 100 90 80 70 60 50 40 30 20 10 0

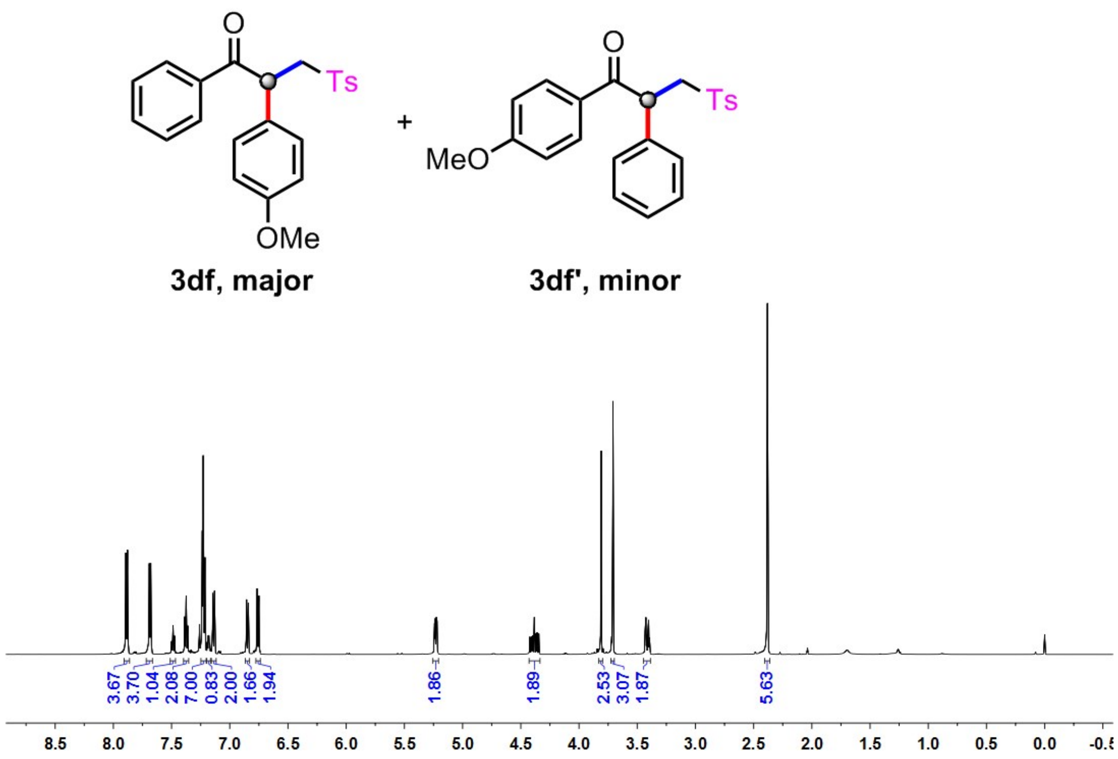
3df, major
3df', minor
Ts
MeO
OMe
3.67
3.70
1.04
2.08
7.00
0.83
2.00
1.66
1.94
1.86
1.89
2.53
3.07
1.87
5.63

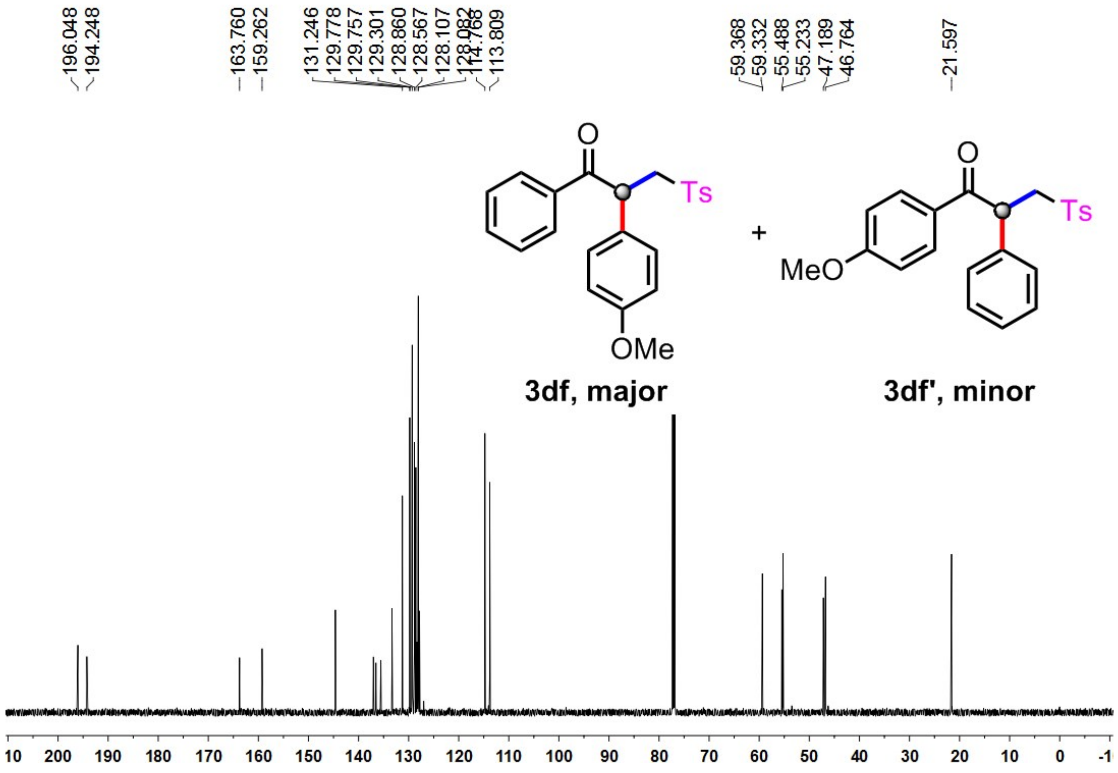
196.048
194.248
163.760
159.262
131.246
129.778
129.757
129.301
128.860
128.567
128.107
113.809
59.368
59.332
55.488
55.233
47.189
46.764
21.597
3df, major
3df', minor

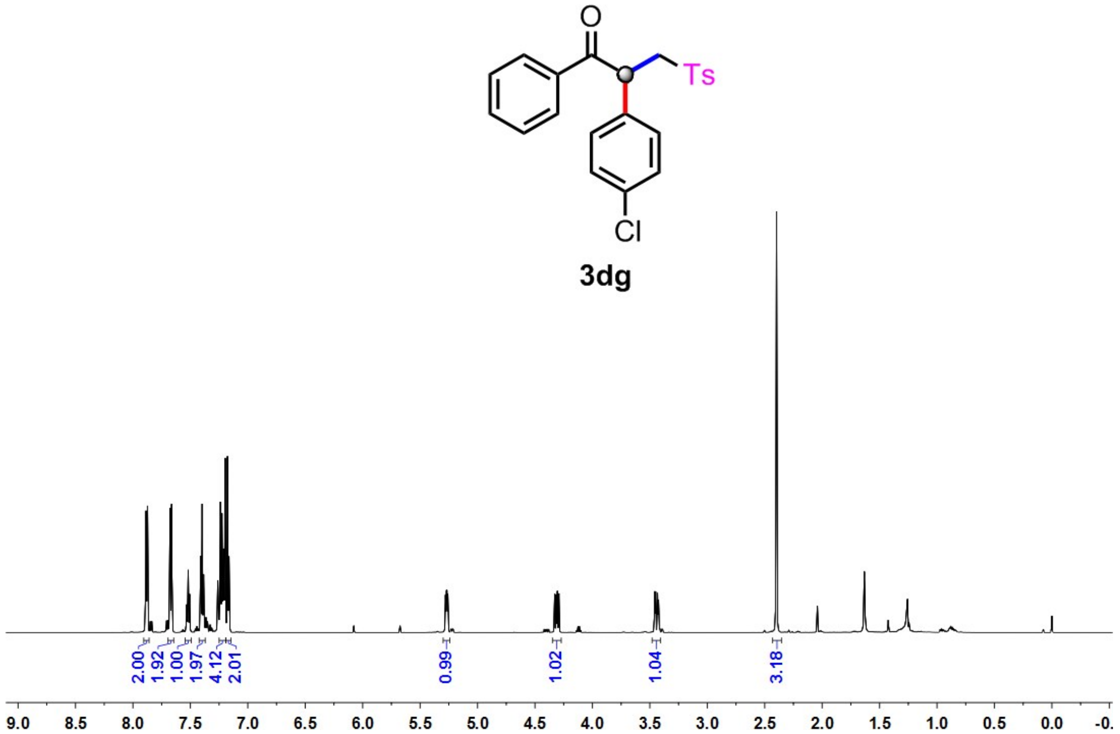
O
Ts
Cl
3dg
2.00
1.92
1.00
1.97
4.12
2.01
0.99
1.02
1.04
3.18
9.0
8.5
8.0
7.5
7.0
6.5
6.0
5.5
5.0
4.5
4.0
3.5
3.0
2.5
2.0
1.5
1.0
0.5
0.0

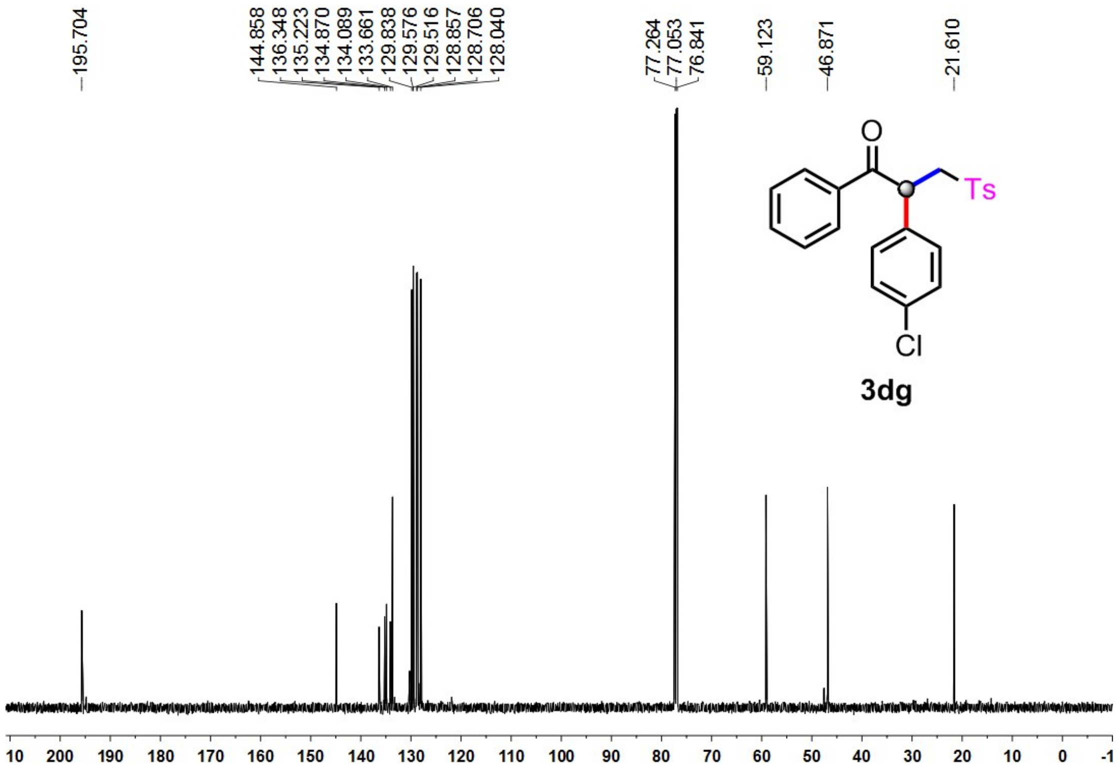
195.704
144.858
136.348
135.223
134.870
134.089
133.661
129.838
129.576
129.516
128.857
128.706
128.040
77.264
77.053
76.841
59.123
46.871
21.610
O
Ts
Cl
3dg
200
190
180
170
160
150
140
130
120
110
100
90
80
70
60
50
40
30
20
10
0

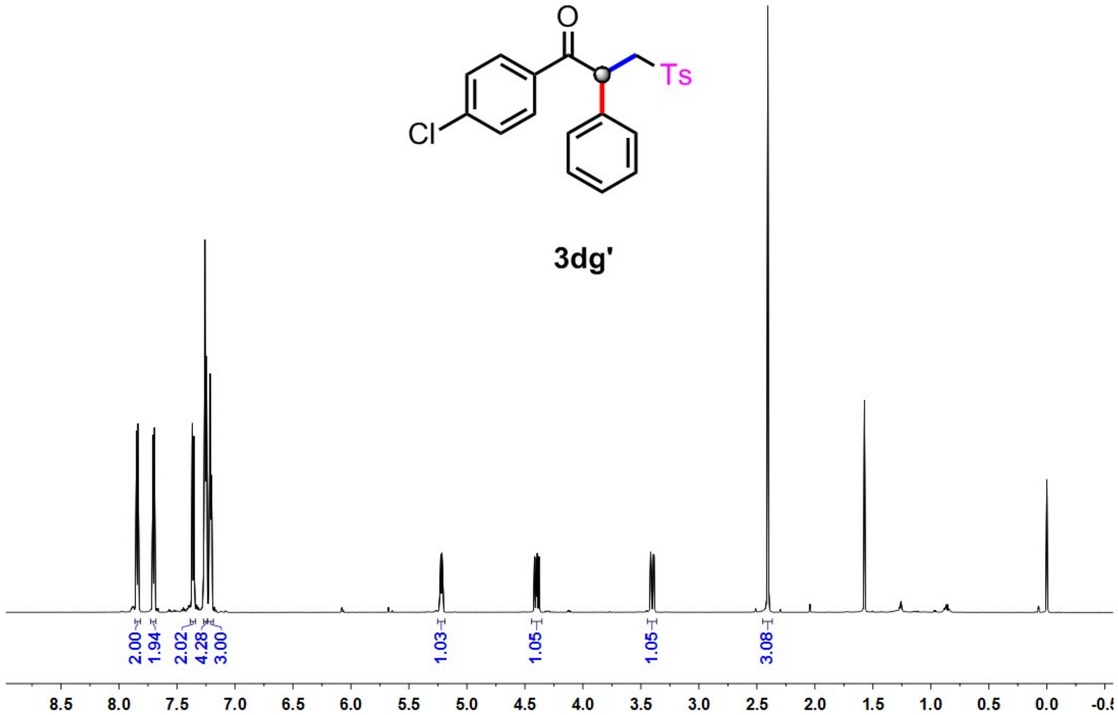

O
Ts
Cl
3dg'
2.00
1.94
2.02
4.28
3.00
1.03
1.05
1.05
3.08
8.5 8.0 7.5 7.0 6.5 6.0 5.5 5.0 4.5 4.0 3.5 3.0 2.5 2.0 1.5 1.0 0.5 0.0

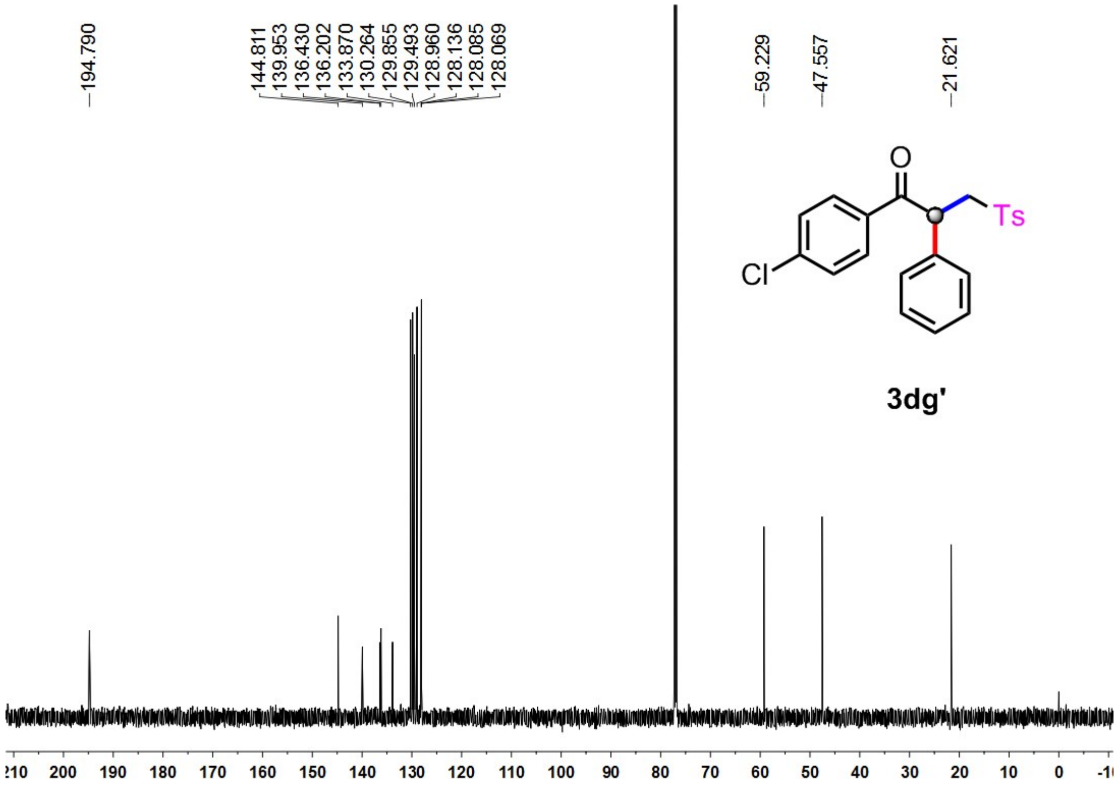

194.790
144.811
139.953
136.430
136.202
133.870
130.264
129.855
129.493
128.960
128.136
128.085
128.069
59.229
47.557
21.621
O
Ts
Cl
3dg'
200 190 180 170 160 150 140 130 120 110 100 90 80 70 60 50 40 30 20 10 0

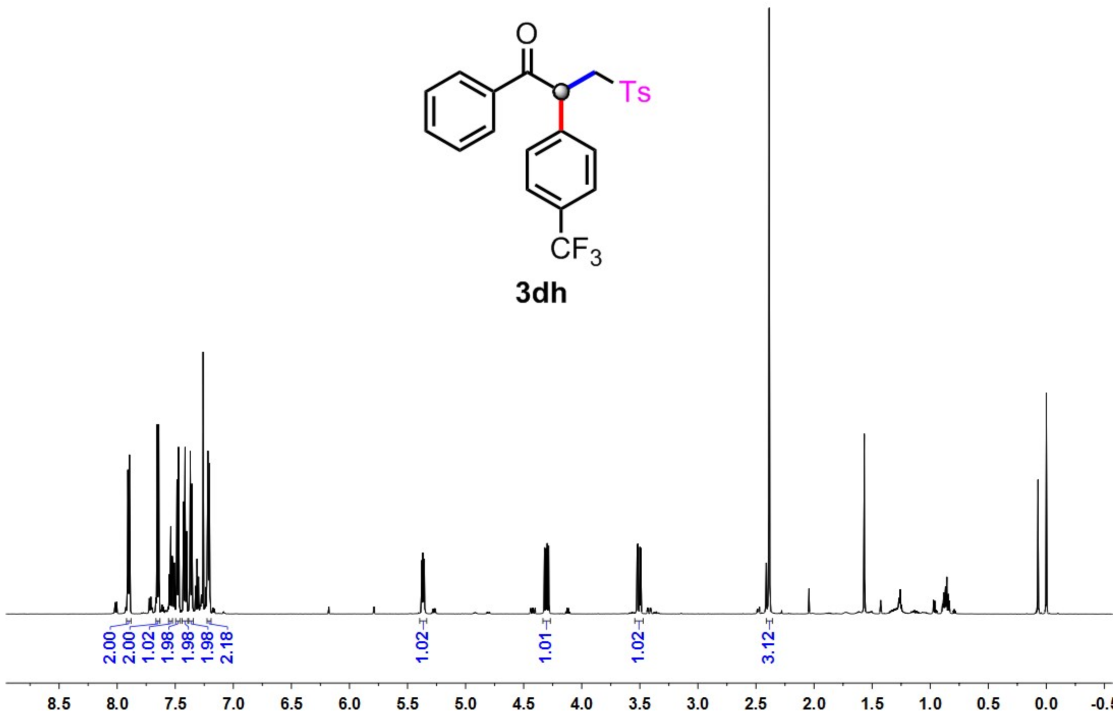

O
Ts
CF3
3dh
2.00
2.00
1.02
1.98
1.98
1.98
2.18
1.02
1.01
1.02
3.12
8.5 8.0 7.5 7.0 6.5 6.0 5.5 5.0 4.5 4.0 3.5 3.0 2.5 2.0 1.5 1.0 0.5 0.0

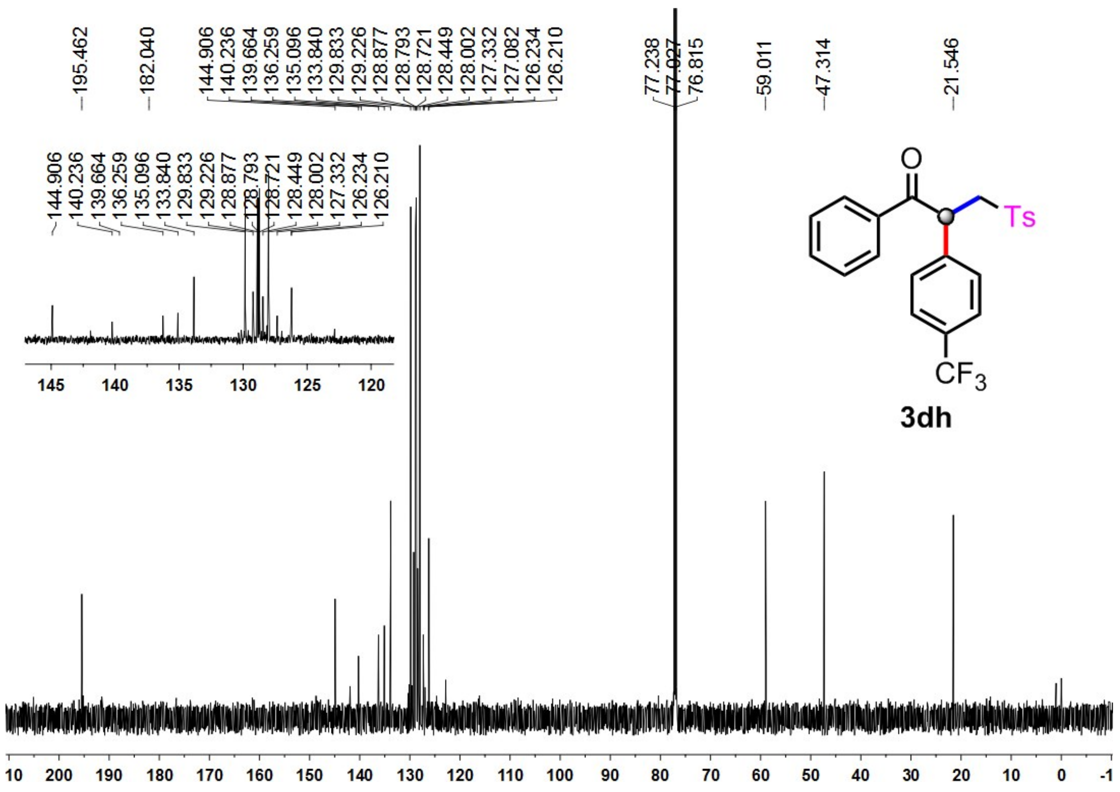

195.462
182.040
144.906
140.236
139.664
136.259
135.096
133.840
129.833
129.226
128.877
128.793
128.721
128.449
128.002
127.332
127.082
126.234
126.210
77.238
77.027
76.815
59.011
47.314
21.546
O
Ts
CF3
3dh
145 140 135 130 125 120
200 190 180 170 160 150 140 130 120 110 100 90 80 70 60 50 40 30 20 10 0

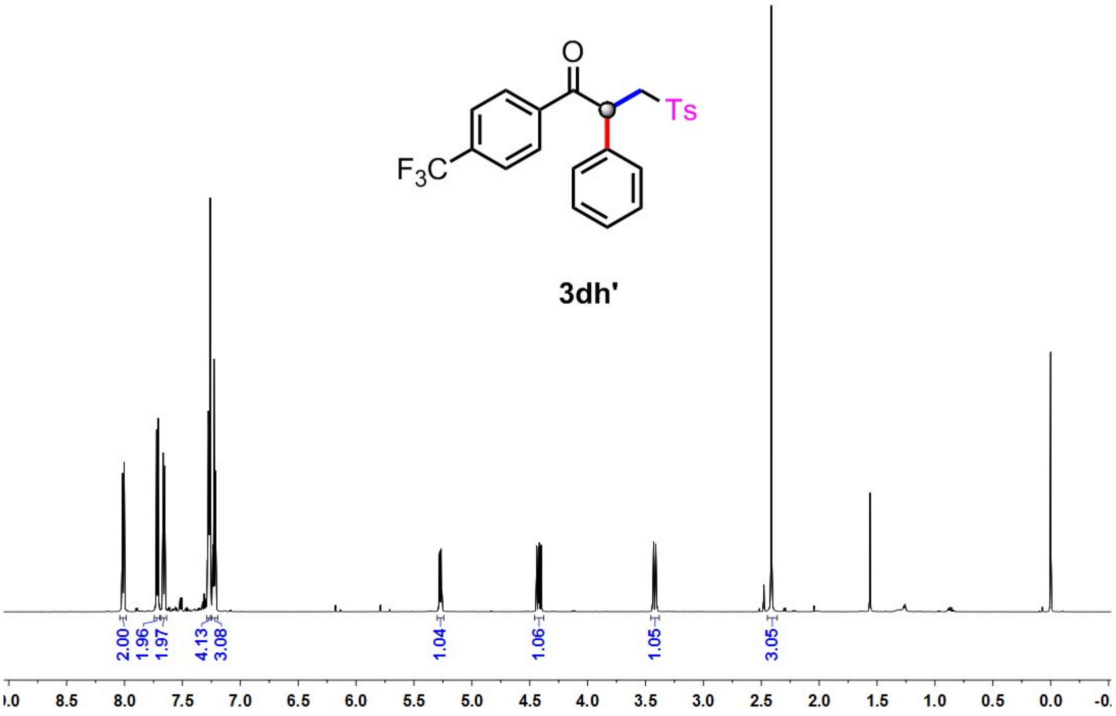
O
Ts
F3C
3dh'
2.00
1.96
1.97
4.13
3.08
1.04
1.06
1.05
3.05
8.5
8.0
7.5
7.0
6.5
6.0
5.5
5.0
4.5
4.0
3.5
3.0
2.5
2.0
1.5
1.0
0.5
0.0

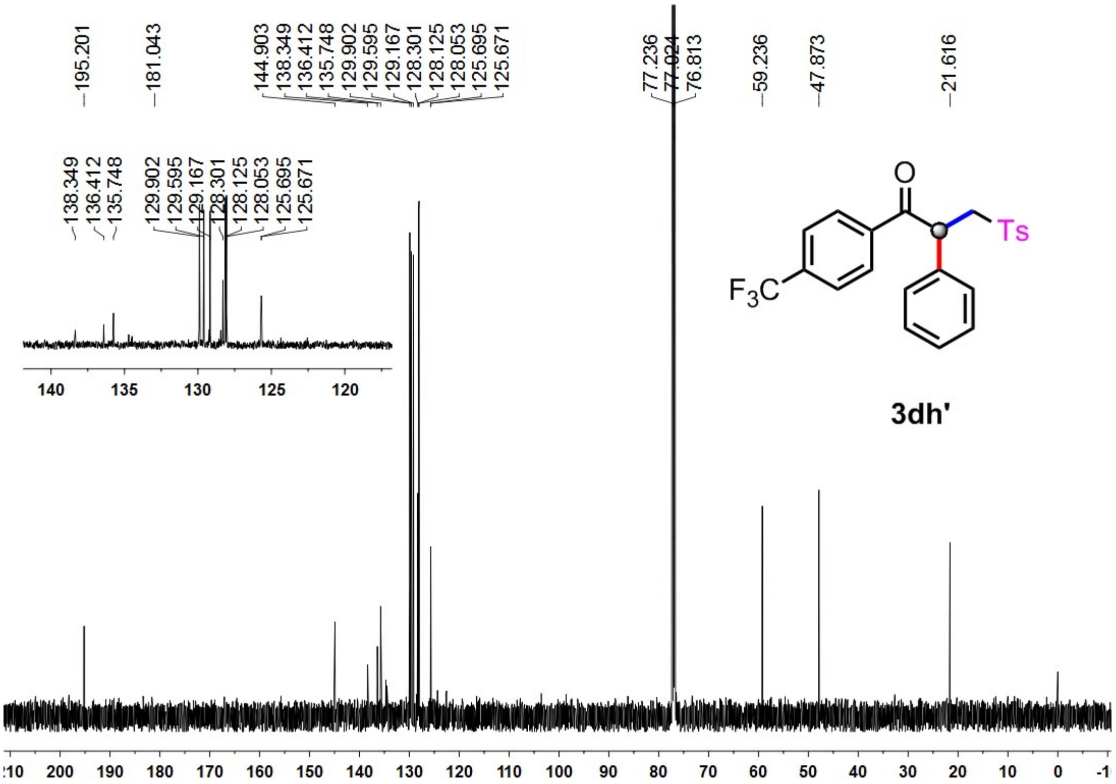
195.201
181.043
144.903
138.349
136.412
135.748
129.902
129.595
129.167
128.301
128.125
128.053
125.695
125.671
77.236
77.024
76.813
59.236
47.873
21.616
O
Ts
F3C
3dh'
140
135
130
125
120
200
190
180
170
160
150
140
130
120
110
100
90
80
70
60
50
40
30
20
10
0

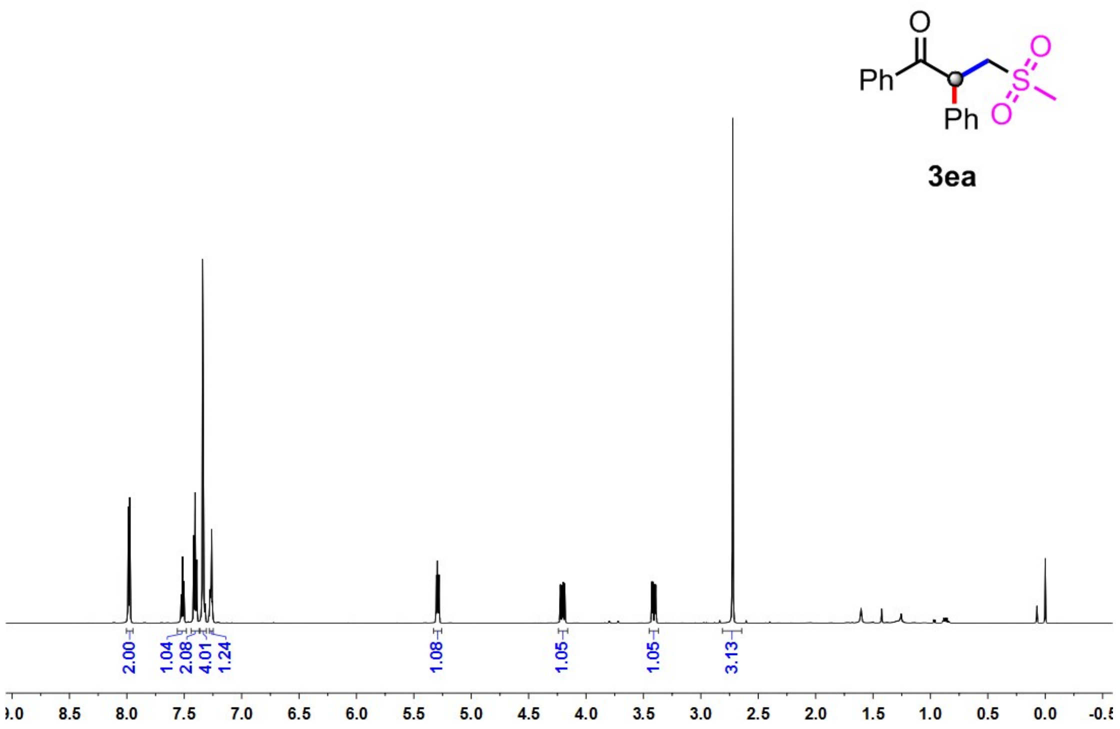
Ph
O
Ph
O
S
O
3ea
2.00
1.04
2.08
4.01
1.24
1.08
1.05
1.05
3.13
8.5
8.0
7.5
7.0
6.5
6.0
5.5
5.0
4.5
4.0
3.5
3.0
2.5
2.0
1.5
1.0
0.5
0.0

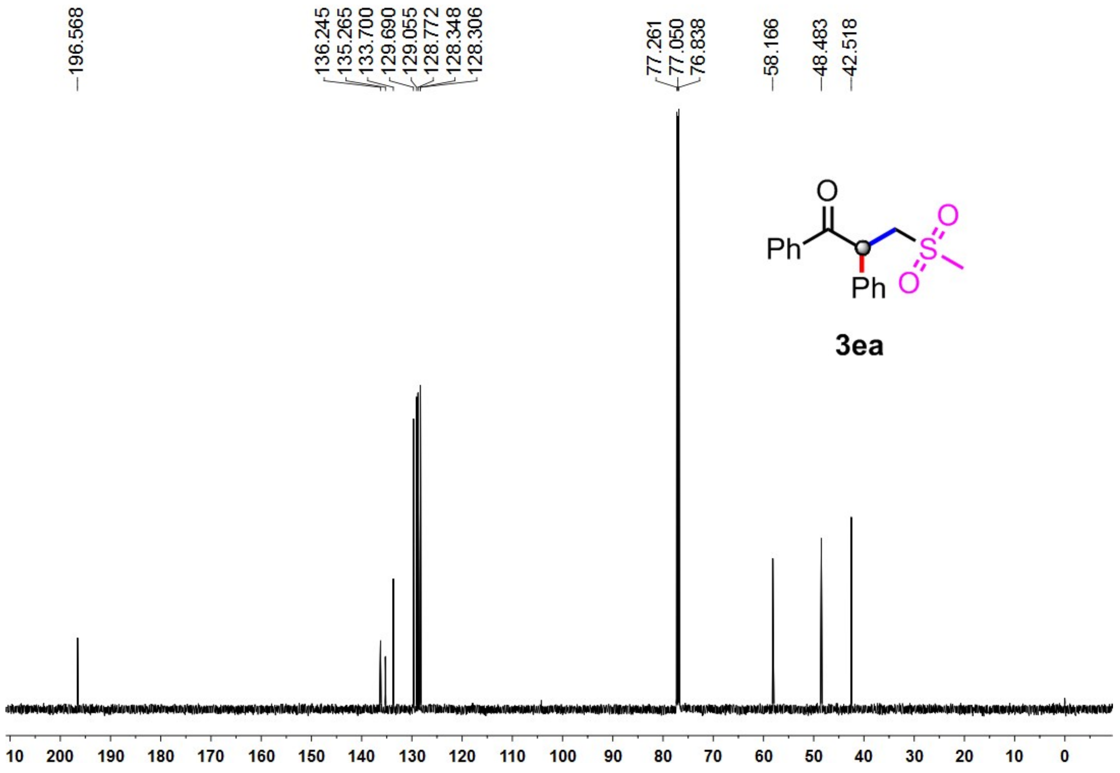
196.568
136.245
135.265
133.700
129.690
129.055
128.772
128.348
128.306
77.261
77.050
76.838
58.166
48.483
42.518
Ph
O
Ph
O
S
O
3ea
200
190
180
170
160
150
140
130
120
110
100
90
80
70
60
50
40
30
20
10
0

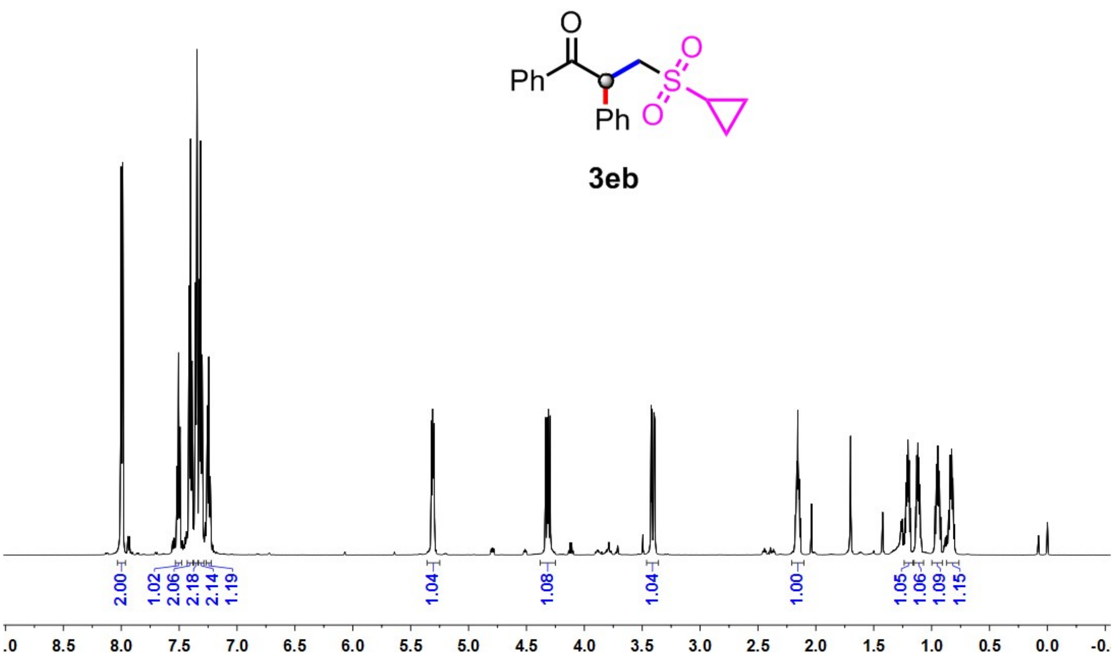
O
Ph
S
O
O
Ph
3eb
2.00
1.02
2.06
2.18
2.14
1.19
1.04
1.08
1.04
1.00
1.05
1.06
1.09
1.15
8.5
8.0
7.5
7.0
6.5
6.0
5.5
5.0
4.5
4.0
3.5
3.0
2.5
2.0
1.5
1.0
0.5
0.0

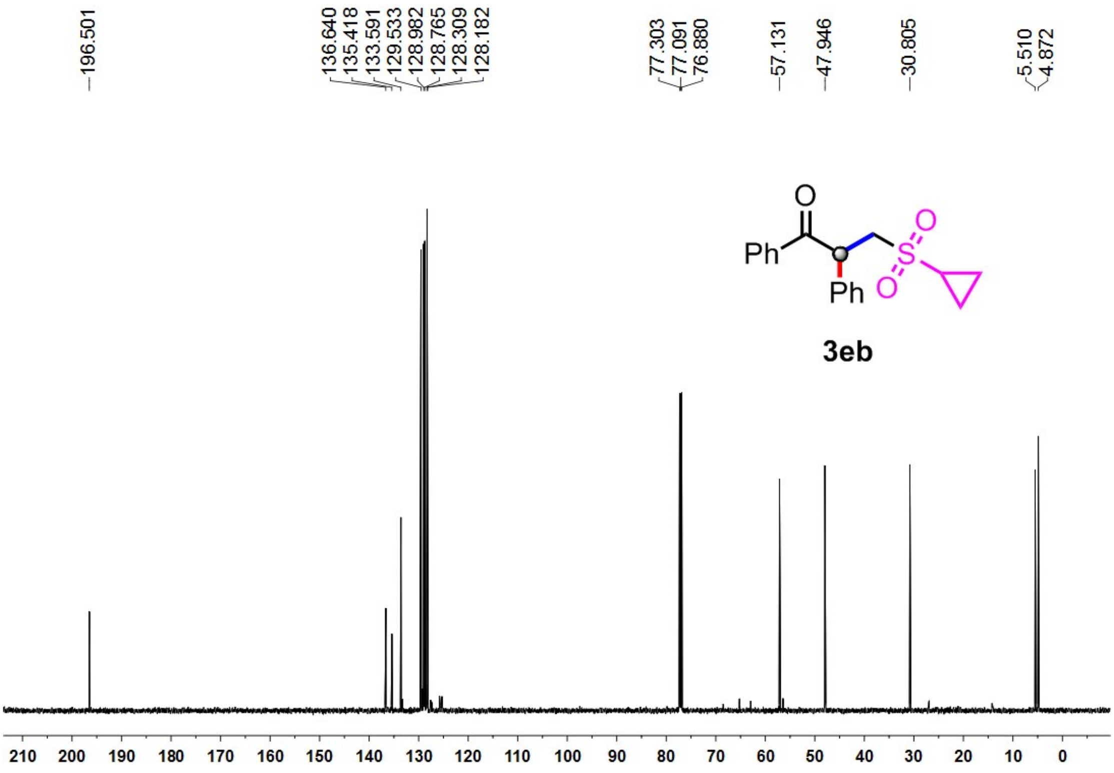
196.501
136.640
135.418
133.591
129.533
128.982
128.765
128.309
128.182
77.303
77.091
76.880
57.131
47.946
30.805
5.510
4.872
O
Ph
S
O
O
Ph
3eb
210
200
190
180
170
160
150
140
130
120
110
100
90
80
70
60
50
40
30
20
10
0

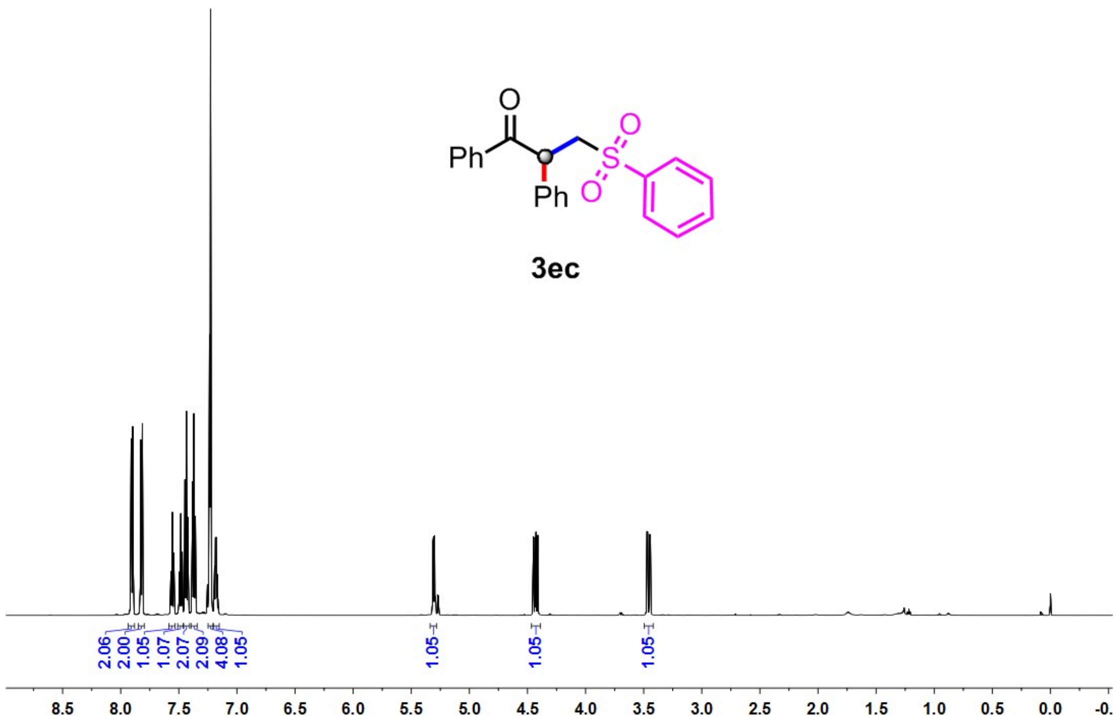

O
Ph
Ph
S
O
O
3ec
2.06
2.00
1.05
1.07
2.07
2.09
4.08
1.05
1.05
1.05
1.05
8.5 8.0 7.5 7.0 6.5 6.0 5.5 5.0 4.5 4.0 3.5 3.0 2.5 2.0 1.5 1.0 0.5 0.0 -0.

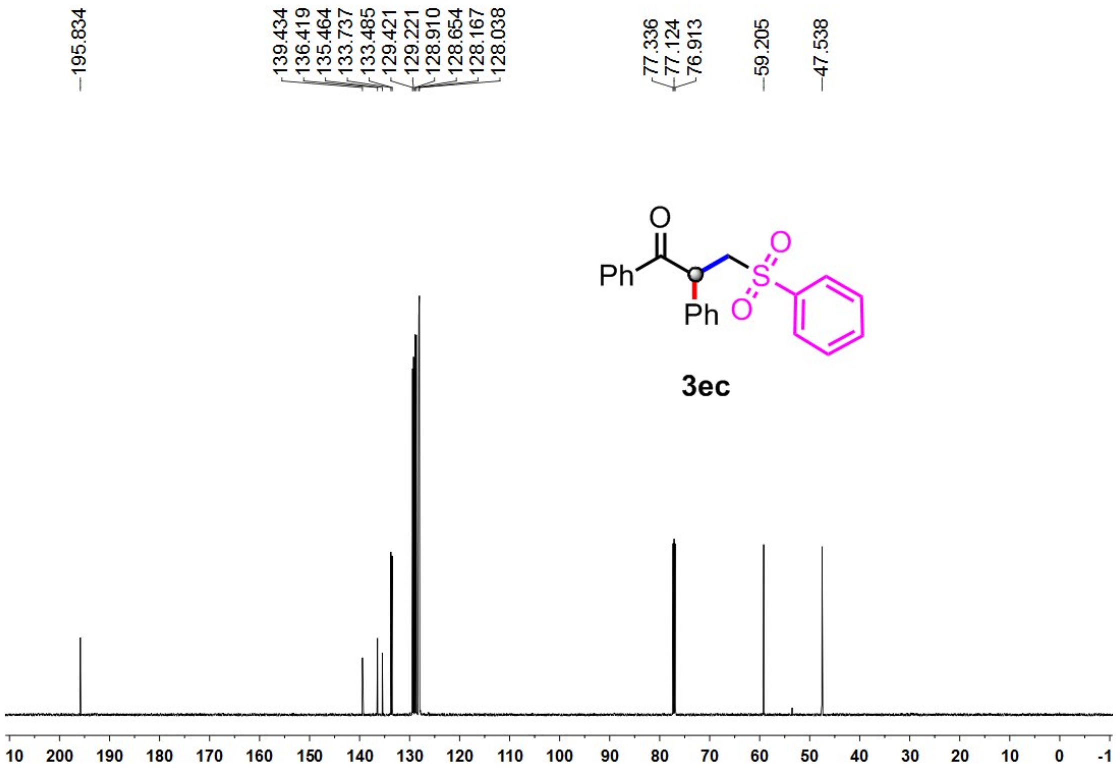

195.834
139.434
136.419
135.464
133.737
133.485
129.421
129.221
128.910
128.654
128.167
128.038
77.336
77.124
76.913
59.205
47.538
O
Ph
Ph
S
O
O
3ec
10 200 190 180 170 160 150 140 130 120 110 100 90 80 70 60 50 40 30 20 10 0 -1

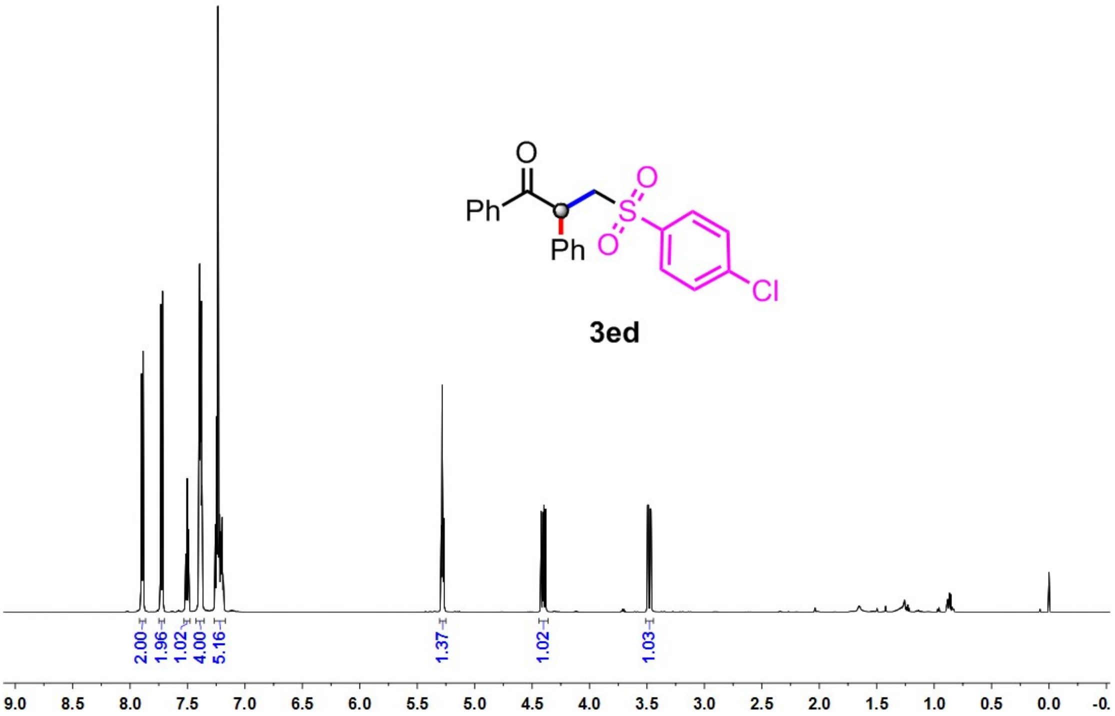
Ph
O
Ph
S
O
O
Cl
3ed
2.00
1.96
1.02
4.00
5.16
1.37
1.02
1.03
9.0 8.5 8.0 7.5 7.0 6.5 6.0 5.5 5.0 4.5 4.0 3.5 3.0 2.5 2.0 1.5 1.0 0.5 0.0 -0.

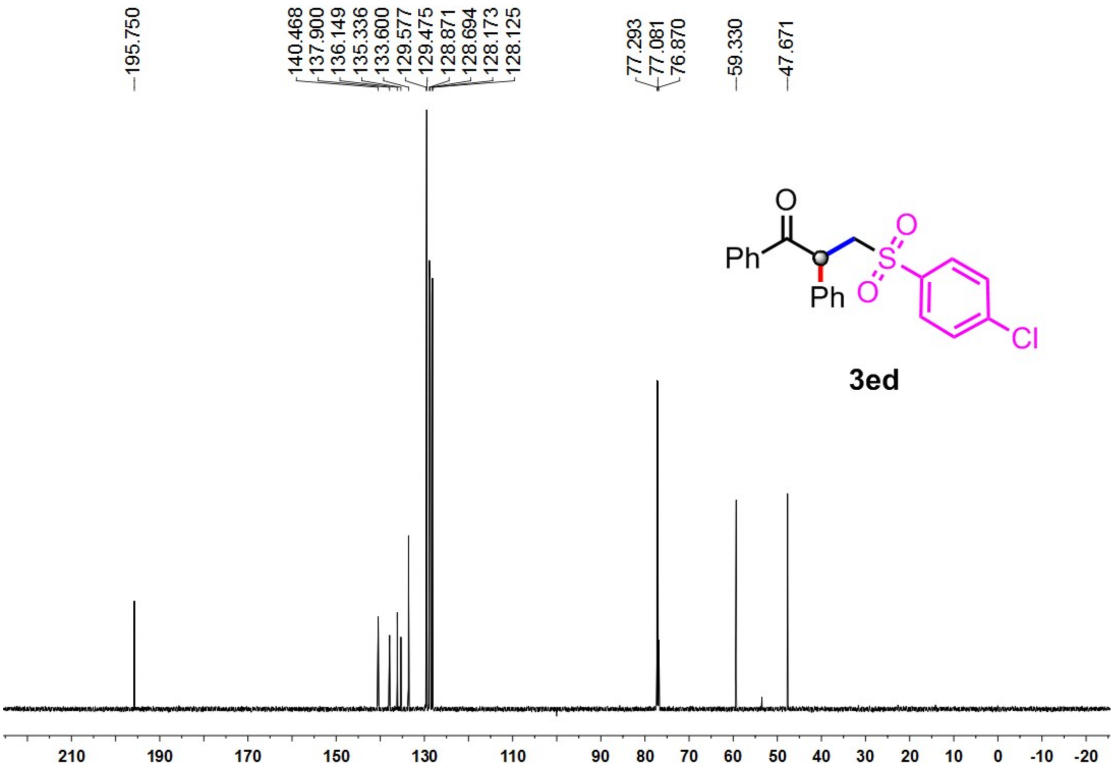
195.750
140.468
137.900
136.149
135.336
133.600
129.577
129.475
128.871
128.694
128.173
128.125
77.293
77.081
76.870
59.330
47.671
Ph
O
Ph
S
O
O
Cl
3ed
210 190 170 150 130 110 90 80 70 60 50 40 30 20 10 0 -10 -20

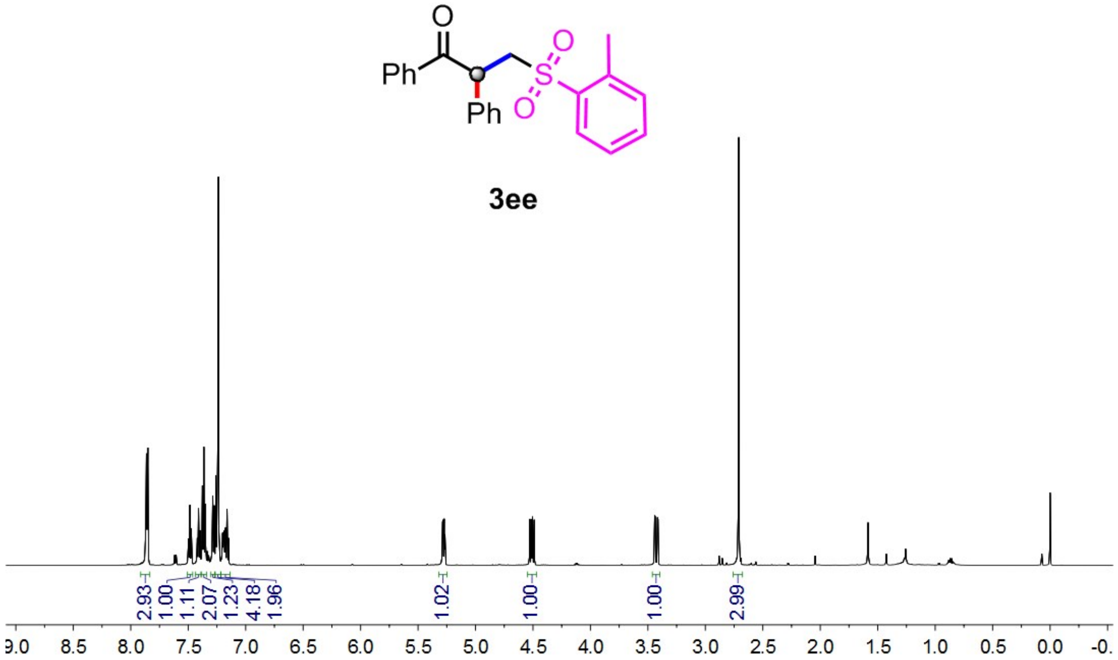
O
Ph
Ph
S
O
O
3ee
2.93
1.00
1.11
2.07
1.23
4.18
1.96
1.02
1.00
1.00
2.99
9.0 8.5 8.0 7.5 7.0 6.5 6.0 5.5 5.0 4.5 4.0 3.5 3.0 2.5 2.0 1.5 1.0 0.5 0.0 -0.

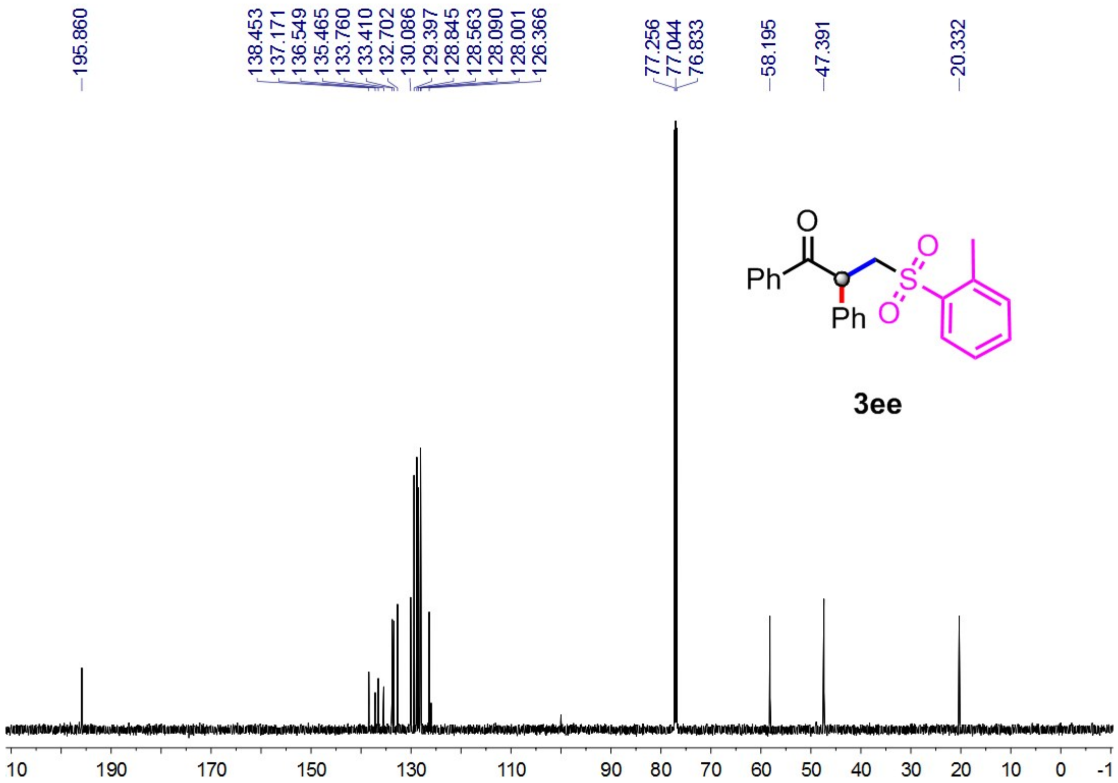
195.860
138.453
137.171
136.549
135.465
133.760
133.410
132.702
130.086
129.397
128.845
128.563
128.090
128.001
126.366
77.256
77.044
76.833
58.195
47.391
20.332
O
Ph
Ph
S
O
O
3ee
10 190 170 150 130 110 90 80 70 60 50 40 30 20 10 0 -1

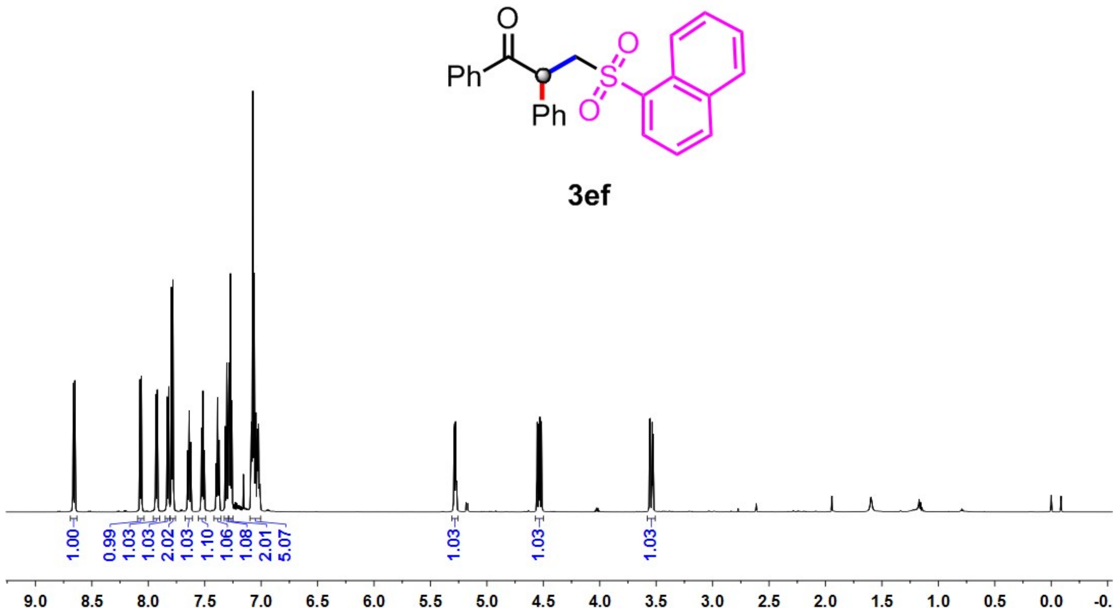
Ph
O
Ph
O
S
O
3ef
1.00
0.99
1.03
1.03
2.02
1.03
1.10
1.06
1.08
2.01
5.07
1.03
1.03
1.03
9.0 8.5 8.0 7.5 7.0 6.5 6.0 5.5 5.0 4.5 4.0 3.5 3.0 2.5 2.0 1.5 1.0 0.5 0.0 -0.

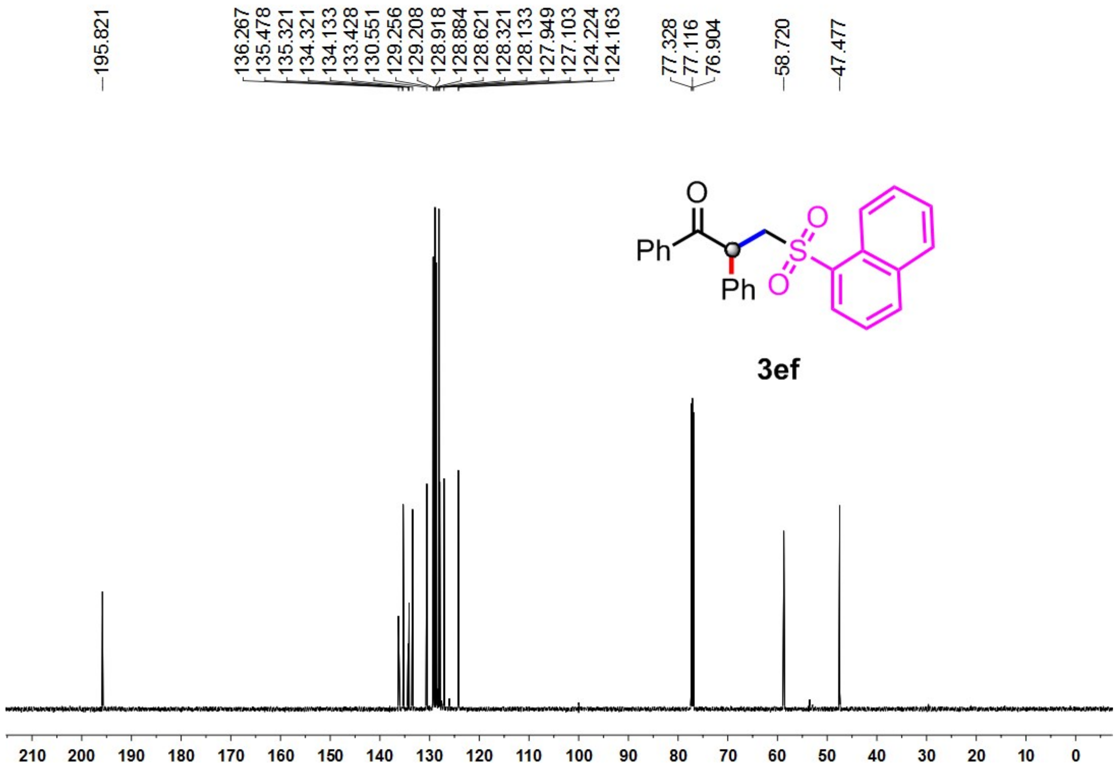
195.821
136.267
135.478
135.321
134.321
134.133
133.428
130.551
129.256
129.208
128.918
128.884
128.621
128.321
128.133
127.949
127.103
124.224
124.163
77.328
77.116
76.904
58.720
47.477
Ph
O
Ph
O
S
O
3ef
210 200 190 180 170 160 150 140 130 120 110 100 90 80 70 60 50 40 30 20 10 0

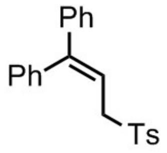

V-2

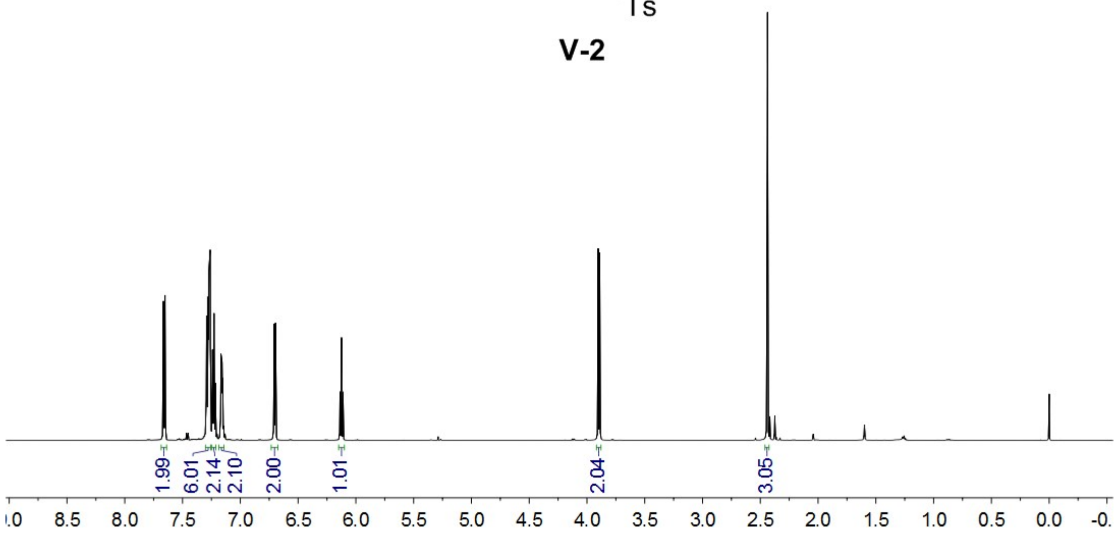
1.99
6.01
2.14
2.10
2.00
1.01
2.04
3.05
8.5 8.0 7.5 7.0 6.5 6.0 5.5 5.0 4.5 4.0 3.5 3.0 2.5 2.0 1.5 1.0 0.5 0.0

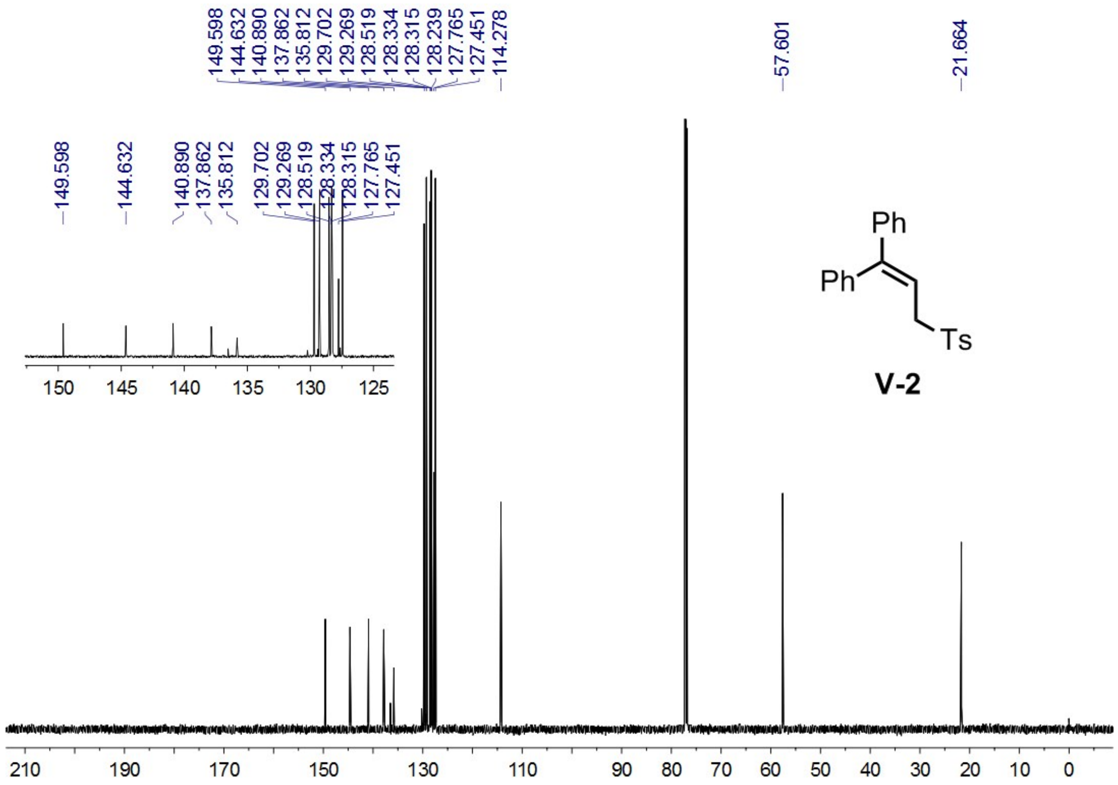
149.598
144.632
140.890
137.862
135.812
129.702
129.269
128.519
128.334
128.315
128.239
127.765
127.451
114.278
57.601
21.664
150 145 140 135 130 125
Ph
Ph
Ts
V-2
210 190 170 150 130 110 90 80 70 60 50 40 30 20 10 0

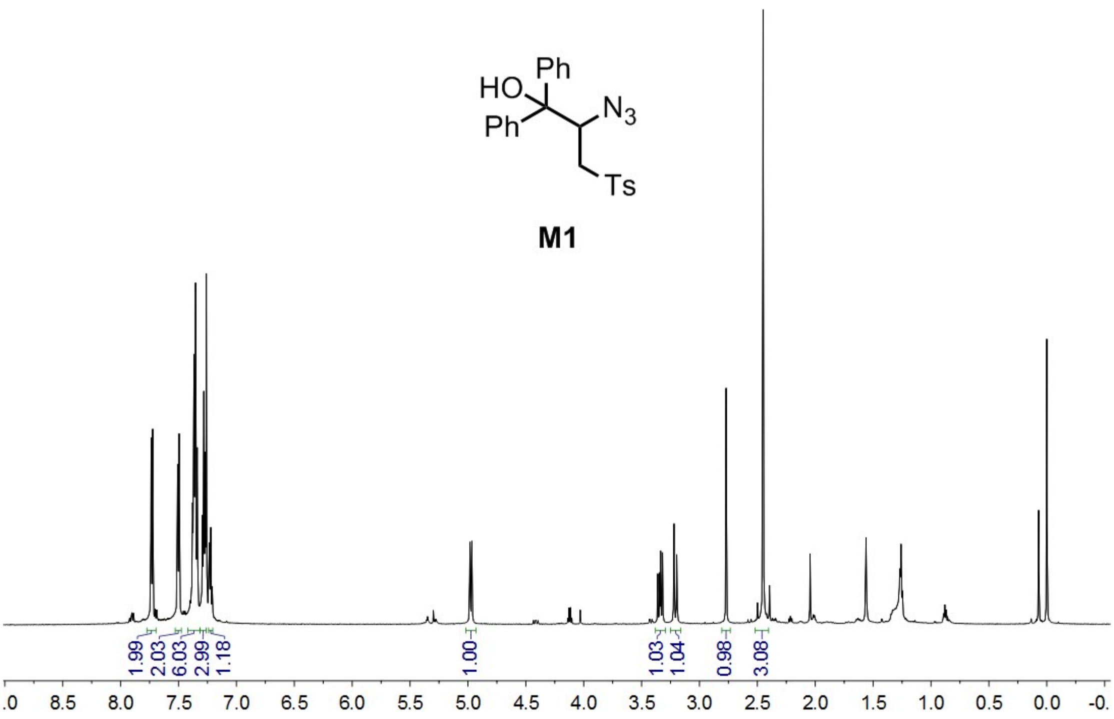
HO
Ph
Ph
N3
Ts
M1
1.99
2.03
6.03
2.99
1.18
1.00
1.03
1.04
0.98
3.08
8.5 8.0 7.5 7.0 6.5 6.0 5.5 5.0 4.5 4.0 3.5 3.0 2.5 2.0 1.5 1.0 0.5 0.0

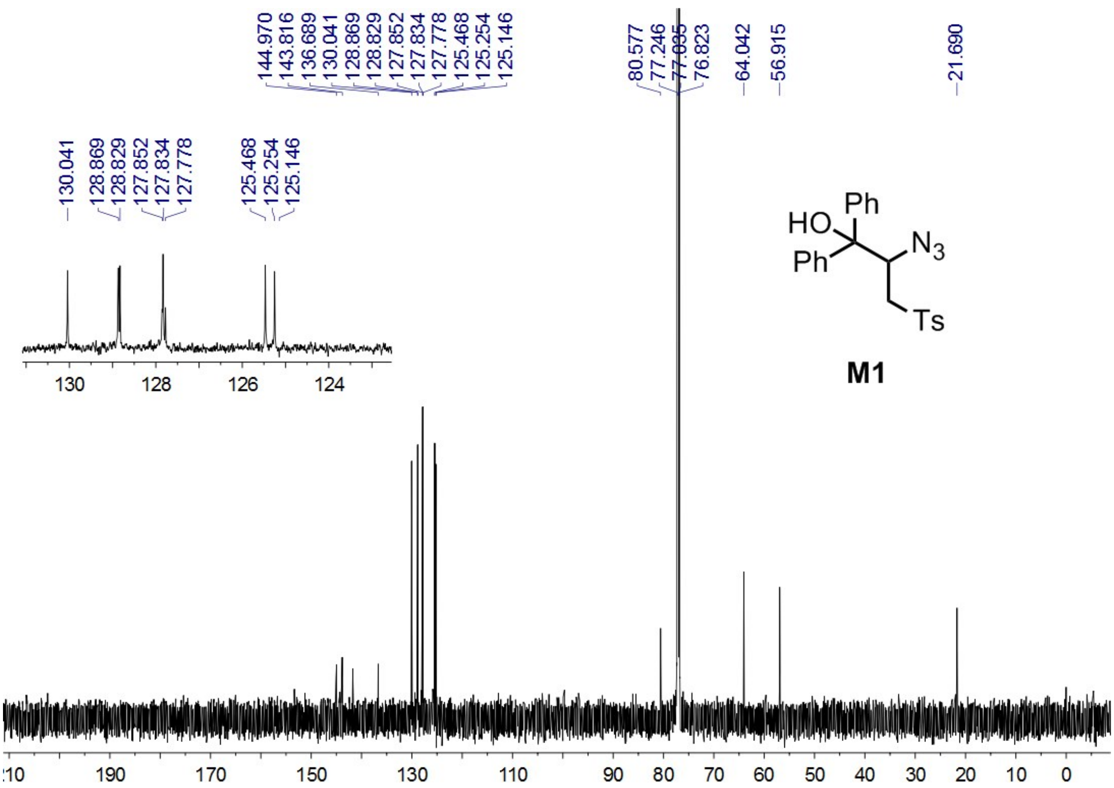
144.970
143.816
136.689
130.041
128.869
128.829
127.852
127.834
127.778
125.468
125.254
125.146
80.577
77.246
77.035
76.823
64.042
56.915
21.690
130.041
128.869
128.829
127.852
127.834
127.778
125.468
125.254
125.146
130 128 126 124
HO
Ph
Ph
N3
Ts
M1
190 170 150 130 110 90 80 70 60 50 40 30 20 10 0

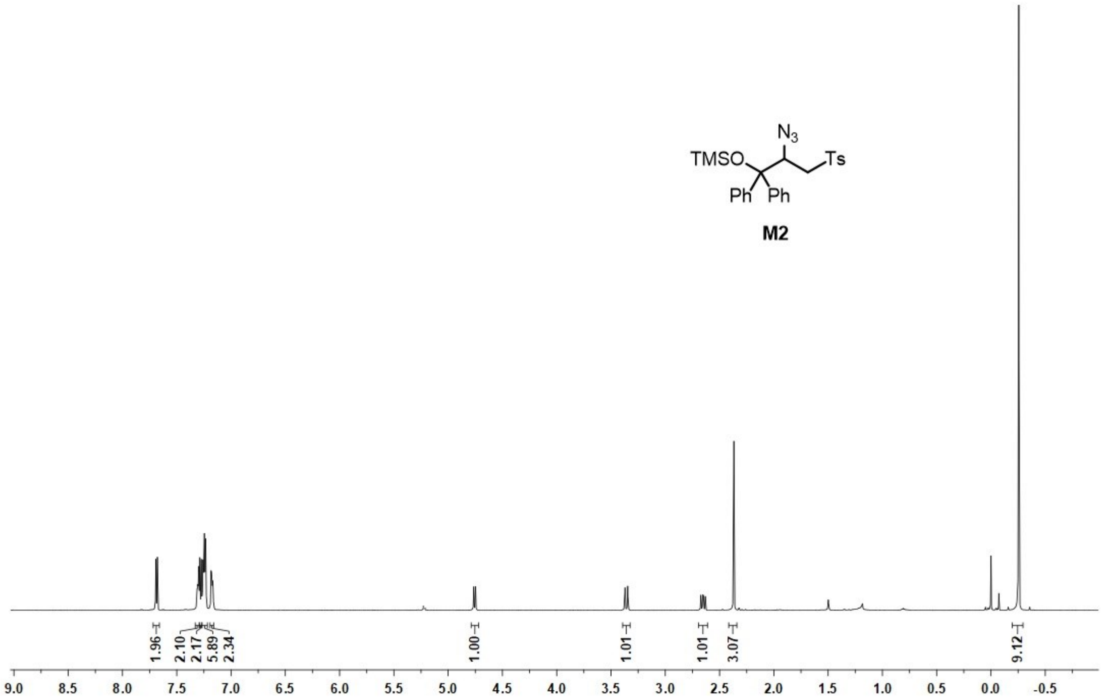
N3
TMSO
Ts
Ph
Ph
M2
1.96
2.10
2.17
5.89
2.34
1.00
1.01
1.01
3.07
9.12
9.0 8.5 8.0 7.5 7.0 6.5 6.0 5.5 5.0 4.5 4.0 3.5 3.0 2.5 2.0 1.5 1.0 0.5 0.0 -0.5

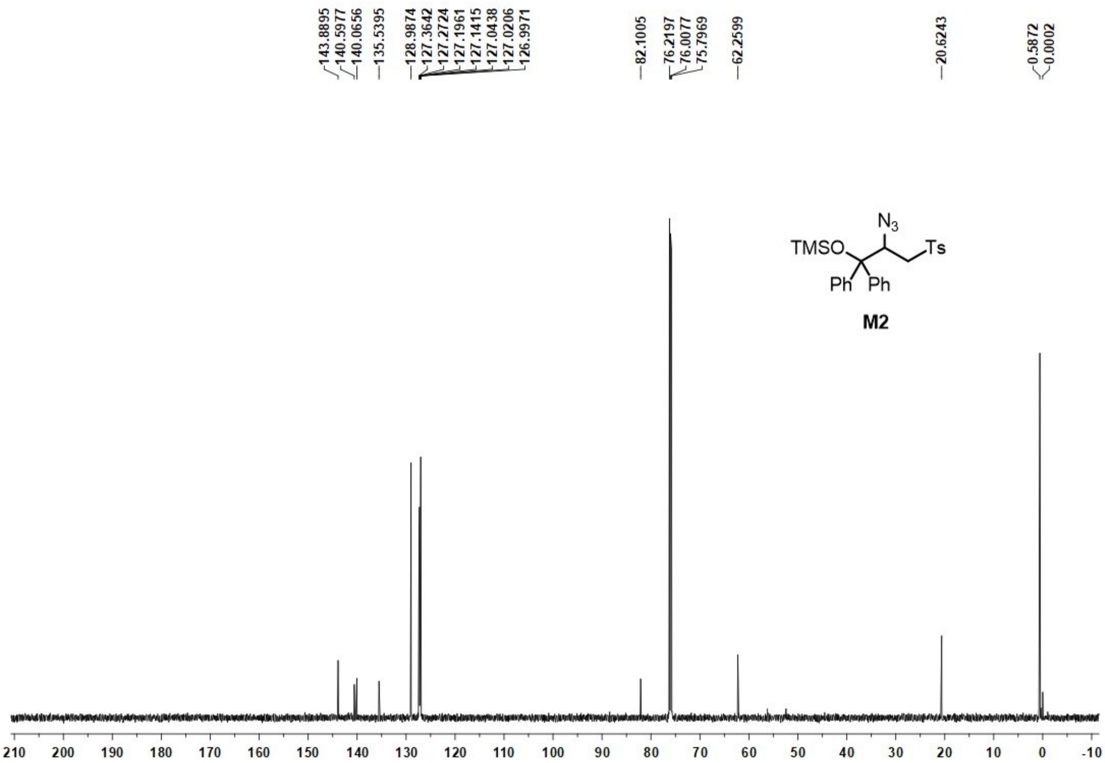
143.8895
140.5977
140.0656
135.5395
128.9874
127.3642
127.2724
127.1961
127.1415
127.0438
127.0206
126.9971
82.1005
76.2197
76.0077
75.7969
62.2599
20.6243
0.5872
0.0002
N3
TMSO
Ts
Ph
Ph
M2
210 200 190 180 170 160 150 140 130 120 110 100 90 80 70 60 50 40 30 20 10 0 -10

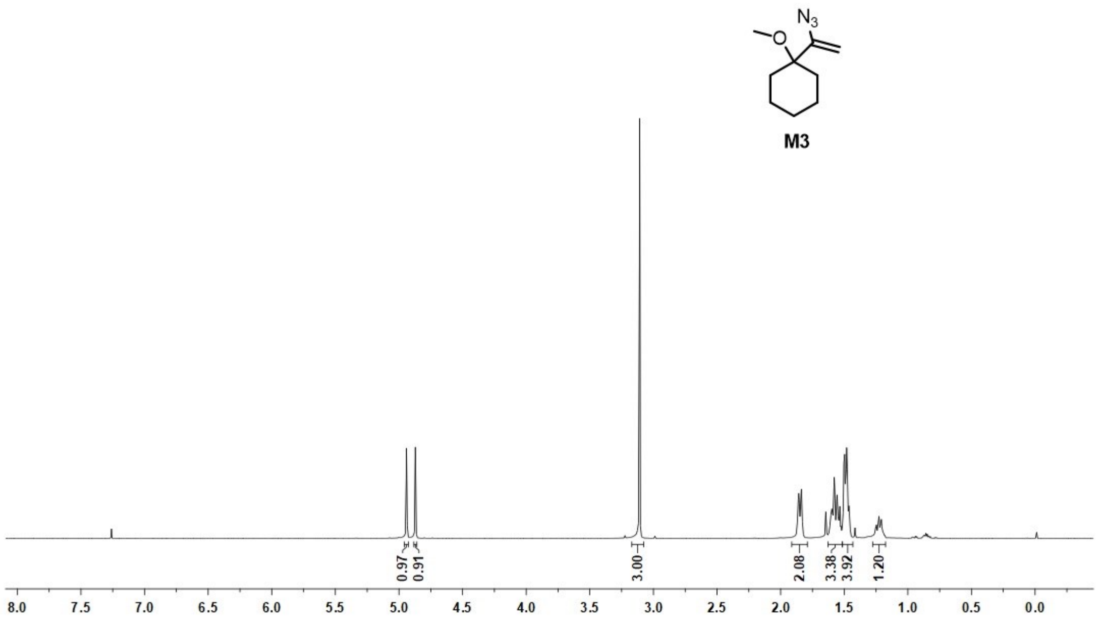
N3
O
M3
0.97
0.91
3.00
2.08
3.38
3.92
1.20
8.0
7.5
7.0
6.5
6.0
5.5
5.0
4.5
4.0
3.5
3.0
2.5
2.0
1.5
1.0
0.5
0.0

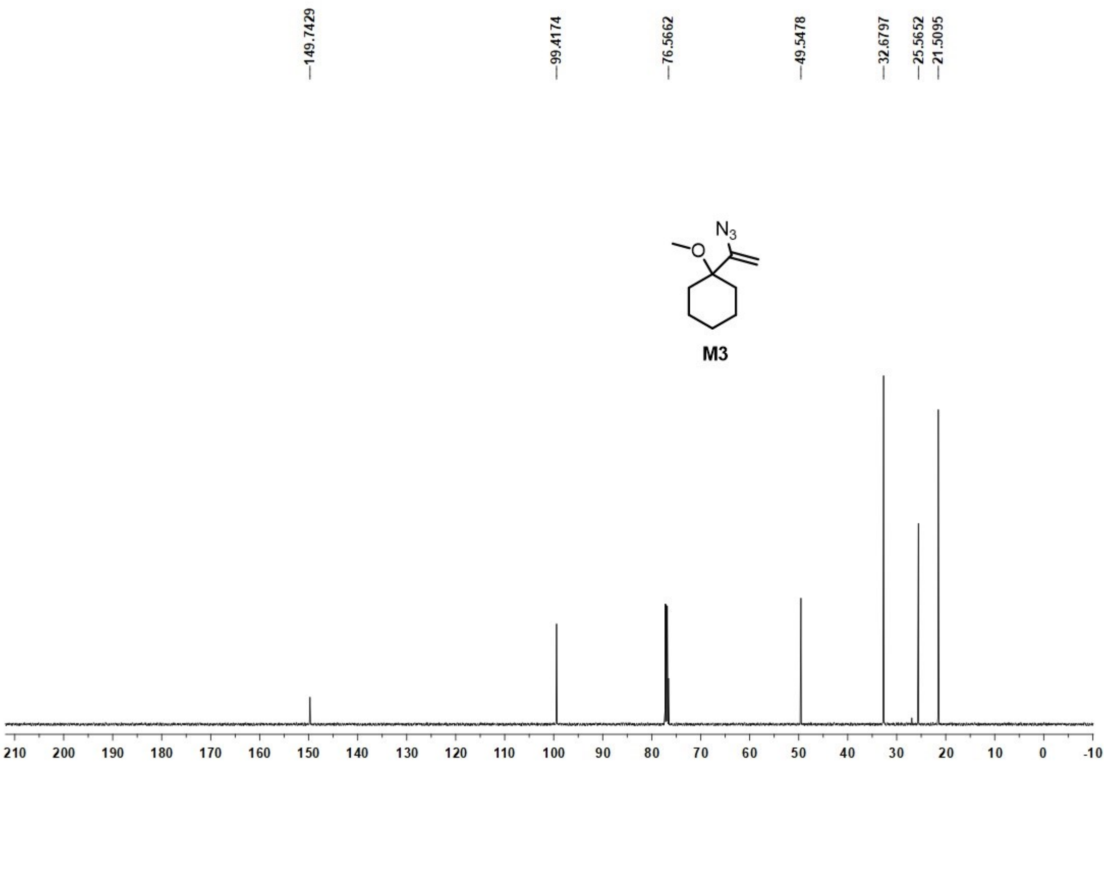
149.7429
99.4174
76.5662
49.5478
32.6797
25.5652
21.5095
N3
O
M3
210
200
190
180
170
160
150
140
130
120
110
100
90
80
70
60
50
40
30
20
10
0
-10

7. References

[1] Liu, Z.; Liu, J.; Zhang, L.; Liao, P.; Song, J.; Bi, X. Silver(I)-catalyzed hydroazidation of ethynyl carbinols: synthesis of 2-azidoallyl alcohols. *Angew. Chem. Int. Ed.* **2014**, *53*, 5305-5309.

[2] Liu, Z.; Liao, P.; Bi, X. General silver-catalyzed hydroazidation of terminal alkynes by combining $TMSN_3$ and H_2O: synthesis of vinyl azides. *Org. Lett.* **2014**, *16*, 3668-3671.

[3] Magnus, P.; Roe, M. B.; Hulme, C. New trialkylsilyl enol ether chemistry: direct 1,2-bis-azidonation of triisopropylsilyl enol ethers: an azido-radical addition process promoted by TEMPO. *J. Chem. Soc., Chem. Commun.* **1995**, 263-265.

[4] (a) Yang, Y.; Tang, L.; Zhang, S.; Guo, X.; Zha, Z.; Wang, Z. Catalyst-free sulfonylation of activated alkenes for highly efficient synthesis of mono-substituted ethyl sulfones in water. Green Chem. 2014, 16, 4106-4109; (b) Sreedhar, B.; Reddy, M. A.; Reddy, P. S. FeCl3/TMSCl: an effective catalytic system for the conjugate addition of sodium *p*-toluenesulfinate to *α,β*-enones. *Synlett.* **2008**, 1949-1952. (c) Bin, Y.; Hua, R. Synthesis of alkyl aryl sulfones *via* reaction of *N*-arylsulfonyl hydroxyamines with electron-deficient alkenes. *Molecules* **2017**, *22*, 39: doi.org/10.3390/molecules22010039.

[5] Chu, X.; Meng, H.; Xu, X.; Ji, S. One-pot synthesis of allylic sulfones, ketosulfones, and triflyl allylic alcohols from domino reactions of allylic alcohols with sulfinic acid under metal-free conditions. *Chem. Eur. J.* **2015**, *21*, 11359-11368.

[6] Sun, X.; Li, X.; Song, S.; Zhu, Y.; Liang, Y.; Jiao, N. Mn-catalyzed highly efficient aerobic oxidative hydroxyazidation of olefins: a direct approach to *β*-azido alcohols. *J. Am. Chem. Soc.* **2015**, *137*, 6059-6066.
